# Supplementary material for: C-SuFEx linkage of sulfonimidoyl fluorides and organotrifluoroborates
Source: Nat Commun. 2024 Jan 25;15:727. doi: 10.1038/s41467-024-44998-6 (PMC10810801; doi:10.1038/s41467-024-44998-6)
Supplement: Supplementary file 1 — Supplementary Information [file 41467_2024_44998_MOESM1_ESM.pdf]

C-SuFEx Linkage of Sulfonimidoyl Fluorides and Organotrifluoroborates

Suqin Zhao, Daming Zeng, Ming Wang* & Xuefeng Jiang*

Shanghai Key Laboratory of Green Chemistry and Chemical Process, School of Chemistry and Molecular Engineering, East China Normal University, 3663 North Zhongshan Road, Shanghai 200062, P. R. China.

Supplementary Information

Table of Contents

I. General Information S2

II. General procedure for the synthesis of sulfonimidoyl fluorides S3

III. General procedure for the synthesis of sulfoximines S6

IV. Characterization of the products S7

V. X-Ray Crystal Structure S34

VI. Fluorine spectrum tracking experiment S35

VII. Compuational details of DFT calculations S37

VIII. Bond heterolysis energy and binding energy S75

IX. NMR spectra S76

X. References S128

I. General Information.

^{1}H, ^{13}C and ^{19}F NMR spectra were recorded on 400 MHz NMR spectrometers (Bruker AVANCE) using $CDCl_3$, CD_3CN. Chemical shifts are reported in parts per million (ppm). Chemical shifts for protons are reported in parts per million relative to chloroform (δ 7.26). Chemical shifts for carbon are reported in parts per million relative to chloroform (δ 77.0). Data are represented as follows: chemical shift, multiplicity (s = singlet, d = doublet, t = triplet, q = quartet, m = multiplet), coupling constants in Hertz (Hz), integration. Mass spectra were recorded on a Shimadzu GCMS-QP2010 Ultra. IR spectra were recorded on TENSOR (27) Series FT-IR 241.Spectrometers. Column chromatography was performed with silica gel (300-400 mesh ASTM). Sulfonimiodyl fluoride **1**[1-2] and potassium aryltrifluoroborate salt **2**[3] were respectively obtained by reported procedures.

II. General procedure for the synthesis of sulfonimidoyl fluorides.

Synthesis of sulfinamides [1-2]:

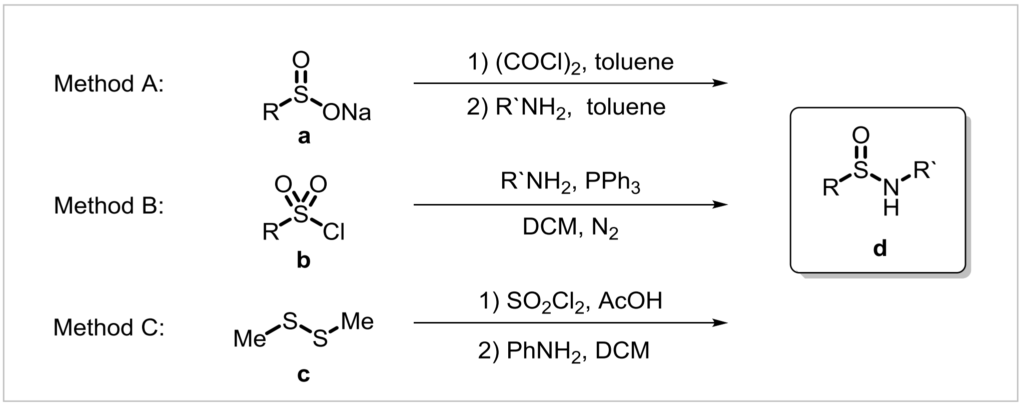

Supplementary Figure 1. Procedure for the synthesis of sulfinamides

Method A:

$(COCl)_2$ (11.0 mol, 1.1 equiv.) was added dropwise into a 50 mL flame-dried Schlenk flask at 0 °C, which was equipped with sodium sulfinate **a** (10.0 mmol, 1.0 equiv.) and anhydrous toluene (25.0 mL, 0.4 M). After dropping, the reaction solution was transferred to room temperature for 1 h to form the sulfinyl chloride. Then the reaction solution was added dropwise to the toluene (25.0 mL, 0.4 M) solution of triethylamine (15.0 mmol, 1.5 equiv.) and amine (13.0 mmol, 1.3 equiv.) in another 100 mL Schlenk flask at 0 °C. The mixture was stirred for 1 h at room temperature and quenched with water. After the extraction of ethyl acetate for three times, the organic phase was washed with brine and dried over anhydrous Na_2SO_4. The solvent was evaporated under reduced pressure, and the residue was purified by column chromatography to yield the corresponding sulfinamide **d**.

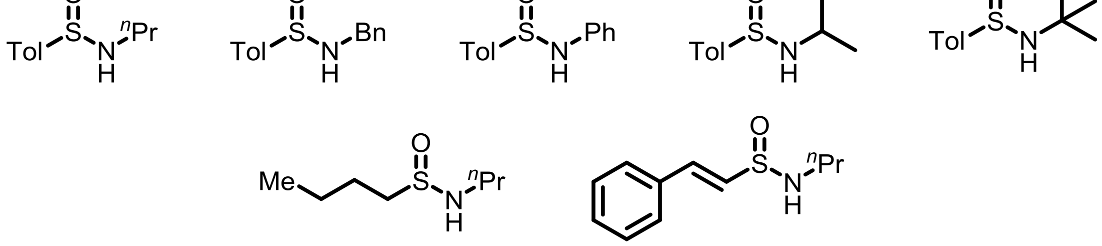

Supplementary Figure 2. The prepared sulfinamides via method A

Method B:

Under nitrogen atmosphere, a DCM (25.0 mL, 0.4 M) solution of amine (10.0 mmol, 1.0 equiv.), triphenylphosphine (10.0 mmol, 1.0 equiv.) and triethylamine (20.0 mmol, 2.0 equiv.) was added dropwise via syringe pump (8 mL/h) to a solution of sulfonyl chloride **b** (10.0 mmol, 1.0 equiv.) in anhydrous DCM (25.0 mL, 0.4 M) at 0 ^{o}C. The mixture was stirred for 18 h at 0 ^{o}C. After TLC monitoring reaction, the solvent was evaporated under reduced pressure, and the residue was purified by column chromatography to yield the corresponding sulfinamide **d**.

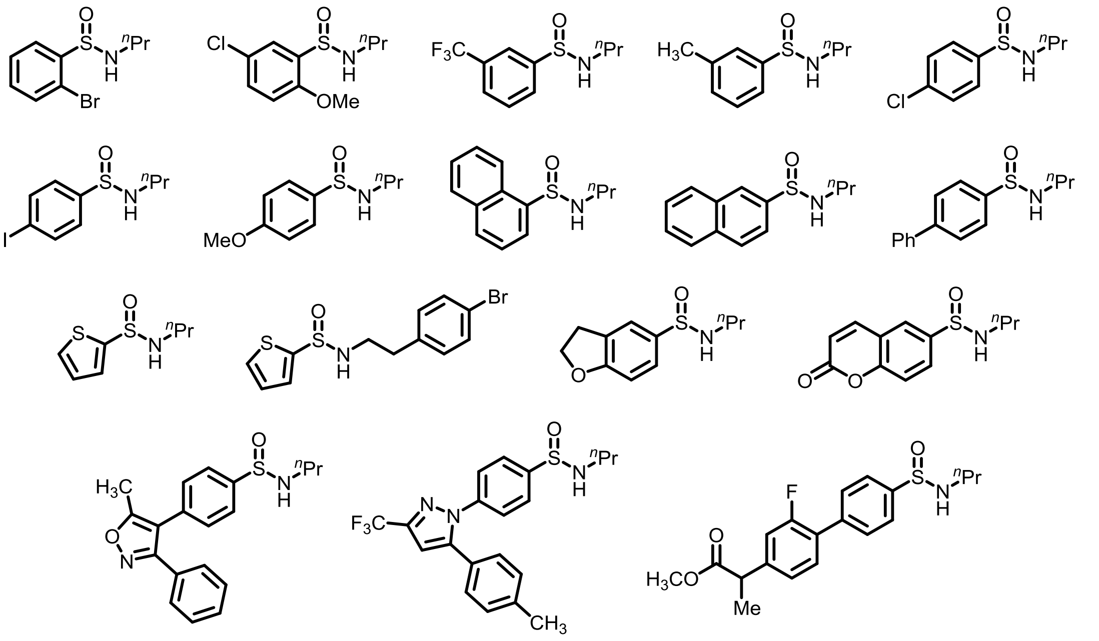

Supplementary Figure 3. The prepared sulfinamides via method B

Method C:

SO_2Cl_2 (15.5 mmol, 3.1 equiv.) was added dropwise into a dry 25 mL oven-dried Schlenk tube, which was equipped with dimethyl disulfide **c** (5 mmol, 1 equiv.) and acetic acid (10 mmol, 2 equiv.) at -20 ^{o}C. The mixture was then stirred at -20 ^{o}C for 3 h and then warmed to rt for 2 h. Subsequently, the reaction mixture was transferred to 35 ^{o}C for 1 h. After completion, the volatiles were evaporated under reduced pressure and the resulting methylsulfinyl

chloride was directly used for the next step without purification. The DCM (15.0 mL, 0.67 M) solution of methylsulfinyl chloride was slowly added into a solution of aniline (10.0 mmol, 2 equiv.) in dry DCM (10 mL, 1 M). Then the mixture was warmed to room temperature for 3 h and quenched with water. After the extraction of DCM for three times, the organic phase was washed with brine and dried over anhydrous Na_2SO_4. The solvent was evaporated under reduced pressure, and the residue was purified by column chromatography to yield the corresponding *N*- phenylmethane sulfinamide.

Synthesis of sulfonimidoyl fluorides [1-2]:

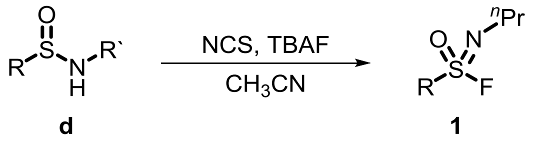

Supplementary Figure 4. Procedure for the synthesis of sulfonimidoyl fluorides

NCS (5.5 mmol, 1.1 equiv.) was added portionwise into a dry 100 mL round-bottom flask, which was equipped with sulfonamide **d**, TBAF (5.5 mL, 1 M in THF, 1.1 equiv.), CH_3CN (40 mL, 0.125 M) and a stirring bar at 0 ^{o}C. The mixture was warmed to room temperature for 30 min. After TLC monitoring reaction, the solvent was evaporated under reduced pressure, and the residue was purified by column chromatography to yield the corresponding sulfonimidoyl fluorides **1**.

III. General procedure for the synthesis of sulfoximines

Under nitrogen atmosphere, dry CH_3CN (2 mL, 0.2 M) was added to a 10 mL oven-dried Schlenk tube, which was equipped with sulfonimidoyl fluoride **1** (0.60 mmol, 1.5 equiv.), potassium organotrifluoroborate salt **2** (0.40 mmol, 1.0 equiv.) and a stirring bar. TMSOTf (0.08 mmol, 0.2 equiv.) was dropwise added via the microsyringe at room temperature. Then the reaction mixture was stirred at 40 ^{o}C for 30 min and quenched with saturated aqueous solution of sodium carbonate. After the extraction of DCM for three times, the organic phase was washed with brine and dried over anhydrous Na_2SO_4. The solvent was evaporated under reduced pressure, and the residue was purified by column chromatography to yield the corresponding sulfoximine product **3**.

IV. Characterization of the products

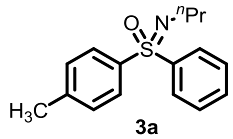
3a

Phenyl(propylimino)(*p*-tolyl)-λ^6-sulfanone (3a). Prepared following general procedure using 4-methyl-*N*-propylbenzenesulfonimidoyl fluoride **1a** (0.6 mmol, 129.2 mg), potassium trifluoro(phenyl)borate **2a** (0.4 mmol, 73.6 mg), TMSOTf (0.08 mmol, 14.4 µL) and CH_3CN (2.0 mL), the reaction was stirred at 40 °C for 30 min giving **3a** in 95% yield (102.7 mg) as a white solid by column chromatography (PE/EA = 5:1, R_f = 0.49). **^{1}H NMR** (400 MHz, $CDCl_3$) δ 7.96 (m, 2H), 7.85 (d, *J* = 8.3 Hz, 2H), 7.50 – 7.39 (m, 3H), 7.25 (d, *J* = 8.2 Hz, 2H), 3.00 (t, *J* = 7.1 Hz, 2H), 2.36 (s, 3H), 1.74 – 1.58 (m, 2H), 0.95 (t, *J* = 7.4 Hz, 3H). **^{13}C NMR** (101 MHz, $CDCl_3$) δ 142.9, 141.1, 137.7, 132.0, 129.7, 128.9, 128.5, 128.3, 45.6, 26.0, 21.3, 11.8. **IR** (film) ν 2960, 2870, 1712, 1593, 1249, 1078, 814, 711, 634, 615, 588 cm^{-1}. **HRMS** (ESI) m/z: $[M+H]^+$ Calcd for $C_{16}H_{20}NOS$ 274.1260, found 274.1252.

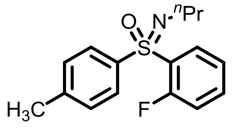
3b

(2-fluorophenyl)(propylimino)(*p*-tolyl)-λ^6-sulfanone (**3 b**). Prepared following general procedure using 4-methyl-*N*-propylbenzenesulfonimidoyl fluoride **1a** (0.6 mmol, 129.2 mg), potassium trifluoro(2-fluorophenyl) borate **2b** (0.4 mmol, 80.8 mg), TMSOTf (0.08 mmol, 14.4 µL) and CH_3CN (2.0 mL), the reaction was stirred at 40 °C for 30 min giving **3b** in 83% yield (96.4 mg) as a colorless oil by column chromatography (PE/EA = 5:1, R_f = 0.41). **^{1}H NMR** (400 MHz, $CDCl_3$) δ 8.15 – 8.09 (m, 1H), 7.97 (d, *J* = 8.0 Hz, 2H), 7.53 – 7.46 (m, 1H), 7.31 – 7.26 (m, 3H), 7.08 – 7.01 (m, 1H), 3.12 – 3.04 (m, 1H), 3.02 – 2.93 (m, 1H), 2.39 (s, 3H), 1.71 – 1.61 (m, 2H), 0.96 (t, *J* = 7.3 Hz, 3H). **^{13}C NMR** (101 MHz, $CDCl_3$) δ 158.9 (d, *J* = 255.7 Hz), 143.4, 137.3, 134.6 (d, *J* = 8.4 Hz), 131.7, 129.4, 128.9 (d, *J* = 2.1 Hz), 128.5 (d, *J* = 13.7 Hz), 124.4 (d, *J* = 4.0 Hz), 117.0 (d, *J* = 22.1 Hz), 45.9, 25.9, 21.4, 11.7. **^{19}F NMR** (376 MHz, $CDCl_3$) δ -108.0. **IR** (film) ν 2958, 2926, 1595, 1446, 1259, 1143, 1093, 1064,

761, 715 cm^{-1}. **HRMS** (ESI) m/z: $[M+H]^+$ Calcd for $C_{16}H_{19}FNOS$ 292.1166, found 292.1161.

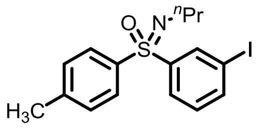

(3-iodophenyl)(propylimino)(*p*-tolyl)-λ^6-sulfanone (**3c**). Prepared following general procedure using 4-methyl-*N*-propylbenzenesulfonimidoyl fluoride **1a** (0.6 mmol, 129.2 mg), potassium trifluoro(3-iodophenyl) borate **2c** (0.4 mmol, 124.0 mg), TMSOTf (0.2 mmol, 36 µL) and CH_3CN (2.0 mL), the reaction was stirred at rt for 30 min giving **3c** in 71% yield (113.0 mg) as a colorless oil by column chromatography (PE/EA = 5:1, R_f = 0.39). **^{1}H NMR** (400 MHz, $CDCl_3$) δ 8.38 (s, 1H), 7.97 (d, *J* = 7.9 Hz, 1H), 7.92 (d, *J* = 8.1 Hz, 2H), 7.88 (d, *J* = 7.9 Hz, 1H), 7.36 (d, *J* = 8.1 Hz, 2H), 7.29 – 7.23 (m, 1H), 3.14 – 3.01 (m, 2H), 2.46 (s, 3H), 1.80 – 1.69 (m, 2H), 1.05 (t, *J* = 7.4 Hz, 3H). **^{13}C NMR** (101 MHz, $CDCl_3$) δ 143.3, 143.2, 140.9, 137.2, 136.8, 130.5, 129.8, 128.5, 127.4, 94.2, 45.6, 26.0, 21.4, 11.8. **IR** (film) ν 2957, 2922, 1448, 1396, 1224, 1139, 1057, 1041, 682, 590 cm^{-1}. **HRMS** (ESI) m/z: $[M+H]^+$ Calcd for $C_{16}H_{19}INOS$ 400.0227, found 400.0218.

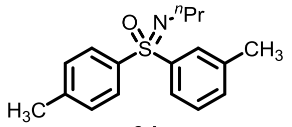

(Propylimino)(*m*-tolyl)(*p*-tolyl)-λ^6-sulfanone (**3d**). Prepared following general procedure using 4-methyl-*N*-propylbenzenesulfonimidoyl fluoride **1a** (0.6 mmol, 129.2 mg), potassium trifluoro(*m*-tolyl)borate **2d** (0.4 mmol, 78.4 mg), TMSOTf (0.2 mmol, 36 µL) and CH_3CN (2.0 mL), the reaction was stirred at rt for 30 min giving **3d** in 92% yield (106.1 mg) as a colorless oil by column chromatography (PE/EA = 5:1, R_f = 0.38). **^{1}H NMR** (400 MHz, $CDCl_3$) δ 7.86 (d, *J* = 7.8 Hz, 2H), 7.81 – 7.74 (m, 2H), 7.39 – 7.31 (m, 1H), 7.31 – 7.24 (m, 3H), 3.02 (t, *J* = 7.2 Hz, 2H), 2.38 (s, 3H) 2.39 (s, 3H), 1.75 – 1.63 (m, 2H), 0.98 (t, *J* = 7.4 Hz, 3H). **^{13}C NMR** (101 MHz, $CDCl_3$) δ 142.8, 141.0, 139.0, 137.9, 132.8, 129.6, 128.7, 128.5(9), 128.5(1), 125.4, 45.7, 26.1, 21.3, 21.2, 11.8. **IR** (film) ν 2957, 2926, 1595, 1475, 1452, 1228, 1141, 788, 696 cm^{-1}. **HRMS** (ESI) m/z: $[M+H]^+$ Calcd for

$C_{17}H_{22}NOS$ 288.1417, found 288.1408.

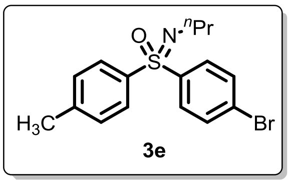

(4-bromophenyl)(propylimino)(*p*-tolyl)-λ^6-sulfanone (**3e**). Prepared following general procedure using 4-methyl-*N*-propylbenzenesulfonimidoyl fluoride **1a** (0.6 mmol, 129.2 mg), potassium (4-bromophenyl)trifluoroborate **2e** (0.4 mmol, 104.8 mg), TMSOTf (0.08 mmol, 14.4 µL) and CH_3CN (2.0 mL), the reaction was stirred at 40 °C for 30 min giving **3e** in 79% yield (110.8 mg) as a colorless oil by column chromatography (PE/EA = 5:1, R_f = 0.49). **^{1}H NMR** (400 MHz, $CDCl_3$) δ 7.81 (m, 4H), 7.57 (d, *J* = 8.5 Hz, 2H), 7.26 (d, *J* = 8.1 Hz, 2H), 3.02 – 2.94 (m, 2H), 2.37 (s, 3H), 1.71 – 1.59 (m, 2H), 0.95 (t, *J* = 7.3 Hz, 3H). **^{13}C NMR** (101 MHz, $CDCl_3$) δ 143.3, 140.4, 137.3, 132.2, 130.0, 129.8, 128.5, 127.1, 45.6, 26.0, 21.4, 11.8. **IR** (film) ν 2957, 2870, 1737, 1570, 1467, 1228, 1064, 1008, 742, 665 cm^{-1}. **HRMS** (ESI) m/z: $[M+H]^+$ Calcd for $C_{16}H_{19}BrNOS$ 352.0522; 354.0335, found 352.0365; 354.0358.

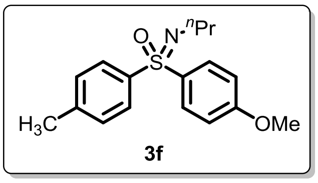

(4-methoxyphenyl)(propylimino)(*p*-tolyl)-λ^6-sulfanone (**3f**). Prepared following general procedure using 4-methyl-*N*-propylbenzenesulfonimidoyl fluoride **1a** (0.6 mmol, 129.2 mg), potassium trifluoro(4-methoxyphenyl)borate **2f** (0.4 mmol, 85.6 mg), TMSOTf (0.08 mmol, 14.4 µL) and CH_3CN (2.0 mL), the reaction was stirred at 40 °C for 30 min giving **3f** in 62% yield (71.5 mg) as a colorless oil by column chromatography (PE/EA = 5:1, R_f = 0.21). **^{1}H NMR** (400 MHz, $CDCl_3$) δ 7.88 (d, *J* = 8.9 Hz, 2H), 7.81 (d, *J* = 8.0 Hz, 2H), 7.23 (d, *J* = 8.1 Hz, 2H), 6.91 (d, *J* = 8.8 Hz, 2H), 3.80 (s, 3H), 2.97 (t, *J* = 7.1 Hz, 2H), 2.35 (s, 3H), 1.73 – 1.59 (m, 2H), 0.95 (t, *J* = 7.3 Hz, 3H). **^{13}C NMR** (101 MHz, $CDCl_3$) δ 162.5, 142.6, 138.4, 132.5, 130.4, 129.6, 128.2, 114.2, 55.4, 45.7, 26.1, 21.3, 11.8. **IR** (film) ν 2958, 2926, 1591, 1492, 1307, 1253, 1222, 1095, 1026, 754 cm^{-1}. **HRMS** (ESI) m/z: $[M+H]^+$ Calcd for $C_{17}H_{22}NO_2S$ 304.1366, found 304.1358.

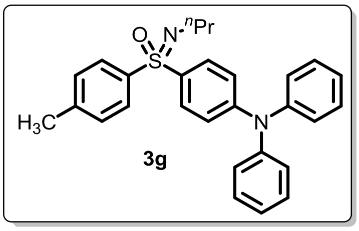

(4-(diphenylamino)phenyl)(propylimino)(*p*-tolyl)-λ^6-sulfanone (**3g**). Prepared following general procedure using 4-methyl-*N*-propylbenzenesulfonimidoyl fluoride **1a** (0.6 mmol, 129.2 mg), potassium (4-(diphenylamino)phenyl)trifluoroborate **2g** (0.4 mmol, 140.0 mg), TMSOTf (0.2 mmol, 36 μL) and CH_3CN (2.0 mL), the reaction was stirred at rt for 30 min giving **3g** in 66% yield (117.0 mg) as a colorless oil by column chromatography (PE/EA = 5:1, R_f = 0.32). **^{1}H NMR** (400 MHz, $CDCl_3$) δ 7.93 (d, *J* = 8.3 Hz, 2H), 7.86 – 7.81 (m, 2H), 7.41 – 7.33 (m, 6H), 7.23 – 7.17 (m, 6H), 7.09 – 7.05 (m, 2H), 3.21 – 2.99 (m, 2H), 2.47 (s, 3H), 1.84 – 1.67 (m, 2H), 1.06 (t, *J* = 7.3 Hz, 3H). **^{13}C NMR** (101 MHz, $CDCl_3$) δ 151.2, 146.3, 142.4, 138.5, 131.7, 129.6, 129.5, 128.3, 125.8, 124.5, 120.0, 45.7, 26.1, 21.3, 11.8. **IR** (film) ν 3061, 2957, 2870, 1579, 1487, 1267, 1224, 1095, 1087 754, 675 cm^{-1}. **HRMS** (ESI) m/z: $[M+H]^+$ Calcd for $C_{28}H_{29}N_2OS$ 441.1995, found 441.1987.

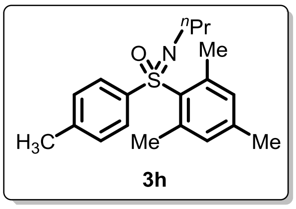

Mesityl(propylimino)(*p*-tolyl)-λ^6-sulfanone (**3h**). Prepared following general procedure using 4-methyl-*N*-propylbenzenesulfonimidoyl fluoride **1a** (0.6 mmol, 129.2 mg), potassium trifluoro(mesityl)borate **2h** (0.4 mmol, 90.4 mg), TMSOTf (0.08 mmol, 14.4 μL) and CH_3CN (2.0 mL), the reaction was stirred at 40 °C for 30 min giving **3h** in 78% yield (94.0 mg) as a colorless oil by column chromatography (PE/EA = 5:1, R_f = 0.63). **^{1}H NMR** (400 MHz, $CDCl_3$) δ 7.79 (d, *J* = 8.0 Hz, 2H), 7.20 (d, *J* = 8.0 Hz, 2H), 6.90 (s, 2H), 3.14 – 3.00 (m, 1H), 2.88 – 2.77 (m, 1H), 2.50 (s, 6H), 2.36 (s, 3H), 2.27 (s, 3H), 1.71 – 1.56 (m, 2H), 0.95 (t, *J* = 7.4 Hz, 3H). **^{13}C NMR** (101 MHz, $CDCl_3$) δ 142.2, 141.6, 140.4, 140.3, 133.6, 132.2, 129.0,

127.2, 45.6, 25.9, 22.9, 21.3, 20.7, 11.9. **IR** (film) v 2957, 2870, 1599, 1450, 1224, 1141, 1093, 1016, 717, 705, 615 cm^{-1}. **HRMS** (ESI) m/z: $[M+H]^+$ Calcd for $C_{19}H_{26}NOS$ 316.1730, found 316.1723.

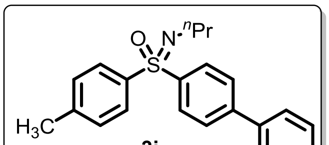

3i

[1,1'-biphenyl]-4-yl(propylimino)(*p*-tolyl)-λ^6-sulfanone (**3i**). Prepared following general procedure using 4-methyl-*N*-propylbenzenesulfonimidoyl fluoride **1a** (0.6 mmol, 129.2 mg), potassium [1,1'-biphenyl]-4-yltrifluoroborate **2i** (0.4 mmol, 104.0 mg), TMSOTf (0.08 mmol, 14.4 µL) and CH_3CN (2.0 mL), the reaction was stirred at 40 °C for 30 min giving **3i** in 98% yield (137.8 mg) as a colorless oil by column chromatography (PE/EA = 5:1, R_f = 0.31). **^{1}H NMR** (400 MHz, $CDCl_3$) δ 8.03 (d, *J* = 8.5 Hz, 2H), 7.90 (d, *J* = 8.2 Hz, 2H), 7.65 (d, *J* = 8.5 Hz, 2H), 7.57 – 7.52 (m, 2H), 7.47 – 7.41 (m, 2H), 7.40 – 7.35 (m, 1H), 7.28 (d, *J* = 8.1 Hz, 2H), 3.05 (t, *J* = 7.1 Hz, 2H), 2.37 (s, 3H), 1.78 – 1.64 (m, 2H), 0.99 (t, *J* = 7.3 Hz, 3H). **^{13}C NMR** (101 MHz, $CDCl_3$) δ 144.9, 142.9, 139.8, 139.3, 137.9, 129.7, 128.8, 128.5, 128.1, 127.6, 127.1, 45.7, 26.1, 21.3, 11.8. **IR** (film) v 3061, 2957, 2870, 1593, 1479, 1396, 1224, 1141, 1095, 841, 675 cm^{-1}. **HRMS** (ESI) m/z: $[M+H]^+$ Calcd for $C_{22}H_{24}NOS$ 350.1573, found 350.1565.

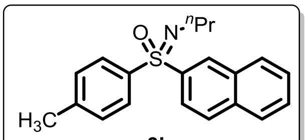

3j

Naphthalen-2-yl(propylimino)(*p*-tolyl)-λ^6-sulfanone (**3j**). Prepared following general procedure using 4-methyl-*N*-propylbenzenesulfonimidoyl fluoride **1a** (0.6 mmol, 129.2 mg), potassium trifluoro(naphthalen-2-yl)borate **2j** (0.4 mmol, 93.6 mg), TMSOTf (0.12 mmol, 21.6 µL) and CH_3CN (2.0 mL), the reaction was stirred at 40 °C for 30 min giving **3j** in 95% yield (123.1 mg) as a colorless oil by column chromatography (PE/EA = 5:1, R_f = 0.35). **^{1}H NMR** (400 MHz, $CDCl_3$) δ 8.58 (s, 1H), 7.97 – 7.81 (m, 6H), 7.60 – 7.51 (m, 2H), 7.25 (d, *J* = 8.2 Hz, 2H), 3.11 – 3.00 (m, 2H), 2.34 (s, 3H), 1.77 – 1.64 (m, 2H), 0.98 (t, *J* = 7.3 Hz, 3H). **^{13}C NMR** (101 MHz,

$CDCl_3$) δ 143.0, 138.1, 137.8, 134.5, 132.4, 129.7(3), 129.7(1), 129.1, 128.5, 128.4, 127.7, 127.1, 123.6, 45.8, 26.1, 21.3, 11.8. **IR** (film) ν 3055, 2957, 2870, 1593, 1491, 1253, 1124, 1082, 812, 673 cm^{-1}. **HRMS** (ESI) m/z: $[M+H]^+$ Calcd for $C_{20}H_{22}NOS$ 324.1417, found 324.1408.

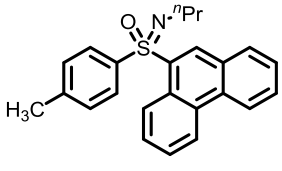

3k

Phenanthren-9-yl(propylimino)(*p*-tolyl)-λ^6-sulfanone (**3k**). Prepared following general procedure using 4-methyl-*N*-propylbenzenesulfonimidoyl fluoride **1a** (0.6 mmol, 129.2 mg), potassium trifluoro(phenanthrene-9-yl)borate **2k** (0.4 mmol, 113.6 mg), TMSOTf (0.2 mmol, 36.0 μL) and CH_3CN (2.0 mL), the reaction was stirred at rt for 30 min giving **3k** in 63% yield (97.4 mg) as a colorless oil by column chromatography (PE/EA = 5:1, R_f = 0.31). ^{1}**H NMR** (400 MHz, $CDCl_3$) δ 8.97 (s, 1H), 8.88 (dd, *J* = 8.3, 1.3 Hz, 1H), 8.72 – 8.60 (m, 2H), 8.09 (dd, *J* = 7.9, 1.4 Hz, 1H), 7.99 (d, *J* = 8.4 Hz, 2H), 7.81 – 7.74 (m, 1H), 7.72 – 7.66 (m, 1H), 7.65 – 7.60 (m, 1H), 7.59 – 7.53 (m, 1H), 7.21 (d, *J* = 8.2 Hz, 2H), 3.22 – 3.11 (m, 1H), 3.04 – 2.94 (m, 1H), 2.32 (s, 3H), 1.78 – 1.60 (m, 2H), 0.95 (t, *J* = 7.4 Hz, 3H). ^{13}**C NMR** (101 MHz, $CDCl_3$) δ 142.9, 138.0, 134.3, 133.8, 132.4, 131.1, 130.4, 129.8, 129.4(6), 129.4(1), 128.0, 127.3, 127.2, 127.0, 126.8, 125.6, 123.0, 122.6, 45.9, 26.0, 21.3, 11.8. **IR** (film) ν 2958, 2868, 1491, 1450, 1226, 1130, 1089, 860, 756, 675 cm^{-1}. **HRMS** (ESI) m/z: $[M+H]^+$ Calcd for $C_{24}H_{24}NOS$ 374.1573, found 374.1565.

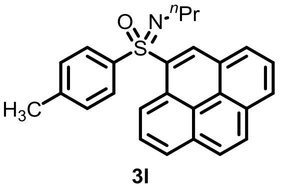

3l

Pyren-9-yl(propylimino)(*p*-tolyl)-λ^6-sulfanone (**3l**). Prepared following general procedure using 4-methyl-*N*-propylbenzenesulfonimidoyl fluoride **1a** (0.6 mmol, 129.2 mg), potassium trifluoro(pyren-2-yl)borate **2l** (0.4 mmol, 123.3 mg), TMSOTf (0.08 mmol, 14.4 μL) and CH_3CN (2.0 mL), the reaction was stirred at 40 °C for 30 min giving **3l** in 68% yield (106.9 mg) as a yellow oil by column chromatography (PE/EA = 5:1, R_f = 0.21). ^{1}**H NMR** (400 MHz, $CDCl_3$) δ 9.21 (d,

J = 8.0 Hz, 1H), 9.02 (d, *J* = 8.2 Hz, 1H), 8.27 (d, *J* = 8.3 Hz, 1H), 8.21 (d, *J* = 7.6 Hz, 2H), 8.18 – 8.12 (m, 2H), 8.09 – 7.98 (m, 4H), 7.21 (d, *J* = 8.1 Hz, 2H), 3.22 – 3.14 (m, 1H), 3.08 – 3.00 (m, 1H), 2.30 (s, 3H), 1.80 – 1.69 (m, 2H), 0.98 (t, *J* = 7.3 Hz, 3H). **^{13}C NMR** (101 MHz, $CDCl_3$) δ 142.7, 138.8, 134.6, 131.9, 130.8, 129.9(9), 129.9(2), 129.4(6), 129.4(0), 128.9, 128.0, 127.0, 126.5, 126.4, 125.1, 124.2, 123.9, 123.3, 45.9, 26.1, 21.3, 11.9. **IR** (film) v 2957, 2870, 1712, 1591, 1253, 1190, 1163, 850, 756, 626 cm^{-1}. **HRMS** (ESI) m/z: $[M+H]^+$ Calcd for $C_{26}H_{24}NOS$ 398.1575, found 398.1567.

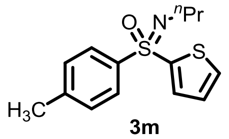
3m

(Propylimino)(thiophen-2-yl)(*p*-tolyl)-λ^6-sulfanone (**3m**). Prepared following general procedure using 4-methyl-*N*-propylbenzenesulfonimidoyl fluoride **1a** (0.6 mmol, 129.2 mg), potassium trifluoro(thiophen-2-yl)borate **2m** (0.4 mmol, 76.0 mg), TMSOTf (0.08 mmol, 14.4 µL) and CH_3CN (2.0 mL), the reaction was stirred at 40 °C for 30 min giving **3m** in 79% yield (87.8 mg) as a white solid by column chromatography (PE/EA = 5:1, R_f = 0.58). **^{1}H NMR** (400 MHz, $CDCl_3$) δ 7.91 (d, *J* = 8.1 Hz, 2H), 7.54 (d, *J* = 5.0 Hz, 1H), 7.50 (d, *J* = 3.5 Hz, 1H), 7.25 (d, *J* = 8.1 Hz, 2H), 7.01 (dd, *J* = 4.8, 3.9 Hz, 1H), 3.14 – 2.99 (m, 2H), 2.36 (s, 3H), 1.73 – 1.59 (m, 2H), 0.95 (t, *J* = 7.3 Hz, 3H). **^{13}C NMR** (101 MHz, $CDCl_3$) δ 143.1(2), 143.1(0), 138.4, 133.2, 132.8, 129.6, 127.9, 127.7, 45.8, 25.9, 21.3, 11.7. **IR** (film) v 2957, 2928, 2872, 1597, 1400, 1255, 1222, 1126, 1001, 746, 678 cm^{-1}. **HRMS** (ESI) m/z: $[M+H]^+$ Calcd for $C_{14}H_{18}NOS_2$ 280.0824, found 280.0816.

Benzofuran-2-yl(propylimino)(*p*-tolyl)-λ^6-sulfanone (**3n**). Prepared following general procedure using 4-methyl-*N*-propylbenzenesulfonimidoyl fluoride **1a** (0.6 mmol, 129.2 mg), potassium benzofuran-2-yltrifluoroborate **2n** (0.4 mmol, 89.6 mg), TMSOTf (0.08 mmol, 14.4 µL) and CH_3CN (2.0 mL), the reaction was stirred at

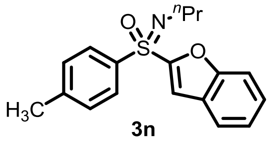
3n

40 °C for 30 min giving **3n** in 99% yield (125.0 mg) as a yellow oil by column chromatography (PE/EA = 5:1, R_f = 0.41). **^{1}H NMR** (400 MHz, $CDCl_3$) δ 8.02 (d, *J* = 8.4 Hz, 2H), 7.61 (d, *J* = 7.8 Hz, 1H), 7.48 – 7.44 (m, 2H), 7.38 – 7.32 (m, 1H), 7.29 – 7.22 (m, 3H), 3.21 – 3.13 (m, 1H), 3.06 – 2.98 (m, 1H), 2.35 (s, 3H), 1.72 – 1.60 (m, 2H), 0.95 (t, *J* = 7.4 Hz, 3H). **^{13}C NMR** (101 MHz, $CDCl_3$) δ 156.2, 151.2, 143.9, 135.9, 129.6, 128.6, 127.0, 126.5, 123.7, 122.5, 114.1, 112.2, 46.0, 25.8, 21.4, 11.7. **IR** (film) v 2958, 2872, 1595, 1533, 1442, 1284, 1251, 1143, 927, 812 cm^{-1}. **HRMS** (ESI) m/z: $[M+H]^+$ Calcd for $C_{18}H_{20}NO_2S$ 314.1209, found 314.1201.

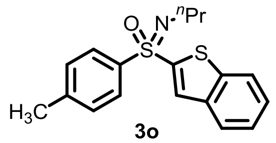

3o

Benzo[*b*]thiophen-2-yl(propylimino)(*p*-tolyl)-λ^6-sulfanone (**3o**). Prepared following general procedure using 4-methyl-*N*-propylbenzenesulfonimidoyl fluoride **1a** (0.6 mmol, 129.2 mg), potassium benzo[*b*]thiophen-2-yltrifluoroborate **2o** (0.4 mmol, 96.0 mg), TMSOTf (0.08 mmol, 14.4 μL) and CH_3CN (2.0 mL), the reaction was stirred at 40 °C for 30 min giving **3o** in 93% yield (122.2 mg) as a colorless oil by column chromatography (PE/EA = 5:1, R_f = 0.43). **^{1}H NMR** (400 MHz, $CDCl_3$) δ 7.99 (d, *J* = 8.4 Hz, 2H), 7.84 – 7.76 (m, 3H), 7.43 – 7.35 (m, 2H), 7.28 (d, *J* = 8.1 Hz, 2H), 3.22 – 3.07 (m, 2H), 2.38 (s, 3H), 1.78 – 1.65 (m, 2H), 1.00 (t, *J* = 7.4 Hz, 3H). **^{13}C NMR** (101 MHz, $CDCl_3$) δ 143.8, 143.5, 143.0, 138.3, 137.9, 130.0, 129.7, 128.3, 126.6, 125.4, 125.0, 122.5, 46.0, 26.0, 21.4, 11.8. **IR** (film) v 3061, 2957, 2870, 1593, 1498, 1255, 1141, 910, 852, 746 cm^{-1}. **HRMS** (ESI) m/z: $[M+H]^+$ Calcd for $C_{18}H_{20}NOS_2$ 330.0981, found 330.0972.

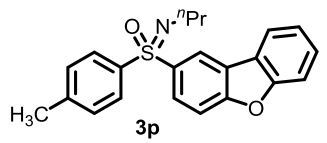

3p

Dibenzo[*b,d*]furan-2-yl(propylimino)(*p*-tolyl)-λ^6-sulfanone (**3p**). Prepared following general procedure using 4-methyl-*N*-propylbenzenesulfonimidoyl fluoride **1a** (0.6 mmol, 129.2 mg), potassium dibenzo[*b,d*]furan-2-yltrifluoroborate **2p** (0.4 mmol, 109.6 mg), TMSOTf (0.2

mmol, 36.0 µL) and CH_3CN (2.0 mL), the reaction was stirred at rt for 30 min giving **3p** in 60% yield (87.6 mg) as a yellow oil by column chromatography (PE/EA = 5:1, R_f = 0.29). **^{1}H NMR** (400 MHz, $CDCl_3$) δ 8.61 (d, *J* = 1.9 Hz, 1H), 8.04 (dd, *J* = 8.7, 2.0 Hz, 1H), 7.96 (dd, *J* = 7.3, 1.0 Hz, 1H), 7.89 (d, *J* = 8.4 Hz, 2H), 7.58 (d, *J* = 8.7 Hz, 1H), 7.54 (d, *J* = 8.3 Hz, 1H), 7.50 – 7.44 (m, 1H), 7.38 – 7.33 (m, 1H), 7.27 – 7.23 (m, 2H), 3.05 (t, *J* = 7.1 Hz, 2H), 2.34 (s, 3H), 1.77 – 1.65 (m, 2H), 0.98 (t, *J* = 7.4 Hz, 3H). **^{13}C NMR** (101 MHz, $CDCl_3$) δ 157.9, 156.8, 142.9, 138.2, 135.7, 129.7, 128.4, 128.1, 127.5, 124.9, 123.4, 123.2, 121.9, 121.1, 112.1, 111.8, 45.8, 26.2, 21.3, 11.8. **IR** (film) v 2957, 2870, 1593, 1446, 1442, 1248, 1226, 1082, 1020, 841, 748 cm^{-1}. **HRMS** (ESI) m/z: $[M+H]^+$ Calcd for $C_{22}H_{22}NO_2S$ 364.1366, found 364.1366.

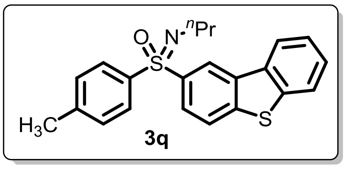

Dibenzo[*b,d*]thiophen-2-yl(propylimino)(*p*-tolyl)-λ^6-sulfanone (**3q**). Prepared following general procedure using 4-methyl-*N*-propylbenzenesulfonimidoyl fluoride **1a** (0.6 mmol, 129.2 mg), potassium dibenzo[*b,d*]thiophen-2-yltrifluoroborate **2q** (0.4 mmol, 116.1 mg), TMSOTf (0.2 mmol, 36.0 µL) and CH_3CN (2.0 mL), the reaction was stirred at rt for 30 min giving **3q** in 54% yield (81.6 mg) as a yellow oil by column chromatography (PE/EA = 5:1, R_f = 0.33). **^{1}H NMR** (400 MHz, $CDCl_3$) δ 8.77 (d, *J* = 1.7 Hz, 1H), 8.23 – 8.18 (m, 1H), 7.96 (dd, *J* = 8.5, 1.8 Hz, 1H), 7.92 (d, *J* = 8.3 Hz, 2H), 7.87 (d, *J* = 8.5 Hz, 1H), 7.84 – 7.76 (m, 1H), 7.49 – 7.44 (m, 2H), 7.25 (d, *J* = 6.4 Hz, 2H), 3.09 – 3.04 (m, 2H), 2.34 (s, 3H), 1.77 – 1.66 (m, 2H), 0.99 (t, *J* = 7.3 Hz, 3H). **^{13}C NMR** (101 MHz, $CDCl_3$) δ 143.5, 142.9, 139.7, 138.1, 137.4, 135.8, 134.6, 129.7, 128.4, 127.5, 125.8, 124.8, 123.2, 122.7, 122.0, 45.8, 26.1, 21.3, 11.8. **IR** (film) v 3059, 2957, 2870, 1595, 1456, 1226, 1141, 1091, 812, 732, 677 cm^{-1}. **HRMS** (ESI) m/z: $[M+H]^+$ Calcd for $C_{22}H_{22}NOS_2$ 380.1137, found 380.1128.

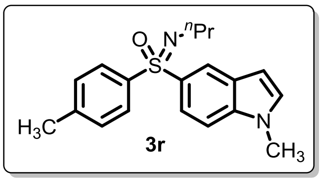

(1-methyl-1*H*-indol-5-yl)(propylimino)(*p*-tolyl)-λ^6-sulfanone (**3r**). Prepared following general procedure using 4-methyl-*N*-propylbenzenesulfonimidoyl fluoride **1a** (0.6 mmol, 129.2 mg), potassium trifluoro(1-methyl-1*H*-indol-5-yl)borate **2r** (0.4 mmol, 94.8 mg), TMSOTf (0.2 mmol, 36.0 μL) and CH_3CN (2.0 mL), the reaction was stirred at rt for 30 min giving **3r** in 66% yield (87.5 mg) as a yellow solid by column chromatography (PE/EA = 5:1, R_f = 0.08). **^{1}H NMR** (400 MHz, $CDCl_3$) δ 8.31 (d, *J* = 1.8 Hz, 1H), 7.87 (d, *J* = 8.3 Hz, 2H), 7.77 (dd, *J* = 8.7, 1.8 Hz, 1H), 7.33 (d, *J* = 8.7 Hz, 1H), 7.21 (d, *J* = 8.1 Hz, 2H), 7.12 (d, *J* = 3.2 Hz, 1H), 6.57 (d, *J* = 3.1 Hz, 1H), 3.77 (s, 3H), 3.03 (t, *J* = 7.0 Hz, 2H), 2.33 (s, 3H), 1.75 – 1.63 (m, 2H), 0.97 (t, *J* = 7.4 Hz, 3H). **^{13}C NMR** (101 MHz, $CDCl_3$) δ 142.2, 139.1, 138.1, 131.3, 130.7, 129.5, 128.2, 128.1, 122.8, 121.3, 109.5, 102.5, 45.9, 33.0, 26.2, 21.3, 11.9. **IR** (film) v 2957, 2924, 2870, 1604, 1512, 1220, 1136. 1080, 1055, 814, 678 cm^{-1}. **HRMS** (ESI) m/z: $[M+H]^+$ Calcd for $C_{19}H_{23}N_2OS$ 327.1526, found 327.1517.

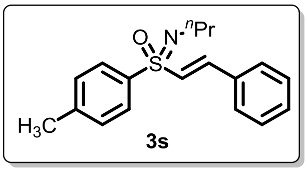

(*E*)-(propylimino)(styryl)(*p*-tolyl)-λ^6-sulfanone (**3s**). Prepared following general procedure using 4-methyl-*N*-propylbenzenesulfonimidoyl fluoride **1a** (0.6 mmol, 129.2 mg), potassium (*E*)-trifluoro(styryl)borate **2s** (0.4 mmol, 84.0 mg), TMSOTf (0.12 mmol, 21.6 μL) and CH_3CN (2.0 mL), the reaction was stirred at 40 °C for 30 min giving **3s** in 72% yield (86.6 mg) as a colorless oil by column chromatography (PE/EA = 5:1, R_f = 0.35). **^{1}H NMR** (400 MHz, $CDCl_3$) δ 7.83 (d, *J* = 8.3 Hz, 2H), 7.52 (d, *J* = 15.4 Hz, 1H), 7.47 – 7.43 (m, 2H), 7.37 – 7.29 (m, 5H), 6.88 (d, *J* = 15.4 Hz, 1H), 3.08 – 2.99 (m, 1H), 2.98 – 2.90 (m, 1H), 2.41 (s, 3H), 1.70 – 1.58 (m, 2H), 0.94 (t, *J* = 7.4 Hz, 3H). **^{13}C NMR** (101 MHz, $CDCl_3$) δ 143.3, 141.8, 137.0, 132.9, 130.5, 129.9, 128.8, 128.6, 128.3, 128.0, 45.6, 26.1, 21.4, 11.8. **IR** (film) v 2957, 2872, 1710, 1448, 1361, 1253, 1219, 1080, 1039, 785 cm^{-1}. **HRMS** (ESI) m/z: $[M+H]^+$ Calcd for $C_{18}H_{22}NOS$ 300.1417, found 300.1411.

Allyl(propylimino)(*p*-tolyl)-λ⁶-sulfanone (**3t**). Prepared following general procedure using 4-methyl-*N*-propylbenzenesulfonimidoyl fluoride **1a** (0.6 mmol, 129.2 mg), potassium allyltrifluoroborate **2t** (0.4 mmol, 94.8 mg), TMSOTf (0.12 mmol, 21.6 μL) and CH_3CN (2.0 mL), the reaction was stirred at 40 °C for 30 min giving **3t** in 54% yield (51.5 mg) as a yellow oil by column chromatography (PE/EA = 5:1, R_f = 0.29). **^{1}H NMR** (400 MHz, $CDCl_3$) δ 7.67 (d, *J* = 8.2 Hz, 2H), 7.29 (d, *J* = 8.0 Hz, 2H), 5.81 – 5.68 (m, 1H), 5.21 (dd, *J* = 10.2, 1.2 Hz, 1H), 5.01 (dd, *J* = 17.1, 1.3 Hz, 1H), 3.81 (d, *J* = 7.3 Hz, 2H), 2.98 – 2.89 (m, 1H), 2.87 – 2.75 (m, 1H), 2.40 (s, 3H), 1.60 – 1.49 (m, 2H), 0.87 (t, *J* = 7.4 Hz, 3H). **^{13}C NMR** (101 MHz, $CDCl_3$) δ 143.4, 134.4, 129.6, 129.5, 125.6, 123.5, 60.9, 45.7, 26.0, 21.4, 11.6. **IR** (film) ν 2957, 2872, 1595, 1421, 1253, 1222, 1136, 1082, 885, 773, 640 cm^{-1}. **HRMS** (ESI) m/z: $[M+H]^+$ Calcd for $C_{13}H_{20}NOS$ 238.1260, found 238.1254.

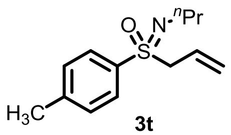

3t

Benzofuran-2-yl(2-bromophenyl)(propylimino)-λ⁶-sulfanone (**3u**). Prepared following general procedure using 2-bromo-*N*-propylbenzenesulfonimidoyl fluoride **1b** (0.6 mmol, 168.1 mg), potassium benzofuran-2-yltrifluoroborate **2n** (0.4 mmol, 89.6 mg), TMSOTf (0.08 mmol, 14.4 μL) and CH_3CN (2.0 mL), the reaction was stirred at 40 °C for 30 min giving **3u** in 72% yield (109.5 mg) as a yellow oil by column chromatography (PE/EA = 5:1, R_f = 0.60). **^{1}H NMR** (400 MHz, $CDCl_3$) δ 8.53 (dd, *J* = 8.0, 1.7 Hz, 1H), 7.69 – 7.62 (m, 3H), 7.52 – 7.44 (m, 2H), 7.41 – 7.32 (m, 2H), 7.31 – 7.24 (m, 1H), 3.22 – 3.13 (m, 1H), 3.11 – 3.03 (m, 1H), 1.74 – 1.62 (m, 2H), 0.96 (t, *J* = 7.4 Hz, 3H). **^{13}C NMR** (101 MHz, $CDCl_3$) δ 156.1, 149.4, 137.4, 135.8, 133.9, 133.0, 127.8, 127.3, 126.3, 123.9, 122.7, 121.5, 115.9, 112.3, 45.8, 25.7, 11.7. **IR** (film) ν 2958, 2872, 2848, 1533, 1442, 1249, 1149, 1070, 790, 723, 601 cm^{-1}. **HRMS** (ESI) m/z: $[M+H]^+$ Calcd for $C_{17}H_{17}BrNO_2S$ 378.0314; 380.0126, found 378.0158; 380.0149.

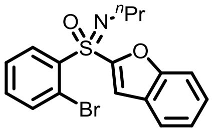

3u

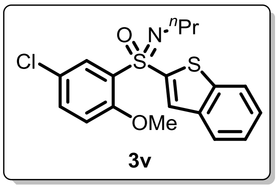

Benzo[*b*]thiophen-2-yl(5-chloro-2-methoxyphenyl)(*p*-ropylimino)-λ⁶-sulfanone (**3v**). Prepared following general procedure using 5-chloro-2-methoxy-*N*-propyl benzenesulfonimidoyl fluoride **1c** (0.6 mmol, 159.0 mg), potassium benzo[*b*]thiophen-2-yltrifluoroborate **2o** (0.4 mmol, 96.0 mg), TMSOTf (0.12 mmol, 21.6 µL) and CH_3CN (2.0 mL), the reaction was stirred at 40 °C for 30 min giving **3v** in 60% yield (90.6 mg) as a white solid by column chromatography (PE/EA = 5:1, R_f = 0.31). **^{1}H NMR** (400 MHz, $CDCl_3$) δ 8.24 (d, *J* = 2.7 Hz, 1H), 7.91 (s, 1H), 7.86 – 7.79 (m, 2H), 7.47 – 7.36 (m, 3H), 6.86 (d, *J* = 8.8 Hz, 1H), 3.83 (s, 3H), 3.07 (t, *J* = 7.0 Hz, 2H), 1.72 – 1.59 (m, 2H), 0.96 (t, *J* = 7.4 Hz, 3H). **^{13}C NMR** (101 MHz, $CDCl_3$) δ 155.8, 143.1, 142.9, 138.0, 134.3, 131.1, 131.0, 129.4, 126.7, 125.7, 125.4, 124.9, 122.4, 113.7, 56.0, 46.1, 25.8, 11.7. **IR** (film) v 2953, 2872, 1589, 1475, 1275, 1151, 756, 725, 646, 601 cm^{-1}. **HRMS** (ESI) m/z: $[M+H]^+$ Calcd for : $C_{18}H_{19}ClNO_2S_2$ 380.0540, found 380.0532.

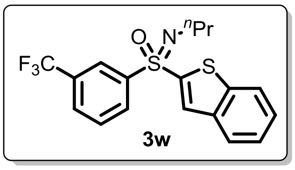

Benzo[*b*]thiophen-2-yl(propylimino)(3-(trifluoromethyl)phenyl)-λ⁶-sulfanone (**3w**). Prepared following general procedure using *N*-propyl-3-(trifluoromethyl) benzenesulfonimidoyl fluoride **1d** (0.6 mmol, 161.6 mg), potassium benzo[*b*]thiophen-2-yltrifluoroborate **2o** (0.4 mmol, 96.0 mg), TMSOTf (0.08 mmol, 14.4 µL) and CH_3CN (2.0 mL), the reaction was stirred at 40 °C for 30 min giving **3w** in 99% yield (152.8 mg) as a yellow oil by column chromatography (PE/EA = 5:1, R_f = 0.63). **^{1}H NMR** (400 MHz, $CDCl_3$) δ 8.40 (s, 1H), 8.30 (d, *J* = 8.0 Hz, 1H), 7.89 (s, 1H), 7.87 – 7.76 (m, 3H), 7.66 – 7.61 (m, 1H), 7.48 – 7.37 (m, 2H), 3.22 – 3.12 (m, 2H), 1.79 – 1.67 (m, 2H), 1.01 (t, *J* = 7.4 Hz, 3H). **^{13}C NMR** (101 MHz, $CDCl_3$) δ 143.2, 142.4(8), 142.4(1), 138.2, 131.7 (q, *J* = 33.5 Hz), 131.5, 131.0, 129.8, 129.2 (q, *J* = 3.5 Hz), 127.1, 125.6, 125.3(6) (q, *J* = 4.04 Hz), 125.3(0), 123.1 (q, *J* = 273.0 Hz), 122.6, 45.9, 25.9, 11.7. **^{19}F NMR** (376 MHz, $CDCl_3$) δ -62.6. **IR** (film) v 2960, 2874, 1498, 1423,

1323, 1124, 1068, 746 cm^{-1}. **HRMS** (ESI) m/z: $[M+H]^+$ Calcd for : $C_{18}H_{17}F_3NOS_2$ 384.0698, found 384.0689.

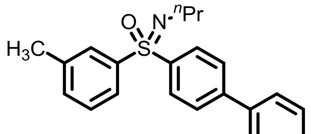

[1,1'-biphenyl]-4-yl(propylimino)(*m*-tolyl)-λ⁶-sulfanone (3x). Prepared following general procedure using 3-methyl-*N*-propylbenzenesulfonimidoyl fluoride **1e** (0.6 mmol, 129.0 mg), potassium [1,1'-biphenyl]-4-yltrifluoroborate **2i** (0.4 mmol, 104.0 mg), TMSOTf (0.08 mmol, 14.4 µL) and CH_3CN (2.0 mL), the reaction was stirred at 40 °C for 30 min giving **3x** in 98% yield (137.0 mg) as a colorless oil by column chromatography (PE/EA = 5:1, R_f = 0.40). **^{1}H NMR** (400 MHz, $CDCl_3$) δ 8.06 – 8.01 (m, 2H), 7.86 – 7.80 (m, 2H), 7.68 – 7.63 (m, 2H), 7.57 – 7.52 (m, 2H), 7.46 – 7.40 (m, 2H), 7.39 – 7.33 (m, 2H), 7.30 (d, *J* = 7.9 Hz, 1H), 3.07 (t, *J* = 7.1 Hz, 2H), 2.39 (s, 3H), 1.77 – 1.66 (m, 2H), 1.00 (t, *J* = 7.4 Hz, 3H). **^{13}C NMR** (101 MHz, $CDCl_3$) δ 145.0, 140.7, 139.5, 139.3, 139.1, 133.0, 128.9, 128.8, 128.7, 128.1, 127.6, 127.1, 125.5, 45.6, 26.0, 21.2, 11.7. **IR** (film) ν 2926, 2870, 1593, 1477, 1226, 1124, 1006, 761, 694 cm^{-1}. **HRMS** (ESI) m/z: $[M+H]^+$ Calcd for : $C_{22}H_{24}NOS$ 350.1573, found 350.1566.

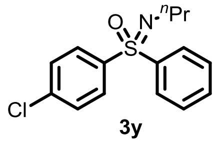

(4-chlorophenyl)(phenyl)(propylimino)-λ⁶-sulfanone (3y). Prepared following general procedure using 4-chloro-*N*-propylbenzenesulfonimidoyl fluoride **1f** (0.6 mmol, 141.0 mg), potassium trifluoro(phenyl)borate **2a** (0.4 mmol, 73.6 mg), TMSOTf (0.12 mmol, 21.6 µL) and CH_3CN (2.0 mL), the reaction was stirred at 40 °C for 30 min giving **3y** in 93% yield (109.0 mg) as a yellow oil by column chromatography (PE/EA = 5:1, R_f = 0.57). **^{1}H NMR** (400 MHz, $CDCl_3$) δ 7.97 – 7.92 (m, 2H), 7.91 – 7.86 (m, 2H), 7.52 – 7.43 (m, 3H), 7.43 – 7.39 (m, 2H), 3.01 – 2.97 (m, 2H), 1.71 – 1.59 (m, 2H), 0.95 (t, *J* = 7.4 Hz, 3H). **^{13}C NMR** (101 MHz, $CDCl_3$) δ 140.6, 139.6, 138.7, 132.3, 129.9, 129.2, 129.1, 128.4, 45.6, 26.0, 11.7. **IR** (film) ν 2957, 2841, 1577, 1473, 1253, 1143, 1078, 763, 744 cm^{-1}. **HRMS** (ESI) m/z: $[M+H]^+$ Calcd for : $C_{15}H_{17}ClNOS$

294.0714, found 294.0707.

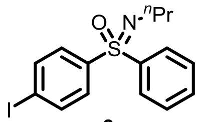
3z

(4-iodophenyl)(phenyl)(propylimino)-λ^6-sulfanone (**3z**). Prepared following general procedure using 4-iodo-*N*-propylbenzenesulfonimidoyl fluoride **1g** (0.6 mmol, 196.0 mg), potassium trifluoro(phenyl)borate **2a** (0.4 mmol, 73.6 mg), TMSOTf (0.08 mmol, 14.4 µL) and CH_3CN (2.0 mL), the reaction was stirred at 40 °C for 30 min giving **3z** in 77% yield (118.8 mg) as a white solid by column chromatography (PE/EA = 5:1, R_f = 0.60). **^{1}H NMR** (400 MHz, $CDCl_3$) δ 7.96 – 7.91 (m, 2H), 7.81 – 7.75 (m, 2H), 7.68 – 7.63 (m, 2H), 7.51 – 7.41 (m, 3H), 3.01 – 2.96 (m, 2H), 1.70 – 1.59 (m, 2H), 0.94 (t, *J* = 7.4 Hz, 3H). **^{13}C NMR** (101 MHz, $CDCl_3$) δ 140.8, 140.5, 138.2, 132.3, 129.9, 129.0, 128.3, 99.8, 45.5, 26.0, 11.7. **IR** (film) ν 3063, 2957, 2870, 1564, 1381, 1228, 1143, 1004, 815, 732 cm^{-1}. **HRMS** (ESI) m/z: $[M+H]^+$ Calcd for : $C_{15}H_{17}INOS$ 386.0070, found 386.0061.

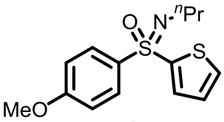
3aa

(4-methoxyphenyl)(propylimino)(thiophen-2-yl)-λ^6-sulfanone (**3aa**). Prepared following general procedure using 4-methoxy-*N*-propylbenzenesulfonimidoyl fluoride **1h** (0.6 mmol, 133.9 mg), potassium trifluoro(thiophen-2-yl)borate **2m** (0.4 mmol, 76.0 mg), TMSOTf (0.08 mmol, 14.4 µL) and CH_3CN (2.0 mL), the reaction was stirred at rt for 30 min giving **3aa** in 95% yield (112.8 mg) as a colorless oil by column chromatography (PE/EA = 5:1, R_f = 0.23). **^{1}H NMR** (400 MHz, $CDCl_3$) δ 7.98 – 7.92 (m, 2H), 7.52 (dd, *J* = 5.0, 1.3 Hz, 1H), 7.48 (dd, *J* = 3.8, 1.4 Hz, 1H), 7.00 (dd, *J* = 5.0, 3.8 Hz, 1H), 6.95 – 6.88 (m, 2H), 3.79 (s, 3H), 3.18 – 2.92 (m, 2H), 1.75 – 1.56 (m, 2H), 0.94 (t, *J* = 7.4 Hz, 3H). **^{13}C NMR** (101 MHz, $CDCl_3$) δ 162.7, 143.5, 132.9(5), 132.9(1), 132.6, 130.1, 127.6, 114.1, 55.4, 45.8, 25.9, 11.7. **IR** (film) ν 3101, 2957, 2872, 1710, 1591, 1492, 1402, 1307, 829, 746, 680 cm^{-1}. **HRMS** (ESI) m/z: $[M+H]^+$ Calcd for : $C_{14}H_{18}NO_2S_2$ 296.0773, found 296.0766.

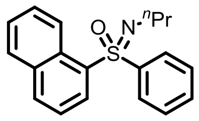
3ab

Naphthalen-1-yl(phenyl)(propylimino)-λ^6-sulfanone (**3ab**). Prepared following general procedure using *N*-propylnaphthalene-1-sulfonimidoyl fluoride **1i** (0.6 mmol, 151.0 mg), potassium trifluoro(phenyl)borate **2a** (0.4 mmol, 73.6 mg), TMSOTf (0.12 mmol, 21.6 µL) and CH_3CN (2.0 mL), the reaction was stirred at 40 °C for 30 min giving **3ab** in 60% yield (74.6 mg) as a white solid by column chromatography (PE/EA = 5:1, R_f = 0.40). **^{1}H NMR** (400 MHz, $CDCl_3$) δ 8.87 – 8.82 (m, 1H), 8.56 (dd, *J* = 7.4, 1.3 Hz, 1H), 8.08 – 8.01 (m, 3H), 7.87 (dd, *J* = 8.0, 1.6 Hz, 1H), 7.60 (dd, *J* = 8.2, 7.4 Hz, 1H), 7.55 – 7.47 (m, 2H), 7.46 – 7.39 (m, 3H), 3.14 – 3.06 (m, 1H), 3.00 – 2.93 (m, 1H), 1.75 – 1.61 (m, 2H), 0.95 (t, *J* = 7.4 Hz, 3H). **^{13}C NMR** (101 MHz, $CDCl_3$) δ 141.3, 135.4, 134.3, 134.2, 132.1, 131.5, 129.2, 128.8, 128.0, 127.7, 126.4, 124.7, 124.6, 45.8, 26.1, 11.8. **IR** (film) v 3059, 2957, 2870, 1712, 1504, 1226, 1122, 1024, 769, 686, 592 cm^{-1}. **HRMS** (ESI) m/z: $[M+H]^+$ Calcd for : $C_{19}H_{20}NOS$ 310.1260, found 310.1253.

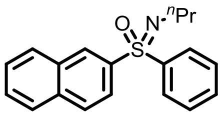
3ac

Naphthalen-2-yl(phenyl)(propylimino)-λ^6-sulfanone (**3ac**). Prepared following general procedure using *N*-propylnaphthalene-2-sulfonimidoyl fluoride **1j** (0.6 mmol, 151.0 mg), potassium trifluoro(phenyl)borate **2a** (0.4 mmol, 73.6 mg), TMSOTf (0.12 mmol, 21.6 µL) and CH_3CN (2.0 mL), the reaction was stirred at 40 °C for 30 min giving **3ac** in 97% yield (124.0 mg) as a colorless oil by column chromatography (PE/EA = 5:1, R_f = 0.45). **^{1}H NMR** (400 MHz, $CDCl_3$) δ 8.60 (s, 1H), 8.08 – 8.03 (m, 2H), 7.98 – 7.81 (m, 4H), 7.57 (m, 2H), 7.51 – 7.42 (m, 3H), 3.13 – 3.01 (m, 2H), 1.77 – 1.66 (m, 2H), 0.99 (t, *J* = 7.3 Hz, 3H). **^{13}C NMR** (101 MHz, $CDCl_3$) δ 140.8, 137.7, 134.5, 132.4, 132.2, 129.9, 129.2, 129.1, 129.0, 128.5(6), 128.5(0), 127.7, 127.1, 123.7, 45.7, 26.1, 11.8. **IR** (film) v 2972, 2922, 1448, 1222, 1116, 1080, 848, 742, 630 cm^{-1}. **HRMS** (ESI) m/z: $[M+H]^+$ Calcd for : $C1_9H_{20}NOS$ 310.1260, found 310.1253.

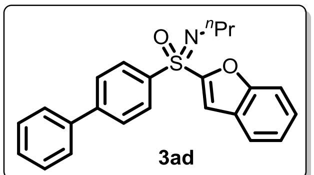

3ad

[1,1'-biphenyl]-4-yl(benzofuran-2-yl)(propylimino)-λ^6-sulfanone (**3ad**). Prepared following general procedure using *N*-propyl-[1,1'-biphenyl]-4-sulfonimidoyl fluoride **1k** (0.6 mmol, 166.4 mg), potassium benzofuran-2-yltrifluoroborate **2n** (0.4 mmol, 89.6 mg), TMSOTf (0.08 mmol, 14.4 μL) and CH_3CN (2.0 mL), the reaction was stirred at 40 °C for 30 min giving **3ad** in 92% yield (138.2 mg) as a yellow oil by column chromatography (PE/EA = 5:1, R_f = 0.38). **^{1}H NMR** (400 MHz, $CDCl_3$) δ 8.19 (d, *J* = 8.5 Hz, 2H), 7.68 – 7.64 (m, 2H), 7.61 (d, *J* = 7.8 Hz, 1H), 7.53 – 7.45 (m, 4H), 7.42 – 7.30 (m, 4H), 7.27 – 7.19 (m, 1H), 3.24 – 3.16 (m, 1H), 3.10 – 3.02 (m, 1H), 1.73 – 1.63 (m, 2H), 0.96 (t, *J* = 7.4 Hz, 3H). **^{13}C NMR** (101 MHz, $CDCl_3$) δ 156.3, 151.0, 145.9, 139.2, 137.5, 129.1, 128.8, 128.3, 127.6, 127.2(6), 127.2(0), 126.5, 123.8, 122.6, 114.4, 112.2, 46.1, 25.8, 11.7. **IR** (film) v 2955, 2870, 1593, 1533, 1259, 1136, 931, 790, 696, 680 cm^{-1}. **HRMS** (ESI) m/z: $[M+H]^+$ Calcd for : $C_{23}H_{22}NO_2S$ 376.1366, found 376.1357.

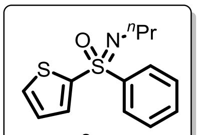

3ae

Phenyl(propylimino)(thiophen-2-yl)-λ^6-sulfanone (**3ae**). Prepared following general procedure using *N*-propylthiophene-2-sulfonimidoyl fluoride **1l** (0.6 mmol, 124.4 mg), potassium trifluoro(phenyl)borate **2a** (0.4 mmol, 73.6 mg), TMSOTf (0.08 mmol, 14.4 μL) and CH_3CN (2.0 mL), the reaction was stirred at 40 °C for 30 min giving **3ae** in 63% yield (66.8 mg) as a white solid by column chromatography (PE/EA = 5:1, R_f = 0.29). **^{1}H NMR** (400 MHz, $CDCl_3$) δ 8.06 – 8.01 (m, 2H), 7.56 (dd, *J* = 5.0, 1.4 Hz, 1H), 7.53 (dd, *J* = 3.7, 1.4 Hz, 1H), 7.50 – 7.43 (m, 3H), 7.03 (dd, *J* = 5.0, 3.8 Hz, 1H), 3.15 – 3.01 (m, 2H), 1.73 – 1.60 (m, 2H), 0.96 (t, *J* = 7.4 Hz, 3H). **^{13}C NMR** (101 MHz, $CDCl_3$) δ 143.0, 141.7, 133.5, 133.2, 132.3, 129.0, 128.0, 127.8, 45.9, 26.0, 11.8. **IR** (film) v 2957, 2928, 1446, 1253, 1126, 1010, 850, 752, 684, 599 cm^{-1}. **HRMS** (ESI) m/z: $[M+H]^+$ Calcd for : $C_{13}H_{16}NOS_2$ 266.0668, found 266.0660.

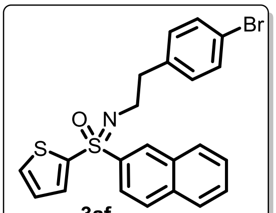

((4-bromophenethyl)imino)(naphthalen-2-yl)(thiophen-2-yl)-λ^6-sulfanone (**3af**). Prepared following general procedure using *N*-(4-bromophenethyl)thiophene-2-sulfonimidoyl fluoride **1m** (0.6 mmol, 209.8 mg), potassium trifluoro(naphthalen-2-yl)borate **2j** (0.4 mmol, 93.6 mg), TMSOTf (0.2 mmol, 36.0 µL) and CH_3CN (2.0 mL), the reaction was stirred at rt for 30 min giving **3af** in 82% yield (149.4 mg) as a white solid by column chromatography (PE/EA = 5:1, R_f = 0.29). **^{1}H NMR** (400 MHz, $CDCl_3$) δ 8.51 (d, *J* = 1.7 Hz, 1H), 7.92 (dd, *J* = 7.3, 2.0 Hz, 1H), 7.89 – 7.78 (m, 3H), 7.63 – 7.52 (m, 3H), 7.46 – 7.36 (m, 3H), 7.18 – 7.11 (m, 2H), 7.00 (dd, *J* = 5.0, 3.8 Hz, 1H), 3.38 (t, *J* = 7.2 Hz, 2H), 2.96 (m, 2H). **^{13}C NMR** (101 MHz, $CDCl_3$) δ 142.4, 139.4, 137.8, 134.6, 133.6, 133.2, 132.2, 131.1, 130.9, 129.3, 129.2, 129.2, 128.7, 127.9, 127.7, 127.2, 123.1, 119.7, 45.5, 38.6. **IR** (film) ν 3081, 2926, 2851, 2358, 2333, 1491, 1240, 1008, 813, 676 cm^{-1}. **HRMS** (ESI) m/z: $[M+H]^+$ Calcd for : $C_{22}H_{19}BrNOS_2$ 458.0242, 456.0086, found 458.0067, 456.0088.

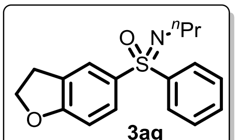

(2,3-dihydrobenzofuran-5-yl)(phenyl)(propylimino)-λ^6-sulfanone (**3ag**). Prepared following general procedure using *N*-propyl-2,3-dihydrobenzofuran-5-sulfonimidoyl fluoride **1n** (0.6 mmol, 146.0 mg), potassium trifluoro(phenyl)borate **2a** (0.4 mmol, 73.6 mg), TMSOTf (0.12 mmol, 21.6 µL) and CH_3CN (2.0 mL), the reaction was stirred at 40 °C for 30 min giving **3ag** in 90% yield (108.4 mg) as a colorless oil by column chromatography (PE/EA = 5:1, R_f = 0.21). **^{1}H NMR** (400 MHz, $CDCl_3$) δ 7.93 – 7.89 (m, 2H), 7.78 – 7.71 (m, 2H), 7.46 – 7.38 (m, 3H), 6.77 (d, *J* = 8.4 Hz, 1H), 4.58 (t, *J* = 8.8 Hz, 2H), 3.17 (t, *J* = 8.8 Hz, 2H), 3.04 – 2.90 (m, 2H), 1.70 – 1.57 (m, 2H), 0.94 (t, *J* = 7.4 Hz, 3H). **^{13}C NMR** (101 MHz, $CDCl_3$) δ 163.5, 141.7, 132.1, 131.7, 129.9, 128.8, 128.2, 128.1, 125.6, 109.4, 72.1, 45.6, 28.9, 26.0, 11.7. **IR** (film) ν 3068, 2953, 2901, 1602, 1483, 1288, 1224, 1103, 1062, 759, 601 cm^{-1}. **HRMS** (ESI) m/z: $[M+H]^+$ Calcd for : $C_{17}H_{20}NO_2S$ 302.1209, found 302.1201.

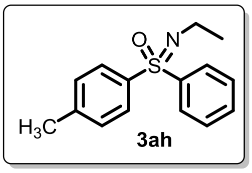

(Ethylimino)(phenyl)(*p*-tolyl)-λ^6-sulfanone (**3ah**). Prepared following general procedure using *N*-ethyl-4-methylbenzenesulfonimidoyl fluoride **1o** (0.6 mmol, 120.6 mg), potassium trifluoro(phenyl)borate **2a** (0.4 mmol, 73.6 mg), TMSOTf (0.08 mmol, 14.4 μL) and CH_3CN (2.0 mL), the reaction was stirred at 40 °C for 30 min giving **3ah** in 92% yield (95.4 mg) as a colorless oil by column chromatography (PE/EA = 5:1, R_f = 0.37). **^{1}H NMR** (400 MHz, $CDCl_3$) δ 7.94 (dd, *J* = 7.8, 1.9 Hz, 2H), 7.84 (d, *J* = 8.3 Hz, 2H), 7.48 – 7.38 (m, 3H), 7.23 (d, *J* = 8.2 Hz, 2H), 3.09 (q, *J* = 7.2 Hz, 2H), 2.34 (s, 3H), 1.25 (t, *J* = 7.2 Hz, 3H). **^{13}C NMR** (101 MHz, $CDCl_3$) δ 142.9, 141.1, 137.7, 132.0, 129.6, 128.9, 128.4, 128.2, 38.6, 21.3, 18.4. **IR** (film) v 2965, 2919, 2856, 1595, 1448, 1240, 1088, 813, 701 cm^{-1}. **HRMS** (ESI) m/z: $[M+H]^+$ Calcd for : $C_{15}H_{18}NOS$ 260.1104, found 260.1106.

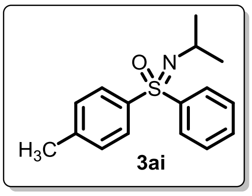

(Isopropylimino)(phenyl)(*p*-tolyl)-λ^6-sulfanone (**3ai**). Prepared following general procedure using *N*-isopropyl-4-methylbenzenesulfonimidoyl fluoride **1p** (0.6 mmol, 129.0 mg), potassium trifluoro(phenyl)borate **2a** (0.4 mmol, 73.6 mg), TMSOTf (0.08 mmol, 14.4 μL) and CH_3CN (2.0 mL), the reaction was stirred at 40 °C for 30 min giving **3ah** in 91% yield (99.9 mg) as a yellow solid by column chromatography (PE/EA = 5:1, R_f = 0.30). **^{1}H NMR** (400 MHz, $CDCl_3$) δ 7.95 (dd, *J* = 7.7, 2.0 Hz, 2H), 7.84 (d, *J* = 8.3 Hz, 2H), 7.48 – 7.38 (m, 3H), 7.23 (d, *J* = 8.1 Hz, 2H), 3.42 – 3.31 (m, 1H), 2.33 (s, 3H), 1.24 (d, *J* = 1.4 Hz, 3H), 1.23 (d, *J* = 1.4 Hz, 3H). **^{13}C NMR** (101 MHz, $CDCl_3$) δ 142.8, 141.5, 138.2, 131.9, 129.6, 128.8, 128.5, 128.3, 46.3, 26.7(1), 26.7(0), 21.2. **IR** (film) v 2965, 2921, 2862, 1596, 1448, 1137, 1093, 792, 698 cm^{-1}. **HRMS** (ESI) m/z: $[M+H]^+$ Calcd for : $C_{16}H_{20}NOS$ 274.1260, found 274.1259.

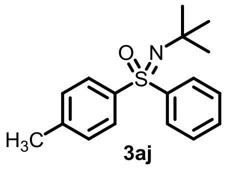
3aj

(*tert*-butylimino)(phenyl)(*p*-tolyl)-λ⁶-sulfanone (**3aj**). Prepared following general procedure using *N*-(*tert*-butyl)-4-methylbenzenesulfonimidoyl fluoride **1q** (0.6 mmol, 137.5 mg), potassium trifluoro(phenyl)borate **2a** (0.4 mmol, 73.6 mg), TMSOTf (0.08 mmol, 14.4 µL) and CH_3CN (2.0 mL), the reaction was stirred at 40 °C for 30 min giving **3aj** in 54% yield (62.0 mg) as a colorless oil by column chromatography (PE/EA = 10:1, R_f = 0.44). **^{1}H NMR** (400 MHz, $CDCl_3$) δ 7.99 – 7.93 (m, 2H), 7.84 (d, *J* = 8.3 Hz, 2H), 7.43 – 7.34 (m, 3H), 7.20 (d, *J* = 8.1 Hz, 2H), 2.34 (s, 3H), 1.28 (s, 9H). **^{13}C NMR** (101 MHz, $CDCl_3$) δ 145.3, 142.1, 142.0, 131.3, 129.3, 128.6, 127.9, 127.7, 54.9, 33.2, 21.2. **IR** (film) ν 2965, 2870, 1812, 1595, 1448, 1253, 1199, 1129, 812, 712 cm^{-1}. **HRMS** (ESI) m/z: $[M+H]^+$ Calcd for : $C_{17}H_{22}NOS$ 288.1417, found 288.1416.

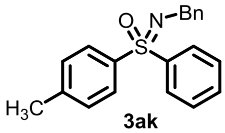
3ak

(Benzylimino)(phenyl)(*p*-tolyl)-λ⁶-sulfanone (**3ak**). Prepared following general procedure using *N*-benzyl-4-methylbenzenesulfonimidoyl fluoride **1r** (0.6 mmol, 158.0 mg), potassium trifluoro(phenyl)borate **2a** (0.4 mmol, 73.6 mg), TMSOTf (0.08 mmol, 14.4 µL) and CH_3CN (2.0 mL), the reaction was stirred at 40 °C for 30 min giving **3ak** in 85% yield (109.5 mg) as a colorless oil by column chromatography (PE/EA = 5:1, R_f = 0.33). **^{1}H NMR** (400 MHz, $CDCl_3$) δ 7.95 (dd, *J* = 7.9, 1.8 Hz, 2H), 7.85 (d, *J* = 8.4 Hz, 2H), 7.43 – 7.35 (m, 5H), 7.29 – 7.23 (m, 2H), 7.21 – 7.13 (m, 3H), 4.24 (s, 2H), 2.29 (s, 3H). **^{13}C NMR** (101 MHz, $CDCl_3$) δ 143.1, 141.5, 140.9, 137.5, 132.1, 129.6, 128.9, 128.5, 128.2, 128.0, 127.2, 126.2, 47.0, 21.2. **IR** (film) ν 3061, 3026, 2918, 2833, 1595, 1444, 1257, 1136, 1093, 844, 696 cm^{-1}. **HRMS** (ESI) m/z: $[M+H]^+$ Calcd for : $C_{20}H_{20}NOS$ 322.1260, found 322.1251.

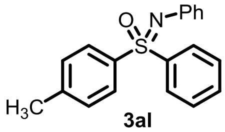
3al

Phenyl(phenylimino)(*p*-tolyl)-λ⁶-sulfanone (**3al**). Prepared following general procedure using 4-methyl-*N*-phenylbenzenesulfonimidoyl fluoride **1s** (0.6

mmol, 150.0 mg), potassium trifluoro(phenyl)borate **2a** (0.4 mmol, 73.6 mg), TMSOTf (0.08 mmol, 14.4 μL) and CH_3CN (2.0 mL), the reaction was stirred at 40 °C for 30 min giving **3al** in 64% yield (78.4 mg) as a white solid by column chromatography (PE/EA = 5:1, R_f = 0.45). **^{1}H NMR** (400 MHz, $CDCl_3$) δ 8.04 – 8.00 (m, 2H), 7.93 – 7.89 (m, 2H), 7.46 – 7.37 (m, 3H), 7.21 (d, *J* = 8.1 Hz, 2H), 7.17 – 7.07 (m, 4H), 6.87 – 6.81 (m, 1H), 2.31 (s, 3H). **^{13}C NMR** (101 MHz, $CDCl_3$) δ 144.7, 143.4, 141.2, 137.7, 132.3, 129.8, 129.1, 128.8, 128.5, 128.2, 123.6, 121.4, 21.3. **IR** (film) v 3059, 2922, 1593, 1444, 1265, 1199, 1072, 775, 754, 686 cm^{-1}. **HRMS** (ESI) m/z: $[M+H]^+$ Calcd for : $C_{19}H_{18}NOS$ 308.1104, found 308.1096.

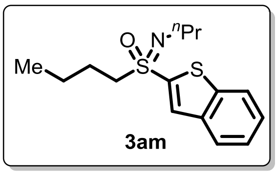

Benzo[*b*]thiophen-2-yl(butyl)(propylimino)-λ^6-sulfanone (**3am**). Prepared following general procedure using *N*-propylbutane-1-sulfonimidoyl fluoride **1t** (0.6 mmol, 109.0 mg), potassium benzo[*b*]thiophen-2-yltrifluoroborate **2o** (0.4 mmol, 96.0 mg), TMSOTf (0.12 mmol, 21.6 μL) and CH_3CN (2.0 mL), the reaction was stirred at 40 °C for 30 min giving **3am** in 90% yield (105.7 mg) as a colorless oil by column chromatography (PE/EA = 5:1, R_f = 0.34). **^{1}H NMR** (400 MHz, $CDCl_3$) δ 7.90 – 7.84 (m, 2H), 7.79 (s, 1H), 7.48 – 7.40 (m, 2H), 3.34 – 3.24 (m, 2H), 3.12 – 3.03 (m, 1H), 2.97 – 2.88 (m, 1H), 1.91 – 1.80 (m, 1H), 1.78 – 1.67 (m, 1H), 1.66 – 1.54 (m, 2H), 1.43 – 1.30 (m, 2H), 0.89 (m, 6H). **^{13}C NMR** (101 MHz, $CDCl_3$) δ 143.0, 141.0, 138.3, 131.1, 126.7, 125.4, 125.1, 122.6, 57.6, 45.8, 25.8, 24.8, 21.3, 13.4, 11.6. **IR** (film) v 2957, 2872, 1500, 1456, 1224, 1122, 976, 748, 725, 605 cm^{-1}. **HRMS** (ESI) m/z: $[M+H]^+$ Calcd for : $C_{15}H_{22}NOS_2$ 296.1137, found 296.1130.

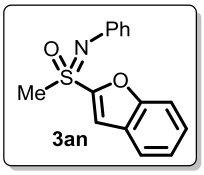

Benzofuran-2-yl(methyl)(phenylimino)- λ^6-sulfanone (**3an**). Prepared following general procedure using *N*-phenylmethanesulfonimidoyl fluoride **1u** (0.6 mmol, 104.0 mg), potassium benzofuran-2-yltrifluoroborate **2n** (0.4 mmol,

89.6 mg), TMSOTf (0.12 mmol, 21.6 µL) and CH_3CN (2.0 mL), the reaction was stirred at 40 °C for 30 min giving **3an** in 73% yield (79.3 mg) as a red solid by column chromatography (PE/EA = 5:1, R_f = 0.19). **^{1}H NMR** (400 MHz, $CDCl_3$) δ 7.64 (d, *J* = 7.6 Hz, 1H), 7.56 (d, *J* = 8.3 Hz, 1H), 7.49 – 7.41 (m, 2H), 7.36 – 7.28 (m, 1H), 7.18 – 7.12 (m, 2H), 7.13 – 7.06 (m, 2H), 6.98 – 6.88 (m, 1H), 3.43 (s, 3H). **^{13}C NMR** (101 MHz, $CDCl_3$) δ 156.1, 149.4, 143.7, 128.9, 127.7, 126.1, 124.1, 123.4, 122.8, 122.5, 115.0, 112.3, 44.7. **IR** (film) v 2926, 1593, 1487, 1294, 1211, 1078, 1045, 960, 792, 694 cm^{-1}. **HRMS** (ESI) m/z: $[M+H]^+$ Calcd for : $C_{15}H_{14}NO_2S$ 272.0740, found 272.0736.

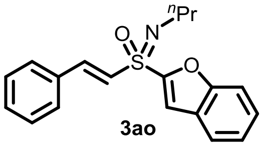

3ao

(*E*)-benzofuran-2-yl(propylimino)(styryl)-λ6-sulfanone (**3ao**). Prepared following general procedure using *(E)*-2-phenyl-*N*-propylethene-1-sulfonimidoyl fluoride **1v** (0.6 mmol, 136.0 mg), potassium benzofuran-2-yltrifluoroborate **2n** (0.4 mmol, 89.6 mg), TMSOTf (0.12 mmol, 21.6 µL) and CH_3CN (2.0 mL), the reaction was stirred at 40 °C for 30 min giving **3ao** in 96% yield (124.4 mg) as a yellow oil by column chromatography (PE/EA = 5:1, R_f = 0.41). **^{1}H NMR** (400 MHz, $CDCl_3$) δ 7.70 – 7.62 (m, 2H), 7.52 (d, *J* = 8.4 Hz, 1H), 7.48 – 7.43 (m, 3H), 7.43 – 7.36 (m, 1H), 7.36 – 7.22 (m, 4H), 7.05 (d, *J* = 15.3 Hz, 1H), 3.20 – 3.10 (m, 1H), 3.03 – 2.90 (m, 1H), 1.71 – 1.55 (m, 2H), 0.93 (t, *J* = 7.3 Hz, 3H). **^{13}C NMR** (101 MHz, $CDCl_3$) δ 156.2, 150.6, 144.1, 132.4, 130.9, 128.8, 128.4, 127.2, 126.5, 125.6, 123.8, 122.5, 114.0, 112.2, 45.9, 25.7, 11.6. **IR** (film) v 2958, 2928, 1710, 1533, 1444, 1261, 1068, 927, 815, 738, 611 cm^{-1}. **HRMS** (ESI) m/z: $[M+H]^+$ Calcd for : $C_{19}H_{20}NO_2S$ 326.1209, found 326.1204.

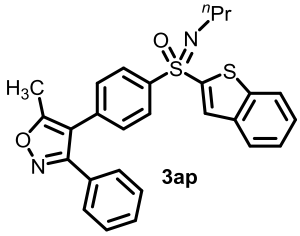

3ap

Benzo[*b*]thiophen-2-yl(4-(5-methyl-3-phenylisoxazol-4-yl)phenyl)(propylimino)-λ^6-sulfanone (**3ap**). Prepared following general procedure using 4-(5-methyl-3-phenylisoxazol-4-yl)-*N*-propylbenzene sulfonimidoyl fluoride **1w** (0.3 mmol, 108.0 mg),

potassium benzo[*b*]thiophen-2-yltrifluoroborate **2o** (0.2 mmol, 48.0 mg), TMSOTf (0.06 mmol, 10.8 µL) and CH_3CN (1.0 mL), the reaction was stirred at 40 °C for 30 min giving **3ap** in 97% yield (102.0 mg) as a red oil by column chromatography (PE/EA = 2:1, R_f = 0.57). **^{1}H NMR** (400 MHz, $CDCl_3$) δ 8.09 (d, *J* = 8.5 Hz, 2H), 7.88 – 7.76 (m, 3H), 7.45 – 7.32 (m, 5H), 7.31 – 7.21 (m, 4H), 3.27 – 3.05 (m, 2H), 2.41 (s, 3H), 1.78 – 1.64 (m, 2H), 0.99 (t, *J* = 7.4 Hz, 3H). **^{13}C NMR** (101 MHz, $CDCl_3$) δ 167.2, 160.9, 143.1, 142.9, 140.1, 138.3, 135.1, 130.7, 130.1, 129.5, 128.5(9), 128.5(2), 128.3, 126.8, 125.5, 125.1, 122.6, 114.3, 46.0, 25.9, 11.7, 11.6. **IR** (film) v 2958, 2872, 1710, 1618, 1464, 1257, 1141, 977, 773, 725, 623 cm^{-1}. **HRMS** (ESI) m/z: $[M+H]^+$ Calcd for : $C_{27}H_{25}N_2O_2S_2$ 473.1346, found 473.1341.

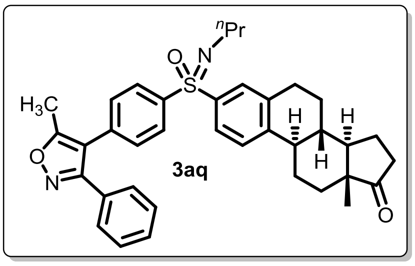

((8*S*,9*R*,13*R*,14*R*)-13-methyl-17-*oxo*-7,8,9,11,12,13,14,15,16,17-decahydro-6*H*-cyclopenta[*a*]phenanthren-3-yl)(4-(5-methyl-3-phenylisoxazol-4-yl)phenyl)(propylimino)-λ^6-sulfanone (**3aq**). Prepared following general procedure using 4-(5-methyl-3-phenylisoxazol-4-yl)-*N*-propylbenzene sulfonimidoyl fluoride **1w** (0.3 mmol, 108.0 mg), potassium trifluoro((8*S*,9*R*,13*R*,14*R*)-13-methyl-17-*oxo*-7,8,9,11,12,13,14,15,16,17deca-hydro-6*H*-cyclopenta[*a*]phenanthren-3-yl)borate **2u** (0.2 mmol, 90.8 mg), TMSOTf (0.06 mmol, 10.8 µL) and CH_3CN (1.0 mL), the reaction was stirred at 40 °C for 30 min giving **3aq** in 55% yield (65.3 mg) as a red oil by column chromatography (PE/EA = 2:1, R_f = 0.29). **^{1}H NMR** (400 MHz, $CDCl_3$) δ 7.96 (d, *J* = 8.4 Hz, 2H), 7.71 – 7.66 (m, 2H), 7.43 – 7.33 (m, 4H), 7.33 – 7.22 (m, 4H), 3.06 – 2.92 (m, 4H), 2.55 – 2.37 (m, 5H), 2.36 – 2.25 (m, 1H), 2.19 – 1.91 (m, 4H), 1.72 – 1.39 (m, 8H), 0.99 – 0.92 (m, 3H), 0.89 (d, *J* = 2.2 Hz, 3H). **^{13}C NMR** (101 MHz, $CDCl_3$) δ 167.0, 160.9, 144.8, 140.5, 140.4, 137.9, 137.4(5), 137.4(3), 134.5, 130.1, 129.5, 129.1, 128.6(5), 128.6(4), 128.5, 128.4, 128.3, 126.2, 125.9, 114.4, 50.3, 47.7, 45.7(2), 45.7(1), 44.4, 37.5, 35.6, 31.4, 29.3(2), 29.3(0), 26.0(9), 26.0(6), 26.0(4), 25.4, 21.4, 13.7, 11.8, 11.6. **IR** (film) v 2924, 2870, 1734, 1452, 1224, 1138, 1080, 773, 727, 632 cm^{-1}. **HRMS** (ESI) m/z:

[M+H]$^+$ Calcd for : $C_{37}H_{41}N_2O_3S$ 593.2821, found 593.2812.

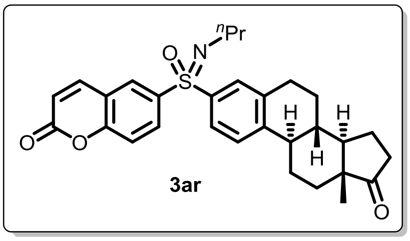

6-((8*S*,9*R*,13*R*,14*R*)-13-methyl-17-*oxo*-*N*-propyl-7,8,9,11,12,13,14,15,16,17-decahydro-6*H*-cyclopenta[*a*]phenanthrene-3-sulfonimidoyl)-2*H*-chromen-2-one (**3ar**). Prepared following general procedure using 2-*oxo*-*N*-propyl-2*H*-chromene-6-sulfonimidoyl fluoride **1x** (0.3 mmol, 80.8 mg), potassium trifluoro((8*S*,9*R*,13*R*,14*R*)-13-methyl-17-*oxo*-7,8,9,11,12,13,14,15,16,17-decahydro-6*H*-cyclopenta[*a*]phenanthren-3-yl)borate **2u** (0.2 mmol, 90.8 mg), TMSOTf (0.06 mmol, 10.8 µL) and CH_3CN (1.0 mL), the reaction was stirred at 40 °C for 30 min giving **3ar** in 92% yield (92.7 mg) as a white solid by column chromatography (PE/EA = 2:1, R_f = 0.51). **^{1}H NMR** (400 MHz, $CDCl_3$) δ 8.13 (d, *J* = 2.2 Hz, 1H), 7.99 (dd, *J* = 8.7, 2.2 Hz, 1H), 7.72 (d, *J* = 9.7 Hz, 1H), 7.66 – 7.60 (m, 2H), 7.33 (d, *J* = 7.8 Hz, 1H), 7.30 (d, *J* = 8.8 Hz, 1H), 6.41 (d, *J* = 9.7 Hz, 1H), 2.97 – 2.85 (m, 4H), 2.47 – 2.37 (m, 1H), 2.36 – 2.28 (m, 1H), 2.26 – 2.18 (m, 1H), 2.11 – 1.93 (m, 3H), 1.90 – 1.84 (m, 1H), 1.64 – 1.32 (m, 8H), 0.91 – 0.86 (m, 3H), 0.80 (d, *J* = 2.3 Hz, 3H). **^{13}C NMR** (101 MHz, $CDCl_3$) δ 159.2, 155.8, 144.9, 142.6, 137.9, 137.5(6), 137.5(4), 137.2(4), 137.2(2), 131.2, 128.5, 126.1, 125.4, 118.7, 117.5(7), 117.5(3), 50.1, 47.4, 44.5, 44.1, 37.3, 35.4, 31.2, 29.0, 25.8, 25.7, 25.2, 21.2, 13.5, 11.6. **IR** (film) ν 2930, 2870, 1736, 1622, 1599, 1427, 1136, 1105, 702, 659 cm^{-1}. **HRMS** (ESI) m/z: [M+H]$^+$ Calcd for : $C_{30}H_{34}NO_4S$ 504.2203, found 504.2194.

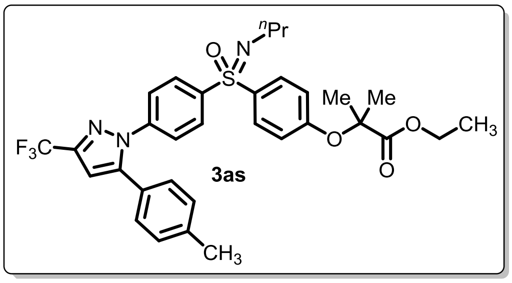

Ethyl 2-methyl-2-(4-(*N*-propyl-4-(5-(ptolyl)-3-(trifluoromethyl)-1*H*-pyrazol-1-yl)phenylsulfonimidoyl)phenoxy)propanoate (**3as**). Prepared following general procedure using *N*-propyl-4-(5-(*p*-tolyl)-3-(trifluoromethyl)-1*H*-pyrazol-1-yl)benzenesulfonimidoy

l fluoride **1y** (0.3 mmol, 128.0 mg), potassium (4-((1-ethoxy-2-methyl-1-oxopropan-2-yl)oxy)phenyl)trifluoroborate **2v** (0.2 mmol, 62.8 mg), TMSOTf (0.10 mmol, 18.0 μL) and CH_3CN (1.0 mL), the reaction was stirred at rt for 30 min giving **3as** in 54% yield (66.7 mg) as a white solid by column chromatography (PE/EA = 5:1, R_f = 0.26). **^{1}H NMR** (400 MHz, $CDCl_3$) δ 7.92 (dd, *J* = 8.8, 2.1 Hz, 2H), 7.79 (dd, *J* = 8.9, 2.1 Hz, 2H), 7.40 (dd, *J* = 8.7, 2.1 Hz, 2H), 7.17 – 7.04 (m, 4H), 6.83 (dd, *J* = 9.0, 2.2 Hz, 2H), 6.70 (s, 1H), 4.25 – 4.16 (m, 2H), 2.98 (t, *J* = 7.1 Hz, 2H), 2.36 (s, 3H), 1.73 – 1.52 (m, 8H), 1.24 – 1.14 (m, 3H), 1.00 – 0.90 (m, 3H). **^{13}C NMR** (101 MHz, $CDCl_3$) δ 173.4, 159.2, 145.1, 143.8 (q, *J* = 38.8 Hz), 142.0, 141.0, 139.6, 132.3, 130.3, 129.6, 129.3, 128.6, 125.6, 125.4, 121.0 (q, *J* = 280.78 Hz), 118.0, 106.0, 79.5, 61.6, 45.6, 26.0, 25.2(9), 25.2(4), 21.2, 13.9, 11.8. **^{19}F NMR** (376 MHz, $CDCl_3$) δ -62.4. **IR** (film) v 2960, 2874, 1734, 1591, 1471, 1234, 1130, 1095, 974, 970, 742 cm^{-1}. **HRMS** (ESI) m/z: $[M+H]^+$ Calcd for : $C_{32}H_{35}F_3N_3O_4S$ 614.2295, found 614.2283.

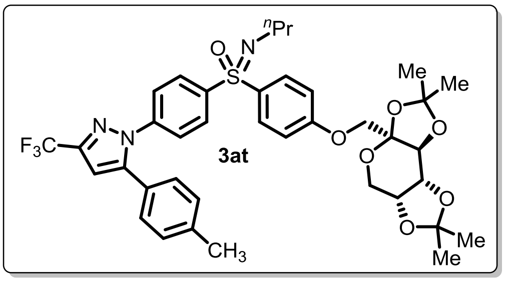

(Propylimino)(4-(((3a*S*,5a*R*,8a*R*,8b*S*)-2,2,7,7-tetramethyltetrahydro-3a*H*-bis([1,3]dioxolo)[4,5-*b*:4',5'-*d*]pyran-3*a*-yl)methoxy)phenyl)(4-(5-(*p*-tolyl)-3-(trifluoromethyl)-1*H*-pyrazol-1-yl)phenyl)-λ^6-sulfanone (**3at**). Prepared following general procedure using *N*-propyl-4-(5-(*p*-tolyl)-3-(trifluoromethyl)-1*H*-pyrazol-1-yl)benzenesulfonicmidoyl fluoride **1y** (0.3 mmol, 128.0 mg), potassium trifluoro(4-(((3a*S*,5a*R*,8a*R*,8b*S*)-2,2,7,7-tetramethyltetrahydro-3a*H*-bis([1,3]di-oxolo)[4,5-*b*:4',5'-*d*]pyran-3*a*-yl)methoxy)phenyl)borate **2w** (0.2 mmol, 88.5 mg), TMSOTf (0.10 mmol, 18.0 μL) and CH_3CN (1.0 mL), the reaction was stirred at rt for 30 min giving **3at** in 56% yield (83.2 mg) as a white solid by column chromatography (PE/EA = 5:1, R_f = 0.14). **^{1}H NMR** (400 MHz, $CDCl_3$) δ 7.92 (d, *J* = 7.9 Hz, 2H), 7.84 (dd, *J* = 9.0, 1.1 Hz, 2H), 7.39 (d, *J* = 8.7 Hz, 2H), 7.13 (d, *J* = 7.9 Hz, 2H), 7.07 (d, *J* = 8.2 Hz, 2H), 6.98 (d, *J* = 8.8 Hz, 2H), 6.70

(s, 1H), 4.62 (dd, J = 7.9, 2.6 Hz, 1H), 4.48 (d, J = 2.6 Hz, 1H), 4.25 (dd, J = 7.9, 1.6 Hz, 1H), 4.18 (dd, J = 10.3, 0.9 Hz, 1H), 4.06 (dd, J = 10.3, 1.1 Hz, 1H), 3.95 (dd, J = 13.0, 1.9 Hz, 1H), 3.77 (d, J = 13.0 Hz, 1H), 2.98 (t, J = 7.1 Hz, 2H), 2.36 (s, 3H), 1.71 – 1.57 (m, 2H), 1.54 (s, 3H), 1.46 (s, 3H), 1.41 (s, 3H), 1.33 (s, 3H), 0.94 (t, J = 7.4 Hz, 3H). **^{13}C NMR** (101 MHz, $CDCl_3$) δ 161.8(3), 161.8(1), 145.0, 143.8 (q, J = 38.5 Hz), 142.0, 141.1, 139.5, 132.0, 130.7, 129.6, 129.2, 128.6, 125.6, 125.3, 121.0 (q, J = 269.0 Hz), 115.1, 109.0, 108.9, 106.0, 101.7, 70.8, 70.0, 69.9, 69.0(5), 69.0(2), 61.2, 45.6, 26.4, 26.0, 25.8, 25.2, 23.9, 21.2, 11.7. **^{19}F NMR** (376 MHz, $CDCl_3$) δ -62.4. **IR** (film) ν 2989, 2933, 2874, 1712, 1591, 1371, 1234, 1134, 1068, 731 cm^{-1}. **HRMS** (ESI) m/z: $[M+H]^+$ Calcd for : $C_{38}H_{43}F_3N_3O_7S$ 742.2768, found 742.2753.

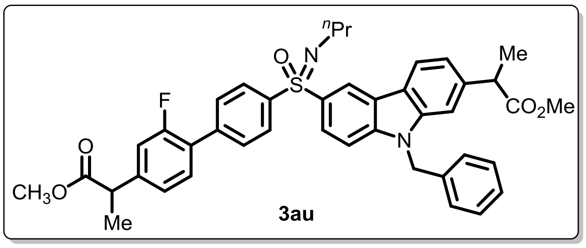

3au

Methyl 2-(9-benzyl-6-(2'-fluoro-4'-(1-methoxy-1-oxopropan-2-yl)-*N*-propyl-[1,1'-biphenyl]-4-sulfonimidoyl)-9*H*-carbazol-2-yl)propanoate (**3au**). Prepared following general procedure using methyl 2-(2-fluoro-4'-(*S*-fluoro-*N*-propylsulfonimidoyl)-[1,1'-biphenyl]-4-yl)propanoate **1z** (0.3 mmol, 114.4 mg), potassium (9-benzyl-7-(1-methoxy-1-oxopropan-2-yl)-9*H*-carbazol-3-yl)trifluoroborate **2x** (0.2 mmol, 89.9 mg), TMSOTf (0.10 mmol, 18.0 μL) and CH_3CN (1.0 mL), the reaction was stirred at rt for 30 min giving **3au** in 51% yield (71.6 mg) as a red oil by column chromatography (PE/EA = 2:1, R_f = 0.34). **^{1}H NMR** (400 MHz, $CDCl_3$) δ 8.77 (d, J = 1.8 Hz, 1H), 8.10 (d, J = 8.1 Hz, 1H), 8.08 – 8.03 (m, 2H), 8.00 (dd, J = 8.7, 1.9 Hz, 1H), 7.58 (dd, J = 8.5, 1.6 Hz, 2H), 7.39 – 7.29 (m, 3H), 7.27 – 7.20 (m, 4H), 7.14 – 7.06 (m, 4H), 5.48 (s, 2H), 3.86 (q, J = 7.1 Hz, 1H), 3.73 (q, J = 7.2 Hz, 1H), 3.66 (s, 3H), 3.60 (s, 3H), 3.12 – 3.05 (m, 2H), 1.77 – 1.67 (m, 2H), 1.53 (d, J = 7.1 Hz, 3H), 1.50 (d, J = 7.2 Hz, 3H), 0.99 (t, J = 7.3 Hz, 3H). **^{13}C NMR** (101 MHz, $CDCl_3$) δ 174.8, 174.1, 159.5 (d, J = 249.5 Hz), 142.8, 142.7, 141.6, 141.0, 139.8, 139.0, 136.0, 130.7, 130.6 (d, J = 3.7

Hz), 129.4 (d, J = 3.2 Hz), 128.8, 128.4, 127.7, 126.3, 126.0, 123.7 (d, J = 3.3 Hz), 123.0, 121.9, 121.7, 121.0, 120.1, 115.4, 115.2, 109.3, 108.2, 52.1, 52.0, 46.7, 45.9, 45.8, 44.8, 26.2, 18.9, 18.3, 11.8. **^{19}F NMR** (376 MHz, $CDCl_3$) δ -117.0. **IR** (film) v 2953, 2872, 1732, 1595, 1496, 1286, 1124, 848, 823, 727 cm^{-1}. **HRMS** (ESI) m/z: $[M+H]^+$ Calcd for : $C_{42}H_{42}FN_2O_5S$ 705.2793, found 705.2778.

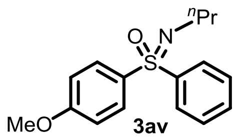

3av

(4-methoxyphenyl)(phenyl)(propylimino)-λ^6-sulfanone (**3av**). Prepared following general procedure using 4-methoxy-*N*-propylbenzenesulfonimidoyl fluoride **1h** (0.6 mmol, 138.6 mg), *N*-propyl-4-(trifluoromethyl)benzene sulfonimidoyl fluoride **1h`** (0.6 mmol, 161.4 mg), potassium trifluoro(phenyl)borate **2a** (0.4 mmol, 73.6 mg), TMSOTf (0.08 mmol, 14.4 μL) and CH_3CN (2.0 mL), the reaction was stirred at 40 °C for 30 min giving **3av** in 73% yield (84.4 mg) as a colorless solid by column chromatography (PE/EA = 5:1, R_f = 0.17). **^{1}H NMR** (400 MHz, $CDCl_3$) δ 8.07 – 7.74 (m, 4H), 7.48 – 7.35 (m, 3H), 6.91 (d, J = 8.9 Hz, 2H), 3.77 (s, 3H), 2.98 (t, J = 7.1 Hz, 2H), 1.75 – 1.51 (m, 2H), 0.94 (t, J = 7.4 Hz, 3H). **^{13}C NMR** (101 MHz, $CDCl_3$) δ 162.5, 141.4, 132.1, 131.8, 130.4, 128.8, 128.0, 114.1, 55.3, 45.5, 26.0, 11.7. **IR** (film) v 2957, 2927, 2842, 1587, 1303, 1249, 1134, 717, 695 cm^{-1}. **HRMS** (ESI) m/z: $[M+H]^+$ Calcd for : $C_{16}H_{20}NO_2S$ 290.1209, found 290.1208.

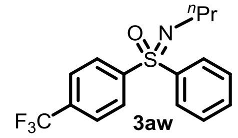

3aw

Phenyl(propylimino)(4-(trifluoromethyl)phenyl)-λ^6-sulfanone (**3aw**). Prepared following general procedure using 4-methoxy-*N*-propylbenzenesulfonimidoyl fluoride **1h** (0.6 mmol, 138.6 mg), *N*-propyl-4-(trifluoromethyl)benzenesulfonimidoyl fluoride, **1h`** (0.6 mmol, 161.4 mg), potassium trifluoro(phenyl)borate **2a** (0.4 mmol, 73.6 mg), TMSOTf (0.08 mmol, 14.4 μL) and CH_3CN (2.0 mL), the reaction was stirred at 40 °C for 30 min giving **3av** in 73% yield (31.4 mg) as a colorless solid by column chromatography (PE/EA = 5:1, R_f = 0.38). **^{1}H NMR** (400 MHz, $CDCl_3$) δ 8.09 (d, J = 8.2 Hz, 2H), 8.01 – 7.96 (m, 2H), 7.72 (d, J = 8.3 Hz, 2H), 7.57 – 7.46 (m,

3H), 3.09 – 2.93 (m, 2H), 1.73 – 1.61 (m, 2H), 0.97 (t, *J* = 7.4 Hz, 3H). **^{13}C NMR** (101 MHz, $CDCl_3$) δ 144.9, 140.1, 133.9 (q, *J* = 33.0 Hz), 132.7, 129.2, 129.0, 128.6, 126.1 (q, *J* = 3.7 Hz), 123.3 (q, *J* = 272.8 Hz), 45.6, 26.0, 11.8. **IR** (film) v 2927, 2848, 1400, 1319, 1063, 844, 723, 603 cm^{-1}. **HRMS** (ESI) m/z: $[M+H]^+$ Calcd for : $C_{16}H_{17}F_3NOS$ 328.0977, found 328.0978.

V. X-Ray Crystal Structure

Supplementary Table 1. Sample and crystal data for **3m** (CCDC 2240513)**.**

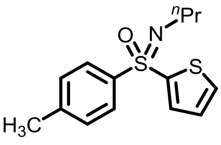

3m

=

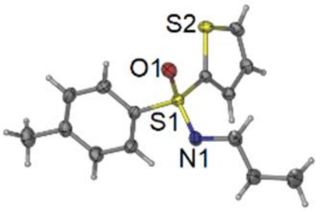

The ellipsoid contour probability level is 50%.

ORTEP representation of **3m**
CCDC 2240513

Bond precision	C-C = 0.0028 A	
	Wavelength = 1.54184	
Cell	*a* = 5.9748(2)	α = 89.999(4)
	b = 7.8641(4)	β = 80.991(3)
	c = 16.8240(7)	γ = 68.477(4)
Temperature	173 K	
Volume	724.89(6)	
Space group	P -1	
Sum formula	C14 H17 N O S2	
Mr	279.40	
Dx,g cm^{-3}	1.280	
Z	2	
Mu (mm-1)	3.224	
F000	296.0	
h,k,lmax	7,9,20	
Nref	2546	
Tmin,Tmax	0.520, 1.000	
Correction method= # Reported T Limits	Tmin=0.520 Tmax=1.000	
AbsCorr = MULTI-SCAN		
Data completeness	0.982	
Theta(max)	67.080	
R(reflections)	0.0323(2398)	
wR_2(reflections)	0.0916(2546)	
S	1.094	
Npar	165	

VI. Fluorine spectrum tracking experiment

Under nitrogen atmosphere, dry CD_3CN (1 mL, 0.2 M) was added to a dry 10 mL oven-dried Schlenk tube, which was equipped with sulfonimidoyl fluoride **1a** or potassium aryltrifluoroborate salt **2a**. TMSOTf was dropwise added via the mircosyringe at room temperature. Then the reaction mixture was stirred at 40 ^{o}C for 5 min, and the fluorine spectra tracking experiments were conducted.

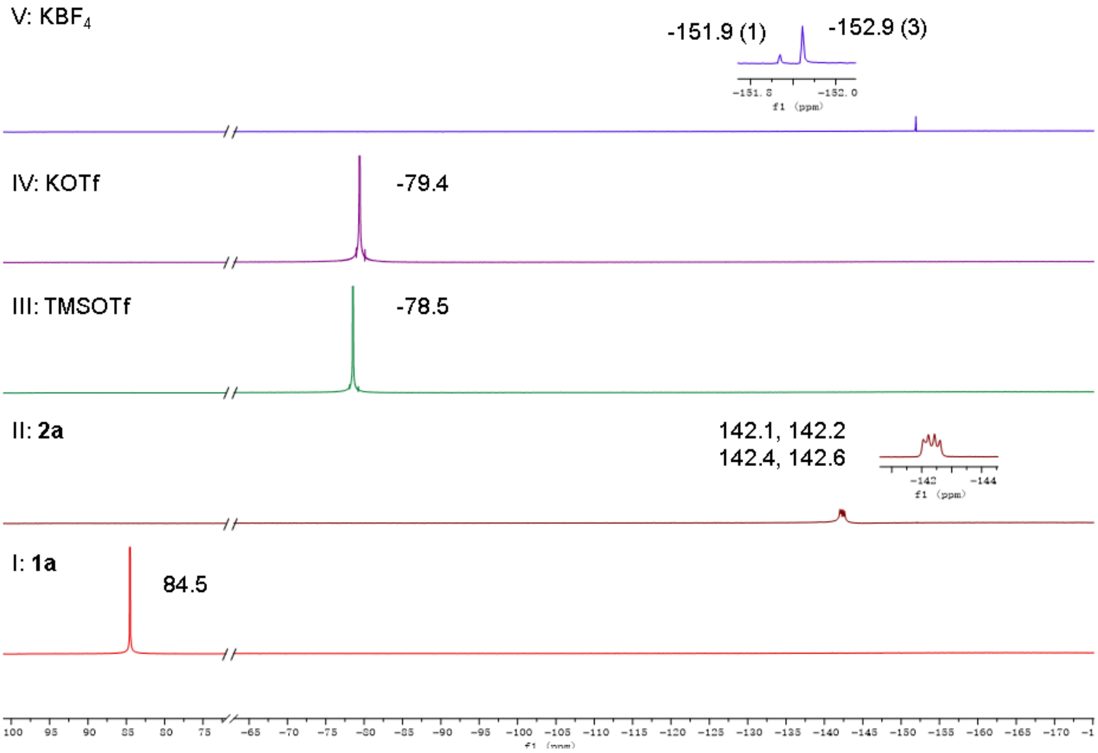

Supplementary Figure 5. Fluorine spectra involving I-V experiments.

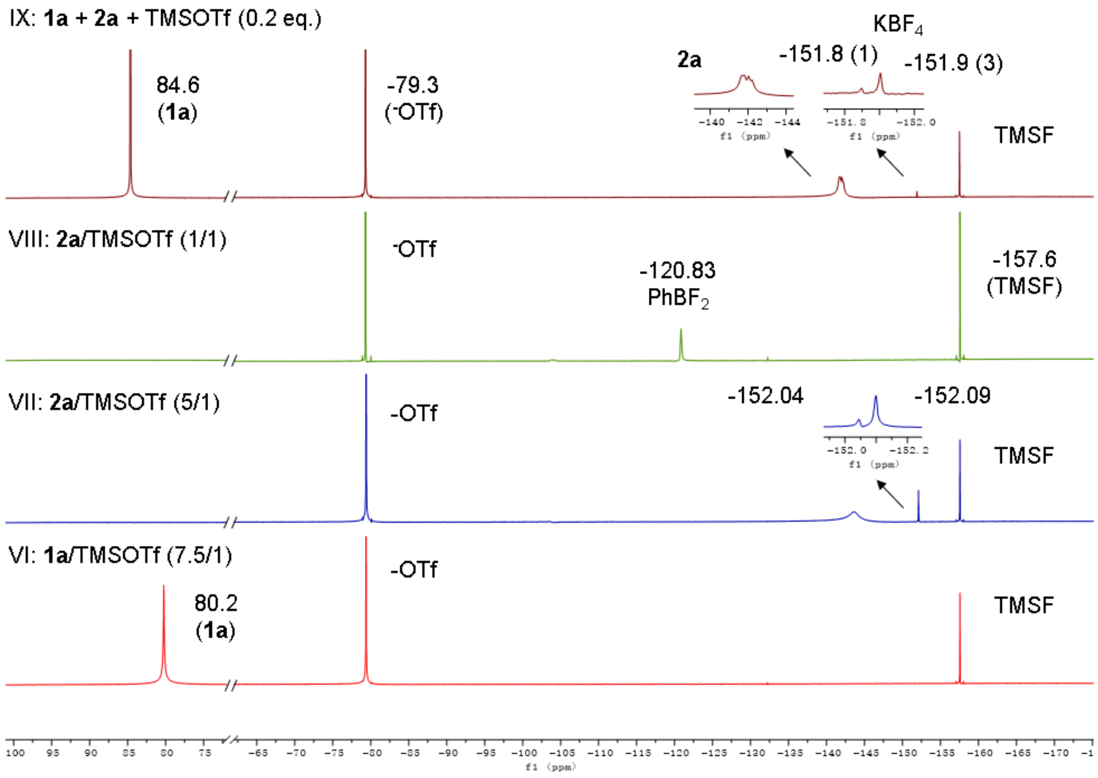

Supplementary Figure 6. Fluorine spectra involving VI-IX experiments.

VII. Compuational details of DFT calculations

All calculations were performed with the Gaussian 16[4] package. Geometry optimizations and frequency calculations were performed with M06-2X[5] and the 6-311+G(d,p) basis set with the SMD solvation model.[6] Meanwhile, transition states structures were verified by frequency calculations and only one imaginary frequency was found in the transition states. Intrinsic Reaction Coordinate (IRC) was utilized to confirm the reaction pathway. All optimized structures were visualized using CYLView program.[7]

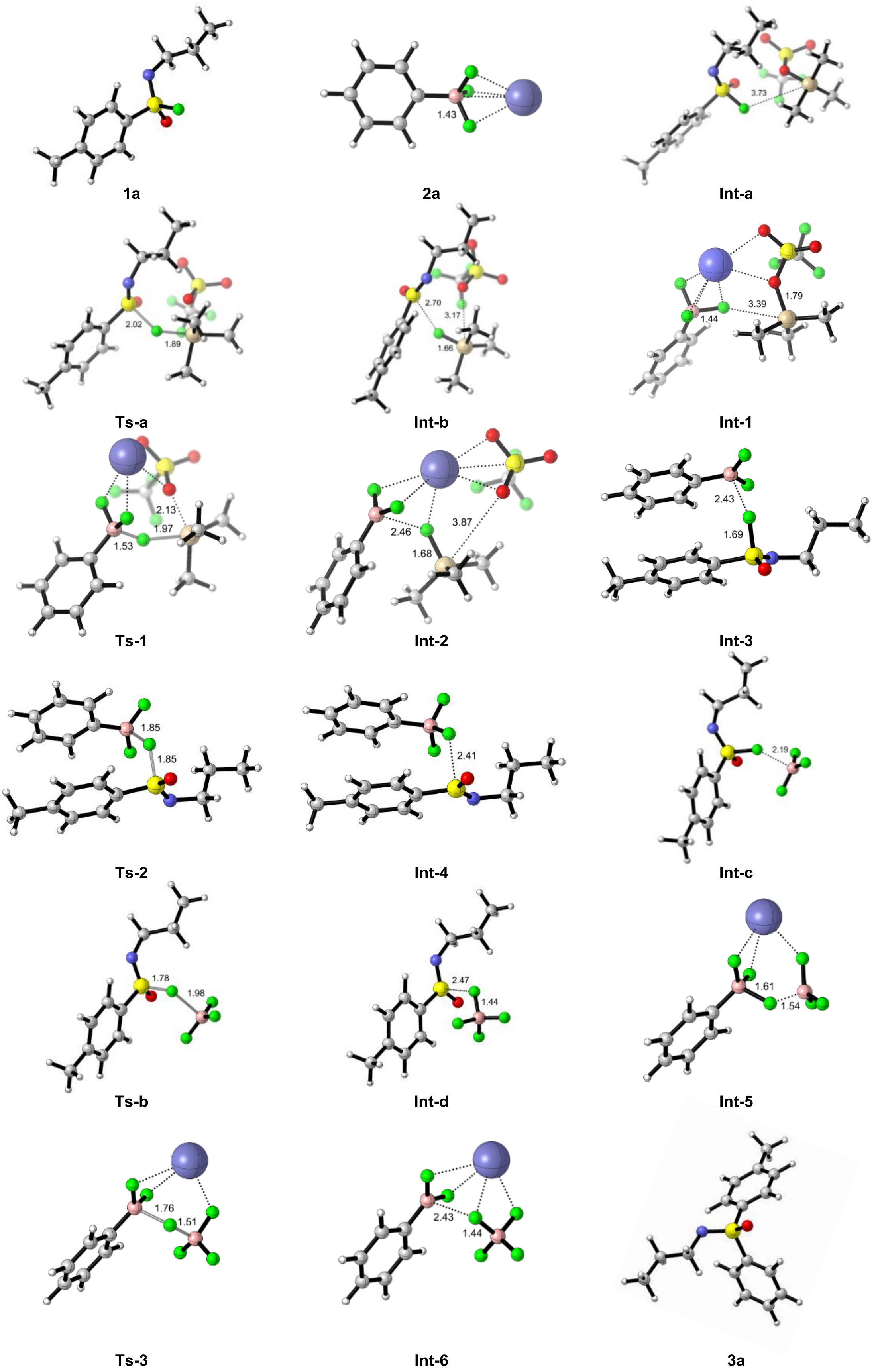

Supplementary Figure 7. Calculated structures including **1a**, **2a**, intermediates, transition states and **3a**.

Supplementary Table 2. Calculated energy data for all structures at the level of M06-2X/6-311+G(d,p)/SMD(CH_3CN).

	H (T) (Hartree)	ΔH (kcal/mol)	G (T) (Hartree)	ΔG (kcal/mol)
**R^1**	-3543.66236	0.0	-3543.83189	0.0
**Int-a**	-3543.66871	-4.0	-3543.81563	10.2
**Ts-a**	-3543.63252	18.7	-3543.77557	35.3
**Int-b**	-3543.66791	-3.5	-3543.81862	8.3
**Int-1**	-3543.67890	-10.4	-3543.83216	-0.2
**Ts-1**	-3543.64606	10.2	-3543.79149	25.3
**Int-2**	-3543.67878	-10.3	-3543.83093	0.6
**Int-3**	-3543.66225	0.07	-3543.82936	1.6
**Ts-2**	-3543.65783	2.8	-3543.82565	3.9
**Int-4**	-3543.66635	-2.5	-3543.83486	-1.9
**3a**	-3543.69521	-20.6	-3543.88149	-31.0
**R^2**	-2497.68722	0.0	-2497.82601	0.0
**Int-c**	-2497.69649	-5.8	-2497.81945	4.1
**Ts-b**	-2497.69747	-6.4	-2497.81961	4.0
**Int-d**	-2497.71391	-16.7	-2497.83762	-7.3
**Int-5**	-2497.71542	-17.7	-2497.83465	-6.8
**Ts-3**	-2497.71487	-17.3	-2497.83963	-5.4
**Int-6**	-2497.71729	-18.9	-2497.83963	-8.5

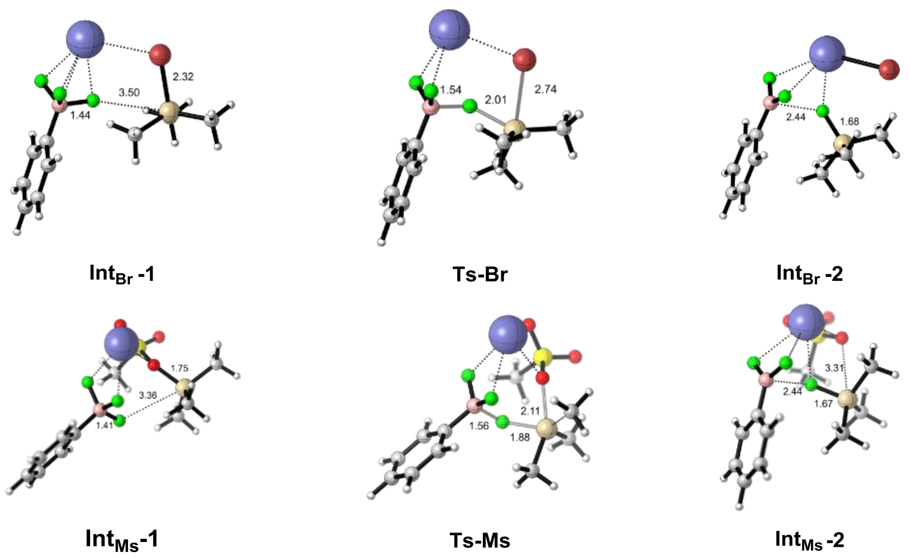

Supplementary Figure 8. Calculated Structures including intermediates and transition states of TMSBr or TMSOTf as Lewis acid.

Supplementary Table 3. Calculated energy data for structures at the level of M06-2X/6-311+G(d,p)/SMD(CH_3CN).

	H (T) (Hartree)	ΔH (kcal/mol)	G (T) (Hartree)	ΔG (kcal/mol)
**1a+2a+TMSBr**	-5156.47693	0.0	-5156.63015	0.0
**Int$_{Br}$-1**	-5156.48526	-5.2	-5156.62234	4.9
**Ts-Br**	-5156.44550	19.7	-5156.57745	33.1
**Int$_{Br}$-2**	-5156.48109	-22.3	-5156.61814	7.5
**1a+2a+TMSOMs**	-3245.93940	0.0	-3246.10224	0.0
**Int$_{Ms}$-1**	-3245.96098	-13.5	-3246.10456	-1.4
**Ts-Ms**	-3245.92154	11.2	-3246.06058	26.1
**Int$_{Ms}$-2**	-3245.93845	0.60	-3246.08403	11.4

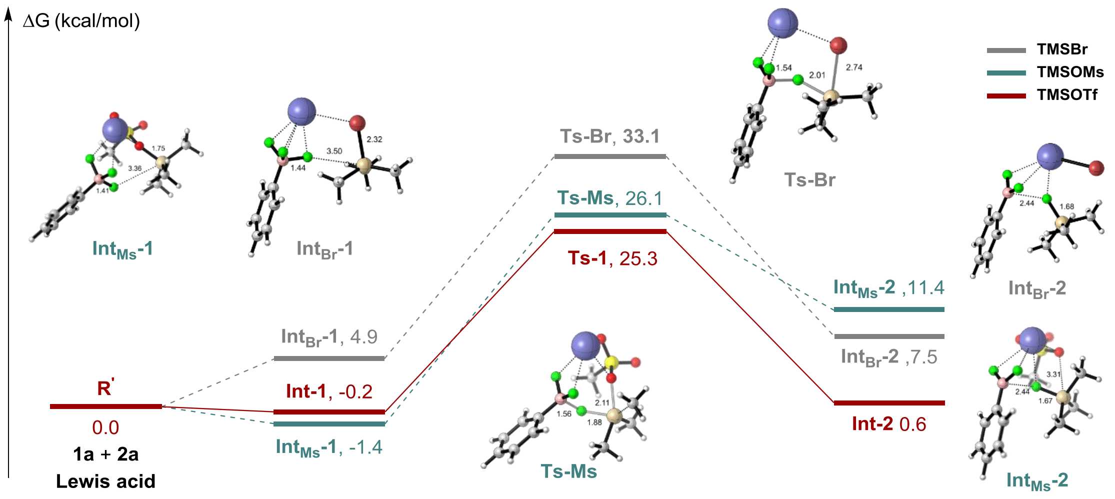

Supplementary Figure 9. Density functional theory (DFT) calculation of the activation of **2a** with TMSOTf, TMSBr and TMSOMs.

Cartesian coordinates for all optimized structures

1a

Imaginary frequency: none

C	3.23831800	0.92271100	-0.23463000
C	1.87936100	1.21804400	-0.22336400
C	0.98217100	0.17758000	-0.02957100
C	1.39592100	-1.13925200	0.15881500
C	2.75398700	-1.40605200	0.14459500
C	3.69213600	-0.38355700	-0.05215600
H	3.95394900	1.72236700	-0.38801800
H	1.52745700	2.23255600	-0.36578600
H	0.67040800	-1.92975100	0.30513500
H	3.09793800	-2.42442700	0.28891000
C	5.15961600	-0.70613400	-0.07506400
H	5.44026200	-1.28244000	0.80934100
H	5.39981200	-1.31414100	-0.95149300
H	5.76308000	0.20085200	-0.10940300
S	-0.73079000	0.56684600	-0.01433200
O	-0.97786200	1.96896200	-0.29171700
N	-1.47163700	-0.56487600	-0.65328100
C	-2.91754000	-0.45667800	-0.92623200
H	-3.14267400	-1.24679100	-1.64390800
H	-3.14898800	0.50188600	-1.40253200
C	-3.77323400	-0.63733900	0.32419400
H	-3.47187700	-1.56155700	0.82556000
H	-3.57856800	0.18557600	1.01907700
C	-5.25663800	-0.68103800	-0.02743900
H	-5.47346000	-1.51737500	-0.69729700
H	-5.86857200	-0.79981000	0.86873000

H	-5.56712500	0.24036900	-0.52750200
F	-0.96769200	0.51954200	1.63892300

2a

Imaginary frequency: none

C	3.09516400	-1.20526600	0.00262900
C	1.70253200	-1.19736400	-0.00998100
C	0.98120500	0.00219100	-0.01367400
C	1.70561700	1.19938000	-0.01023900
C	3.09860700	1.20325400	0.00199600
C	3.79665600	-0.00183800	0.00952200
H	3.63430600	-2.14642800	0.00347300
H	1.16581300	-2.14147800	-0.02445700
H	1.17130000	2.14465500	-0.02467000
H	3.64030400	2.14295100	0.00218800
H	4.88105200	-0.00338700	0.01679100
B	-0.61209200	0.00128900	-0.00071500
F	-1.18433100	-0.06117700	1.31449100
F	-1.18752900	1.17001900	-0.59374300
F	-1.18619100	-1.10876300	-0.70009800
K	-3.45729100	-0.00029600	-0.00195400

TMSOTf

Imaginary frequency: none

Si	1.95498000	0.15403200	-0.00302600
C	3.26959400	-0.86918600	-0.80595200
H	3.20201300	-1.91299000	-0.48955600
H	3.18688300	-0.82760000	-1.89488900
H	4.25645700	-0.49002800	-0.52458700
C	1.91086600	0.00454200	1.84341400

H	1.07638700	0.55402900	2.28444300
H	1.85089600	-1.03828600	2.16230100
H	2.83929300	0.42873900	2.23947100
C	1.89111000	1.90070600	-0.61796200
H	1.73826000	1.92338300	-1.70020600
H	1.09234800	2.47212900	-0.13992300
H	2.84003700	2.40024800	-0.40031500
O	0.46872400	-0.56292900	-0.64138300
S	-0.91133600	-0.97238500	-0.08964500
O	-1.62303600	-1.70901700	-1.09832600
O	-0.82954600	-1.46221700	1.26224500
C	-1.70844000	0.69130800	0.01270400
F	-2.96468100	0.55014300	0.39394200
F	-1.66479000	1.28687900	-1.16561300
F	-1.05706700	1.42977500	0.90127400

Int-a

Imaginary frequency: none

C	-4.91735000	-1.45170000	0.11231400
C	-3.58632500	-1.17018500	-0.17692100
C	-3.15777600	0.14635200	-0.08856500
C	-4.00975800	1.18399200	0.28299500
C	-5.32919300	0.87693700	0.56775500
C	-5.80160100	-0.44012500	0.48643300
H	-5.27048700	-2.47426400	0.04396900
H	-2.89966800	-1.95571000	-0.46784800
H	-3.64666600	2.20267700	0.33973800
H	-6.00908000	1.67006800	0.85919200
C	-7.24195200	-0.74107400	0.79193100
H	-7.43370200	-1.81395100	0.77434600

H	-7.51510000	-0.35153500	1.77534500
H	-7.89401200	-0.26070400	0.05759500
S	-1.47181500	0.48178600	-0.45735400
O	-0.77036500	-0.70467000	-0.90848000
N	-1.39974200	1.81460500	-1.13350100
C	-0.13602800	2.27446600	-1.74241500
H	-0.41983700	2.87226200	-2.61078300
H	0.45934100	1.42778100	-2.09595300
C	0.68757500	3.12684600	-0.78387700
H	0.09828000	3.99718100	-0.48129700
H	0.89682400	2.54506600	0.11815000
C	1.99351600	3.56326600	-1.43982300
H	1.79998900	4.09380800	-2.37653200
H	2.56224600	4.23077800	-0.78952800
H	2.62168000	2.69659300	-1.66778000
F	-0.91853300	0.61565200	1.10951700
Si	2.77124000	0.59360100	1.66022300
C	4.00590000	1.89770900	1.20379700
H	4.66539800	1.55766400	0.40123600
H	3.50771100	2.81651900	0.88463200
H	4.62513800	2.13465300	2.07459200
C	3.54661400	-0.96920400	2.29932700
H	2.80960900	-1.76485700	2.43730300
H	4.34884700	-1.33416000	1.65601300
H	3.97597200	-0.75044000	3.28276500
C	1.43425600	1.20014500	2.78567400
H	0.91248500	2.07274100	2.38934200
H	0.70176300	0.41712300	2.98976500
H	1.90477400	1.48570300	3.73285000
O	1.94393400	0.19207500	0.14170000

S	2.43124700	-0.77179800	-0.97013200
O	1.86441400	-0.40051000	-2.23713300
O	3.85713500	-0.98440700	-0.86517600
C	1.62369900	-2.38480200	-0.42603900
F	2.58854900	-3.25907500	-0.17645000
F	0.86238600	-2.85102200	-1.39305600
F	0.91404500	-2.21479100	0.67346000

Ts-a

Imaginary frequency: -202.12

C	-4.36709000	-1.39641500	-0.62320100
C	-3.05045400	-1.00575600	-0.80155300
C	-2.71638900	0.30881300	-0.48847200
C	-3.63340100	1.23078300	0.00490300
C	-4.94244800	0.80742900	0.17268000
C	-5.32730600	-0.50119800	-0.13854800
H	-4.65426700	-2.41440100	-0.85975200
H	-2.30597700	-1.69992400	-1.17251200
H	-3.33264400	2.24247800	0.24367400
H	-5.67907900	1.50590800	0.55260200
C	-6.75629100	-0.93324400	0.01751800
H	-7.29507100	-0.77297400	-0.92138100
H	-6.82014200	-1.99466100	0.26026900
H	-7.25902300	-0.35608600	0.79418200
S	-1.04976300	0.77342300	-0.73283400
O	-0.34474200	-0.05242200	-1.68055300
N	-0.82866000	2.19559000	-0.39481600
C	0.37150400	2.97566700	-0.75569800
H	-0.00757000	3.82939600	-1.32321800
H	1.04416500	2.39473900	-1.38908100

C	1.08731000	3.45723400	0.49800600
H	0.38035600	4.00774600	1.12487700
H	1.42661700	2.58658400	1.06130800
C	2.27345200	4.34053500	0.12438600
H	1.94768500	5.21953600	-0.43805300
H	2.79550800	4.68619400	1.01844400
H	2.98903200	3.79028800	-0.49250800
F	-0.54338200	-0.31735000	0.89049300
Si	0.92469000	-0.71460900	2.01267900
C	0.80299500	0.78766300	3.10568800
H	1.71181200	1.39128800	3.05175100
H	-0.05312700	1.41715900	2.85176000
H	0.68170100	0.45662400	4.14244600
C	2.59658100	-1.54469300	2.30818400
H	2.78087900	-2.39155100	1.63869400
H	3.44150100	-0.86057900	2.22849000
H	2.56611700	-1.94824400	3.32589400
C	-0.19751800	-2.13421200	2.57216000
H	-1.22174000	-1.80715100	2.76249600
H	-0.23336700	-2.91662100	1.80670400
H	0.20773800	-2.58478300	3.48288900
O	1.68463800	0.19989100	0.41258000
S	2.72763300	-0.11507600	-0.63773200
O	2.62690700	0.80293900	-1.75235900
O	4.04649600	-0.36224200	-0.09474000
C	2.13786000	-1.78034400	-1.28157800
F	3.13044000	-2.65740000	-1.21517900
F	1.74128500	-1.68445300	-2.53618300
F	1.12929300	-2.23613400	-0.53924100

Int-b

Imaginary frequency: none

C	-4.27959000	-0.47220200	-1.44037200
C	-2.94314500	-0.15499400	-1.60035000
C	-2.41257100	0.83569500	-0.77441500
C	-3.15694500	1.51134300	0.19221900
C	-4.48887900	1.16192300	0.32829000
C	-5.06551500	0.16977700	-0.47501300
H	-4.72109900	-1.23588400	-2.06982000
H	-2.33029800	-0.66140000	-2.33491100
H	-2.70893400	2.28000000	0.80846300
H	-5.09474900	1.67078600	1.06873800
C	-6.49863100	-0.22480100	-0.28252500
H	-6.55307900	-1.06654400	0.41539400
H	-7.08036800	0.59648500	0.13646400
H	-6.94691100	-0.54456700	-1.22385000
S	-0.72809600	1.21851900	-0.99109600
O	-0.10609800	0.63248400	-2.14711500
N	-0.27134900	2.27031500	-0.05936300
C	1.04315500	2.93071100	-0.05255800
H	0.86883200	3.90514800	-0.52194900
H	1.77087700	2.37042400	-0.64047400
C	1.51276900	3.11120700	1.38282800
H	0.74079500	3.64268100	1.94574500
H	1.63928200	2.12509300	1.83295500
C	2.82813000	3.88254000	1.40872600
H	2.71051200	4.87377900	0.96271200
H	3.17607400	4.01337600	2.43501600
H	3.59669800	3.34018300	0.85345500
F	-0.75806900	-1.35849700	-0.18636900

Si	-0.87240500	-1.74612800	1.41977500
C	-0.80940100	-0.18530600	2.42888200
H	0.11143200	0.36963900	2.24480800
H	-1.66897300	0.46472400	2.24510500
H	-0.83740500	-0.46295500	3.48842400
C	0.51957200	-2.91165800	1.79936900
H	0.51148900	-3.76744700	1.11849900
H	1.48503600	-2.40634900	1.71820600
H	0.41599100	-3.29473300	2.81942400
C	-2.55224800	-2.53639600	1.56587700
H	-3.33139700	-1.82585900	1.26875700
H	-2.63920700	-3.42447600	0.93382600
H	-2.74935600	-2.83554900	2.59970400
O	1.52609300	0.03462900	0.35278100
S	2.97028000	-0.20498300	0.25205400
O	3.75393700	0.96339000	-0.14685800
O	3.53386700	-1.00660000	1.33657700
C	3.08998500	-1.31875000	-1.21222400
F	4.35521600	-1.68176500	-1.42600300
F	2.64398000	-0.71019300	-2.30797800
F	2.36891900	-2.42457800	-1.02596300

Int-1

Imaginary frequency: none

Si	1.35863200	1.99849600	-0.64157300
C	0.22051900	2.03606700	-2.09824300
H	0.72963800	1.70166500	-3.00628000
H	-0.66004600	1.41268200	-1.92378000
H	-0.12442100	3.06091800	-2.26618100
C	2.97006000	2.87651700	-0.88901700

H	3.64031600	2.73870000	-0.03662600
H	3.47988600	2.54273100	-1.79562900
H	2.76798300	3.94810600	-0.98571200
C	0.54811800	2.38349300	0.97602500
H	-0.27602200	1.69454800	1.16892600
H	1.25147700	2.35066000	1.81044300
H	0.13976500	3.39822000	0.92428100
O	1.75504200	0.25473000	-0.58732300
S	2.95119200	-0.63088300	-0.17416800
O	2.61867000	-1.97896400	-0.58146500
O	4.22279100	-0.05243800	-0.50447400
C	2.78488200	-0.58064000	1.66849500
F	3.61577000	-1.45625700	2.19644800
F	1.54320500	-0.87465000	2.00365700
F	3.08572200	0.63599400	2.08991300
C	-4.90565900	0.42273400	2.13252400
C	-3.73698800	-0.03661100	1.53110000
C	-3.65188500	-0.21253000	0.14428900
C	-4.78046500	0.09469800	-0.62282600
C	-5.95503200	0.55554900	-0.03127800
C	-6.01959200	0.71945400	1.34983500
H	-4.94955400	0.55259000	3.20845400
H	-2.87343200	-0.25975100	2.15148600
H	-4.73926700	-0.02458200	-1.70121500
H	-6.81809600	0.78870400	-0.64545300
H	-6.93120000	1.07897800	1.81406000
B	-2.31318300	-0.76246700	-0.52385300
F	-2.14601500	-2.18072000	-0.40370400
F	-2.22060000	-0.49082700	-1.92564200
F	-1.12292100	-0.20500300	0.05312800

K	0.11982800	-1.75013000	-1.68591100

Ts-1

Imaginary frequency: -161.26

Si	-0.67063700	2.16188400	0.03026000
C	-0.27239400	2.67555500	1.76405800
H	-0.88044500	2.15280400	2.50413700
H	0.78243400	2.53365300	2.00051200
H	-0.49746300	3.74540900	1.83717200
C	-2.39548200	2.66293200	-0.49934200
H	-2.68931700	2.23167100	-1.45969100
H	-3.15780100	2.41405400	0.23845100
H	-2.36623000	3.75007100	-0.63114700
C	0.39977300	3.04555200	-1.23266900
H	1.45919100	3.02284600	-0.96988900
H	0.28549400	2.59505500	-2.22358600
H	0.07292500	4.08733000	-1.30493000
O	-1.54139100	0.32281000	0.65525000
S	-2.57114600	-0.61456100	0.08067600
O	-2.51860200	-1.88301700	0.79988000
O	-3.87136500	-0.02432700	-0.14030200
C	-1.91529800	-0.97394600	-1.62519700
F	-2.92270100	-1.29773400	-2.42016600
F	-1.04670200	-1.96570300	-1.60505600
F	-1.32079000	0.11493500	-2.11117200
C	4.67341700	-1.45980900	-1.22015200
C	3.36618500	-1.43026600	-0.73863500
C	2.90424100	-0.35826800	0.03031600
C	3.79225100	0.68889900	0.30544900
C	5.09897700	0.66853700	-0.17272200

C	5.54145500	-0.40884200	-0.93805800
H	5.01499800	-2.30061100	-1.81371200
H	2.69606200	-2.25474700	-0.96124900
H	3.45851900	1.53132600	0.90517900
H	5.77312400	1.48809200	0.05031800
H	6.55935400	-0.42888500	-1.31100100
B	1.41643000	-0.31482100	0.57432000
F	0.69182100	-1.50358000	0.42627300
F	1.27833200	0.11464300	1.90713800
F	0.64666900	0.72331800	-0.23698800
K	-0.66912100	-1.42558200	2.65032700

Int-2

Imaginary frequency: none

Si	0.90808400	1.68616000	0.45305700
C	1.18702100	1.34636000	2.25523800
H	0.33086000	0.82443200	2.69198200
H	2.08415100	0.74314300	2.41640900
H	1.31451900	2.28924800	2.79601000
C	-0.65468000	2.62633900	0.12639100
H	-0.87920000	2.67454900	-0.94229100
H	-1.49602900	2.16233900	0.64525500
H	-0.54713100	3.65174700	0.49393800
C	2.37981500	2.42436100	-0.40302600
H	3.31411200	1.94195700	-0.10582600
H	2.27958400	2.35540300	-1.48990600
H	2.44923600	3.48536500	-0.14222100
O	-2.46553200	-0.01336700	1.28427600
S	-3.69171200	-0.43358100	0.58641300
O	-3.57176900	-1.79468500	0.04153800

O	-4.94956200	-0.09856300	1.22719200
C	-3.64154900	0.59467300	-0.94286600
F	-4.62088000	0.26194500	-1.77500500
F	-2.47350600	0.38990500	-1.56481400
F	-3.74607400	1.88803100	-0.66211800
C	5.22765300	0.16340700	-1.58002200
C	4.06015000	-0.57838000	-1.44871600
C	3.58469600	-0.93243700	-0.17949300
C	4.30401200	-0.53875800	0.95645600
C	5.47470600	0.19900700	0.82619200
C	5.93233900	0.55174300	-0.44205200
H	5.58935100	0.44021800	-2.56327300
H	3.50825900	-0.87814400	-2.33341800
H	3.94501400	-0.81222700	1.94305000
H	6.02942900	0.50004800	1.70704800
H	6.84348300	1.13040400	-0.54404100
B	2.26690200	-1.70209700	-0.03747300
F	1.59631800	-2.20917700	-1.07939400
F	1.69273200	-1.95595200	1.14583600
F	0.67474600	0.16947800	-0.22938100
K	-0.89926400	-1.86578800	0.10213000

Int-3

Imaginary frequency: none

C	-2.50684800	-1.43052000	1.02352000
C	-1.17746100	-1.13649900	1.27002100
C	-0.24021500	-1.44538200	0.28523900
C	-0.59221300	-2.04660200	-0.91452300
C	-1.93487800	-2.33221700	-1.13627400
C	-2.90409000	-2.02645300	-0.18071400

H	-3.25386300	-1.18741800	1.77129500
H	-0.87648300	-0.66794600	2.19995900
H	0.16203600	-2.27648000	-1.65583400
H	-2.23067300	-2.79735800	-2.06982700
C	-4.35985000	-2.28296300	-0.44308600
H	-4.49975500	-2.92956700	-1.30956200
H	-4.83721900	-2.74305200	0.42446600
H	-4.87302700	-1.33503800	-0.63536300
S	1.44830600	-1.11683900	0.64179100
O	1.87590000	-1.65576400	1.91925100
N	2.23771100	-1.20981100	-0.62144100
C	3.71266000	-1.20100900	-0.58376500
H	4.03798000	-1.65387000	-1.52127600
H	4.08417100	-1.82366500	0.23686900
C	4.27714600	0.21150200	-0.46679700
H	3.86019000	0.82377900	-1.27078600
H	3.94976800	0.65505100	0.47914400
C	5.80040000	0.20167000	-0.54043300
H	6.14126800	-0.21729100	-1.49095900
H	6.20267800	1.21285400	-0.45493600
H	6.22766400	-0.40031900	0.26601500
C	-1.69016700	1.38511500	-1.28662000
C	-3.05918800	1.14598800	-1.24853000
C	-3.80690100	1.57359000	-0.15307600
C	-3.18723700	2.23653100	0.90429600
C	-1.81911200	2.48153000	0.85947500
C	-1.05587700	2.06050900	-0.23632000
H	-1.10767700	1.05032500	-2.13902600
H	-3.54371300	0.62338100	-2.06587000
H	-4.87495900	1.38742500	-0.12242700

H	-3.77148400	2.56453100	1.75626100
H	-1.33758300	3.00409900	1.67951800
B	0.45864700	2.35460300	-0.29752700
F	1.24147200	0.50888200	1.07179500
F	1.21554600	1.92295200	-1.30487900
F	1.06146600	3.14072500	0.59118600

Ts-2

Imaginary frequency: -320.49

C	-2.49422800	-1.57743500	0.93750700
C	-1.18375200	-1.30261400	1.28792900
C	-0.20189000	-1.45144100	0.31070200
C	-0.48392600	-1.85611900	-0.98818200
C	-1.80608200	-2.13190500	-1.30698200
C	-2.82318600	-1.98980600	-0.35954500
H	-3.27879700	-1.45775700	1.67646300
H	-0.93049500	-0.97531700	2.28933500
H	0.30584200	-1.94905100	-1.72228100
H	-2.05321400	-2.44621500	-2.31459000
C	-4.25443700	-2.26757700	-0.71754500
H	-4.39732300	-2.27095400	-1.79847000
H	-4.55663900	-3.24534400	-0.33067900
H	-4.91420300	-1.51991000	-0.27214300
S	1.43303300	-1.04255600	0.76926300
O	1.68907900	-1.17976500	2.18441500
N	2.37280500	-1.33417800	-0.33945700
C	3.83209800	-1.16137900	-0.21125600
H	4.27598900	-1.85497500	-0.92606700
H	4.17234500	-1.43630000	0.79136500
C	4.24315800	0.27044900	-0.54295600

H	3.85270700	0.52741700	-1.53111200
H	3.78147400	0.95375500	0.17607400
C	5.76044900	0.41940300	-0.51319500
H	6.23160800	-0.24500300	-1.24218400
H	6.05416600	1.44336600	-0.75096300
H	6.15885400	0.17467100	0.47488700
C	-1.97733900	1.35357400	-1.16889400
C	-3.34655700	1.34070200	-0.91043500
C	-3.82578600	1.80499400	0.31074600
C	-2.93527400	2.29029900	1.26842100
C	-1.57122000	2.31132900	0.99939100
C	-1.07173100	1.83632900	-0.22016800
H	-1.60896600	0.98096700	-2.11940200
H	-4.03660700	0.96190400	-1.65682100
H	-4.89018600	1.79057000	0.51775000
H	-3.30760200	2.65355300	2.21971700
H	-0.88342600	2.69257700	1.74825100
B	0.46565700	1.85926800	-0.53042500
F	1.15120600	0.78063800	0.80391000
F	0.92237100	1.16824800	-1.60283400
F	1.20238000	2.94568800	-0.20525000

Int-4

Imaginary frequency: none

C	-2.37615900	-1.65189600	0.87766200
C	-1.05731700	-1.47070400	1.25607700
C	-0.08429400	-1.55855200	0.26103800
C	-0.37318400	-1.80735800	-1.08243600
C	-1.69935400	-1.98933500	-1.42041200
C	-2.71462100	-1.90615000	-0.45546200

H	-3.15715500	-1.57598200	1.62524800
H	-0.79316500	-1.25676200	2.28439200
H	0.41356800	-1.84972200	-1.82378800
H	-1.96050700	-2.18056400	-2.45480600
C	-4.14769500	-2.09546300	-0.85110200
H	-4.32405500	-1.73007300	-1.86376000
H	-4.39601700	-3.16158600	-0.83415500
H	-4.81648600	-1.58289300	-0.15867500
S	1.54589400	-1.26137100	0.74536300
O	1.77310300	-1.20853300	2.16586800
N	2.50691300	-1.28618900	-0.37385300
C	3.94957100	-1.00985800	-0.26898700
H	4.42278200	-1.64459600	-1.01831500
H	4.33018600	-1.27114200	0.72063700
C	4.20064300	0.46607400	-0.58000300
H	3.77016500	0.69846500	-1.55646400
H	3.68296200	1.07837800	0.16294400
C	5.69687100	0.75834400	-0.56739200
H	6.21807000	0.16054100	-1.31907700
H	5.88018200	1.81169800	-0.78582800
H	6.13234100	0.53401700	0.40943200
C	-2.03305100	1.45069400	-1.12115400
C	-3.41711400	1.42166200	-0.95134700
C	-3.96786900	1.65280300	0.30637700
C	-3.12770400	1.91749700	1.38751600
C	-1.74784000	1.94435000	1.20412300
C	-1.17000000	1.70823800	-0.05109100
H	-1.61433500	1.25456100	-2.10401800
H	-4.06524900	1.21416000	-1.79660400
H	-5.04338900	1.62831500	0.44439200

H	-3.55070600	2.09850400	2.36998700
H	-1.10396000	2.13914600	2.05765400
B	0.42341400	1.74780300	-0.24874200
F	1.08273900	1.10105300	0.88261900
F	0.83860900	1.04159700	-1.40064300
F	0.94586100	3.05379100	-0.30550000

3a

Imaginary frequency: none

C	-3.66290600	0.12044500	0.73057100
C	-2.33828700	0.17052900	1.15228000
C	-1.36441900	-0.43485300	0.36988700
C	-1.68107900	-1.08036300	-0.82222000
C	-3.00804100	-1.12094500	-1.22298800
C	-4.01516200	-0.52135100	-0.45729700
H	-4.43325400	0.58417100	1.33666200
H	-2.06844000	0.66202400	2.07967200
H	-0.90306700	-1.55124600	-1.40961000
H	-3.27112900	-1.62750600	-2.14566500
C	-5.44542400	-0.57474700	-0.91851700
H	-5.55783900	-0.06012000	-1.87632200
H	-5.76365300	-1.60977200	-1.06504100
H	-6.11153400	-0.10550800	-0.19419400
S	0.32466800	-0.31753100	0.92090300
O	0.32322900	-0.04866900	2.37275100
N	1.01082200	-1.54280300	0.30269500
C	2.47696500	-1.61181300	0.42037400
H	2.76697100	-1.96354700	1.41803800
H	2.94900100	-0.62878600	0.27679200
C	3.01366100	-2.57603300	-0.62827100

H	2.52288500	-3.54518400	-0.49639000
H	2.73289500	-2.20633100	-1.61956500
C	4.52754200	-2.73342300	-0.53170000
H	4.81866600	-3.11812500	0.44965100
H	4.90173100	-3.42700600	-1.28755300
H	5.03135200	-1.77387300	-0.67818500
C	0.91803400	1.20708500	0.15784600
C	1.03075700	2.36148300	0.92178900
C	1.24067500	1.18479900	-1.19588200
C	1.47698300	3.52851400	0.30750100
H	0.77986900	2.34036600	1.97509600
C	1.68511400	2.35632700	-1.79658900
H	1.15147500	0.26585000	-1.76536300
C	1.80113900	3.52488600	-1.04578600
H	1.57206400	4.43819300	0.88821200
H	1.94368100	2.35684500	-2.84861300
H	2.14873900	4.43577400	-1.51893800

BF_3

Imaginary frequency: none

B	0.00000000	0.00000000	0.00000000
F	0.00000000	1.31394800	0.00000000
F	1.13791300	-0.65697400	0.00000000
F	-1.13791300	-0.65697400	0.00000000

Int-c

Imaginary frequency: none

C	3.44070400	0.24135400	-0.83307600
C	2.06707100	0.21786300	-1.04391700
C	1.29130200	-0.57862300	-0.21250500

C	1.83461000	-1.33882100	0.82074500
C	3.20509000	-1.29565700	1.00794200
C	4.02538700	-0.50927700	0.18735500
H	4.06549100	0.85220700	-1.47440400
H	1.61341400	0.79983200	-1.83667900
H	1.19981300	-1.94932500	1.45033500
H	3.65162700	-1.88109100	1.80410900
C	5.51072800	-0.48922400	0.41133600
H	5.73849900	-0.18935000	1.43702800
H	5.93020700	-1.48755200	0.26236900
H	6.00406000	0.20057700	-0.27321300
S	-0.43883300	-0.58852800	-0.49254300
O	-0.81422800	0.12795800	-1.69335900
N	-1.00372400	-1.89590200	-0.05311000
C	-2.41775500	-2.23487300	-0.31206000
H	-2.51698800	-3.29089200	-0.05953900
H	-2.64235600	-2.12005200	-1.37715400
C	-3.39224300	-1.40578200	0.51969500
H	-3.09755200	-1.46417500	1.57122900
H	-3.32213100	-0.35481900	0.22085100
C	-4.82265700	-1.90196400	0.33754500
H	-4.91846800	-2.94154400	0.66156400
H	-5.52137400	-1.30054900	0.92197500
H	-5.12501600	-1.84565400	-0.71156100
F	-0.85131000	0.59884700	0.69755200
B	-1.15793700	2.72259600	0.24875400
F	-0.02913100	2.81828800	-0.43007500
F	-2.29394200	2.51932000	-0.38997900
F	-1.17692100	3.04060400	1.52920100

Ts-b

Imaginary frequency: -207.88

C	-3.42824800	0.16089400	0.86379600
C	-2.05743400	0.14842200	1.09034100
C	-1.26193600	-0.59888200	0.23197100
C	-1.77990400	-1.31890300	-0.84245000
C	-3.14834000	-1.28623800	-1.04381300
C	-3.98921400	-0.54928000	-0.19843700
H	-4.06973200	0.73113800	1.52556600
H	-1.62171900	0.70097300	1.91350100
H	-1.12879300	-1.89033000	-1.49170200
H	-3.57700600	-1.84099300	-1.87103300
C	-5.46980600	-0.52573100	-0.44975600
H	-5.86137100	-1.54236000	-0.52900600
H	-5.99794100	-0.00585800	0.34933400
H	-5.68381900	-0.01839600	-1.39435300
S	0.46223700	-0.59794200	0.53010600
O	0.83131300	0.10371000	1.73909500
N	1.06511500	-1.86109800	0.02789500
C	2.48822600	-2.18014700	0.26362500
H	2.60971400	-3.21813800	-0.04624900
H	2.71353300	-2.11522900	1.33237800
C	3.43398500	-1.28152600	-0.52825400
H	3.13612000	-1.29469700	-1.58025900
H	3.33561500	-0.24985200	-0.17543400
C	4.87800800	-1.74731300	-0.37588500
H	5.00067500	-2.76591800	-0.75288900
H	5.55660800	-1.09763900	-0.93153800
H	5.18350800	-1.73575900	0.67366500
F	0.86058100	0.67149700	-0.65924200

B	1.02781900	2.60673500	-0.26322200
F	-0.09047300	2.70803100	0.44813000
F	2.19090600	2.53871300	0.37212800
F	1.01358600	2.98694700	-1.53461800

Int-d

Imaginary frequency: none

C	-3.10543500	-0.51816900	0.94682100
C	-1.76453100	-0.51947600	1.29195600
C	-0.85430000	-0.93149100	0.32101300
C	-1.22084900	-1.32145300	-0.96969600
C	-2.56520800	-1.30568200	-1.27636200
C	-3.52201000	-0.90346100	-0.33071500
H	-3.84017300	-0.20850700	1.68011800
H	-1.43723700	-0.20838100	2.27535300
H	-0.47614100	-1.61718500	-1.69694500
H	-2.88644100	-1.60112300	-2.26845500
C	-4.97179800	-0.87074700	-0.70859700
H	-5.14864400	-0.05636200	-1.41729300
H	-5.26083600	-1.80094500	-1.20216400
H	-5.60590200	-0.71384500	0.16326500
S	0.81747400	-0.87642700	0.74296200
O	1.12081400	-0.31860700	2.03370600
N	1.69557600	-1.55119100	-0.23399800
C	3.16733500	-1.60249100	-0.16156400
H	3.45755200	-2.43301900	-0.80345500
H	3.48667800	-1.80768700	0.86272200
C	3.77319500	-0.29197700	-0.66466700
H	3.37077200	-0.07394300	-1.65655100
H	3.47137500	0.52138700	0.00047000

C	5.29306100	-0.40260400	-0.71171300
H	5.60580600	-1.20029000	-1.38972400
H	5.73131200	0.53279300	-1.06341100
H	5.70071500	-0.61691300	0.27924000
F	0.92223400	1.31073100	-0.40162700
B	-0.15358000	2.23399400	-0.16230800
F	-0.48029500	2.16318400	1.19358000
F	0.27900100	3.51078100	-0.50116200
F	-1.24236600	1.85412500	-0.94518600

Int-5

Imaginary frequency: none

C	3.87897200	0.40205000	-1.05790500
C	2.49799800	0.57630700	-1.09515800
C	1.69760100	0.20244700	-0.01143300
C	2.31593100	-0.35039200	1.11572100
C	3.69529000	-0.52779200	1.15947500
C	4.47837200	-0.15160000	0.07033600
H	4.48709900	0.69722100	-1.90559600
H	2.03760600	1.01173100	-1.97641800
H	1.71161900	-0.64304100	1.96897600
H	4.16072600	-0.95719500	2.03953200
H	5.55347700	-0.28848000	0.10205900
B	0.13620700	0.38701500	-0.05301700
F	-0.48066200	-1.02686000	-0.49692500
F	-0.50812000	0.61744600	1.15948600
F	-0.38023900	1.24826500	-1.01317000
K	-2.48706200	2.10781600	0.30570500
B	-1.91458000	-1.49649700	-0.17284000
F	-1.87156800	-1.98314700	1.09723600

F	-2.19759700	-2.42021400	-1.13021500
F	-2.69397800	-0.34964900	-0.28239900

Ts-3

Imaginary frequency: -61.61

C	-3.72145700	-0.63043100	-0.99068200
C	-2.37894600	-0.98725800	-0.91152200
C	-1.56391700	-0.48566000	0.10898300
C	-2.12506300	0.37982900	1.05423800
C	-3.46735500	0.73775700	0.98066700
C	-4.26578500	0.23306400	-0.04331000
H	-4.34302000	-1.02486100	-1.78643300
H	-1.96019000	-1.66540700	-1.64827300
H	-1.50505300	0.77872800	1.84986900
H	-3.89199000	1.40851400	1.71886700
H	-5.31199000	0.51160700	-0.10189400
B	-0.06219000	-0.89611400	0.19017700
F	0.81795800	0.25679000	-0.80829500
F	0.64075200	-0.70923300	1.35378800
F	0.37706800	-2.01331100	-0.47349800
K	2.94591500	-1.39296900	0.15549900
B	1.41392900	1.54656800	-0.29375600
F	1.59331100	2.34397600	-1.38715800
F	0.54535900	2.05210700	0.63194600
F	2.62736400	1.14986700	0.27666600

Int-6

Imaginary frequency: none

C	-3.79760300	-0.47118400	-1.03014000
C	-2.52364200	-1.00124500	-0.86479100

C	-1.69857200	-0.55374300	0.17477400
C	-2.17241700	0.43374400	1.04800800
C	-3.44597000	0.96391500	0.88421700
C	-4.25675900	0.51038200	-0.15472400
H	-4.43219200	-0.82008000	-1.83627500
H	-2.16567300	-1.76738300	-1.54436800
H	-1.53618900	0.79045300	1.85013200
H	-3.80846200	1.72853500	1.56122600
H	-5.25075700	0.92416600	-0.28240200
B	-0.29999400	-1.15188000	0.36459900
F	1.14547400	0.29326700	-0.94376900
F	0.50393100	-0.83712800	1.39007600
F	0.19499400	-2.12209200	-0.41843700
K	2.84072100	-1.42421900	0.05365000
B	1.59681400	1.48675100	-0.28127200
F	1.97344000	2.41766700	-1.23049400
F	0.58823100	1.95977300	0.53846500
F	2.71673700	1.09283300	0.49434100

TMSBr

Imaginary frequency: none

Si	-0.85311800	0.00000600	-0.00012700
C	-1.37897700	-0.81429400	-1.58899300
H	-1.01320300	-0.26054000	-2.45693900
H	-1.00825300	-1.84042700	-1.64893400
H	-2.47238800	-0.84247300	-1.63742000
C	-1.38058500	1.78284200	0.08931900
H	-1.01140300	2.26006600	1.00031900
H	-1.01593800	2.34726200	-0.77211500
H	-2.47431200	1.83677300	0.09452500

C	-1.38004200	-0.96929700	1.49918200
H	-1.01439100	-1.99797500	1.45357200
H	-1.01031200	-0.50860100	2.41847000
H	-2.47359600	-0.99709500	1.54665500
Br	1.43643000	0.00021200	0.00018900

Int$_{Br}$-1

Imaginary frequency: none

Si	-2.10897200	-1.59626700	0.22345900
C	-0.73382100	-1.76495700	-1.01390900
H	-0.09391000	-0.88248200	-1.01069500
H	-0.12352200	-2.63737300	-0.75738500
H	-1.13464900	-1.90974800	-2.02045600
C	-3.47204000	-2.83312500	-0.04504500
H	-4.30205700	-2.66620000	0.64555100
H	-3.85220800	-2.79002700	-1.06842000
H	-3.07908500	-3.84011200	0.13060500
C	-1.55632200	-1.53291900	1.99548800
H	-0.81090400	-0.75221700	2.15092200
H	-2.40427100	-1.36085200	2.66290700
H	-1.10647200	-2.49640300	2.25822500
C	4.95336700	-0.64677400	0.92886000
C	3.85587700	0.21041600	0.91678200
C	2.86633400	0.11252800	-0.06862800
C	3.01683900	-0.87461100	-1.04930500
C	4.11067400	-1.73776900	-1.04621900
C	5.08190500	-1.62513500	-0.05456800
H	5.70938100	-0.55194500	1.70078600
H	3.77037700	0.97429400	1.68426200
H	2.26936600	-0.96614700	-1.83192100

H	4.20848200	-2.49467100	-1.81690200
H	5.93571700	-2.29357600	-0.04958400
B	1.59732000	1.07541200	-0.05130600
F	1.87450200	2.39928200	0.40294200
F	0.97940400	1.22416700	-1.33497200
F	0.54083100	0.60858800	0.81070800
K	-0.74146200	2.72425100	-0.02183500
Br	-3.10425600	0.45551800	-0.21210700

TS-Br

Imaginary frequency: -164.03

Si	0.69889400	-1.73584000	-0.16190100
C	0.32217700	-1.71616900	1.65766800
H	0.12841300	-0.70131500	2.00816600
H	-0.55991800	-2.33187700	1.85996700
H	1.17045400	-2.11419000	2.21854600
C	1.96067500	-3.00947500	-0.72010900
H	2.33122000	-2.77476700	-1.72157600
H	2.81407900	-3.07697800	-0.04662500
H	1.44994700	-3.97780700	-0.77146000
C	-0.78145000	-2.19260000	-1.22234600
H	-1.71375300	-1.76216400	-0.85465500
H	-0.64211600	-1.86889300	-2.25853300
H	-0.87654800	-3.28286800	-1.22850100
C	-4.40319900	0.07557000	-0.99993400
C	-3.12784000	0.62954000	-1.05641300
C	-2.28722700	0.62379100	0.06270000
C	-2.76905100	0.06293900	1.24955100
C	-4.04394800	-0.49508600	1.31444200
C	-4.86088800	-0.49365300	0.18678400

H	-5.04052700	0.08574400	-1.87720200
H	-2.77680700	1.06793200	-1.98586600
H	-2.13983400	0.05926400	2.13404700
H	-4.40125100	-0.92824400	2.24210900
H	-5.85321700	-0.92806900	0.23368800
B	-0.81843200	1.19813400	-0.03968600
F	-0.64445200	2.25271500	-0.95153800
F	-0.22114300	1.56411600	1.17586100
F	0.15351600	0.14971700	-0.60660000
K	1.80007200	2.66627100	-0.11537800
Br	2.93055200	-0.18348300	0.15175800

Int$_{Br}$-2

Imaginary frequency: none

Si	-0.04096300	1.73457900	-0.40460200
C	-0.33490900	1.72320400	1.42518600
H	-1.30765500	1.27157800	1.64157300
H	0.44467400	1.17281600	1.95816600
H	-0.34150600	2.74964400	1.80565400
C	-1.38596600	2.57506500	-1.36376700
H	-1.22967400	2.47015000	-2.44103900
H	-2.35933700	2.15182400	-1.09988400
H	-1.40034500	3.64394000	-1.12895000
C	1.65385900	2.32512400	-0.88324400
H	2.43291200	1.89467700	-0.24880500
H	1.87735600	2.08268700	-1.92603900
H	1.69744200	3.41403800	-0.77818100
C	4.60185600	-0.13777500	-0.73817400
C	3.40814300	-0.83944600	-0.85488100
C	2.54651200	-0.95926800	0.24298300

C	2.90529100	-0.37157900	1.46278600
C	4.10103100	0.32706200	1.58219100
C	4.94560500	0.44544100	0.48007400
H	5.26387000	-0.04342000	-1.59090000
H	3.13757100	-1.29118600	-1.80352900
H	2.24529800	-0.46278800	2.31903200
H	4.37534300	0.77937700	2.52794000
H	5.87675400	0.99319200	0.57156700
B	1.20394000	-1.68538900	0.09453400
F	0.85739500	-2.36606900	-1.00523600
F	0.28527700	-1.72885100	1.06705300
F	-0.11512000	0.12292600	-0.87475000
K	-1.86498800	-1.78918600	-0.76074800
Br	-3.84630400	0.07233800	0.54391000

TMSOMs

Imaginary frequency: none

Si	-1.46587500	0.02104400	0.01996100
C	-2.57111600	0.38334900	1.46424400
H	-2.50408300	-0.40703500	2.21628400
H	-2.30558400	1.33340200	1.93475100
H	-3.61128400	0.44769700	1.13143300
C	-1.79291300	-1.63739600	-0.74730700
H	-1.11147100	-1.82366400	-1.58006600
H	-1.67168700	-2.43851000	-0.01295900
H	-2.81919800	-1.67823600	-1.12470800
C	-1.43017100	1.39577700	-1.23104900
H	-1.18173700	2.35326700	-0.76508700
H	-0.70943800	1.19377900	-2.02767600
H	-2.41822400	1.49466400	-1.69133300

O	0.10653300	-0.04072800	0.76415900
S	1.51615300	-0.17900800	0.06990400
O	2.36446100	-0.94069800	0.96228600
O	1.32523600	-0.66779300	-1.28608400
C	2.07258100	1.49869600	0.01390600
H	3.06754900	1.49073400	-0.43116500
H	1.38095000	2.07278300	-0.60152400
H	2.10789000	1.88183300	1.03248800

Int_{Ms}-1

Imaginary frequency: none

Si	2.41773900	1.57053700	-0.36278100
C	4.23415600	1.55668600	-0.75092400
H	4.82230300	1.22111500	0.10573100
H	4.44984600	0.90042500	-1.59859000
H	4.55885600	2.56768500	-1.01619400
C	2.04415900	2.46321000	1.22098300
H	0.99520300	2.34583700	1.50030400
H	2.67802100	2.12063500	2.04368100
H	2.24303300	3.53018300	1.07721000
C	1.38261500	2.11103400	-1.80002800
H	1.63427300	1.54777800	-2.70347000
H	0.32127700	1.98201900	-1.58413800
H	1.57315500	3.16861800	-2.00705900
O	1.93475300	-0.10437100	-0.16985500
S	2.34471900	-1.16388100	0.92862500
O	2.00824100	-2.44905200	0.32749300
O	3.71597600	-0.93384200	1.33622100
C	1.25060900	-0.83901800	2.27537600
C	-5.42433800	0.04627800	-0.71680600

C	-4.05602300	-0.05366500	-0.96177900
C	-3.11565300	0.16098100	0.05173200
C	-3.59689900	0.48681700	1.32584900
C	-4.96173600	0.58805700	1.58326100
C	-5.88030900	0.36654100	0.55922600
H	-6.13449800	-0.12260500	-1.51916700
H	-3.71240400	-0.29782300	-1.96255300
H	-2.89070800	0.66996400	2.13096200
H	-5.31087800	0.84213600	2.57833300
H	-6.94399400	0.44626700	0.75443400
B	-1.54303400	0.01697500	-0.21323100
F	-1.05454800	-1.29146000	0.16519200
F	-1.21203100	0.13370800	-1.60428200
F	-0.77554500	0.95902000	0.50320600
K	0.51364700	-1.77085300	-1.80826300
H	1.47498300	0.14904000	2.67358000
H	1.44924800	-1.60777300	3.02282700
H	0.23219500	-0.90242900	1.89892400

TS-Ms

Imaginary frequency: -114.28

Si	-0.72564000	1.92189000	0.16395000
C	-1.45678600	2.12926000	1.87443400
H	-2.52929000	1.93621000	1.89315000
H	-0.96804200	1.45572900	2.58409200
H	-1.26130500	3.15067800	2.21472400
C	-1.64001800	2.60902600	-1.30069800
H	-1.09510400	2.39672900	-2.22528800
H	-2.66254400	2.24164000	-1.39597400
H	-1.66799500	3.69813500	-1.19095300

C	0.87082300	2.92211600	0.30258500
H	1.51351500	2.53355500	1.09960000
H	1.44715100	2.92135700	-0.62667800
H	0.62735700	3.95982500	0.55393000
O	-1.89018600	0.15811200	0.21098600
S	-2.74759500	-0.62966500	-0.77260000
O	-2.94559400	-1.96898300	-0.18763700
O	-3.96655100	0.08986700	-1.12644500
C	-1.78022200	-0.82990300	-2.24570900
C	4.89742400	0.20207400	0.53070300
C	3.54263300	0.12175800	0.84477100
C	2.63186000	-0.46589000	-0.03875100
C	3.11567500	-0.97415100	-1.25010000
C	4.46716800	-0.89558900	-1.57190200
C	5.36054400	-0.30587800	-0.67980500
H	5.59073000	0.65868900	1.22819500
H	3.18940900	0.51685800	1.79213400
H	2.42745500	-1.43902900	-1.95011800
H	4.82596200	-1.29349900	-2.51456600
H	6.41438800	-0.24474200	-0.92734700
B	1.09287100	-0.54338300	0.30607300
F	0.43460600	-1.72242800	-0.04862100
F	0.73666300	-0.20486600	1.61905600
F	0.35415500	0.54374500	-0.53215000
K	-1.35526500	-1.83397200	1.90974100
H	-1.57332900	0.15140500	-2.66984400
H	-2.37739300	-1.42154900	-2.93940600
H	-0.85960100	-1.34839800	-1.98775000

Int$_{Ms}$-1

Imaginary frequency: none

Si	0.01871600	2.02945700	-0.08579000
C	0.82185300	2.42629400	-1.71495300
H	1.87314900	2.12770400	-1.71980700
H	0.29430900	1.95985000	-2.55367200
H	0.78469700	3.50734500	-1.88296000
C	0.95867600	2.65154500	1.38327400
H	0.55441000	2.25083500	2.31675900
H	2.01258400	2.38100700	1.30240500
H	0.87929400	3.74256800	1.42547000
C	-1.76953500	2.53074000	-0.05225500
H	-2.29674300	2.19246400	-0.94782000
H	-2.28266600	2.12731500	0.82581100
H	-1.84181600	3.62225100	-0.01170600
O	2.98508400	0.55966000	-0.17972500
S	3.40171400	-0.56523000	0.70462500
O	2.91837500	-1.85361100	0.13845400
O	4.83284200	-0.56038200	1.02695900
C	2.52059100	-0.34732400	2.23667800
C	-5.01705000	0.10636400	0.03563700
C	-3.88054000	-0.21267200	-0.69744100
C	-2.84236800	-0.94888700	-0.11287700
C	-2.96299800	-1.36141400	1.22028300
C	-4.09412500	-1.03370100	1.95832800
C	-5.11956700	-0.30057000	1.36440500
H	-5.81920000	0.67305800	-0.42225700
H	-3.79570400	0.11165500	-1.72929200
H	-2.16434900	-1.93292500	1.68151900
H	-4.17975900	-1.34819100	2.99176600

H	-6.00317400	-0.04674500	1.93913400
B	-1.57190200	-1.27218600	-0.91091500
F	-0.65637100	-2.15895800	-0.50811900
F	-1.30979900	-0.72887600	-2.10888900
F	-0.00444000	0.35702300	-0.00559500
K	1.48715800	-0.88960300	-1.80766900
H	2.84608600	0.58395700	2.69828900
H	2.75508100	-1.19144300	2.88453600
H	1.45331600	-0.31631700	2.02061400

VIII. Bond heterolysis energy and binding energy

Bond heterolysis energy of Si-X (X = O, Br) is calculated according to the following equation (1). Binding energy of $PhBF_2$ with sylvite is calculated according to the following equation (2).

$$E_{\text{Si-X heterolysis}} = E(\text{cation}) + E(\text{anion}) - E(\text{Lewis acid}) \quad (1)$$

$$E_b = E(PhBF_2) + E(KX) - E(PhBF_2\text{-}KX) \quad (2)$$

Supplementary Table 4. Bond heterolysis energies of Si-X (X = O, Br). Calculated energy data at the level of M06-2X/6-311+G(d,p)/SMD(CH_3CN).

	***E*(Lewis acid) (kcal/mol)**	***E*(cation) (kcal/mol)**	***E*(anion) (kcal/mol)**	**$E_{\text{Si-X heterolysis}}$ (kcal/mol)**
**TMSOTf**	-1370.57358	-408.93896	-961.57120	39.8
**TMSOMs**	-1072.84394	-408.93896	-663.81884	54.0
**TMSOAc**	-637.60376	-408.93896	-228.55245	70.5
**TMSBr**	-2983.37184	-408.93896	-2574.35247	50.4

Supplementary Table 5. Binding energies of $PhBF_2$ with sylvite. Calculated energy data at the level of M06-2X/6-311+G(d,p)/SMD(CH_3CN).

	**$E(PhBF_2)$ (kcal/mol)**	***E*(KX) (kcal/mol)**	**$E(PhBF_2\text{-}KX)$ (kcal/mol)**	**E_b (kcal/mol)**
**KOTf**	-456.21061	-1561.44240	-2017.66147	5.3
**KOMs**	-456.21061	-1263.69712	-1719.92994	13.9
**KOAc**	-456.21061	-828.44440	-1284.69561	25.4
**KBr**	-456.21061	-3174.22783	-3630.44114	1.7

IX. NMR Spectra

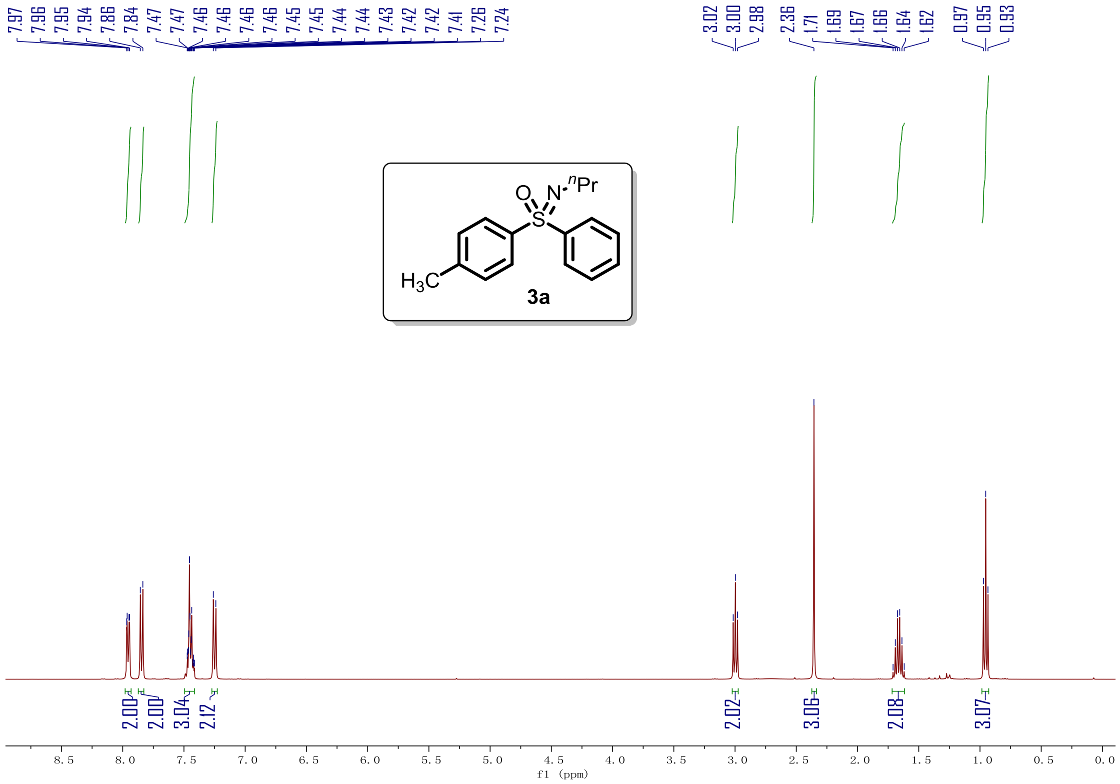

Supplementary Figure 10. ^{1}H NMR (400 MHz, $CDCl_3$) spectra of compound **3a.**

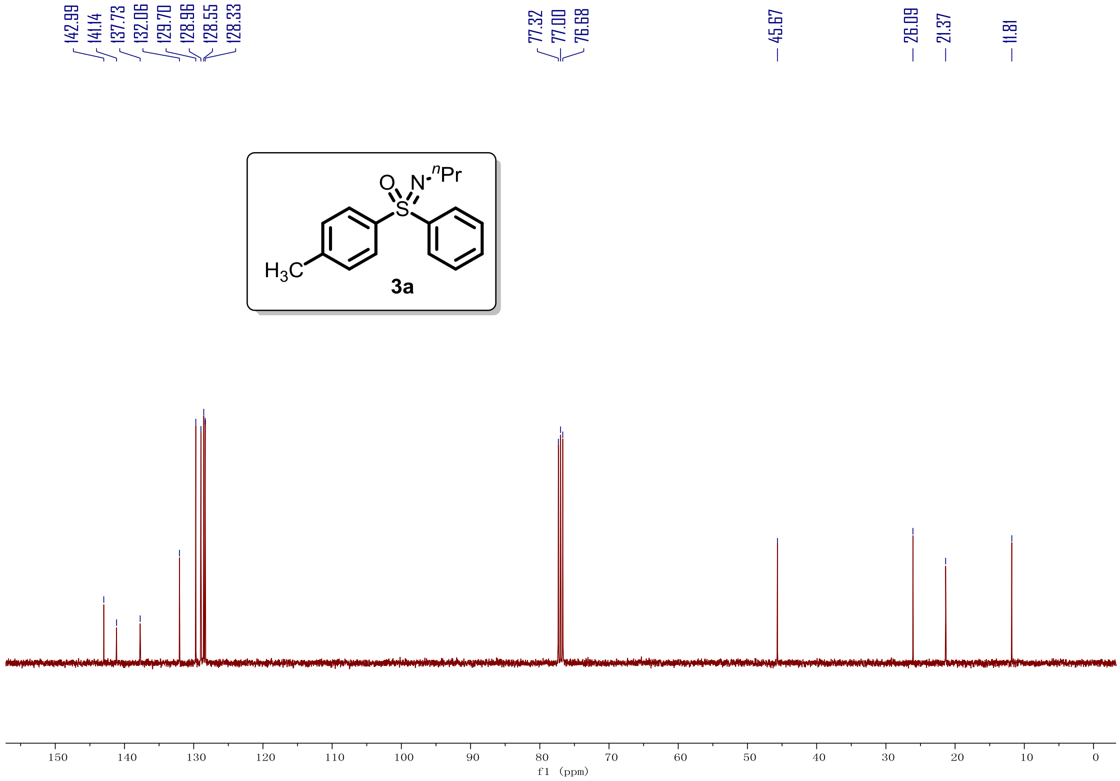

Supplementary Figure 11. ^{13}C NMR (101 MHz, $CDCl_3$) spectra of compound **3a.**

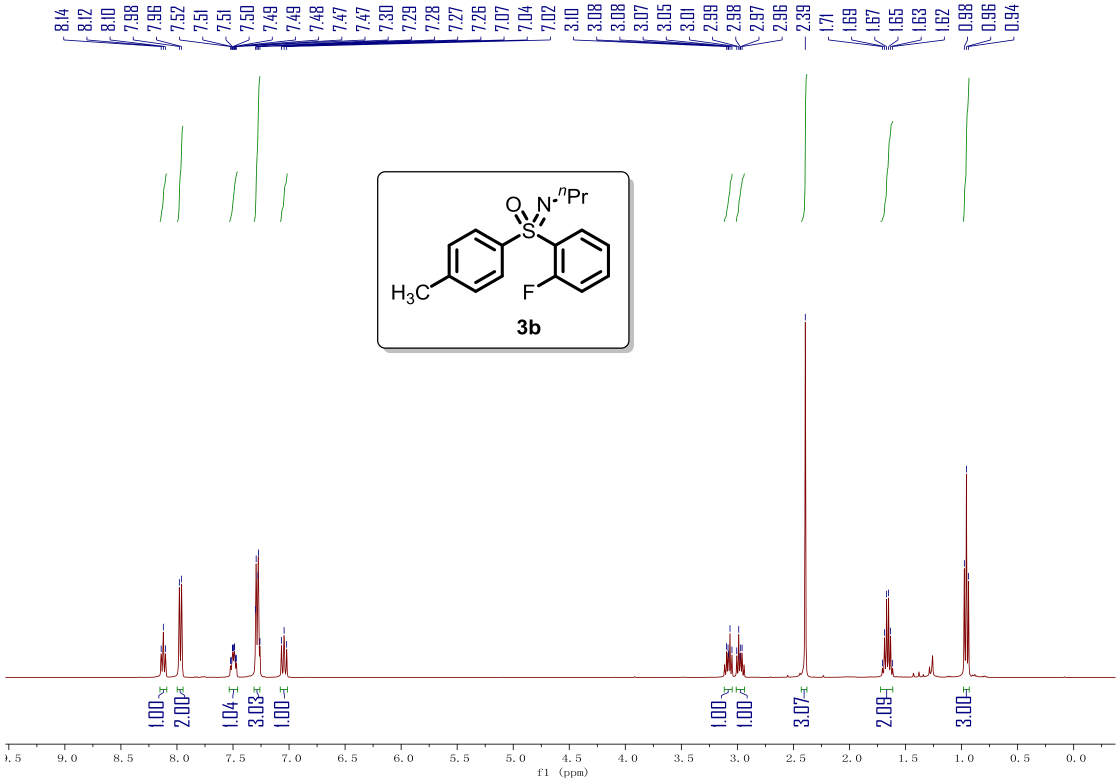

Supplementary Figure 12. ^{1}H NMR (400 MHz, $CDCl_3$) spectra of compound **3b.**

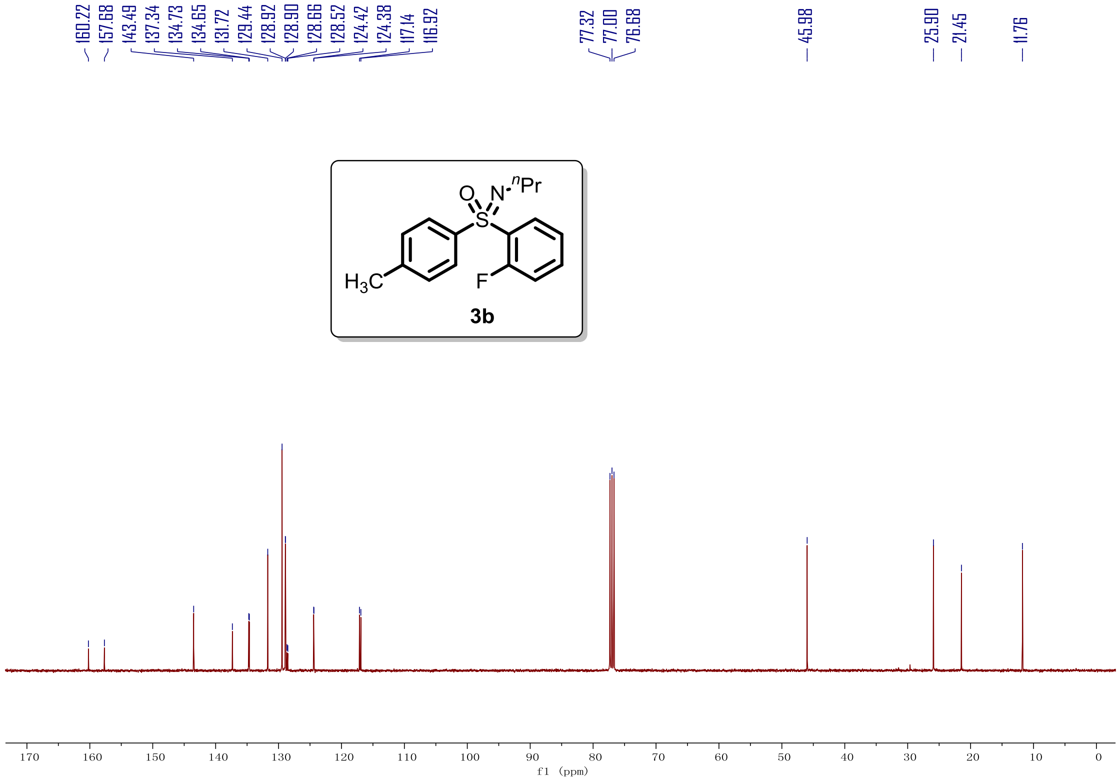

Supplementary Figure 13. ^{13}C NMR (101 MHz, $CDCl_3$) spectra of compound **3b.**

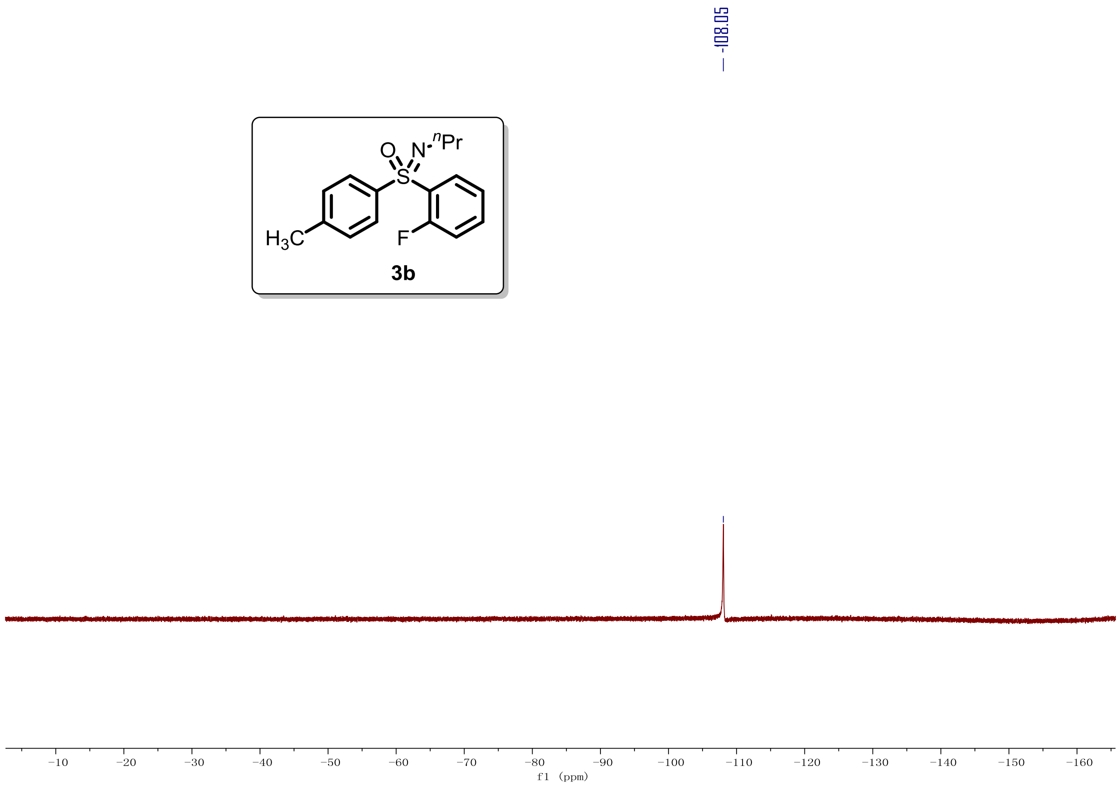

Supplementary Figure 14. ^{19}F NMR (376 MHz, $CDCl_3$) spectra of compound **3b.**

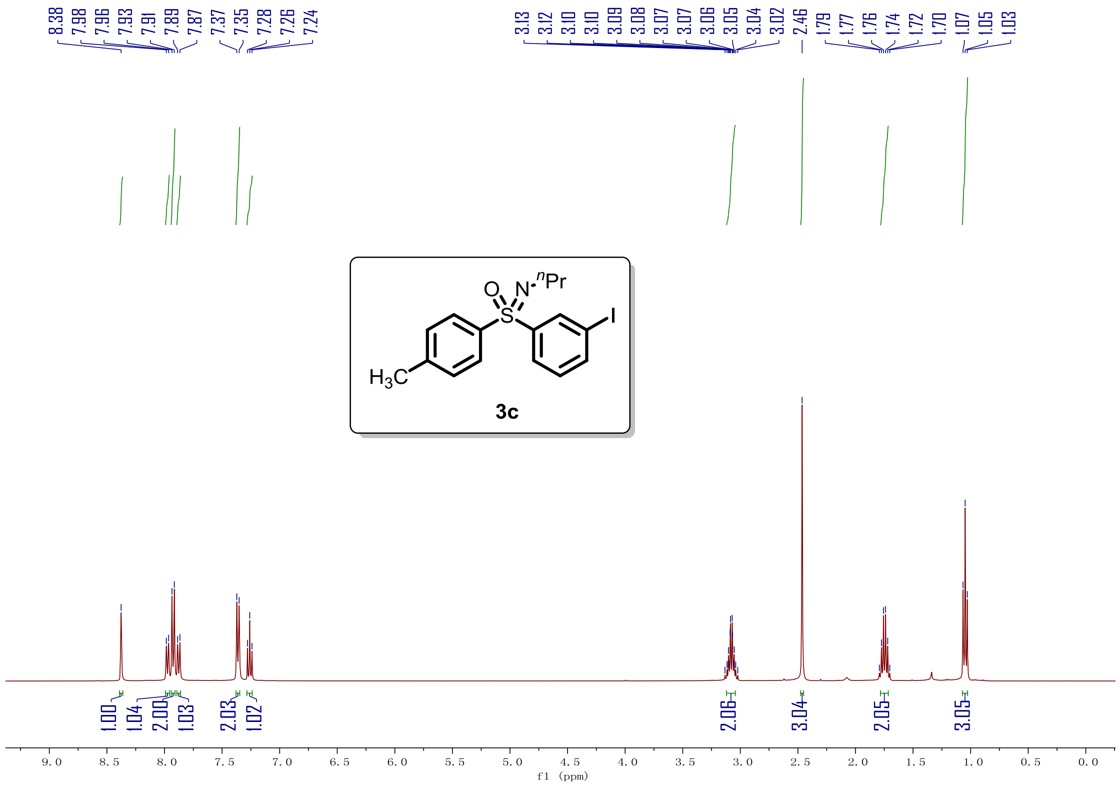

Supplementary Figure 15. ^{1}H NMR (400 MHz, $CDCl_3$) spectra of compound **3c.**

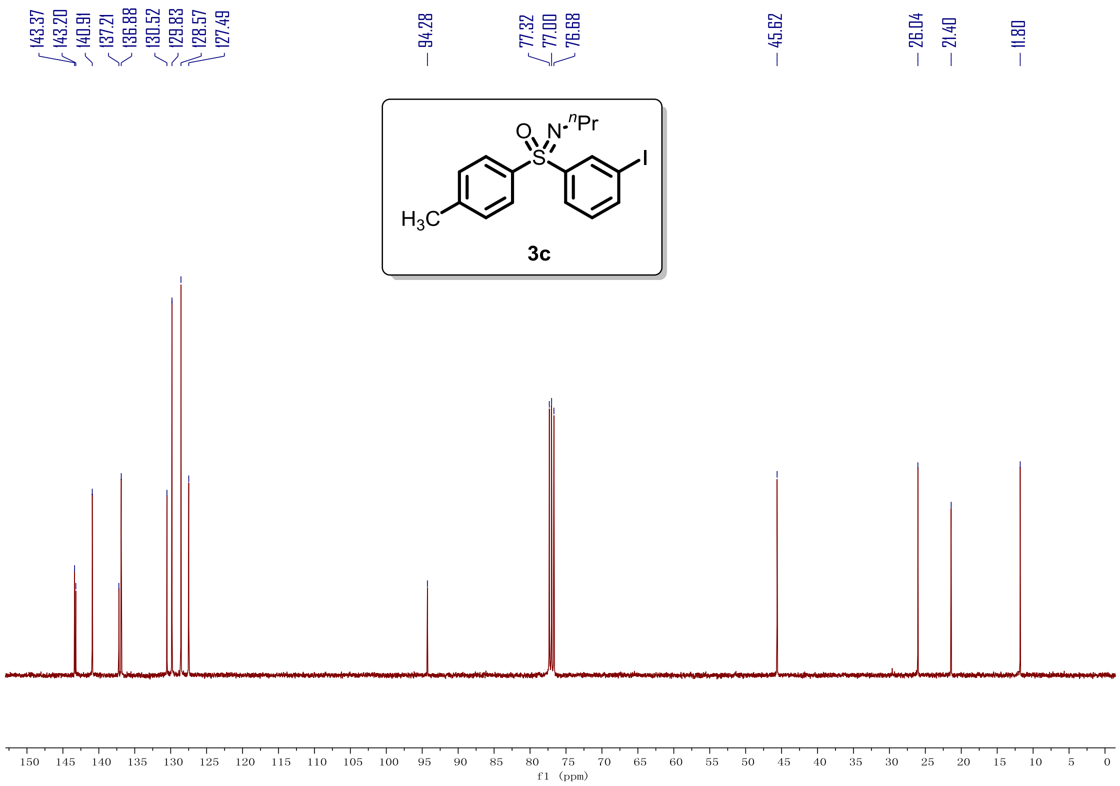

Supplementary Figure 16. ^{13}C NMR (101 MHz, $CDCl_3$) spectra of compound **3c.**

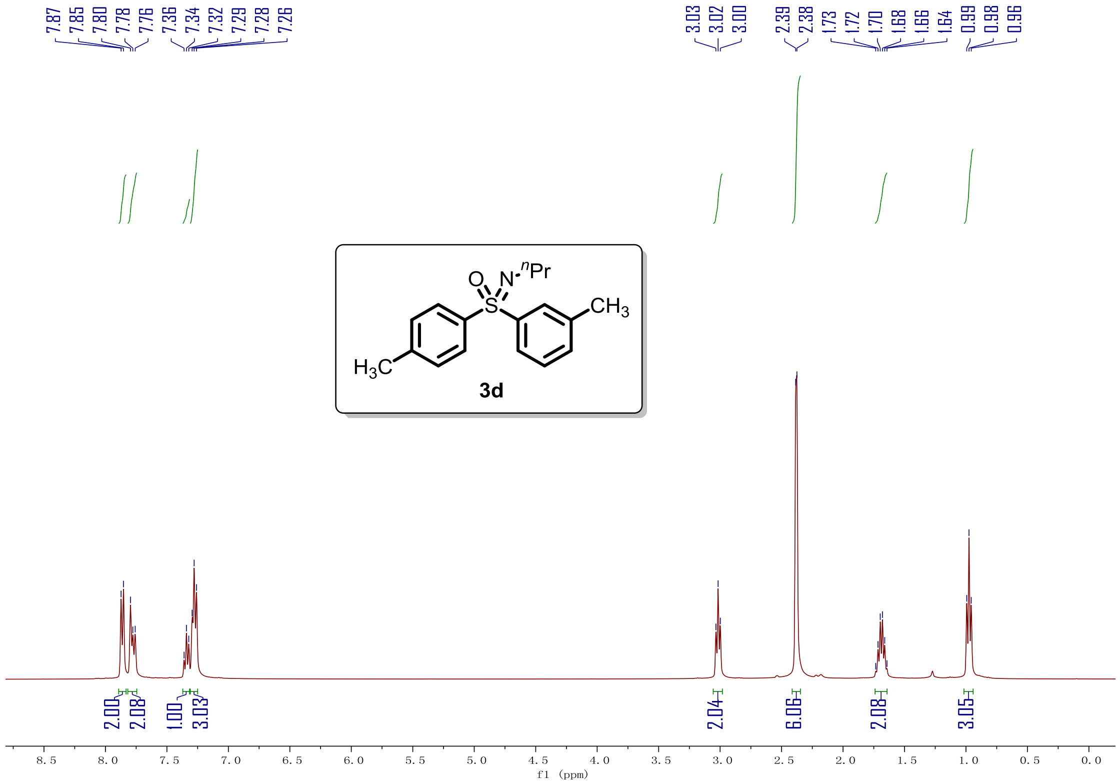

Supplementary Figure 17. ^{1}H NMR (400 MHz, $CDCl_3$) spectra of compound **3d.**

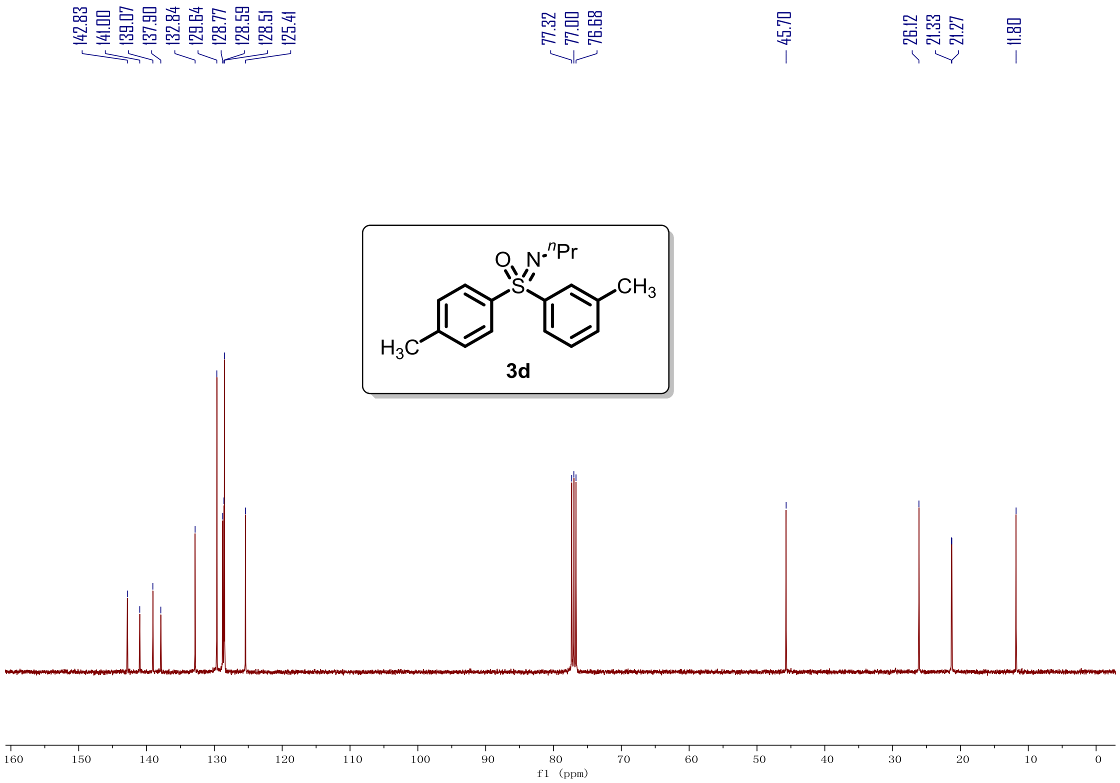

Supplementary Figure 18. ^{13}C NMR (101 MHz, $CDCl_3$) spectra of compound **3d.**

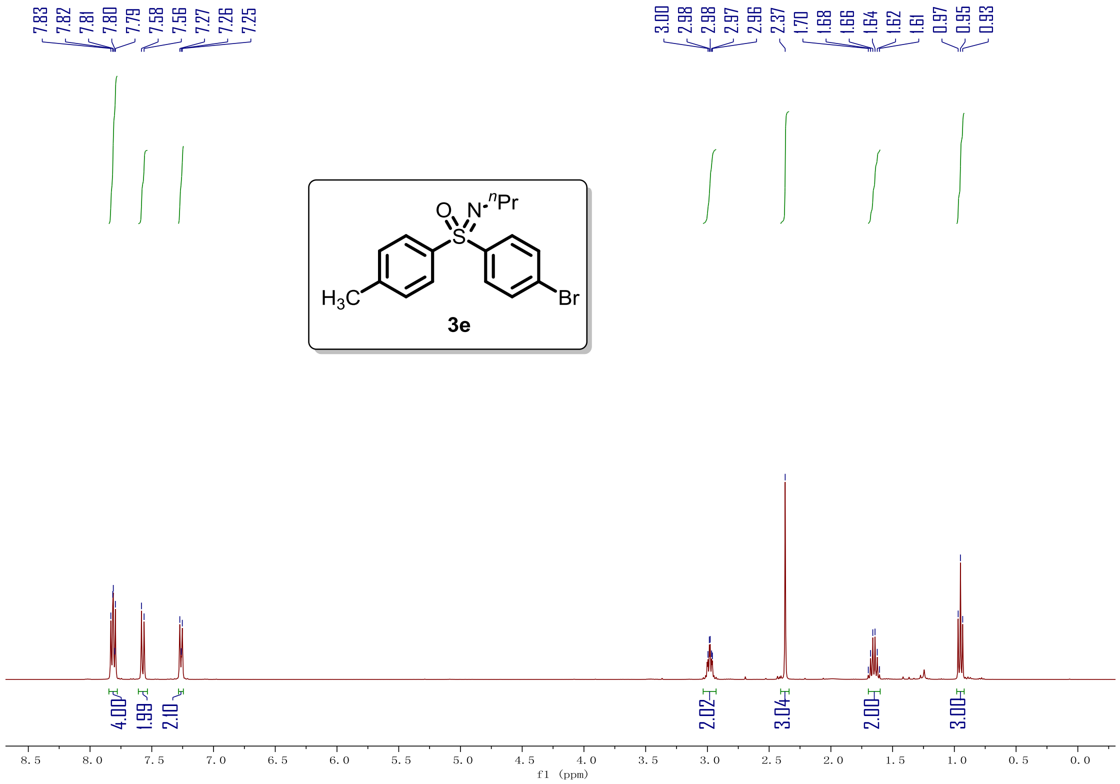

Supplementary Figure 19. ^{1}H NMR (400 MHz, $CDCl_3$) spectra of compound **3e.**

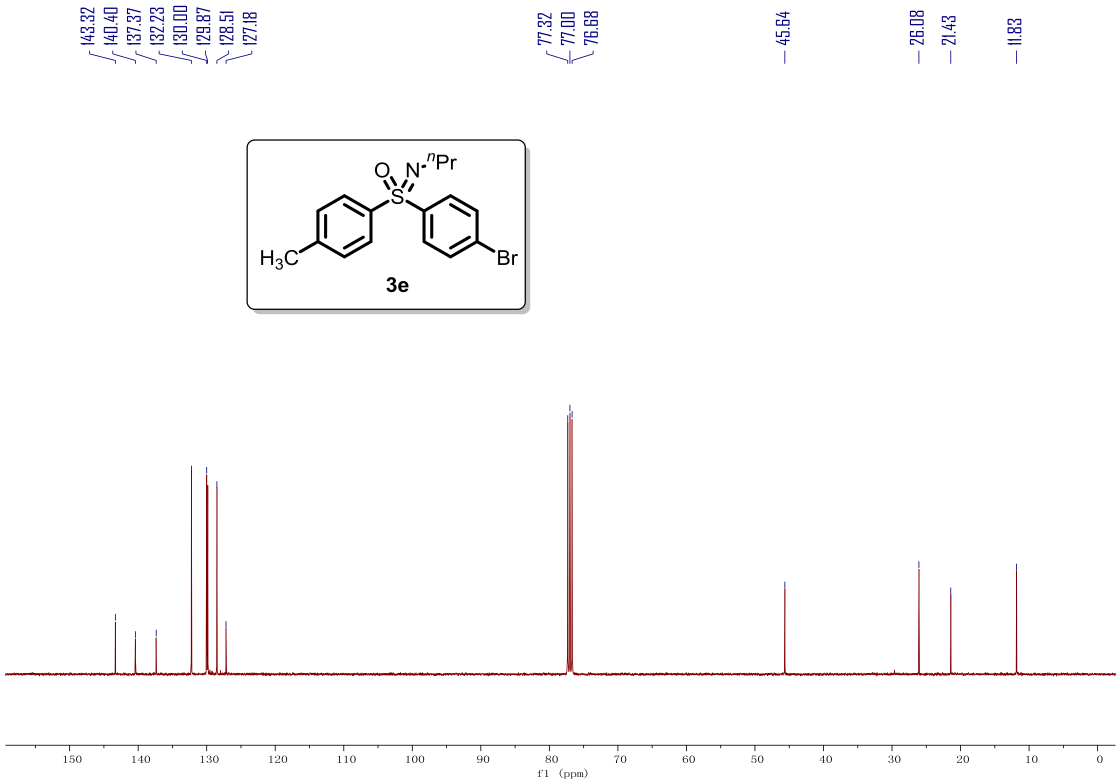

Supplementary Figure 20. ^{13}C NMR (101 MHz, $CDCl_3$) spectra of compound **3e.**

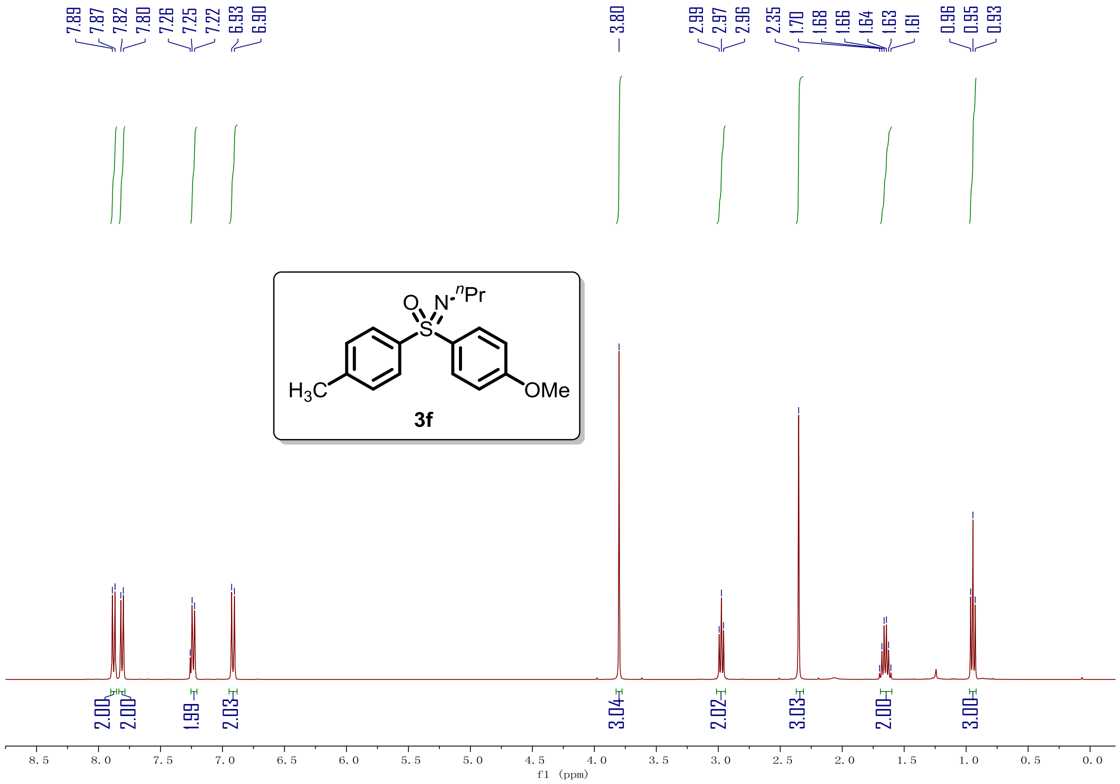

Supplementary Figure 21. ^{1}H NMR (400 MHz, $CDCl_3$) spectra of compound **3f.**

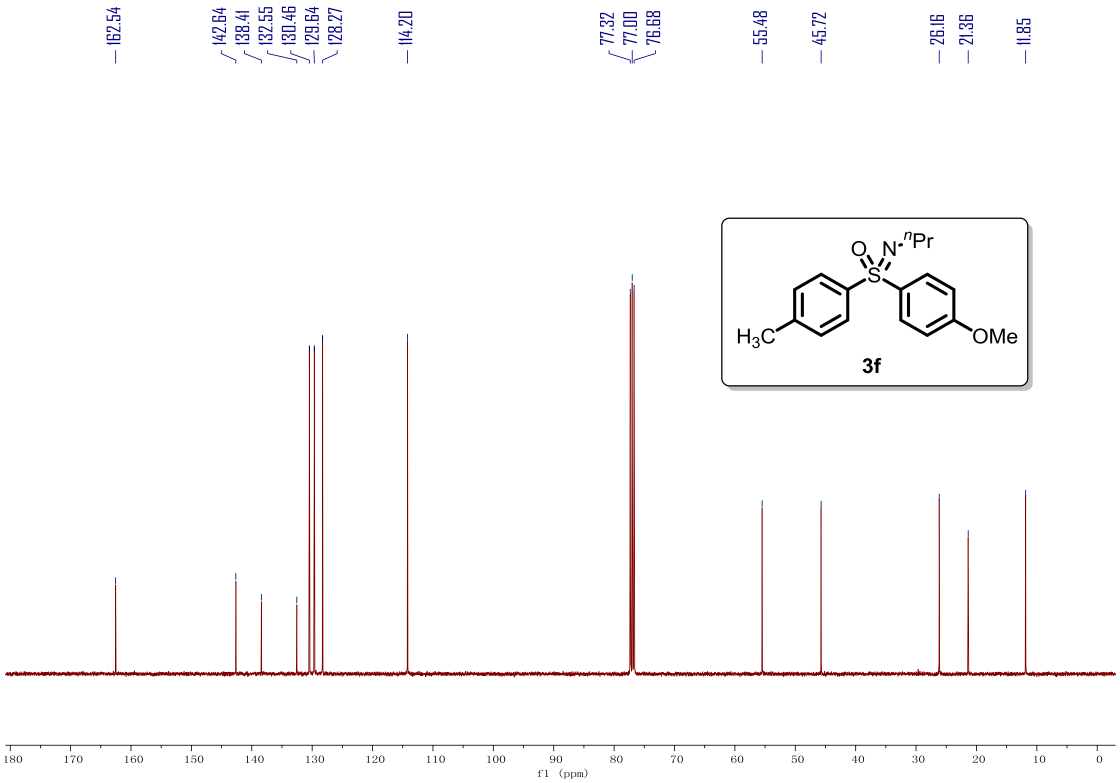

Supplementary Figure 22. ^{13}C NMR (101 MHz, $CDCl_3$) spectra of compound **3f.**

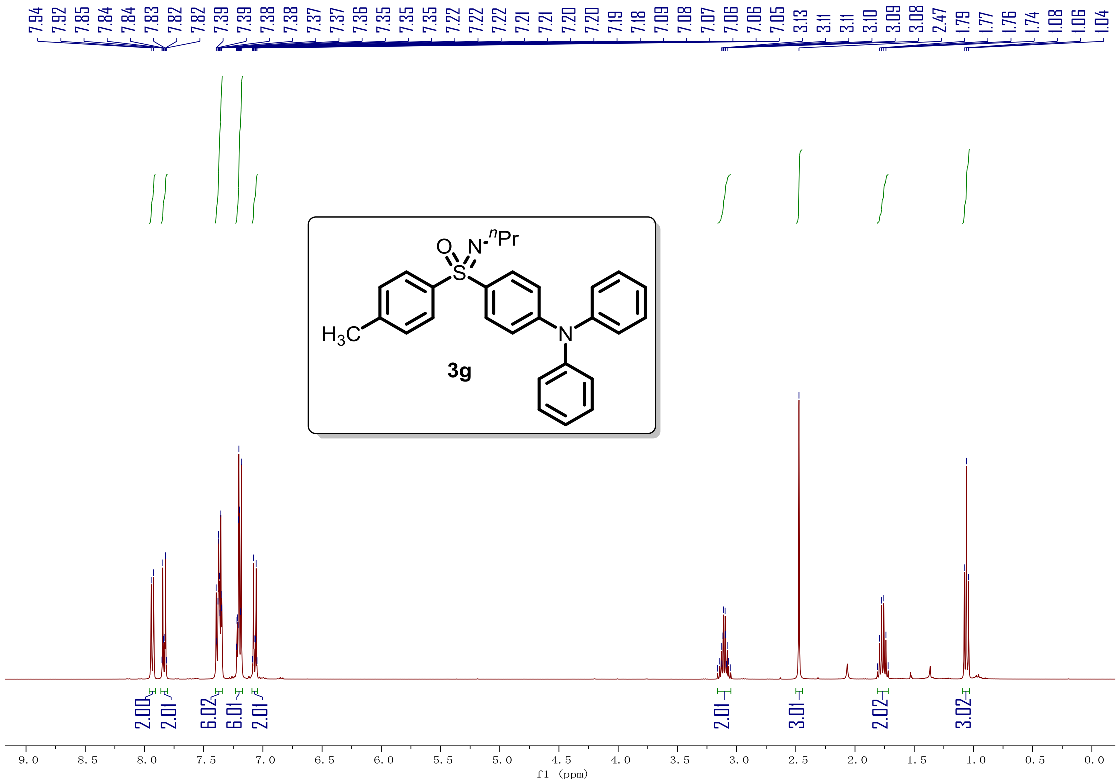

Supplementary Figure 23. ^{1}H NMR (400 MHz, $CDCl_3$) spectra of compound **3g.**

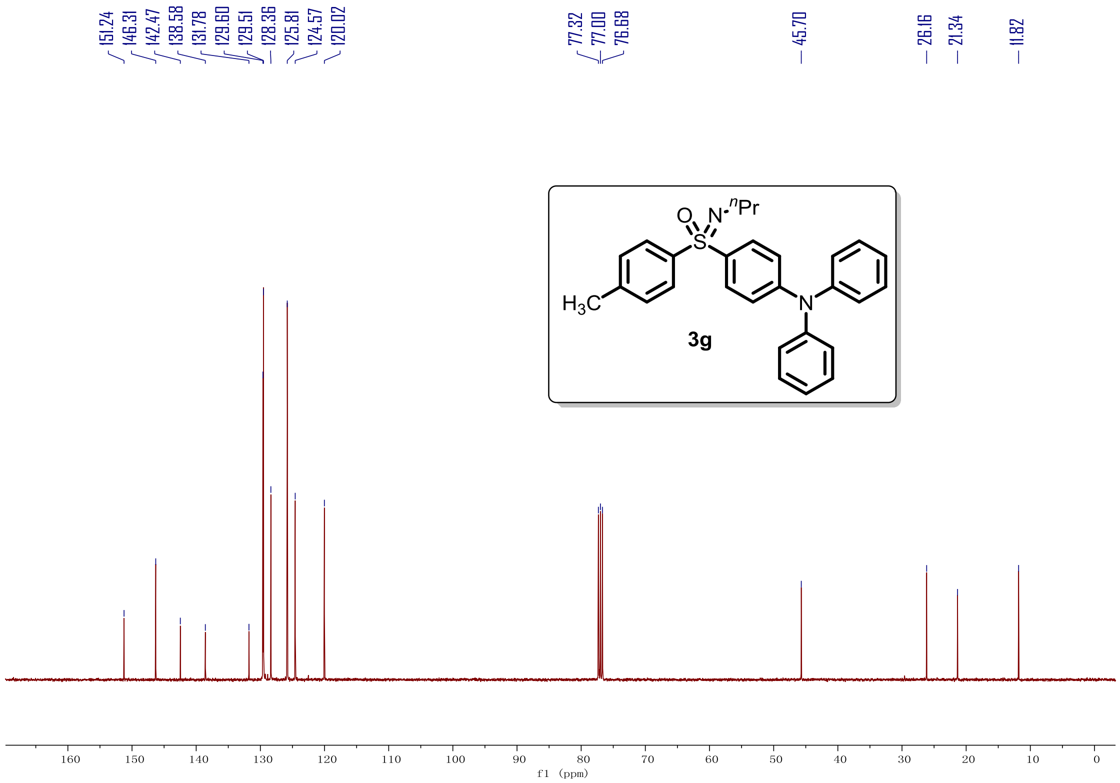

Supplementary Figure 24. ^{13}C NMR (101 MHz, $CDCl_3$) spectra of compound **3g.**

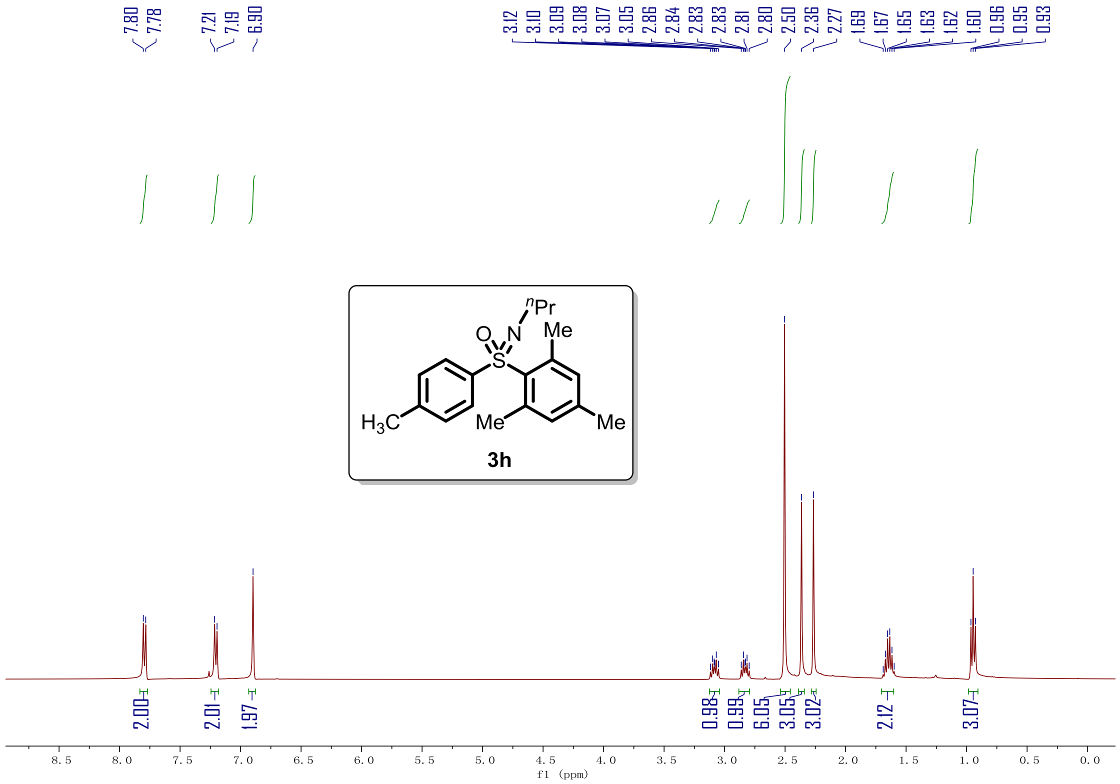

Supplementary Figure 25. ^{1}H NMR (400 MHz, $CDCl_3$) spectra of compound **3h.**

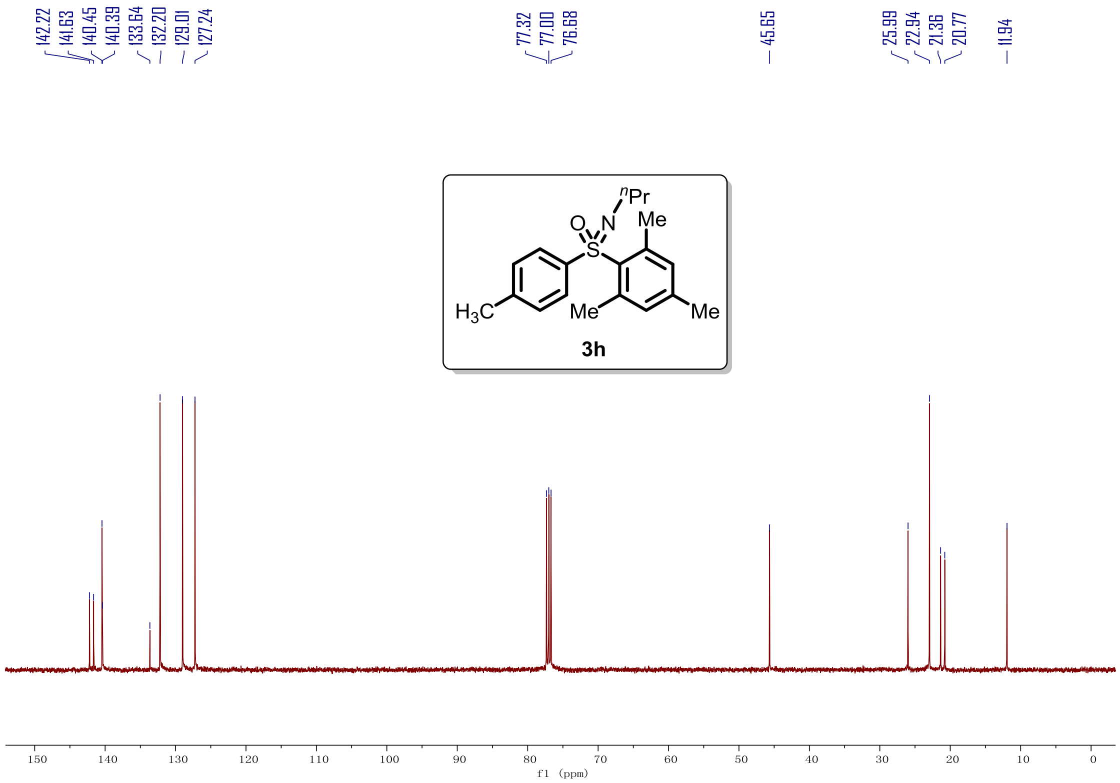

Supplementary Figure 26. ^{13}C NMR (101 MHz, $CDCl_3$) spectra of compound **3h.**

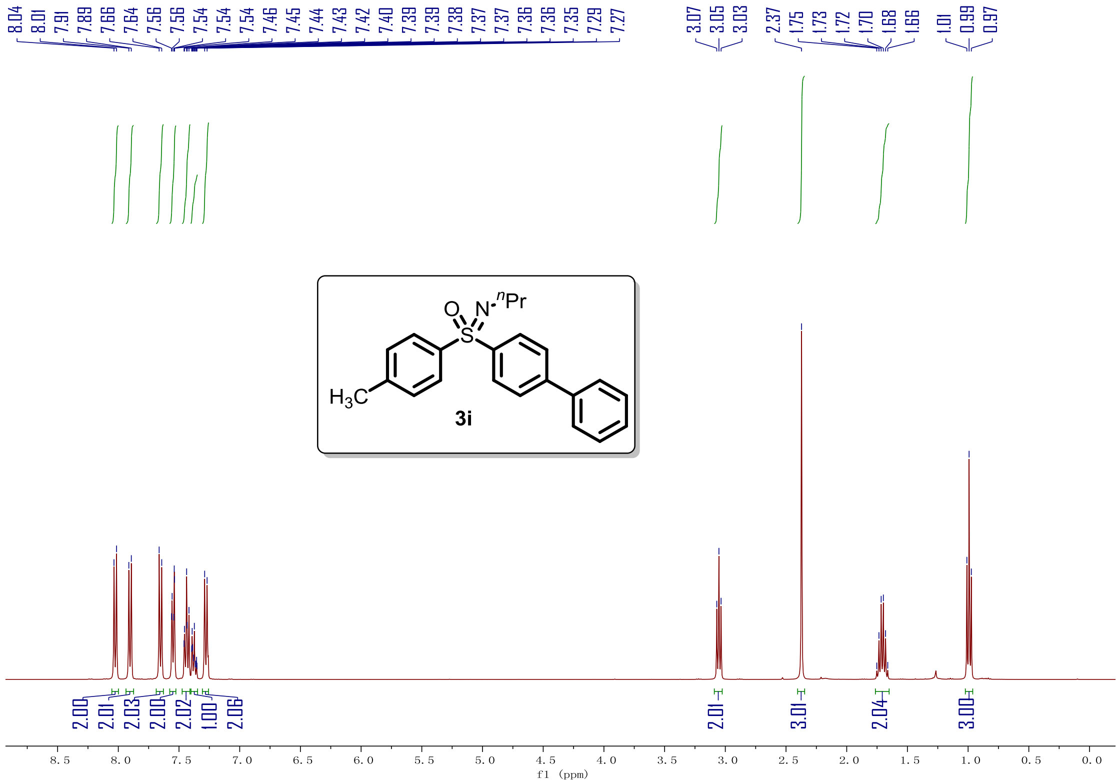

Supplementary Figure 27. ^{1}H NMR (400 MHz, $CDCl_3$) spectra of compound **3i.**

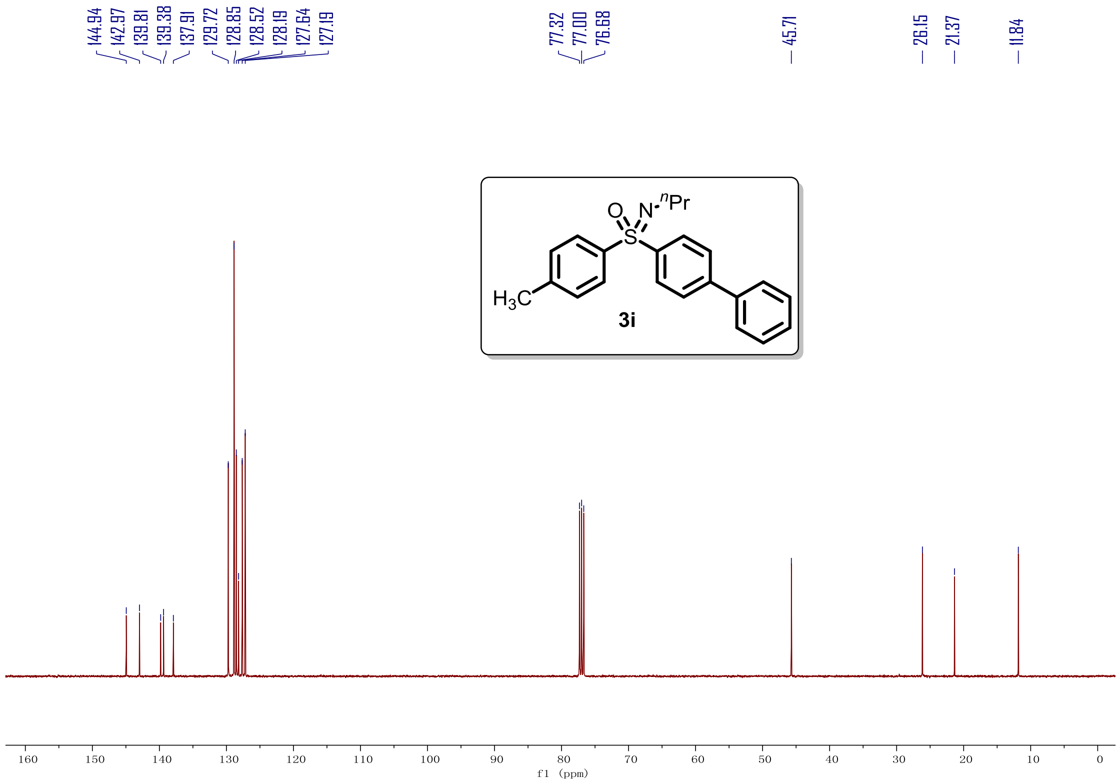

Supplementary Figure 28. ^{13}C NMR (101 MHz, $CDCl_3$) spectra of compound **3i.**

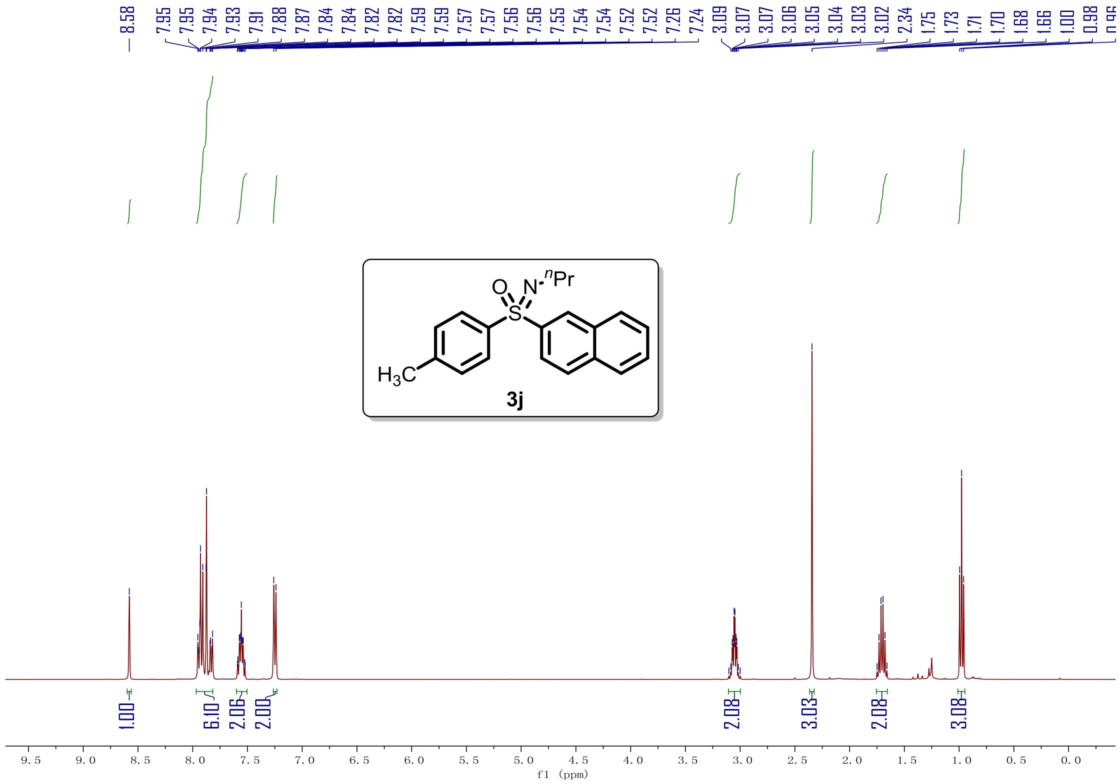

Supplementary Figure 29. ^{1}H NMR (400 MHz, $CDCl_3$) spectra of compound **3j.**

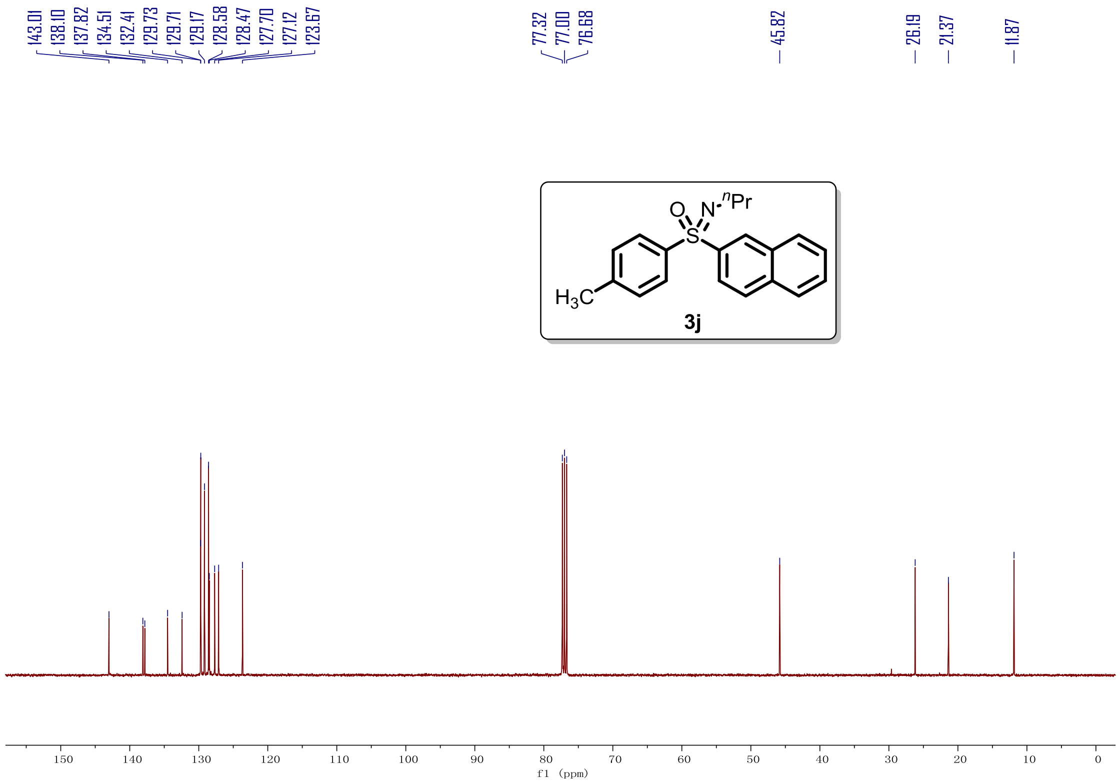

Supplementary Figure 30. ^{13}C NMR (101 MHz, $CDCl_3$) spectra of compound **3j.**

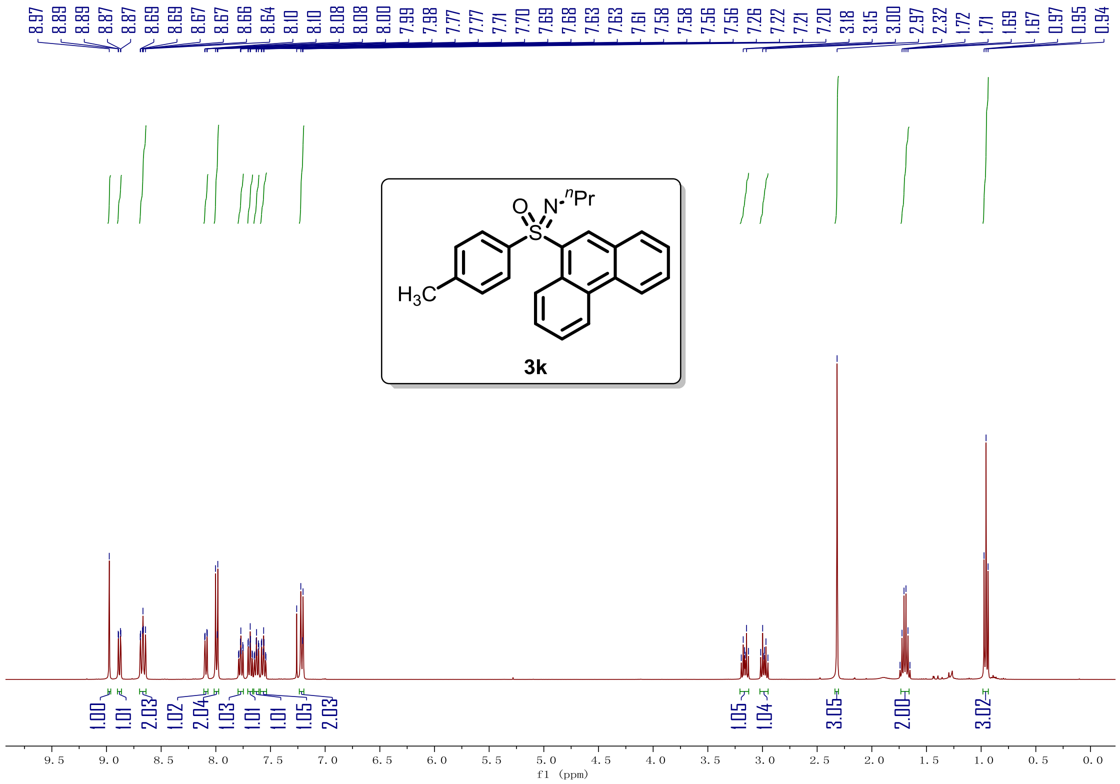

Supplementary Figure 31. ^{1}H NMR (400 MHz, $CDCl_3$) spectra of compound **3k.**

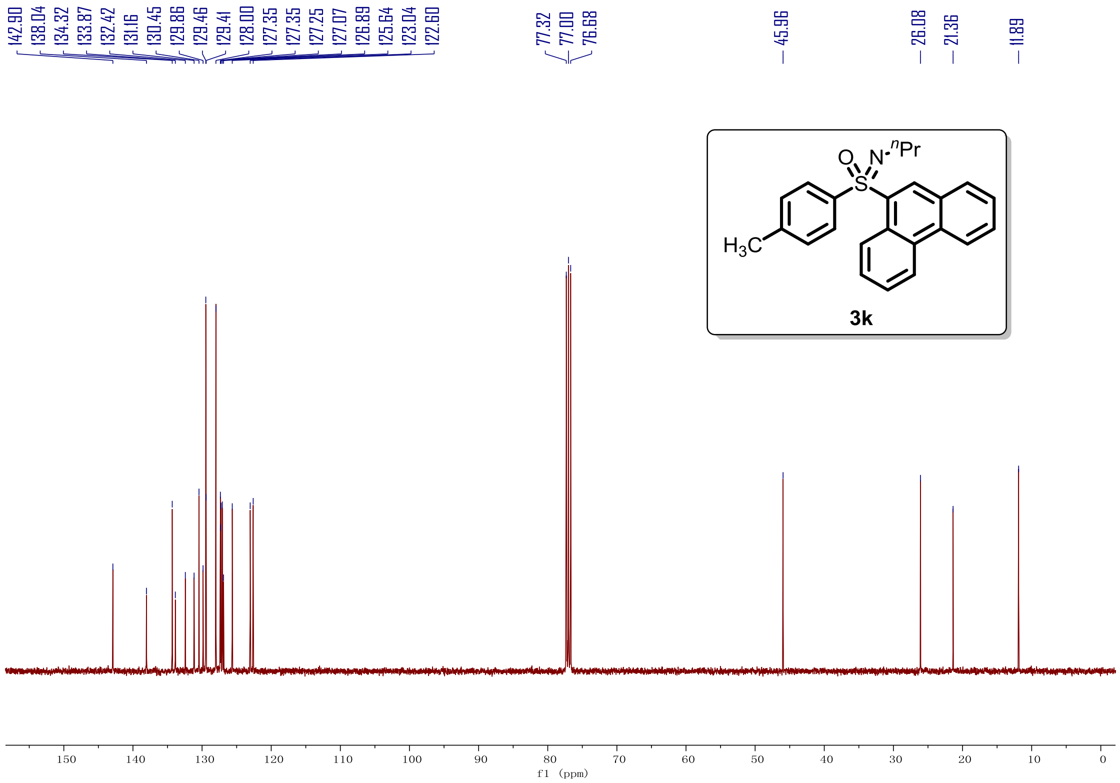

Supplementary Figure 32. ^{13}C NMR (101 MHz, $CDCl_3$) spectra of compound **3k.**

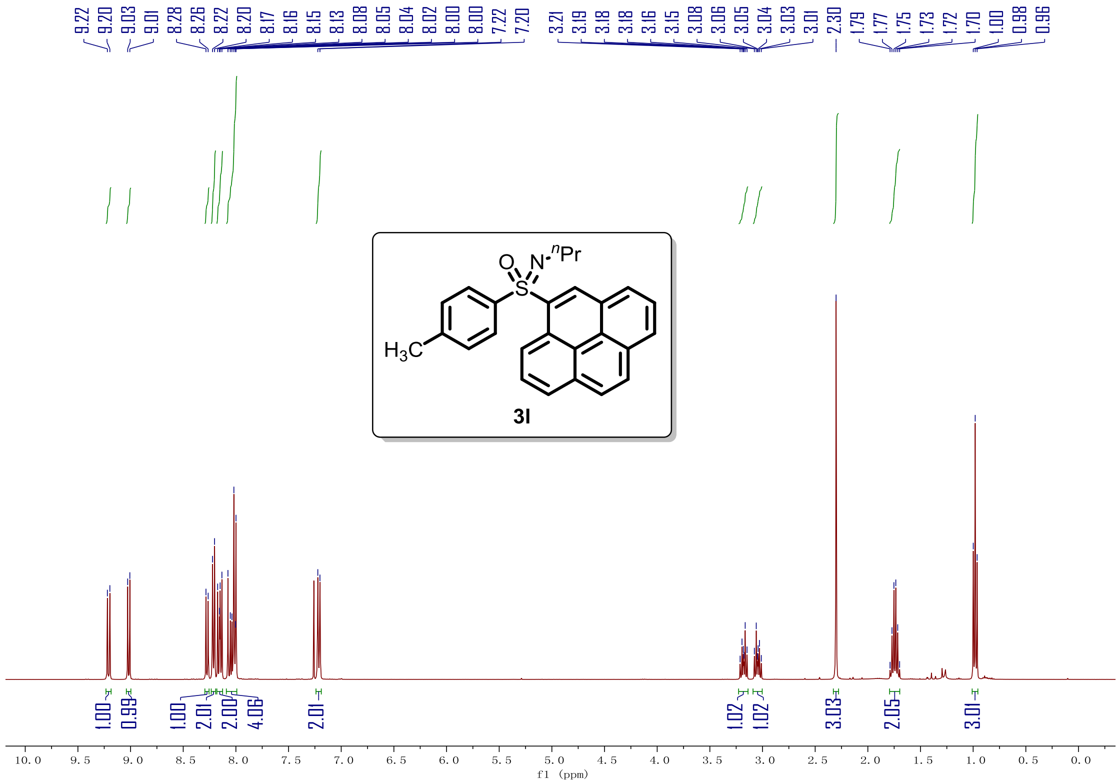

Supplementary Figure 33. ^{1}H NMR (400 MHz, $CDCl_3$) spectra of compound **3l.**

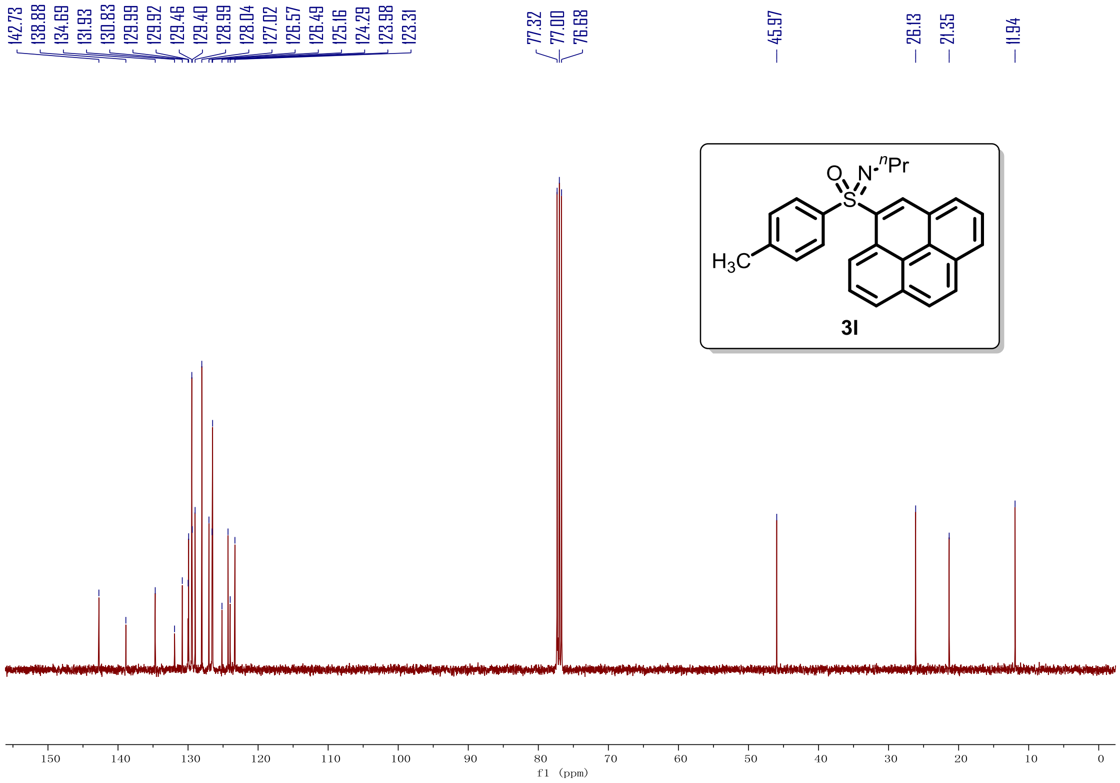

Supplementary Figure 34. ^{13}C NMR (101 MHz, $CDCl_3$) spectra of compound **3l.**

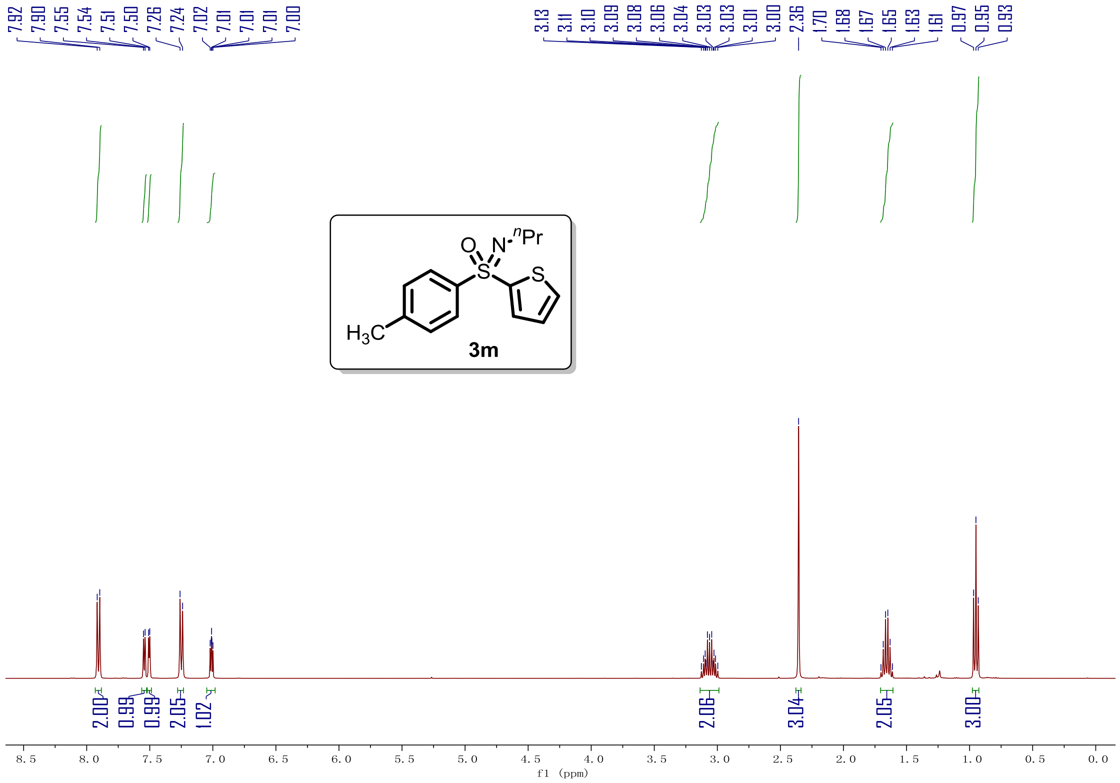

Supplementary Figure 35. ^{1}H NMR (400 MHz, $CDCl_3$) spectra of compound **3m.**

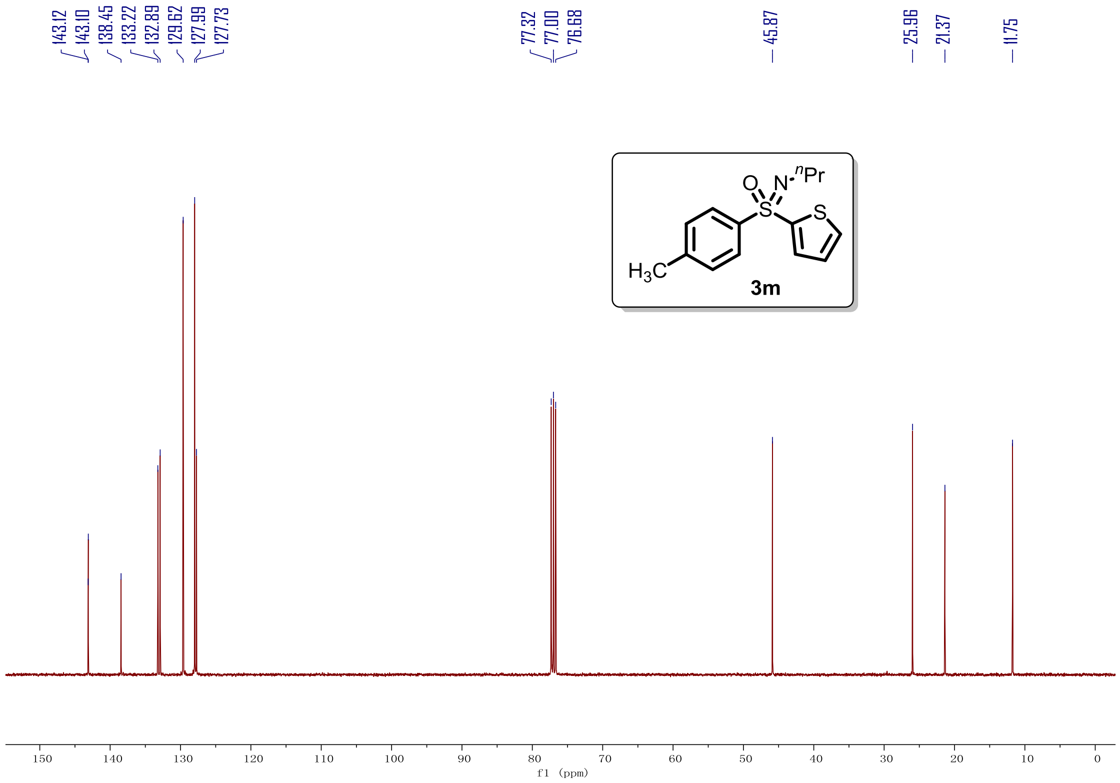

Supplementary Figure 36. ^{13}C NMR (101 MHz, $CDCl_3$) spectra of compound **3m.**

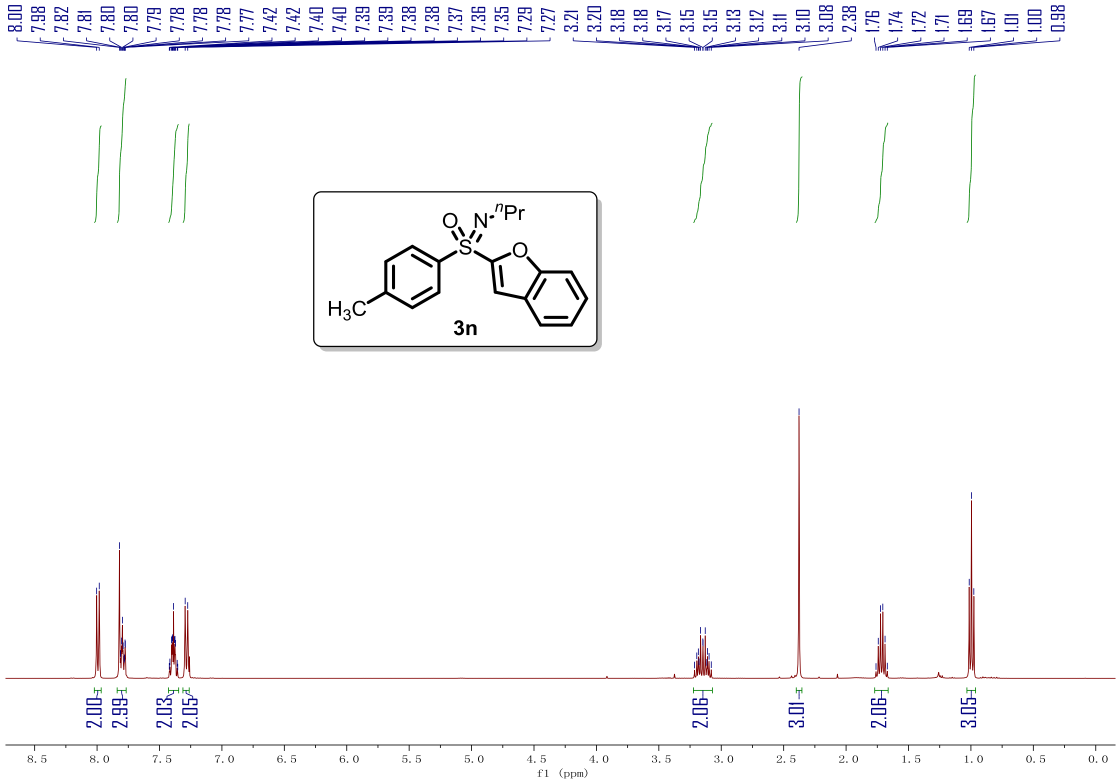

Supplementary Figure 37. ^{1}H NMR (400 MHz, $CDCl_3$) spectra of compound **3n.**

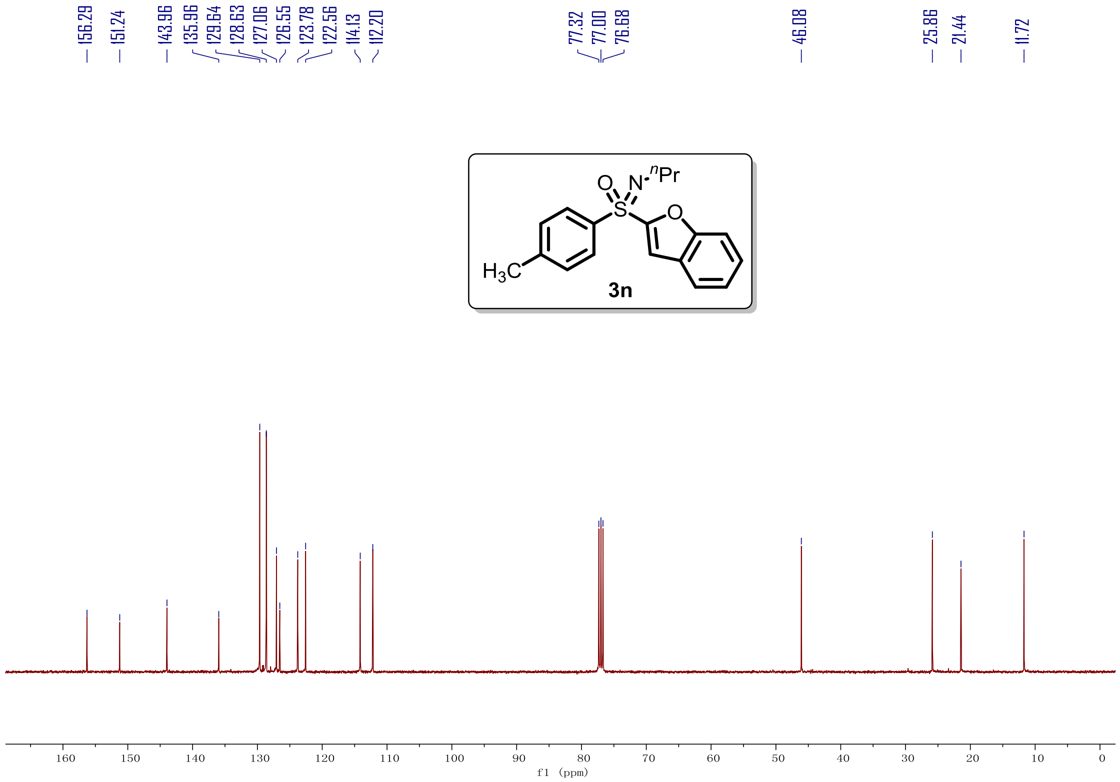

Supplementary Figure 38. ^{13}C NMR (101 MHz, $CDCl_3$) spectra of compound **3n.**

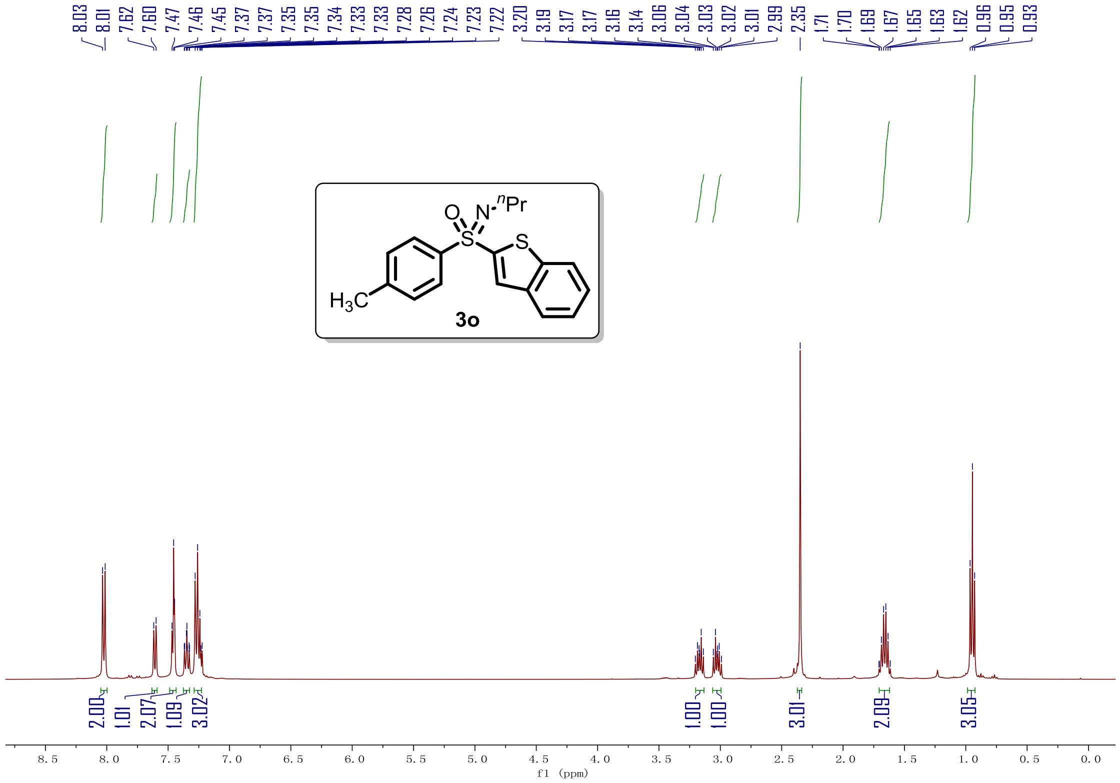

Supplementary Figure 39. ^{1}H NMR (400 MHz, $CDCl_3$) spectra of compound **3o.**

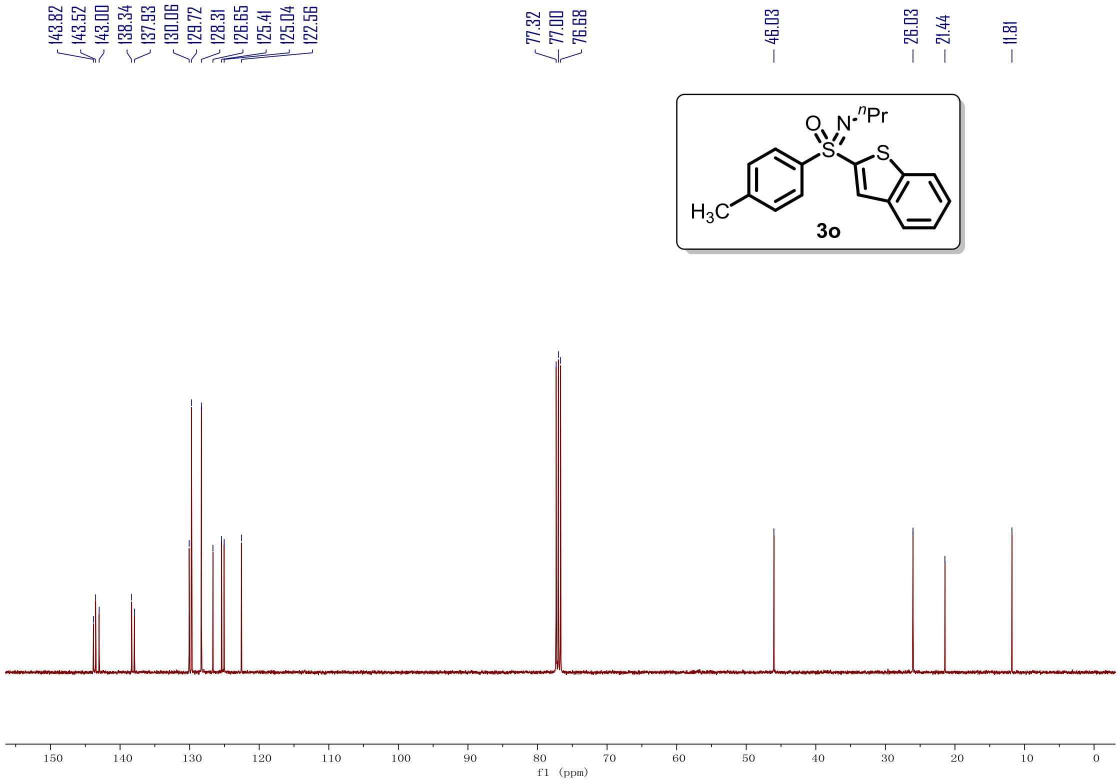

Supplementary Figure 40. ^{13}C NMR (101 MHz, $CDCl_3$) spectra of compound **3o.**

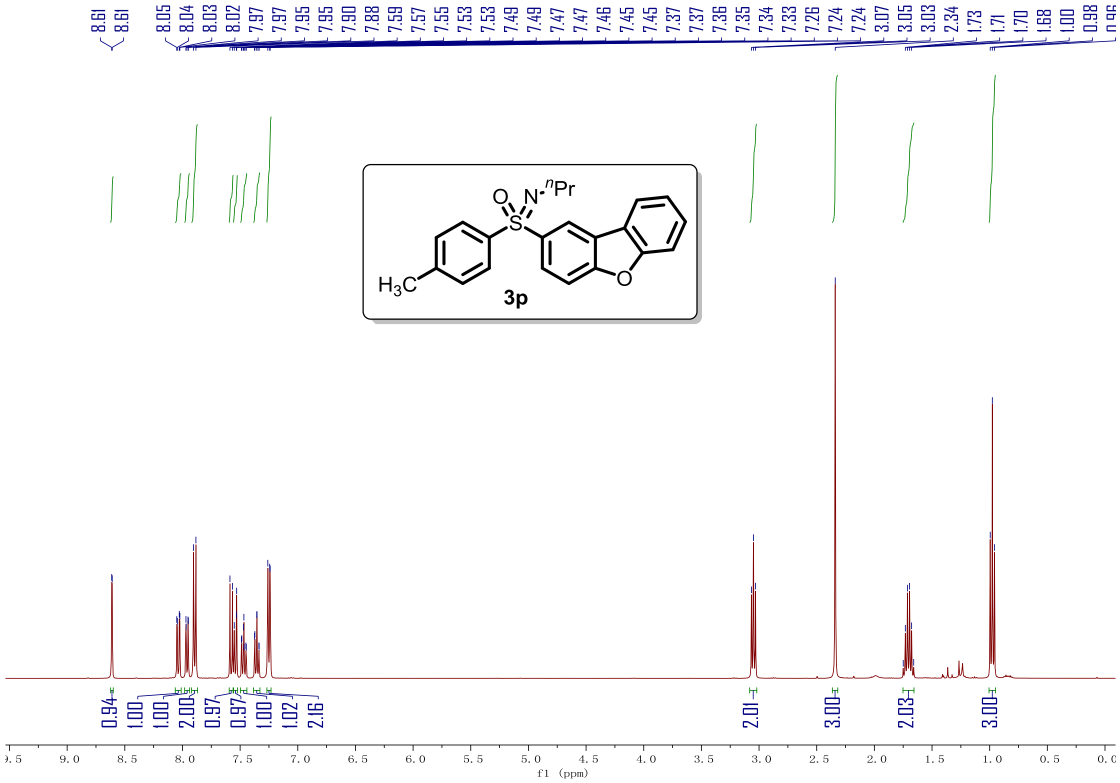

Supplementary Figure 41. ^{1}H NMR (400 MHz, $CDCl_3$) spectra of compound **3p.**

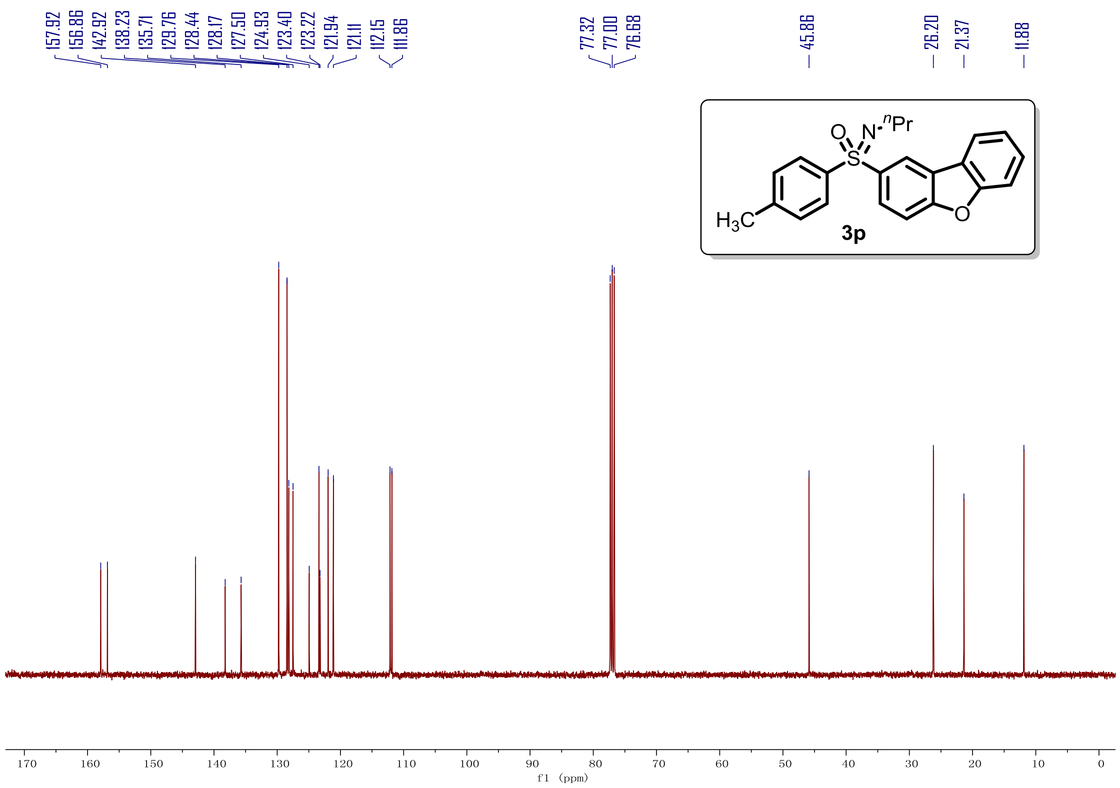

Supplementary Figure 42. ^{13}C NMR (101 MHz, $CDCl_3$) spectra of compound **3p.**

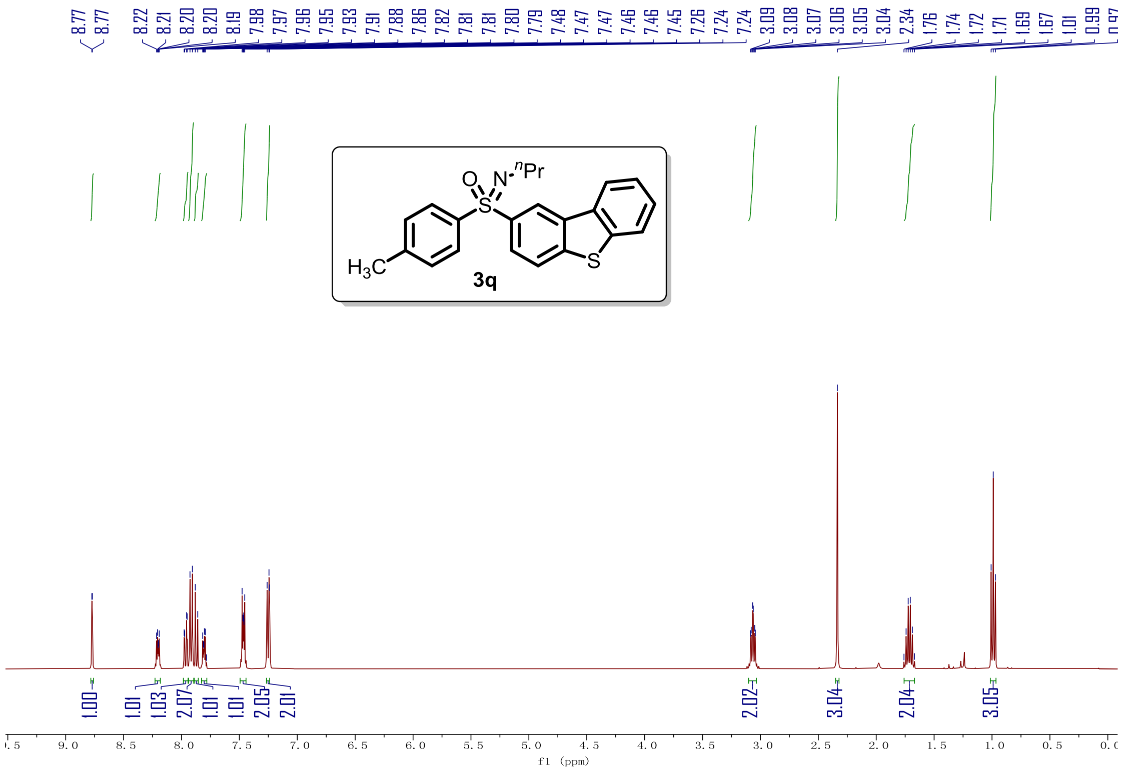

Supplementary Figure 43. ^{1}H NMR (400 MHz, $CDCl_3$) spectra of compound **3q.**

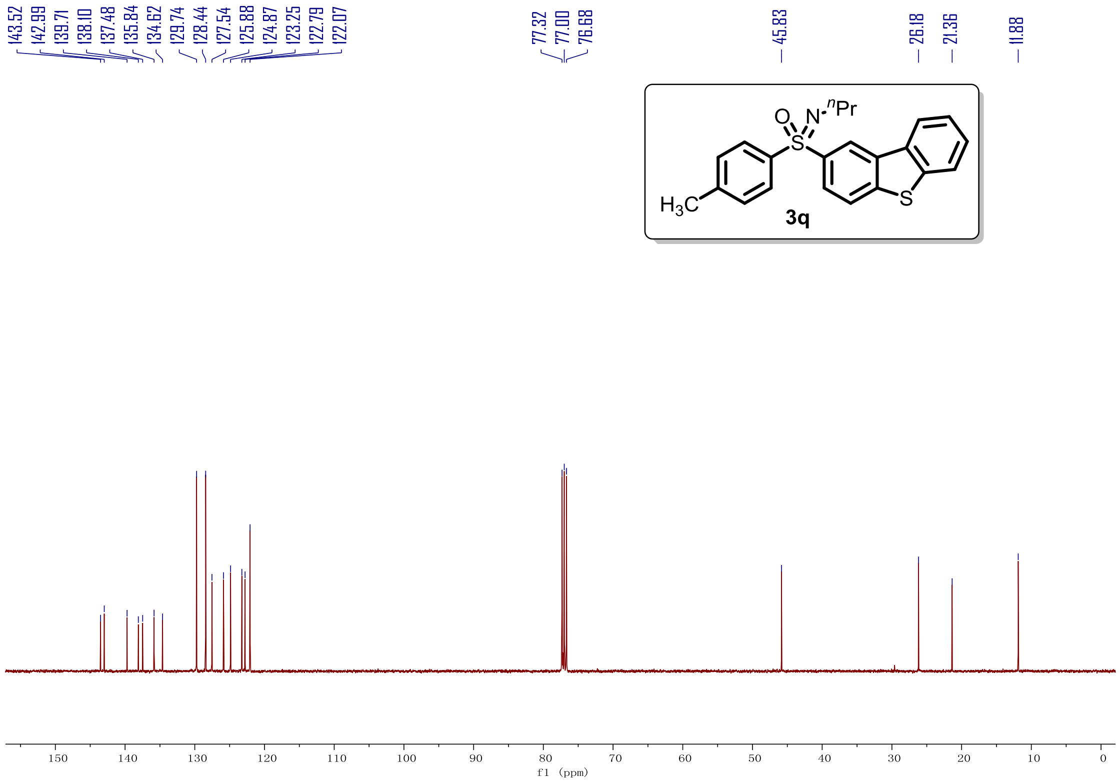

Supplementary Figure 44. ^{13}C NMR (101 MHz, $CDCl_3$) spectra of compound **3q.**

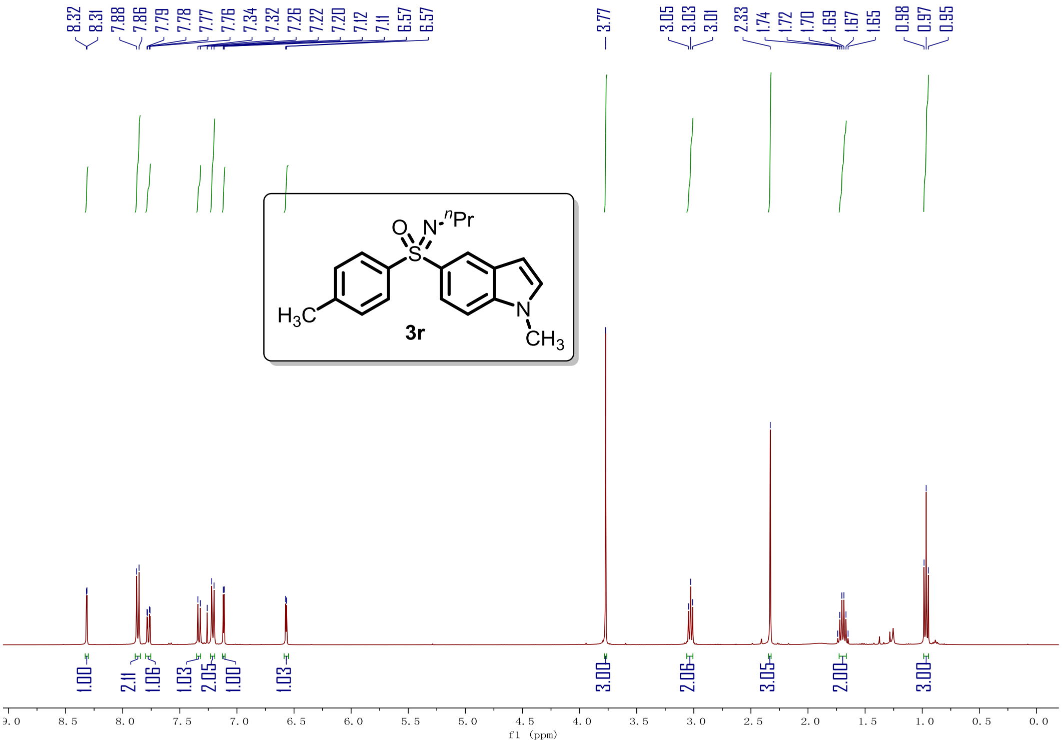

Supplementary Figure 45. ^{1}H NMR (400 MHz, $CDCl_3$) spectra of compound **3r.**

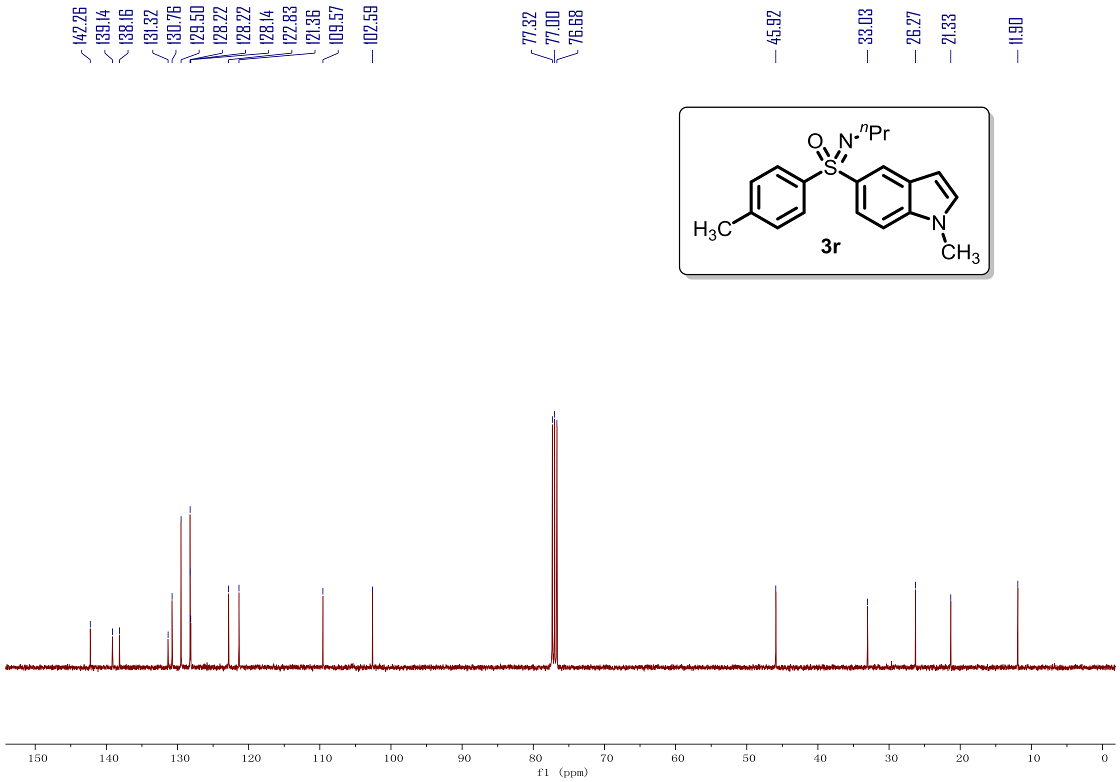

Supplementary Figure 46. ^{13}C NMR (101 MHz, $CDCl_3$) spectra of compound **3r.**

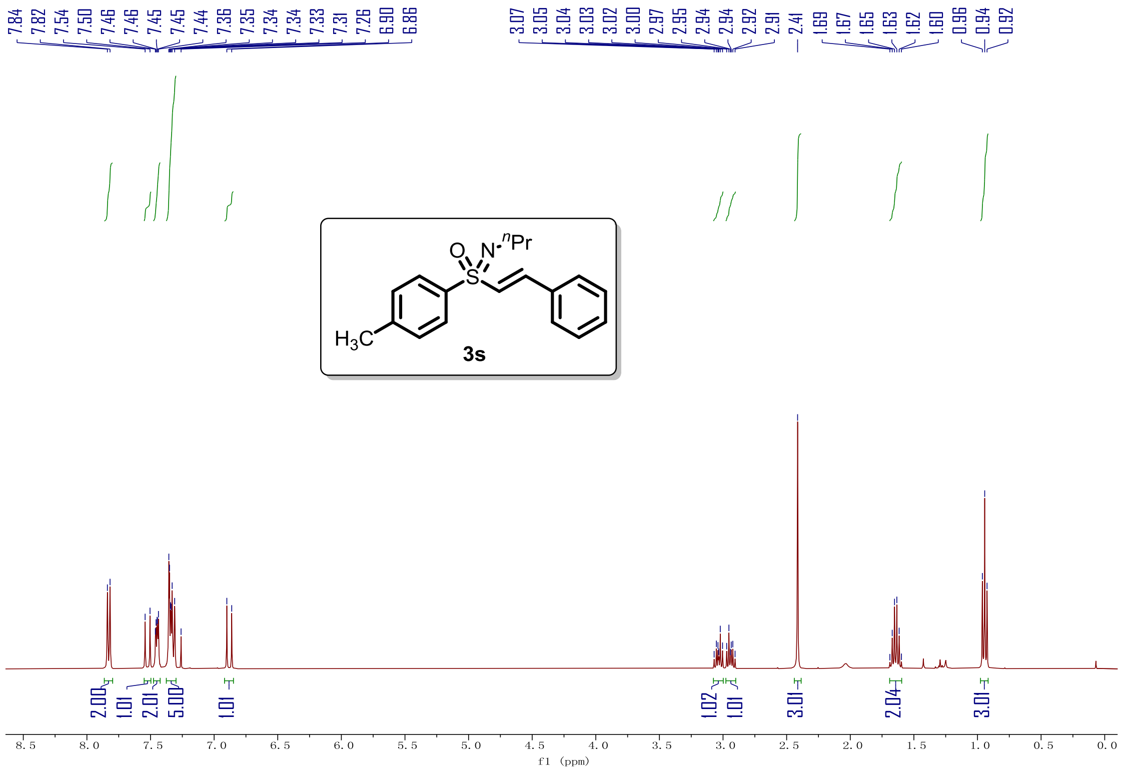

Supplementary Figure 47. ^{1}H NMR (400 MHz, $CDCl_3$) spectra of compound **3s.**

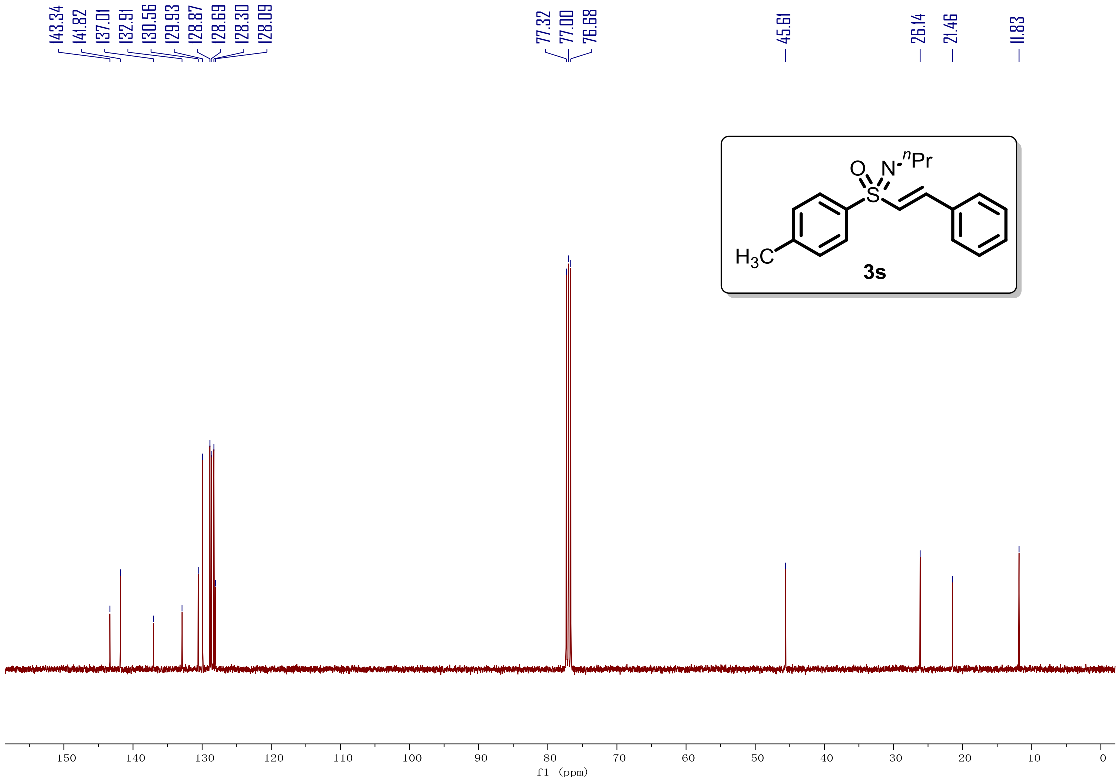

Supplementary Figure 48. ^{13}C NMR (101 MHz, $CDCl_3$) spectra of compound **3s.**

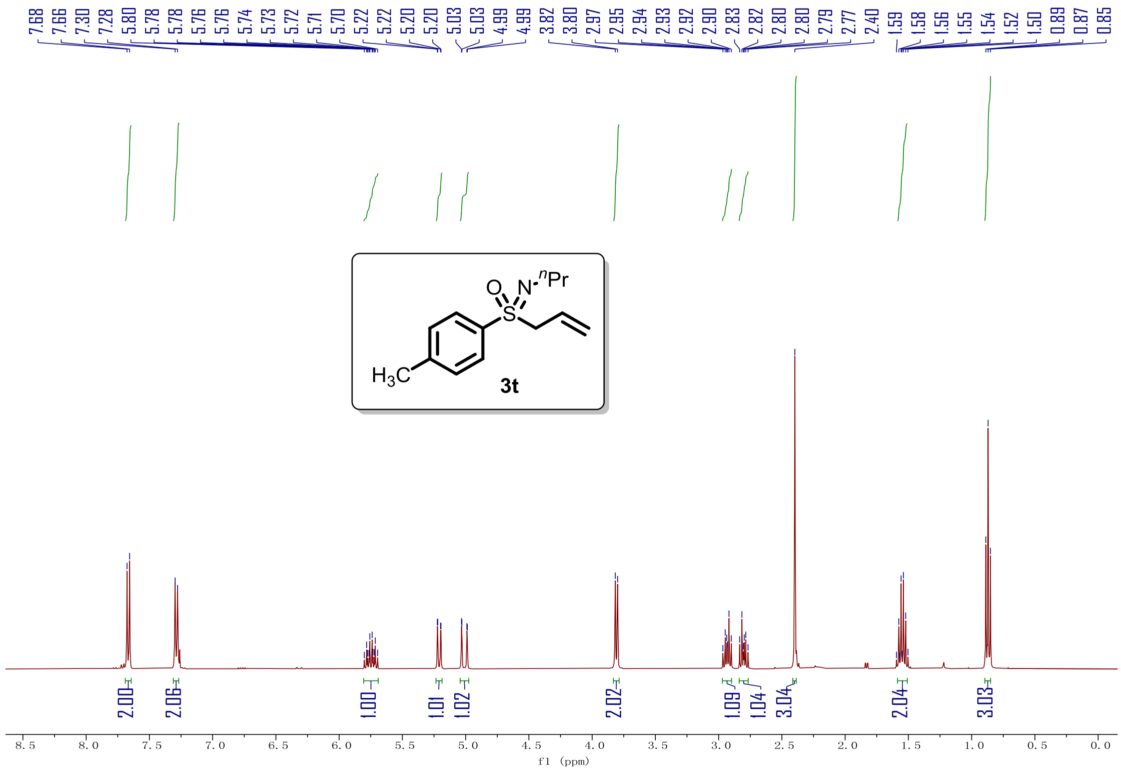

Supplementary Figure 49. ^{1}H NMR (400 MHz, $CDCl_3$) spectra of compound **3t.**

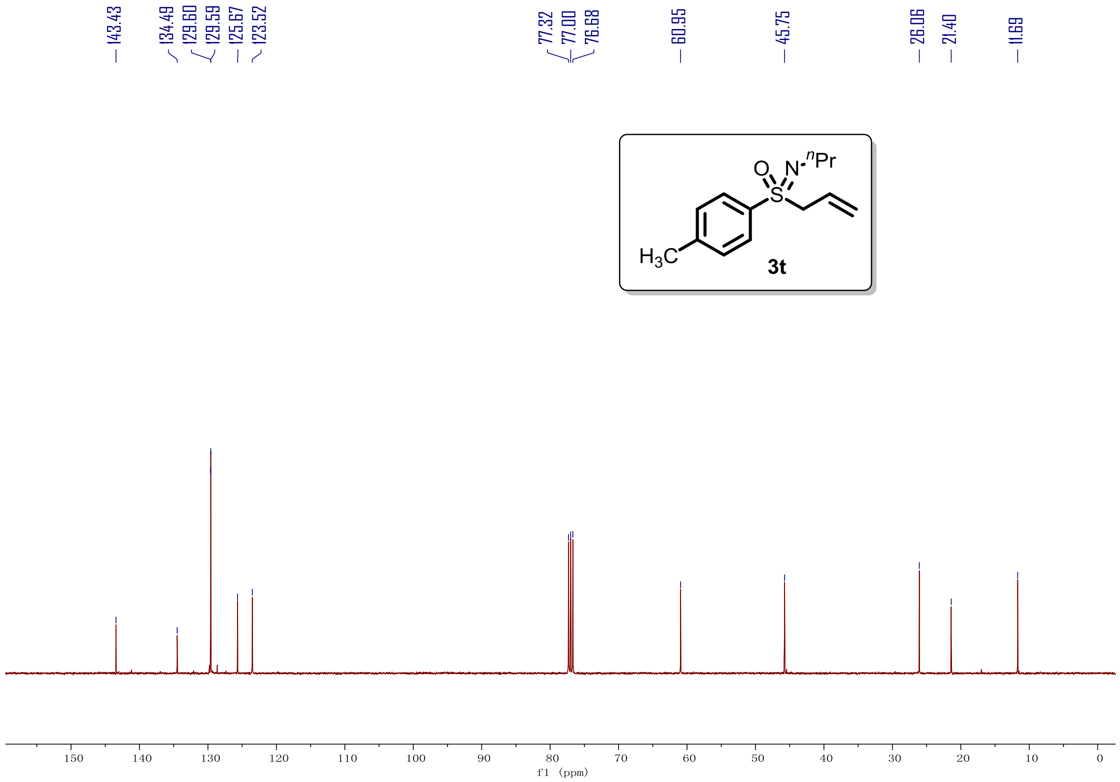

Supplementary Figure 50. ^{13}C NMR (101 MHz, $CDCl_3$) spectra of compound **3t.**

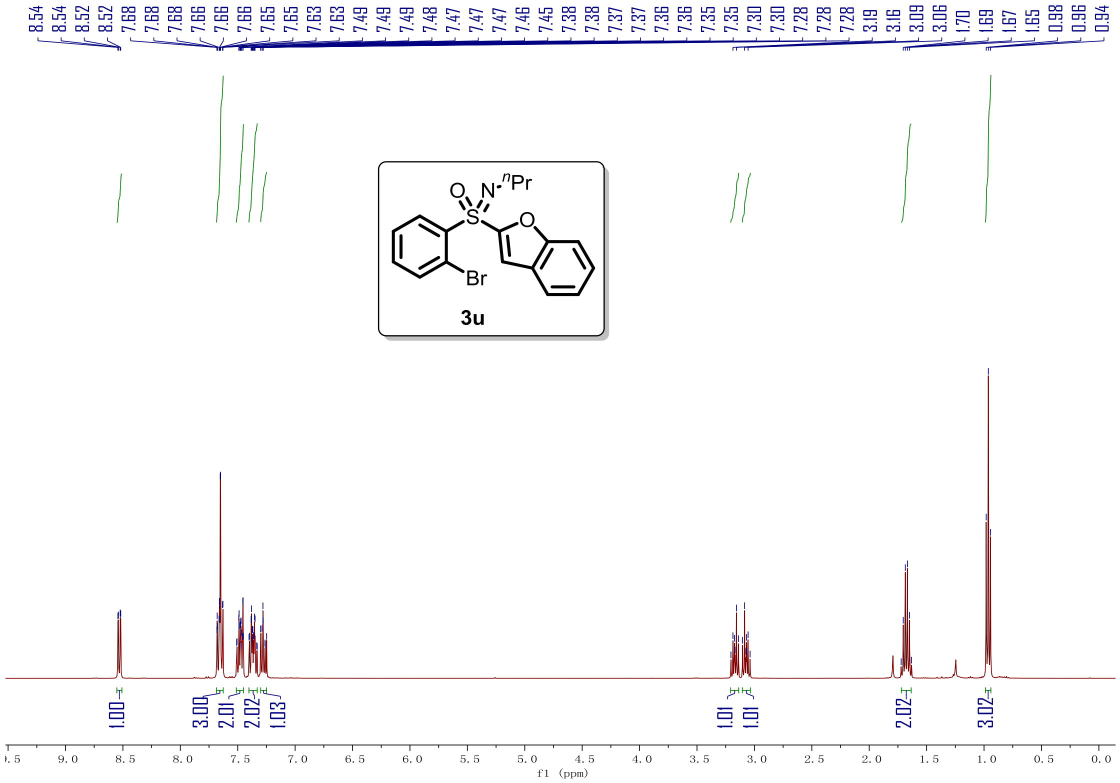

Supplementary Figure 51. ^{1}H NMR (400 MHz, $CDCl_3$) spectra of compound **3u.**

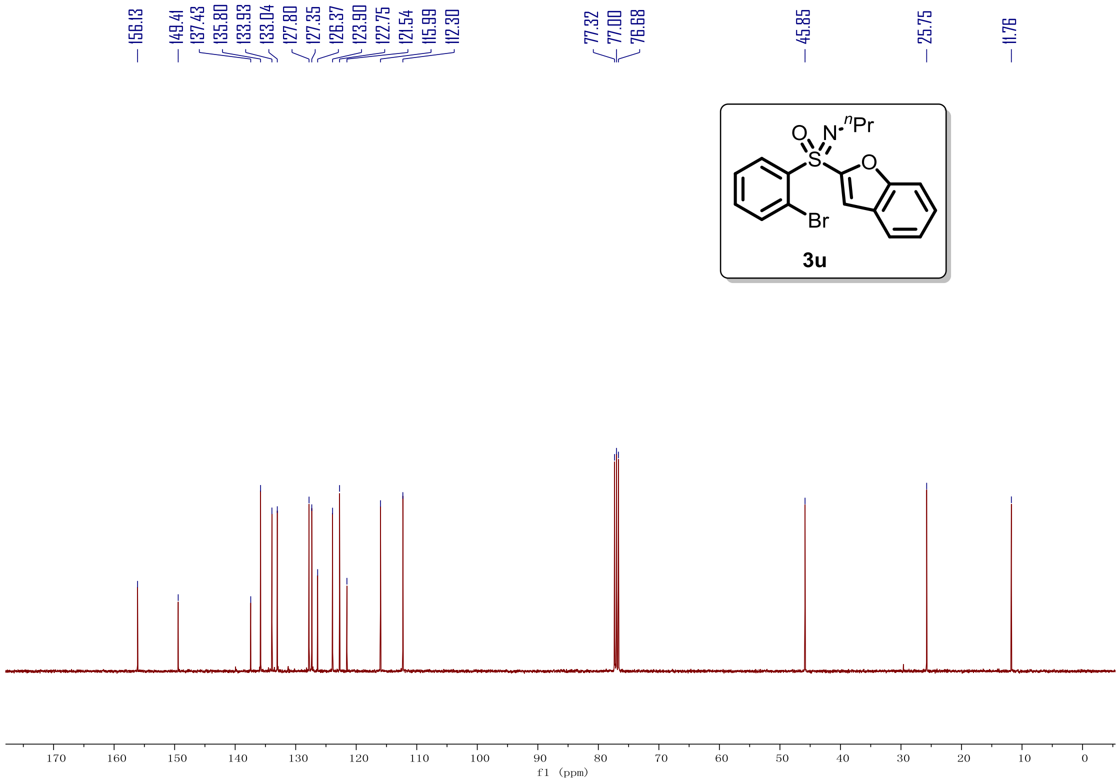

Supplementary Figure 52. ^{13}C NMR (101 MHz, $CDCl_3$) spectra of compound **3u.**

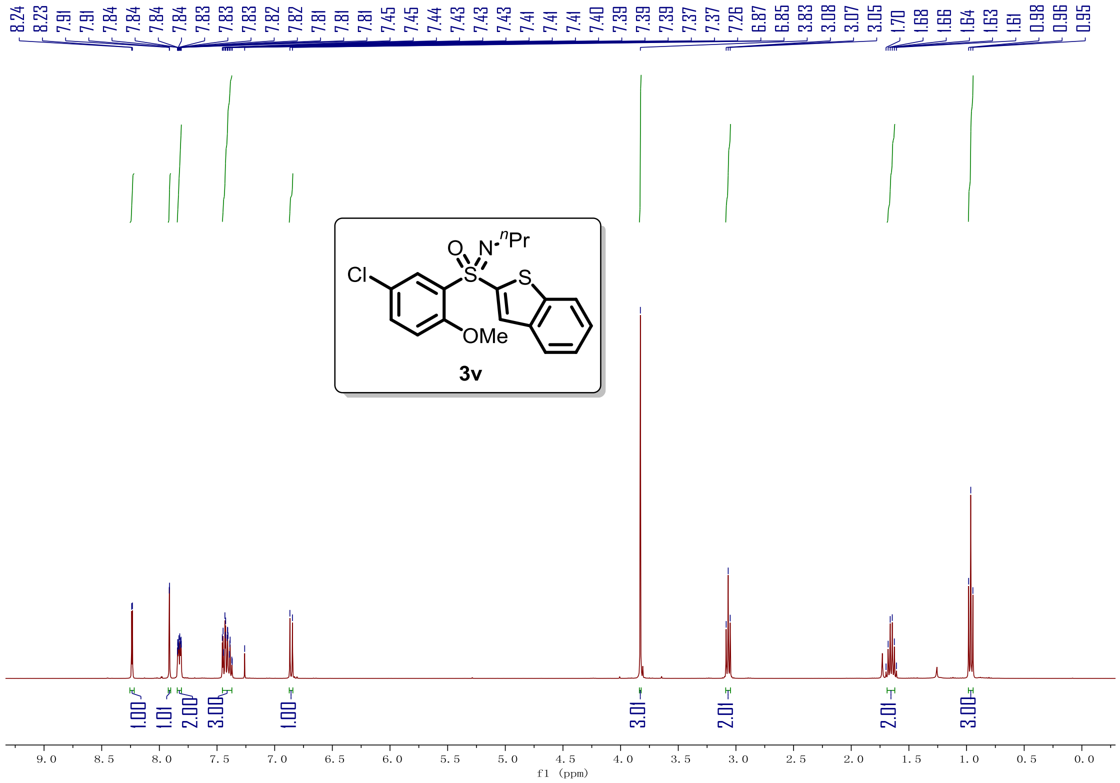

Supplementary Figure 53. ^{1}H NMR (400 MHz, $CDCl_3$) spectra of compound **3v.**

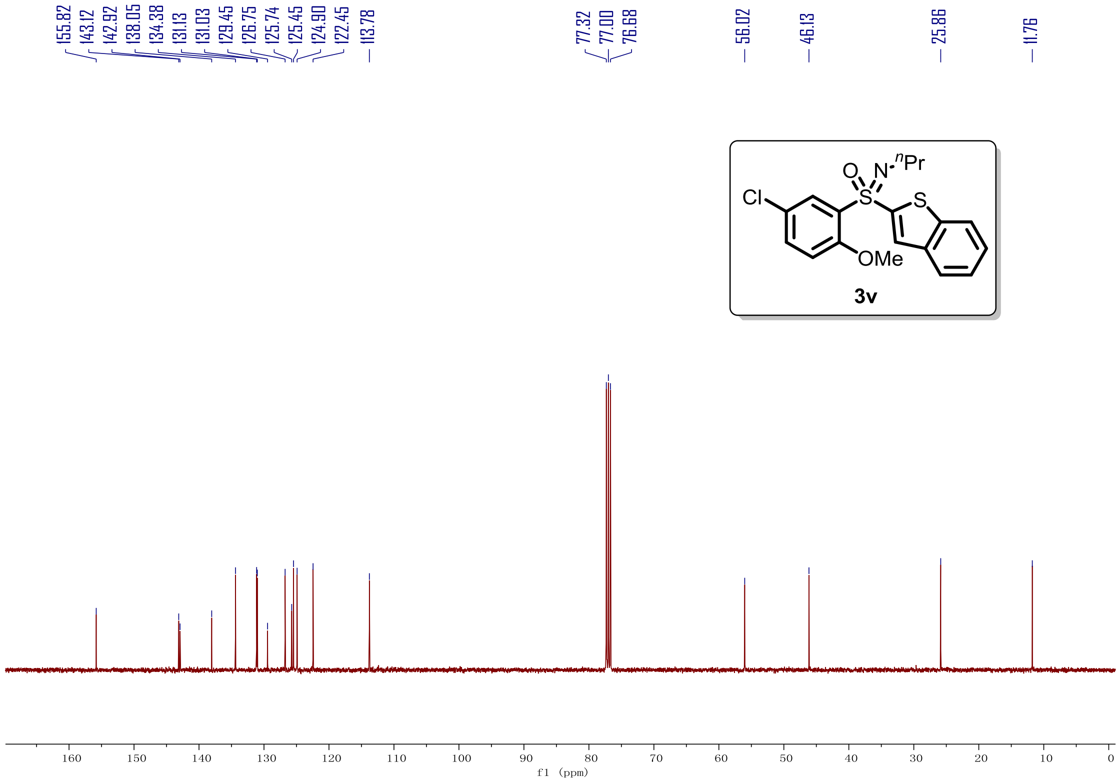

Supplementary Figure 54. ^{13}C NMR (101 MHz, $CDCl_3$) spectra of compound **3v.**

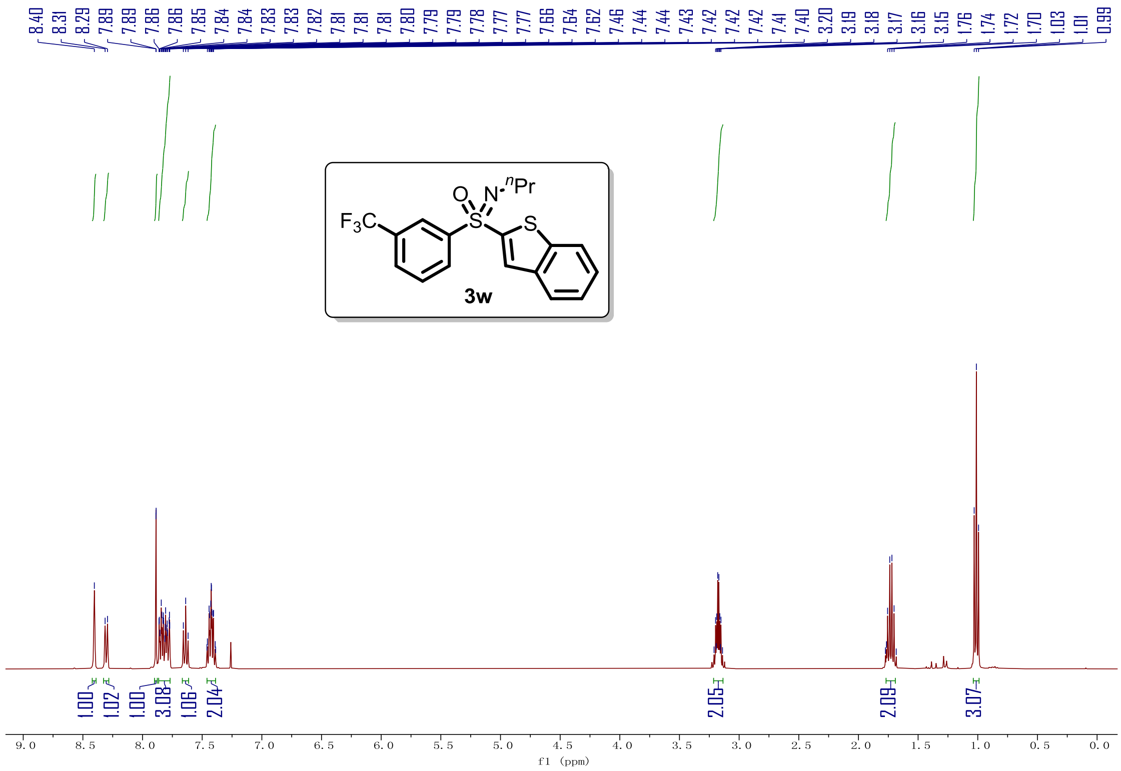

Supplementary Figure 55. ^{1}H NMR (400 MHz, $CDCl_3$) spectra of compound **3w.**

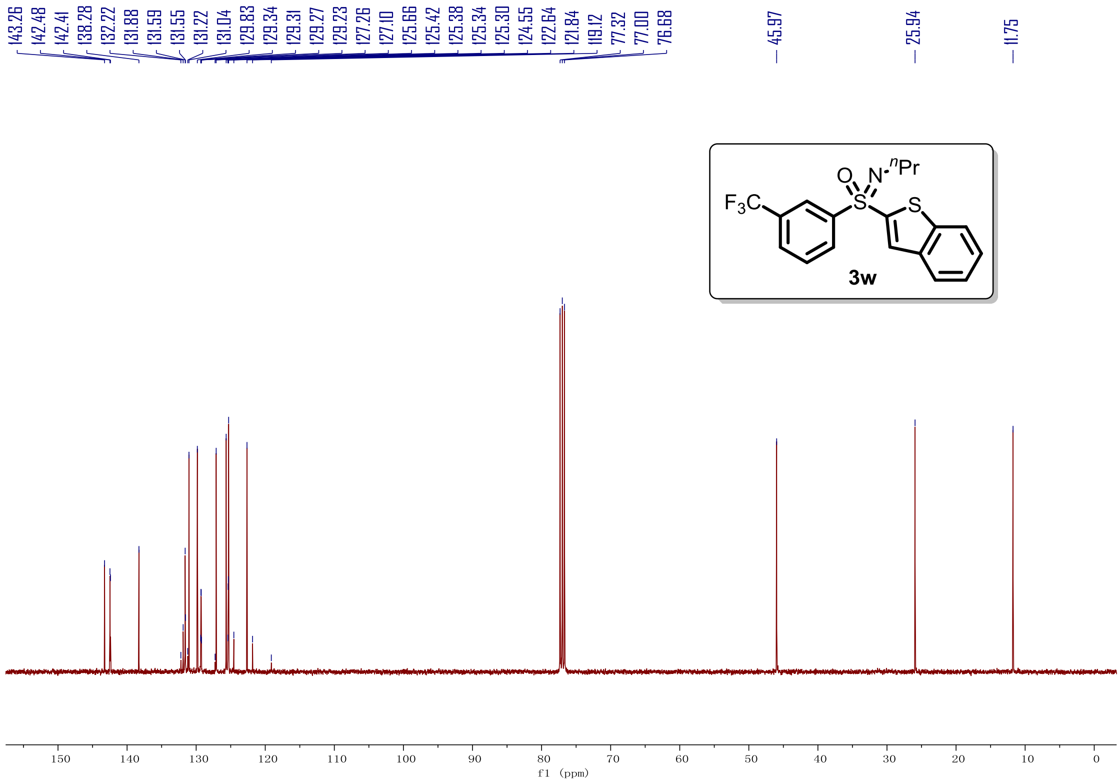

Supplementary Figure 56. ^{13}C NMR (101 MHz, $CDCl_3$) spectra of compound **3w.**

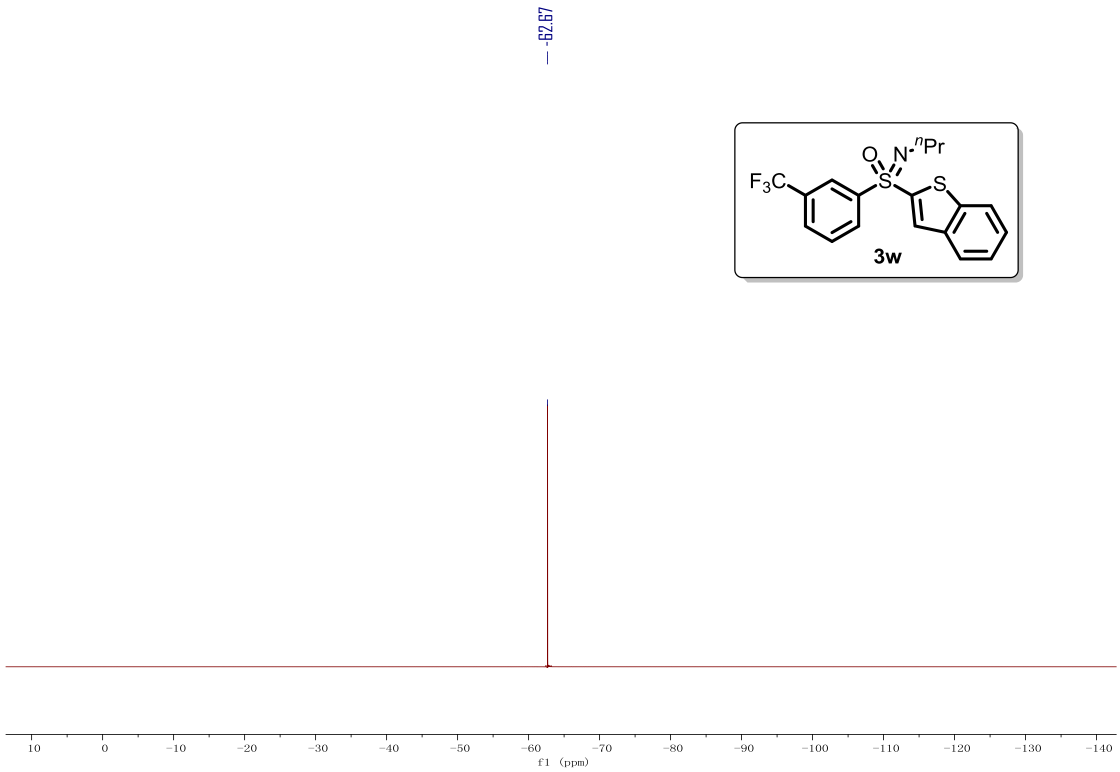

Supplementary Figure 57. ^{19}F NMR (376 MHz, $CDCl_3$) spectra of compound **3w.**

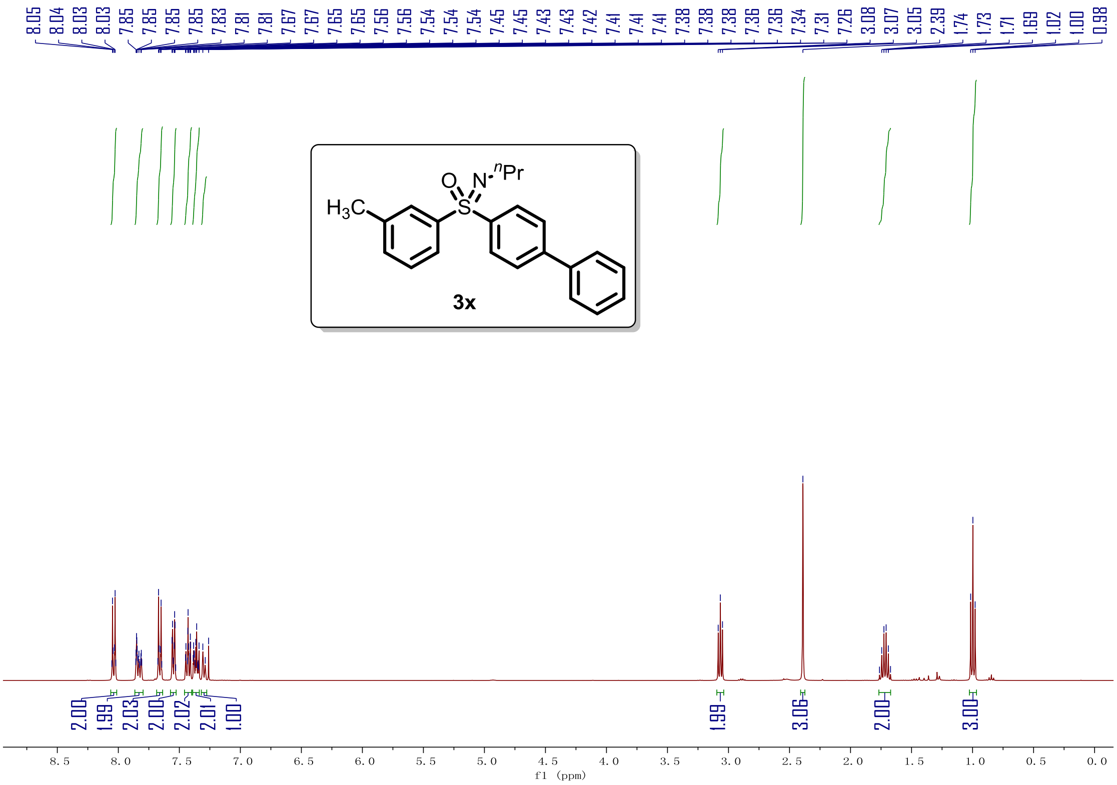

Supplementary Figure 58. ^{1}H NMR (400 MHz, $CDCl_3$) spectra of compound **3x.**

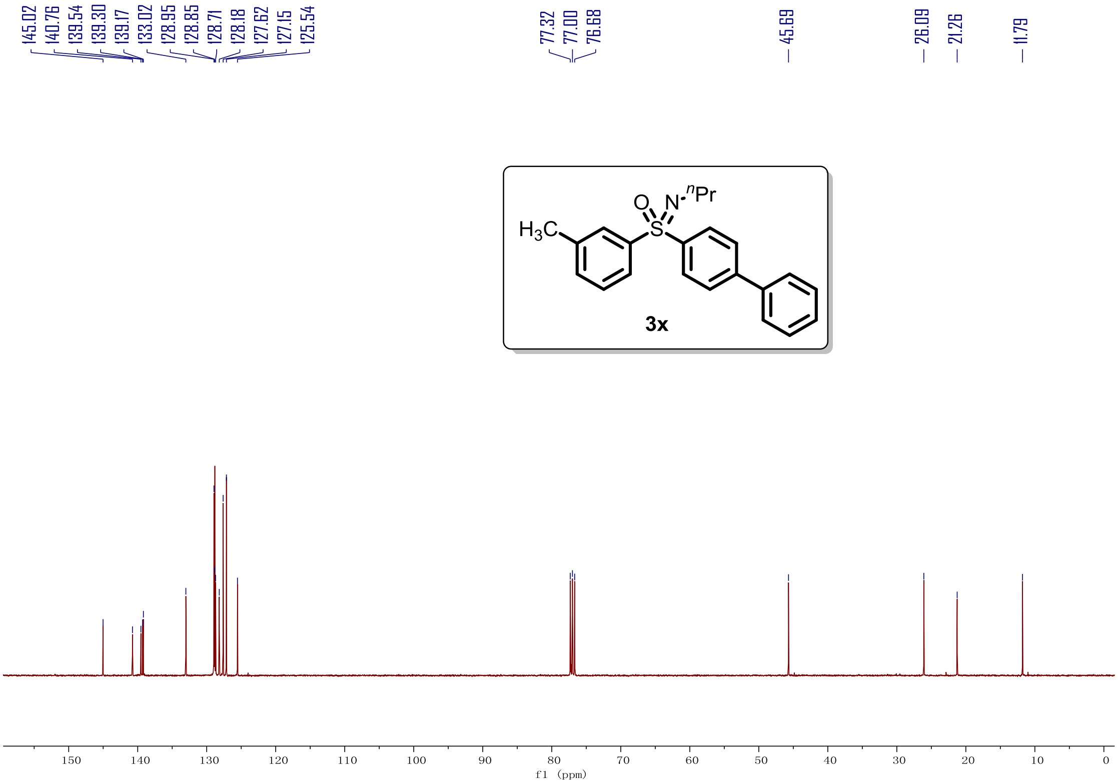

Supplementary Figure 59. ^{13}C NMR (101 MHz, $CDCl_3$) spectra of compound **3x.**

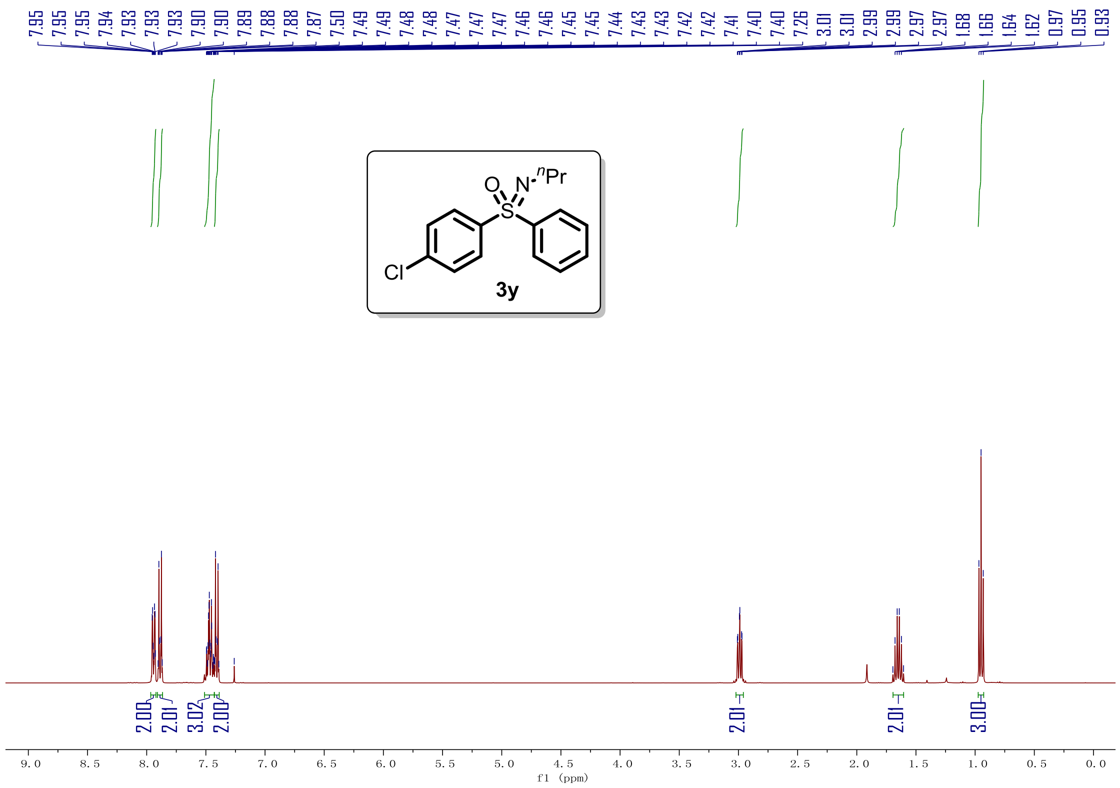

Supplementary Figure 60. ^{1}H NMR (400 MHz, $CDCl_3$) spectra of compound **3y.**

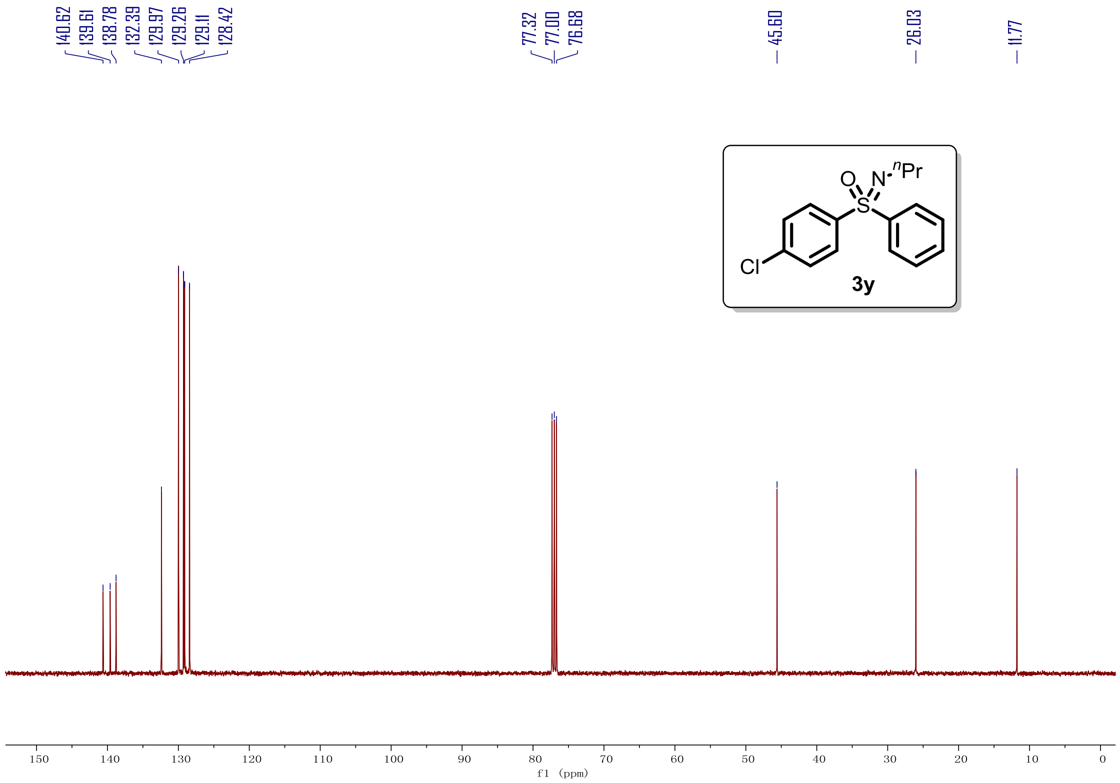

Supplementary Figure 61. ^{13}C NMR (101 MHz, $CDCl_3$) spectra of compound **3y.**

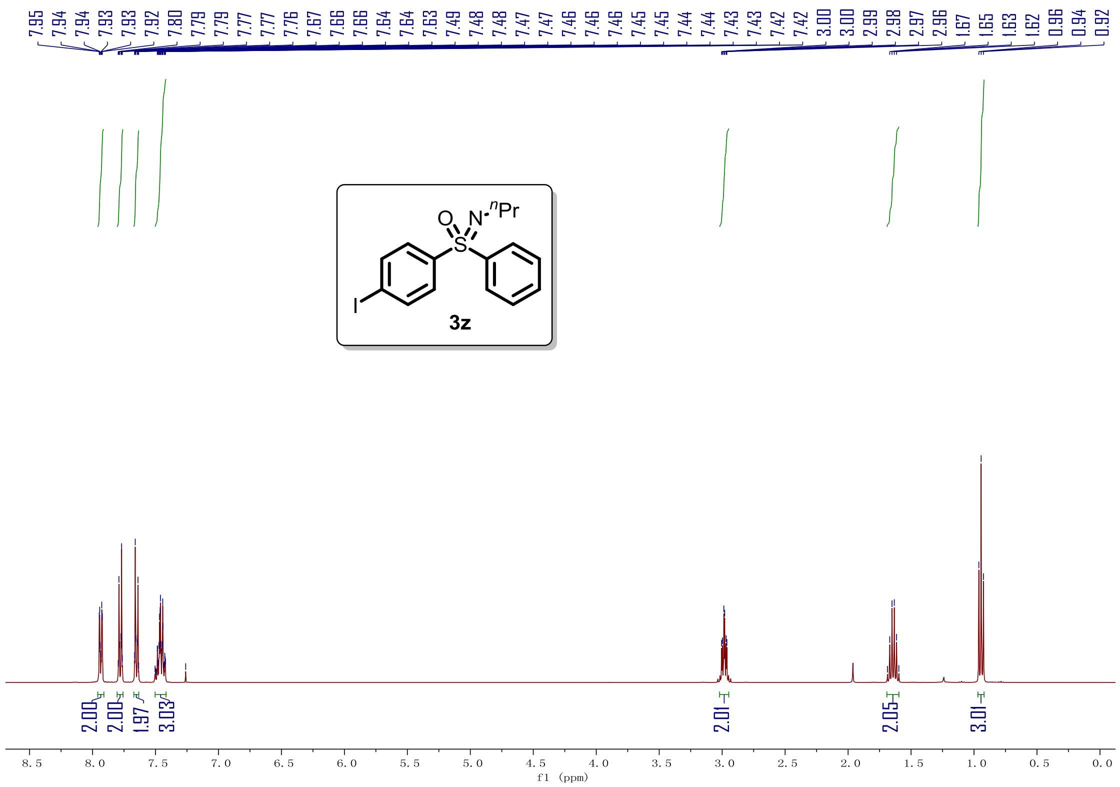

Supplementary Figure 62. ^{1}H NMR (400 MHz, $CDCl_3$) spectra of compound **3z.**

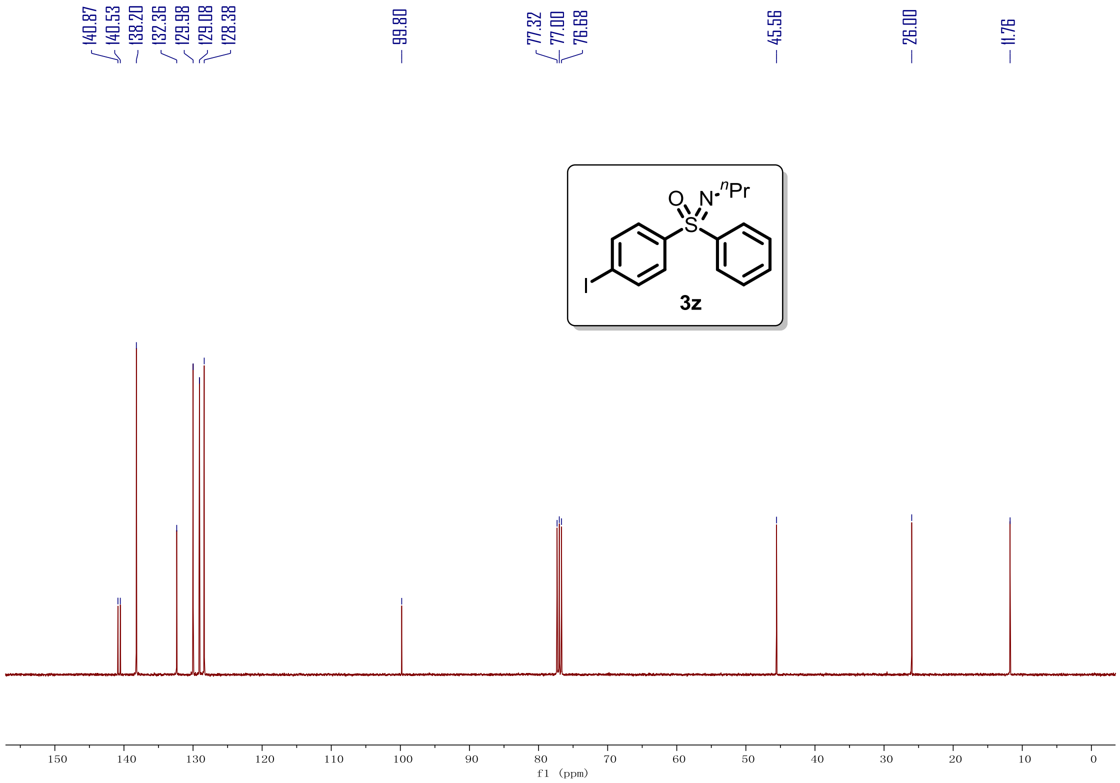

Supplementary Figure 63. ^{13}C NMR (101 MHz, $CDCl_3$) spectra of compound **3z.**

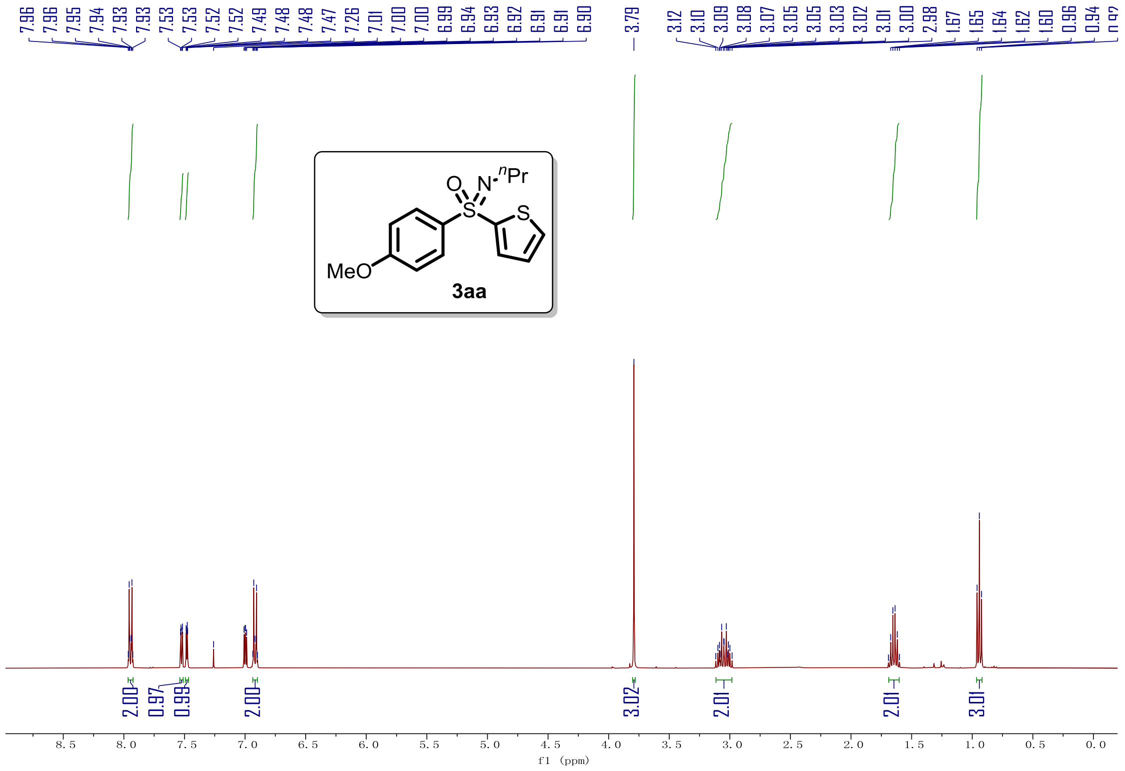

Supplementary Figure 64. ^{1}H NMR (400 MHz, $CDCl_3$) spectra of compound **3aa.**

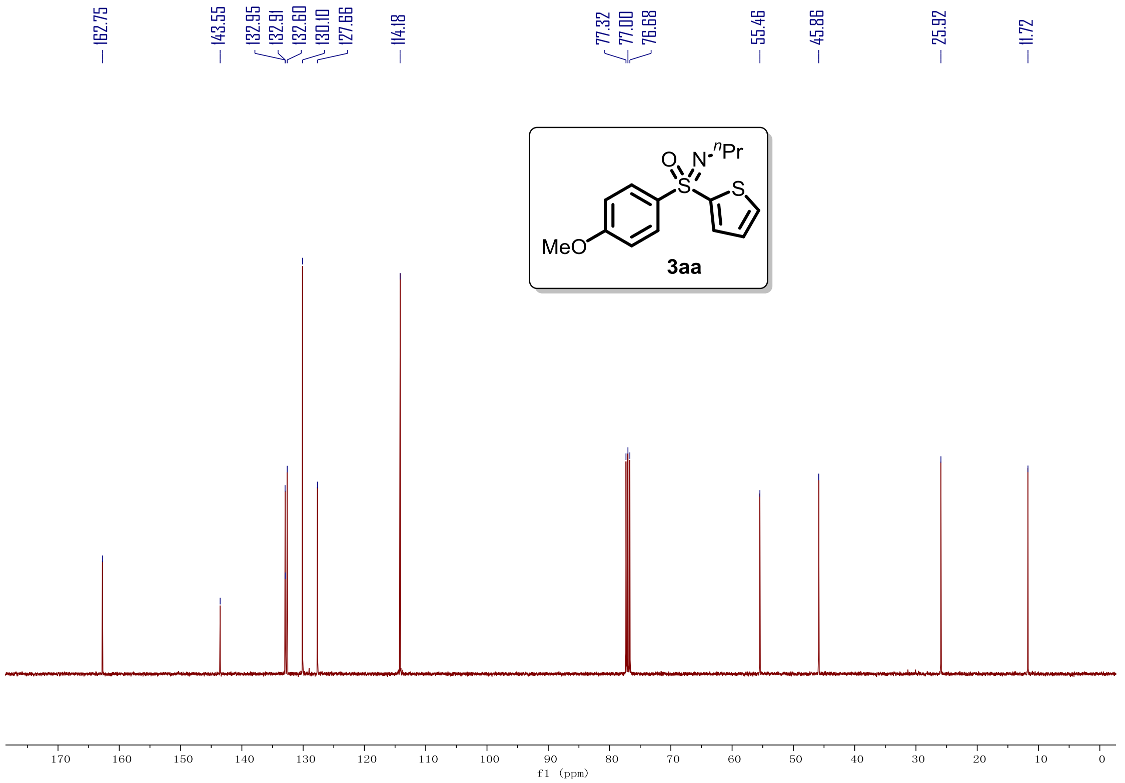

Supplementary Figure 65. ^{13}C NMR (101 MHz, $CDCl_3$) spectra of compound **3aa.**

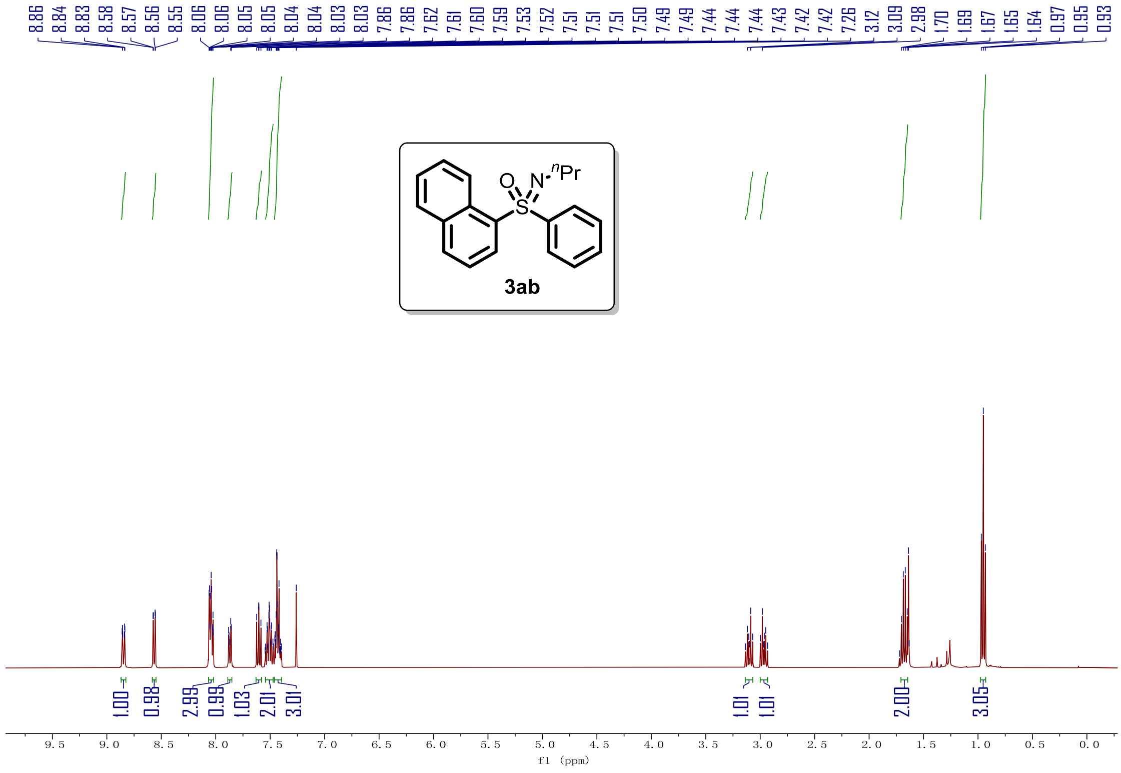

Supplementary Figure 66. ^{1}H NMR (400 MHz, $CDCl_3$) spectra of compound **3ab.**

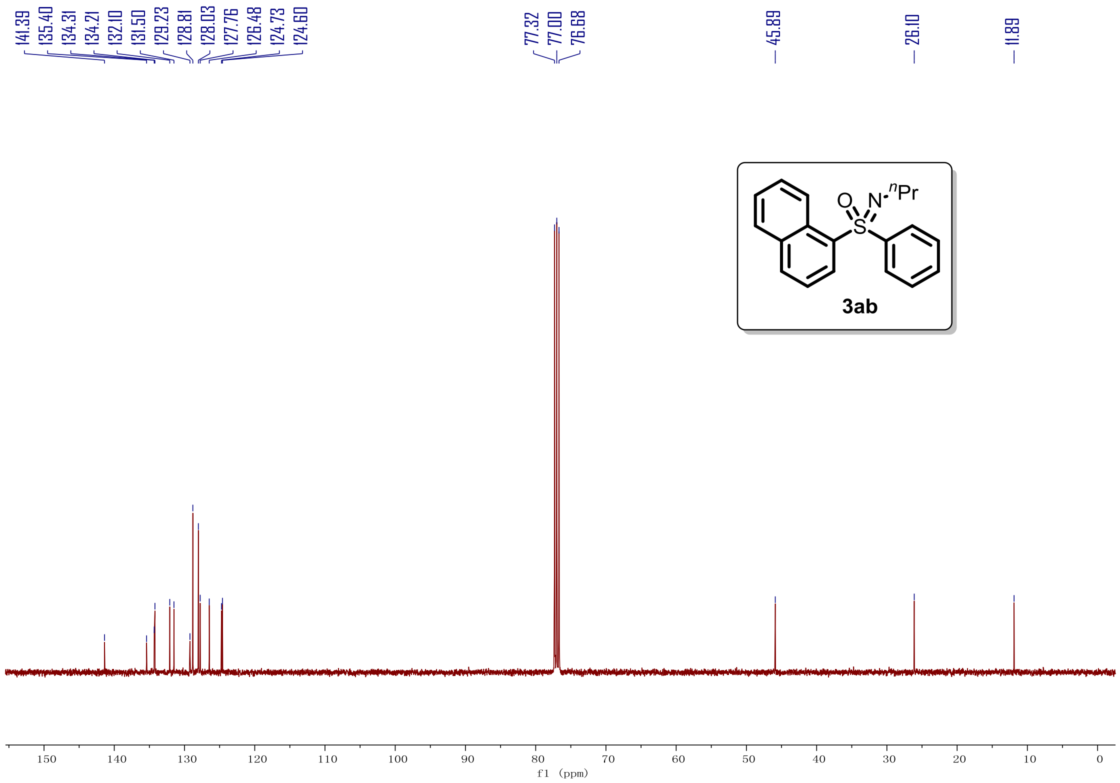

Supplementary Figure 67. ^{13}C NMR (101 MHz, $CDCl_3$) spectra of compound **3ab.**

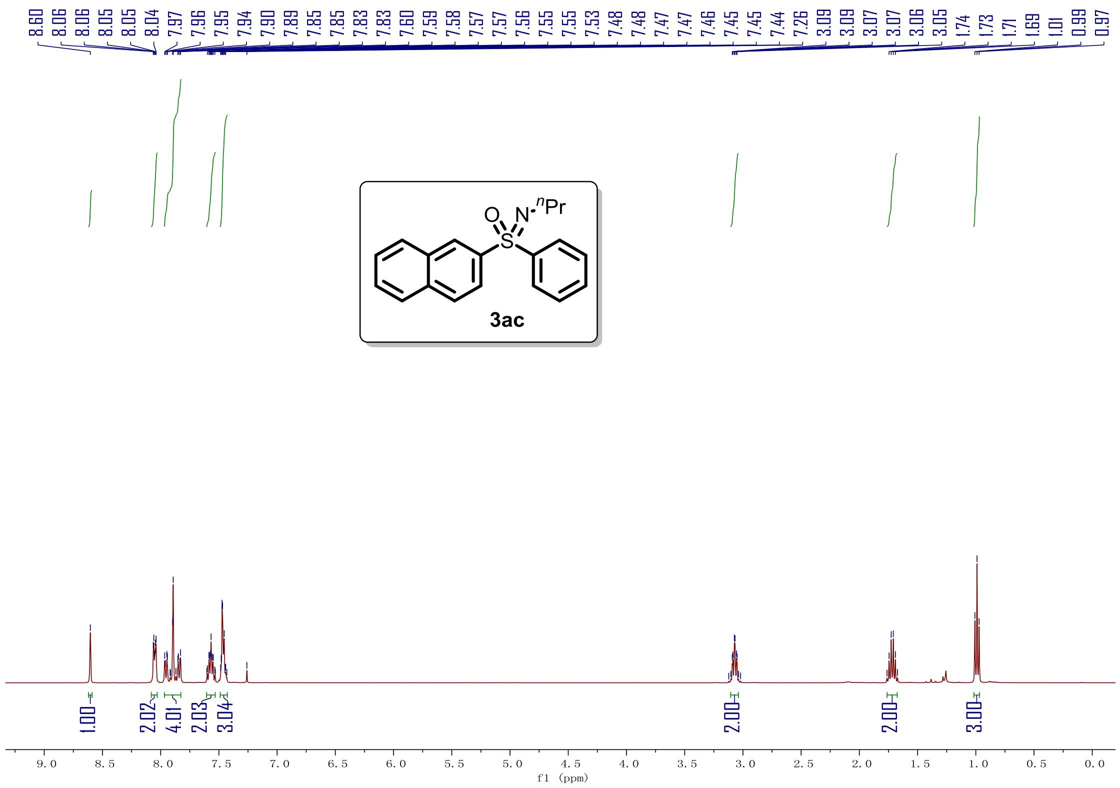

Supplementary Figure 68. ^{1}H NMR (400 MHz, $CDCl_3$) spectra of compound **3ac.**

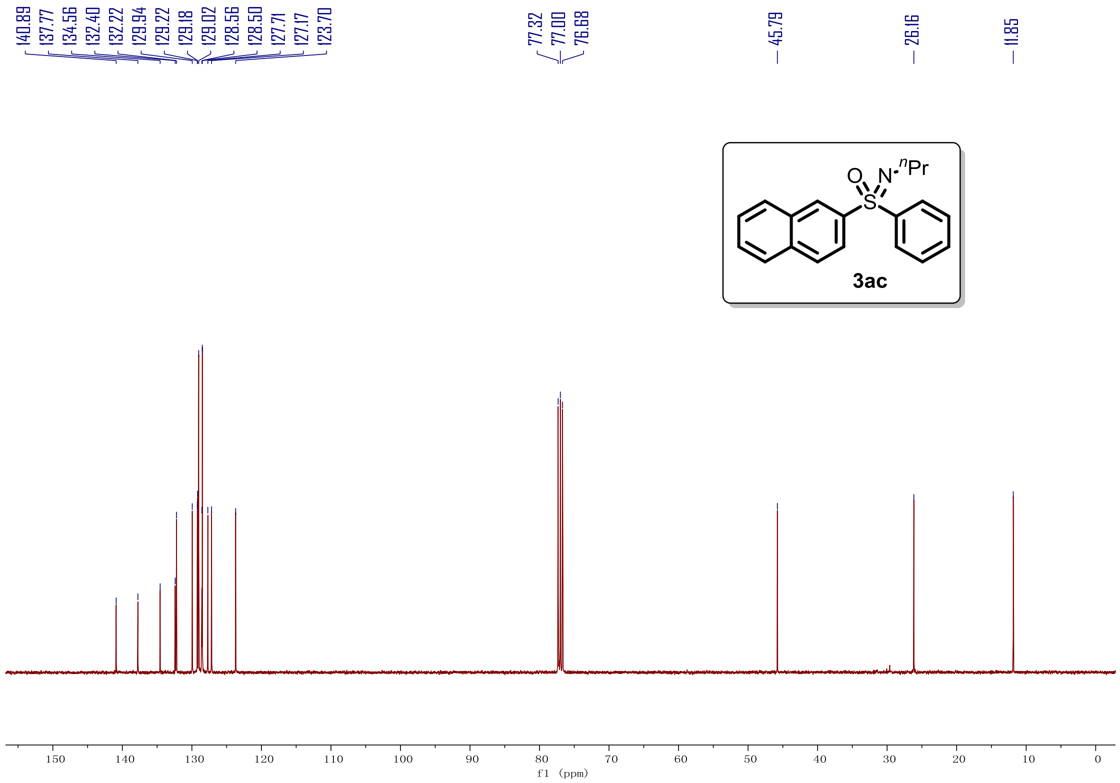

Supplementary Figure 69. ^{13}C NMR (101 MHz, $CDCl_3$) spectra of compound **3ac.**

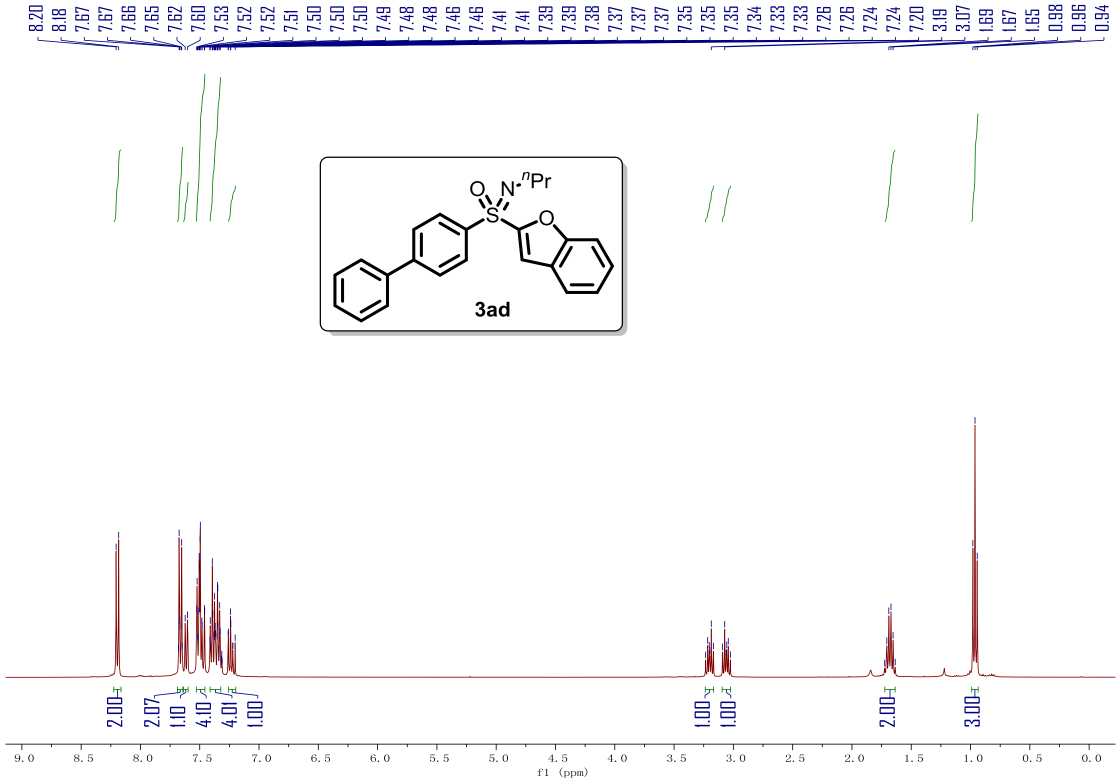

Supplementary Figure 70. ^{1}H NMR (400 MHz, $CDCl_3$) spectra of compound **3ad.**

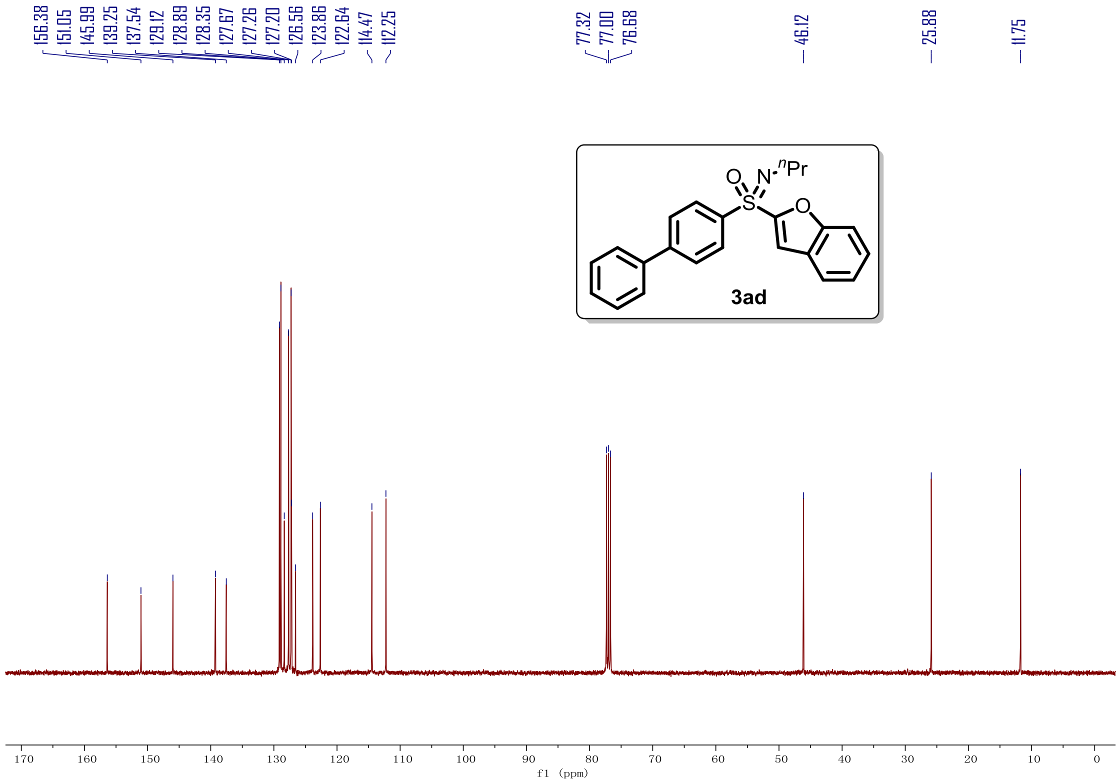

Supplementary Figure 71. ^{13}C NMR (101 MHz, $CDCl_3$) spectra of compound **3ad.**

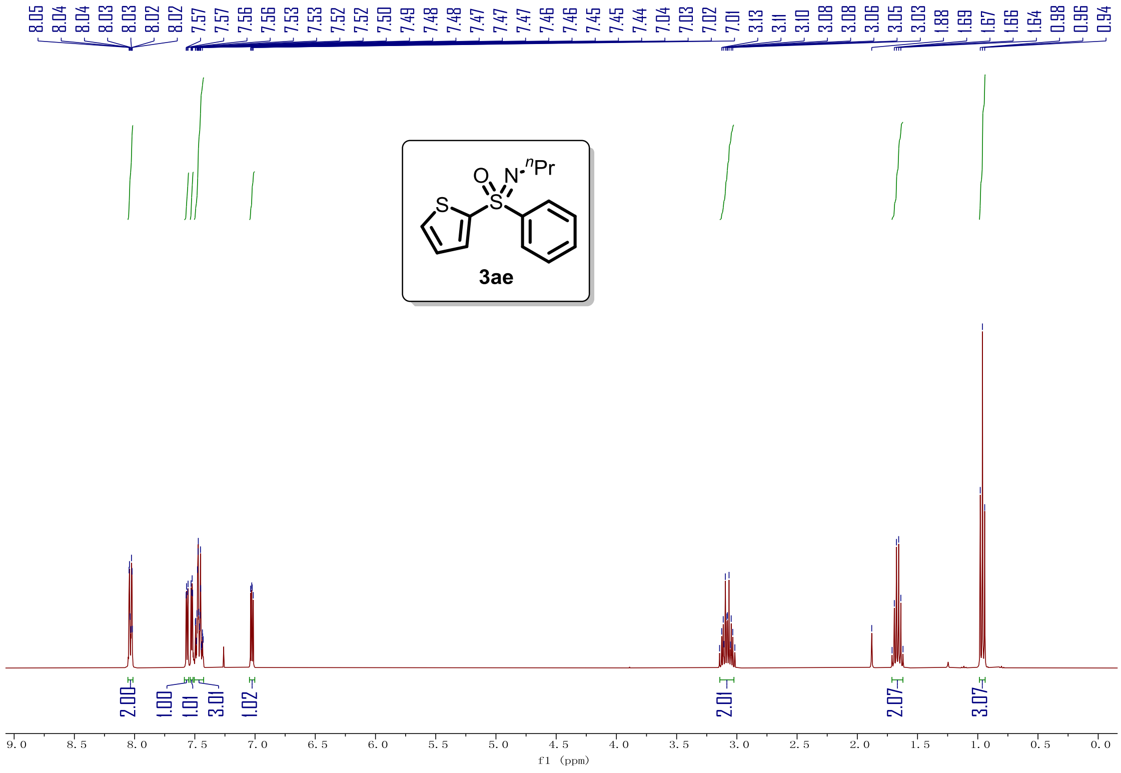

Supplementary Figure 72. ^{1}H NMR (400 MHz, $CDCl_3$) spectra of compound **3ae.**

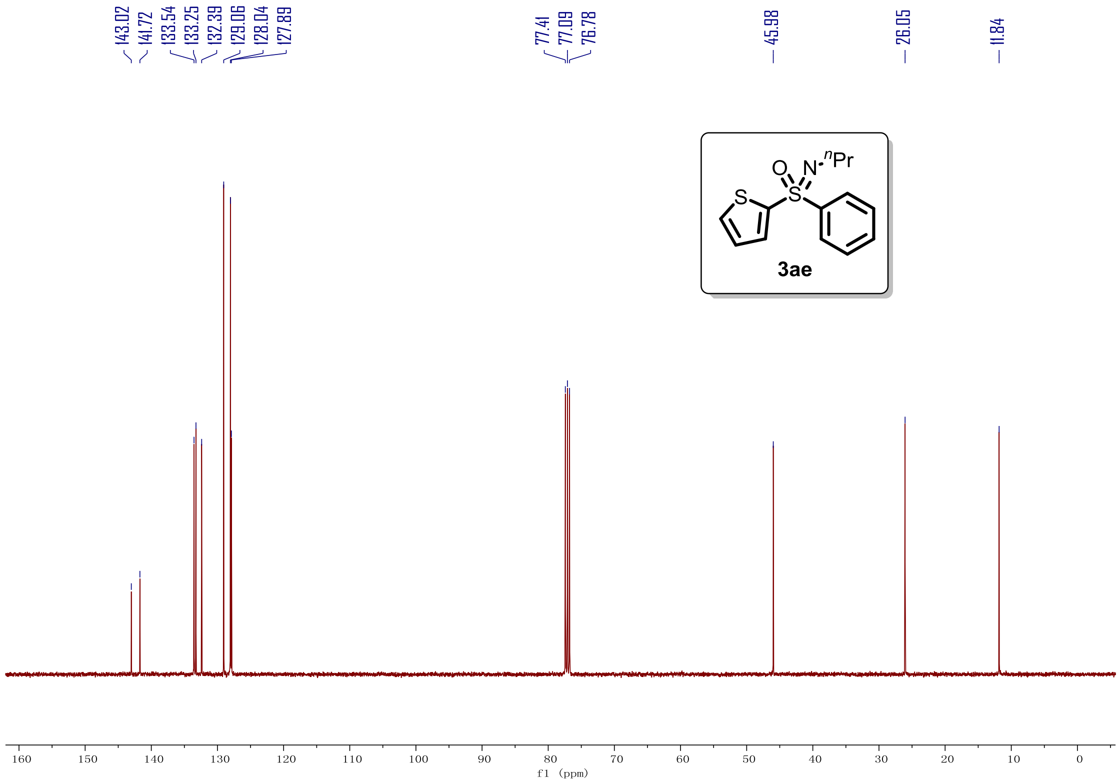

Supplementary Figure 73. ^{13}C NMR (101 MHz, $CDCl_3$) spectra of compound **3ae.**

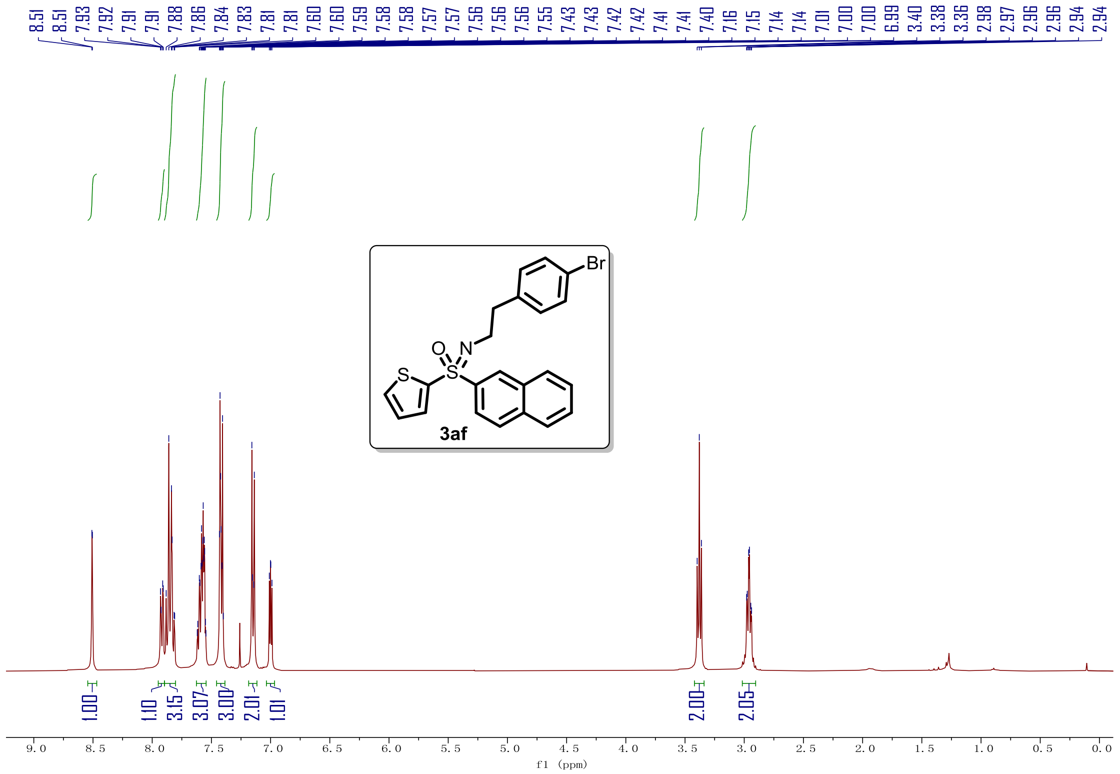

Supplementary Figure 74. ^{1}H NMR (400 MHz, $CDCl_3$) spectra of compound **3af.**

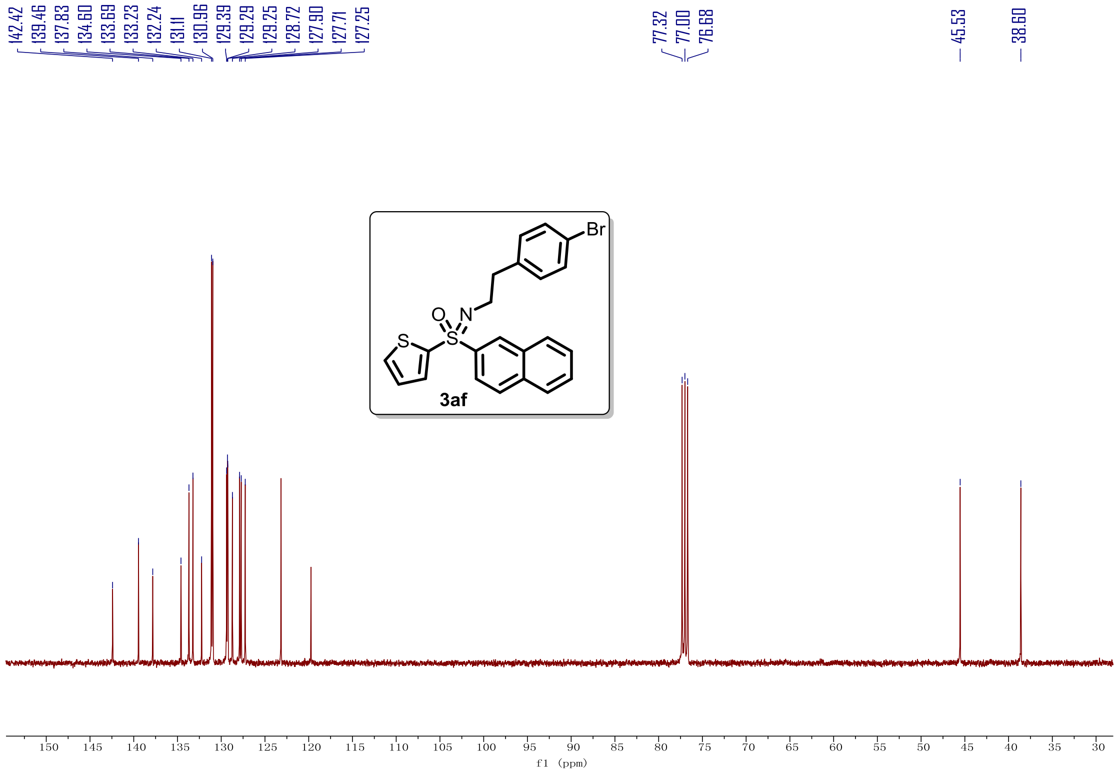

Supplementary Figure 75. ^{13}C NMR (101 MHz, $CDCl_3$) spectra of compound **3af.**

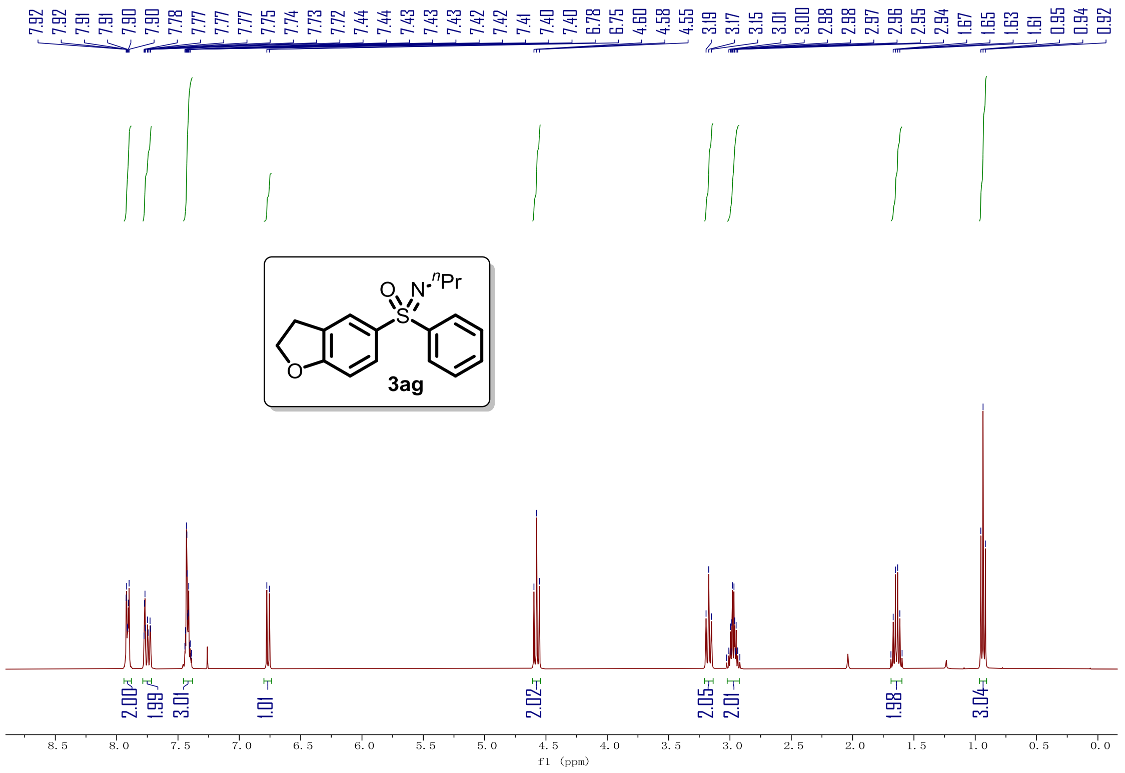

Supplementary Figure 76. ^{1}H NMR (400 MHz, $CDCl_3$) spectra of compound **3ag.**

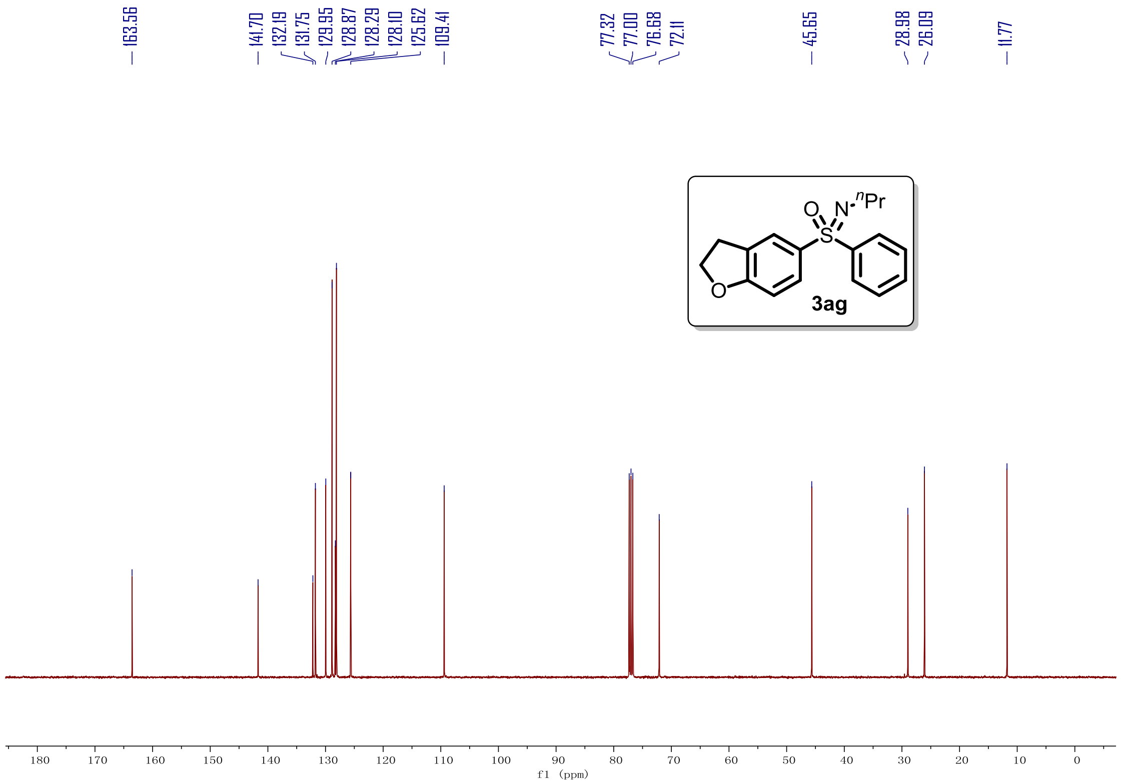

Supplementary Figure 77. ^{13}C NMR (101 MHz, $CDCl_3$) spectra of compound **3ag.**

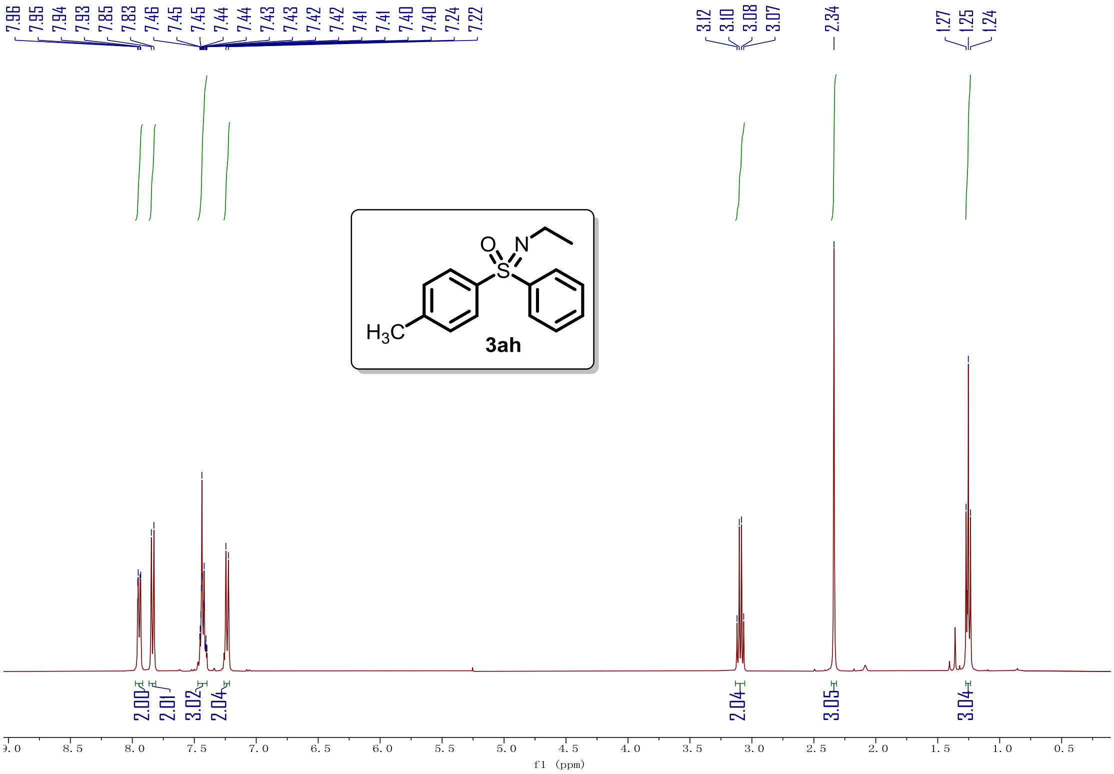

Supplementary Figure 78. ^{1}H NMR (400 MHz, $CDCl_3$) spectra of compound **3ah.**

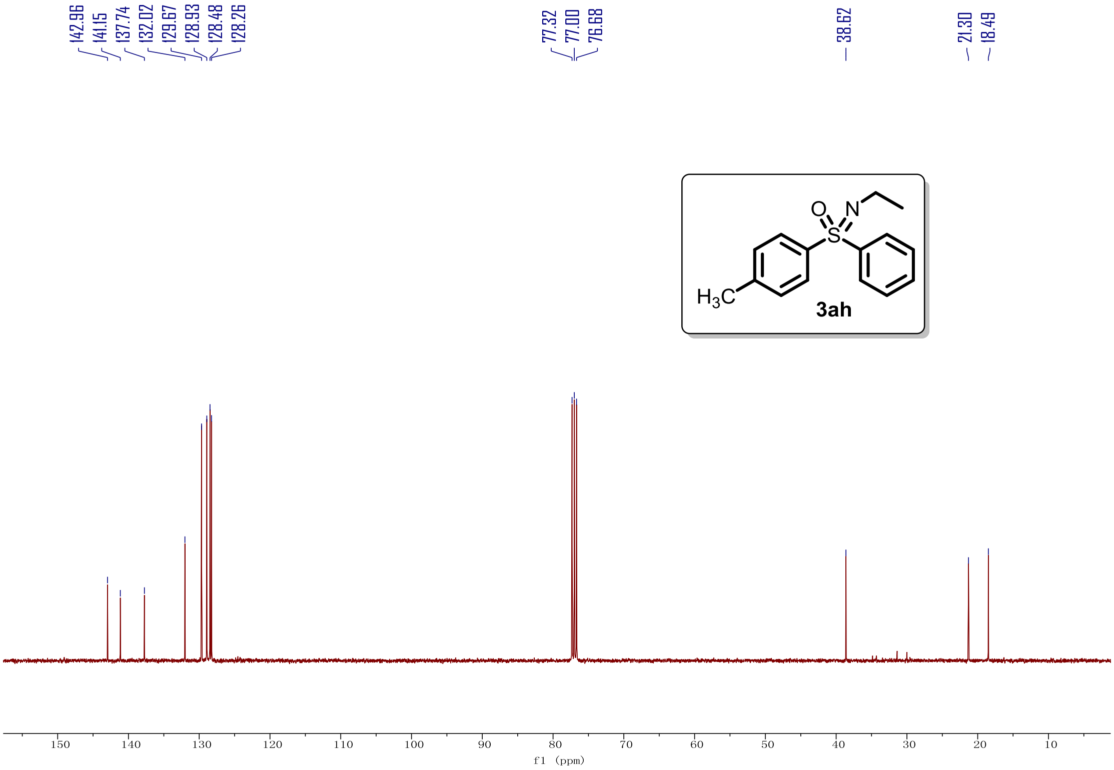

Supplementary Figure 79. ^{13}C NMR (101 MHz, $CDCl_3$) spectra of compound **3ah.**

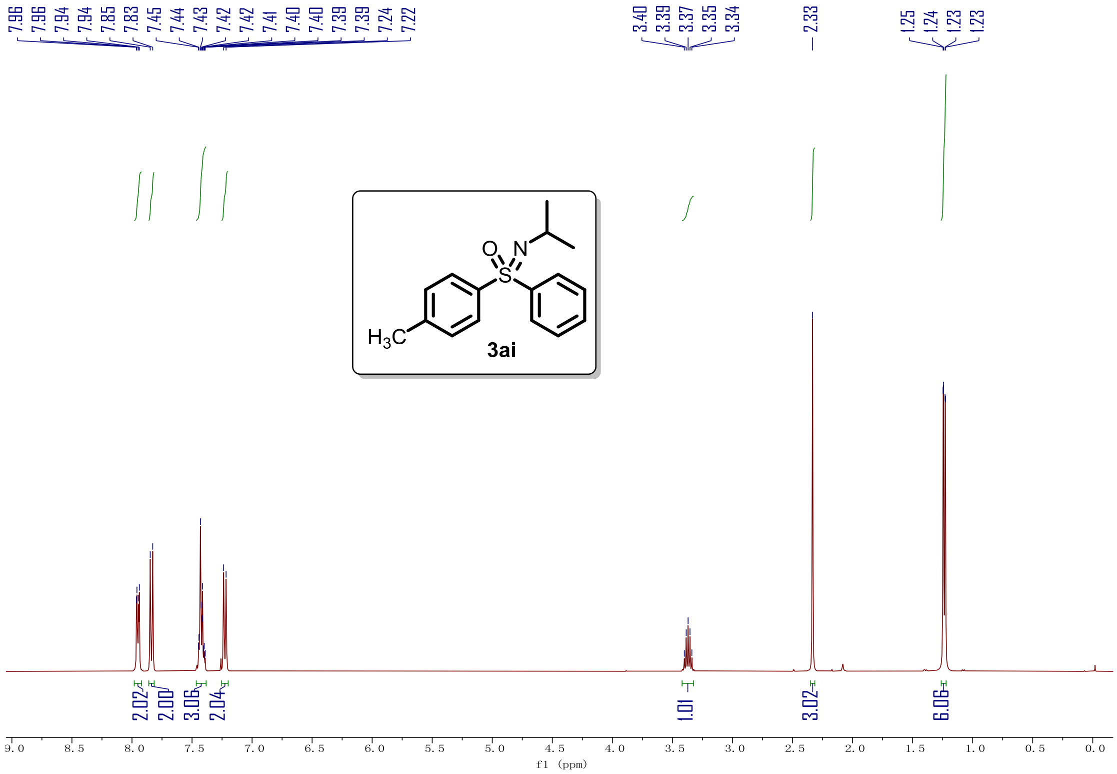

Supplementary Figure 80. ^{1}H NMR (400 MHz, $CDCl_3$) spectra of compound **3ai.**

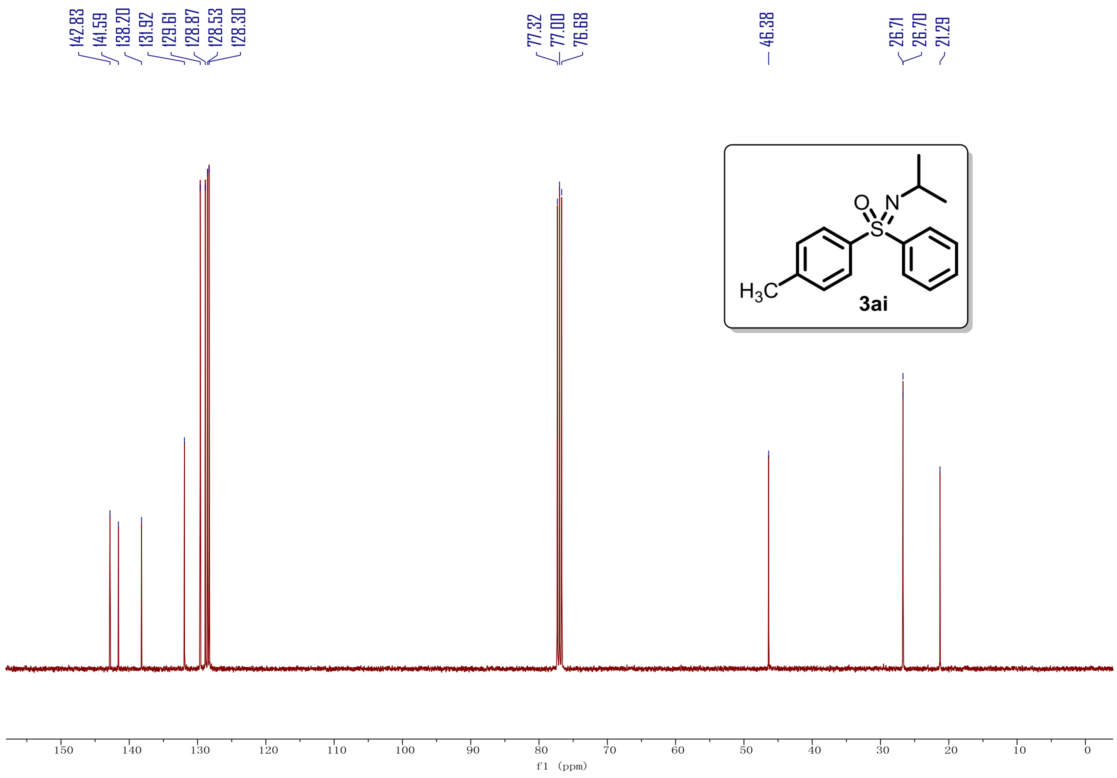

Supplementary Figure 81. ^{13}C NMR (101 MHz, $CDCl_3$) spectra of compound **3ai.**

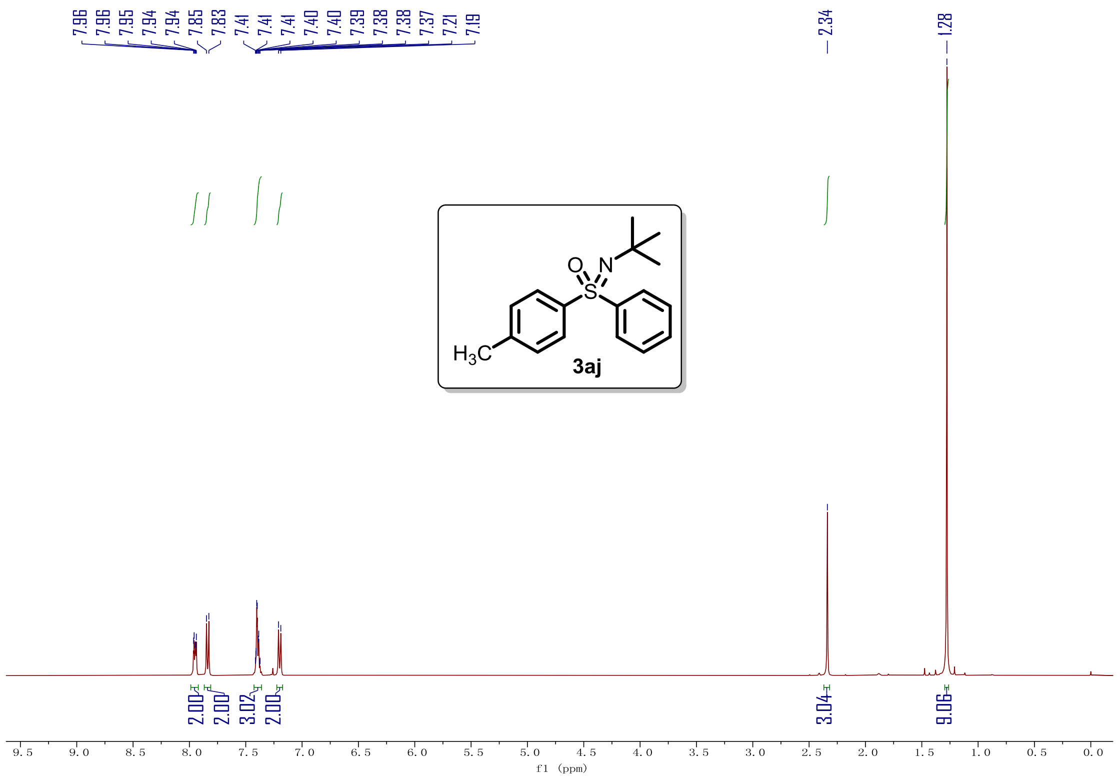

Supplementary Figure 82. ^{1}H NMR (400 MHz, $CDCl_3$) spectra of compound **3aj.**

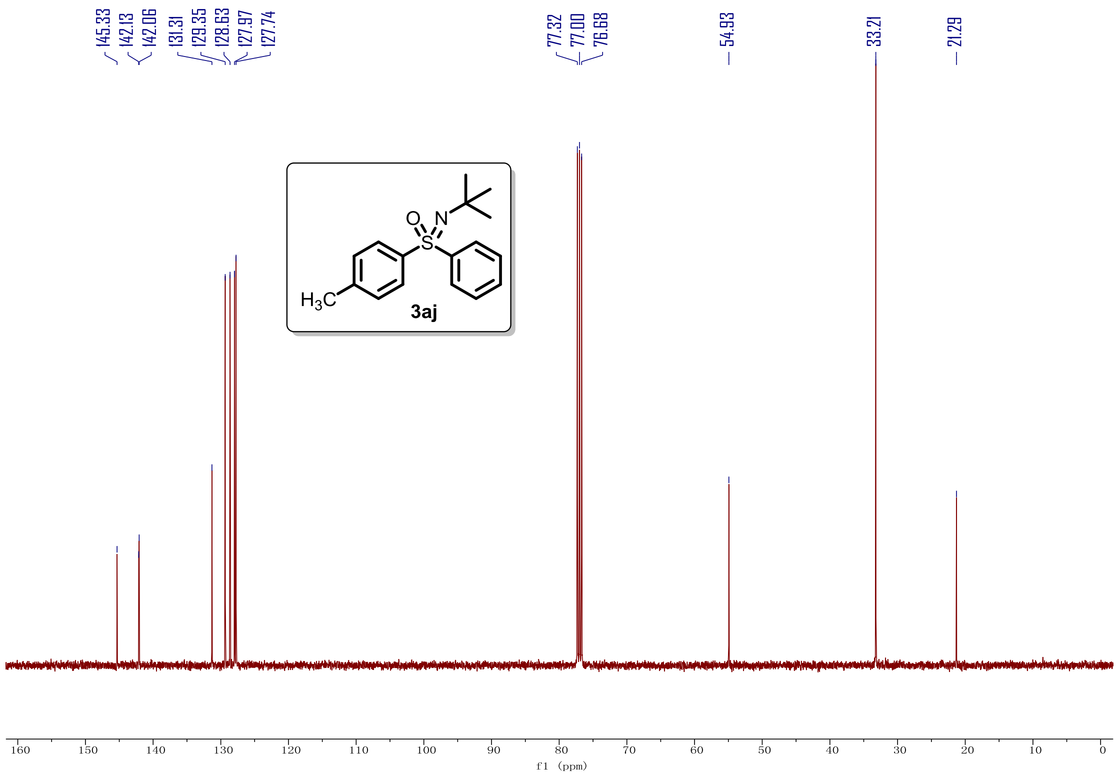

Supplementary Figure 83. ^{13}C NMR (101 MHz, $CDCl_3$) spectra of compound **3aj.**

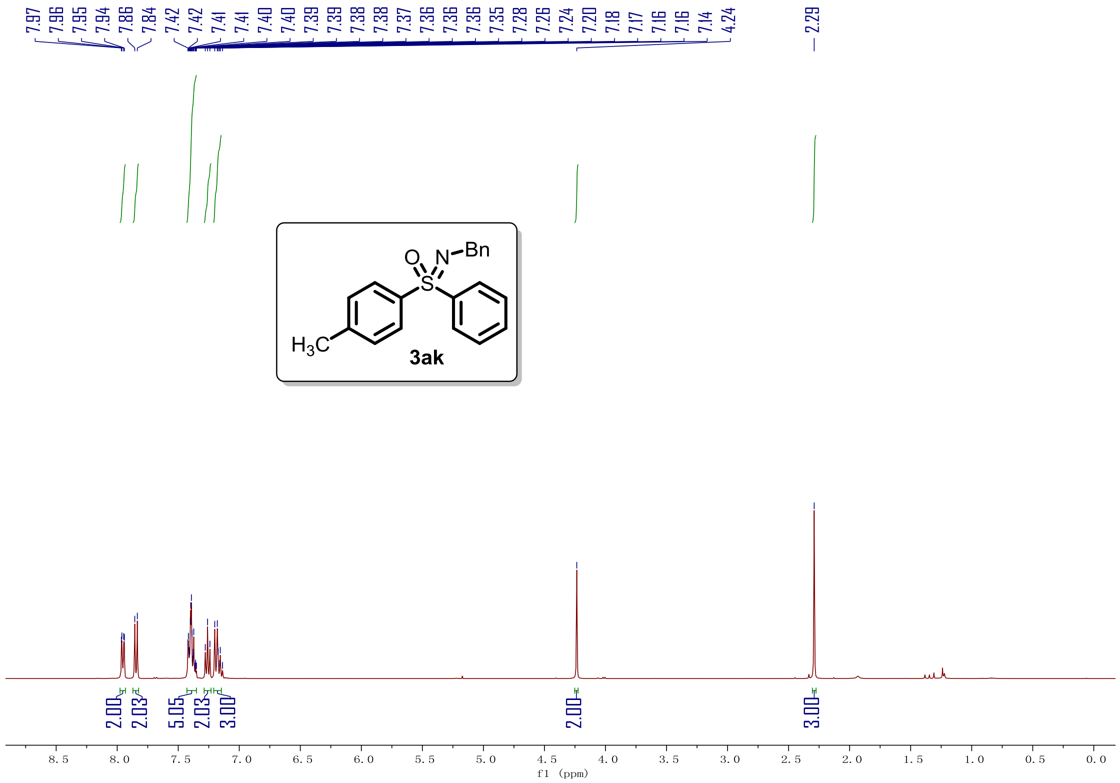

Supplementary Figure 84. ^{1}H NMR (400 MHz, $CDCl_3$) spectra of compound **3ak.**

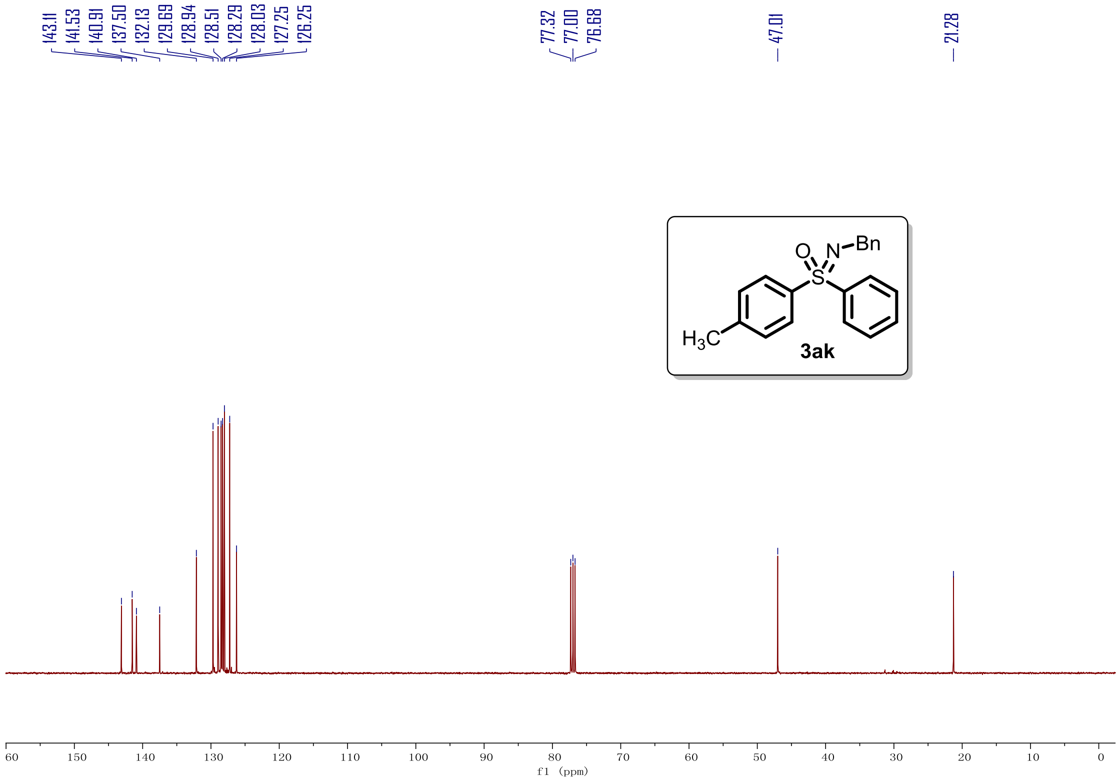

Supplementary Figure 85. ^{13}C NMR (101 MHz, $CDCl_3$) spectra of compound **3ak.**

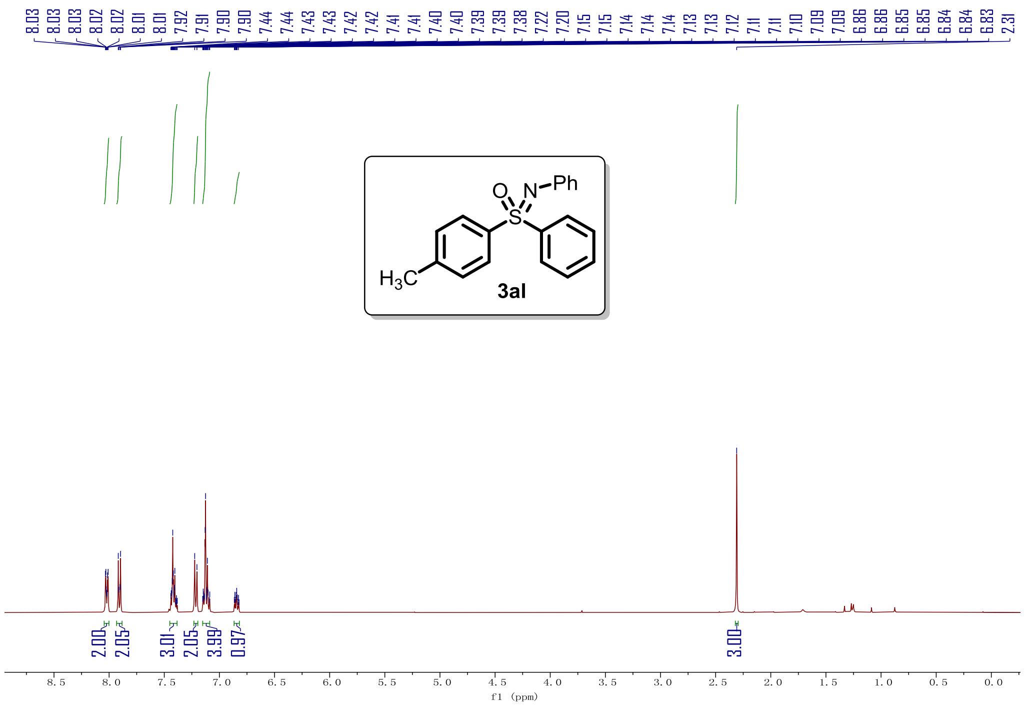

Supplementary Figure 86. ^{1}H NMR (400 MHz, $CDCl_3$) spectra of compound **3al.**

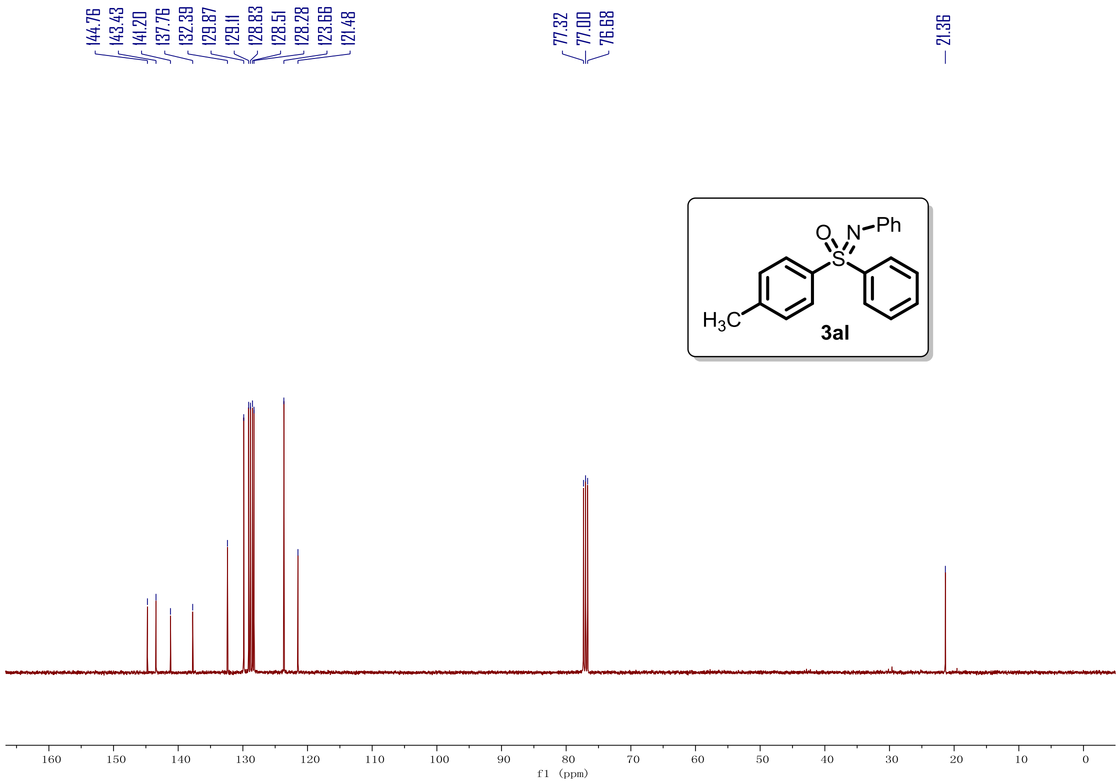

Supplementary Figure 87. ^{13}C NMR (101 MHz, $CDCl_3$) spectra of compound **3al.**

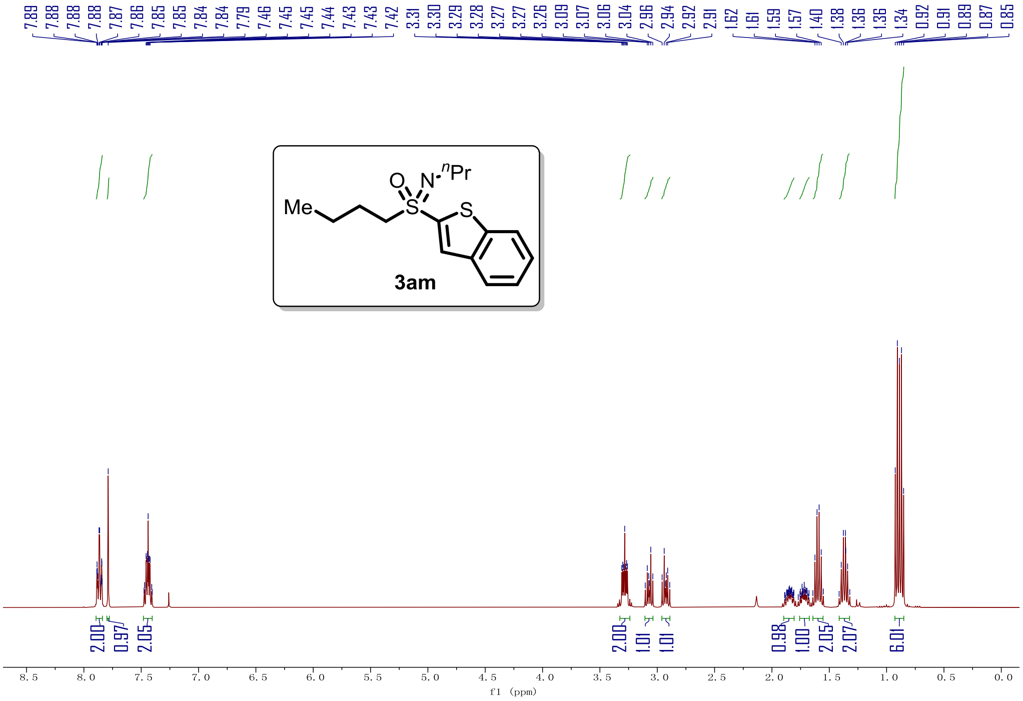

Supplementary Figure 88. ^{1}H NMR (400 MHz, $CDCl_3$) spectra of compound **3am.**

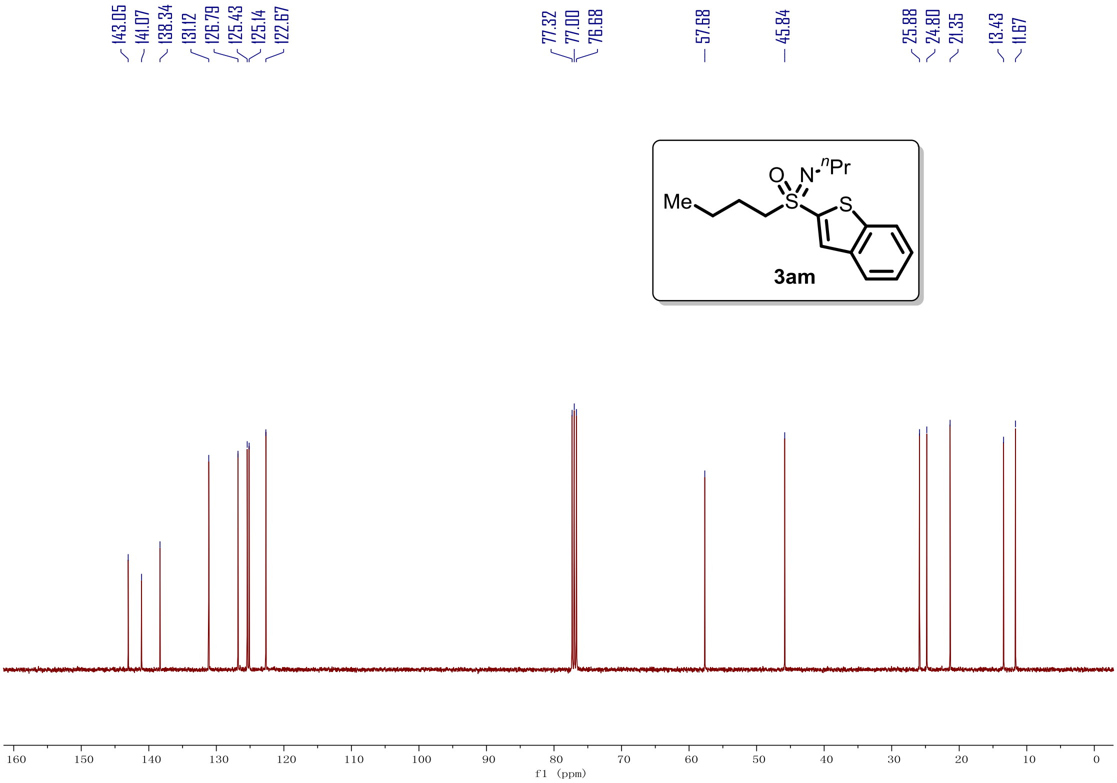

Supplementary Figure 89. ^{13}C NMR (101 MHz, $CDCl_3$) spectra of compound **3am.**

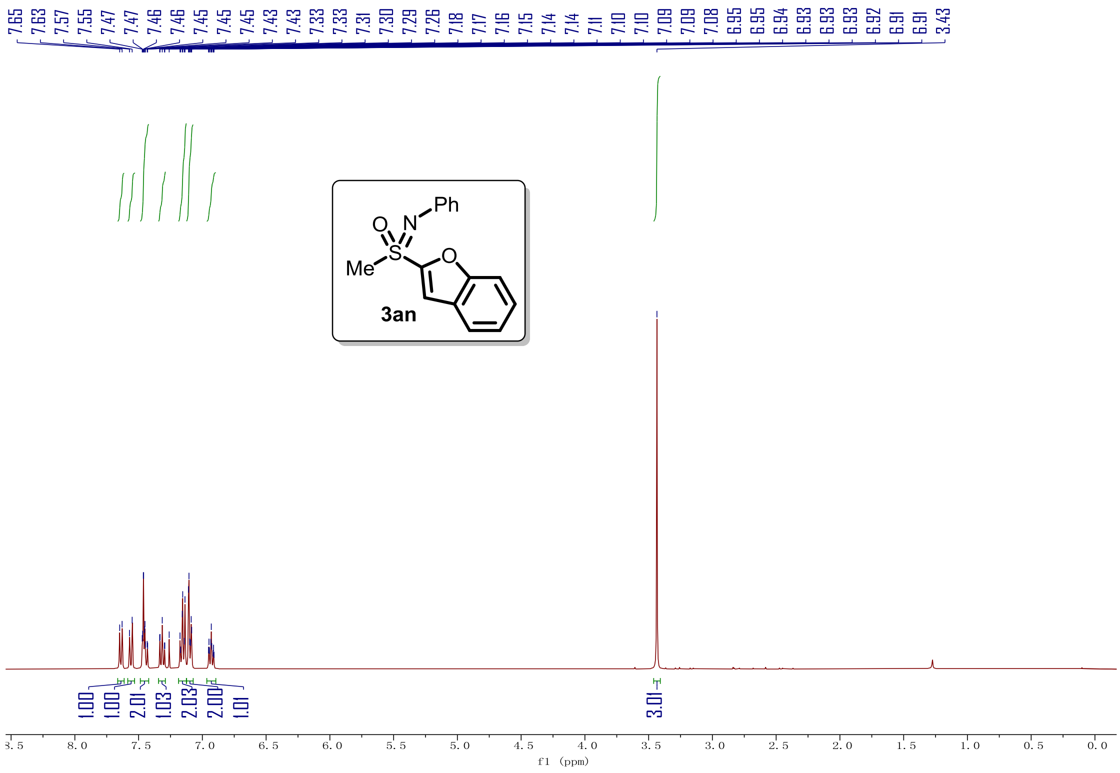

Supplementary Figure 90. ^{1}H NMR (400 MHz, $CDCl_3$) spectra of compound **3an.**

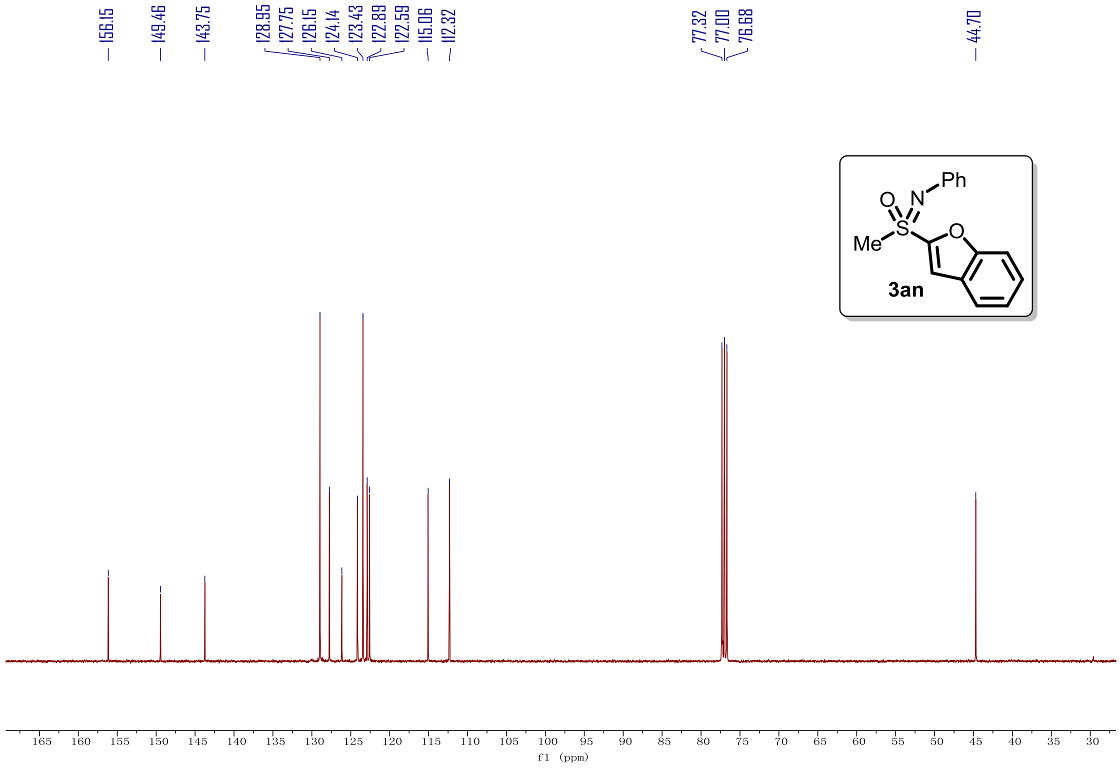

Supplementary Figure 91. ^{13}C NMR (101 MHz, $CDCl_3$) spectra of compound **3an.**

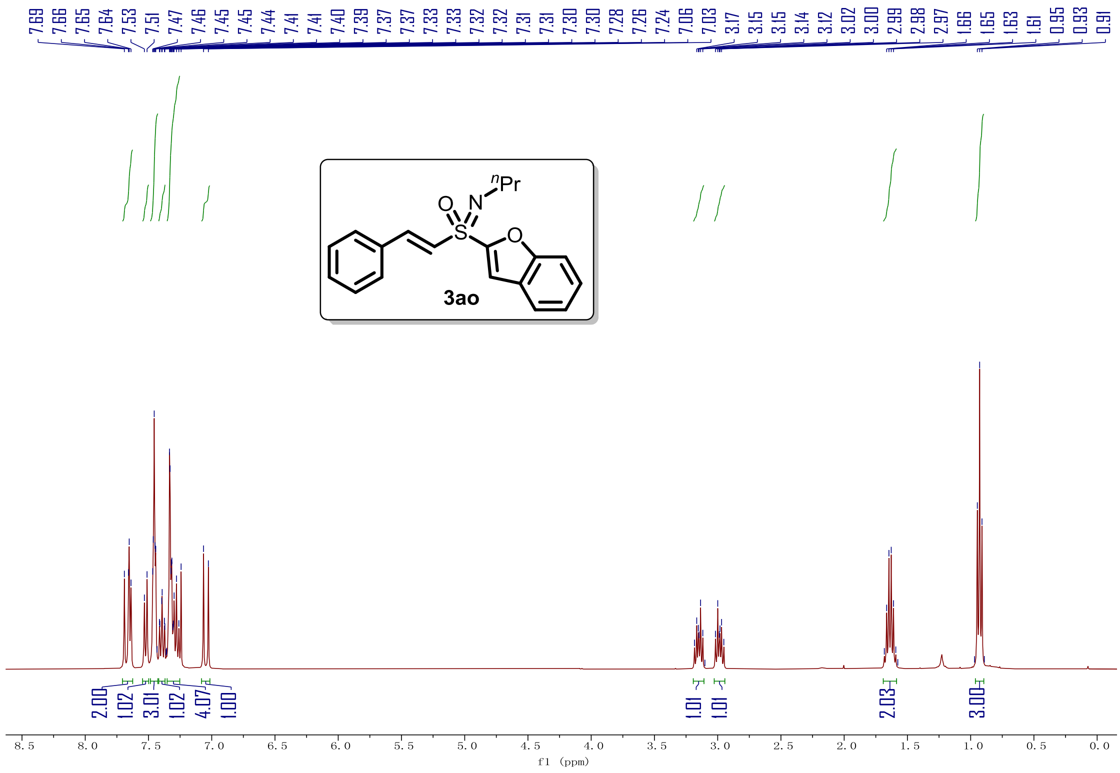

Supplementary Figure 92. ^{1}H NMR (400 MHz, $CDCl_3$) spectra of compound **3ao.**

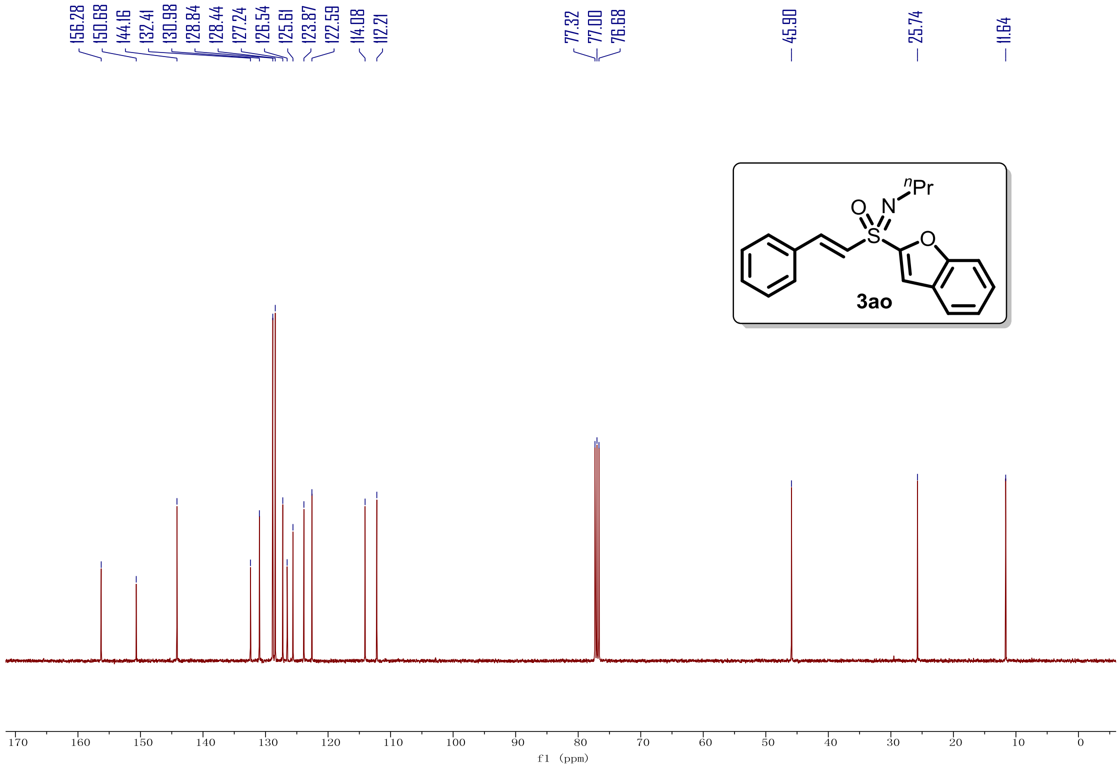

Supplementary Figure 93. ^{13}C NMR (101 MHz, $CDCl_3$) spectra of compound **3ao.**

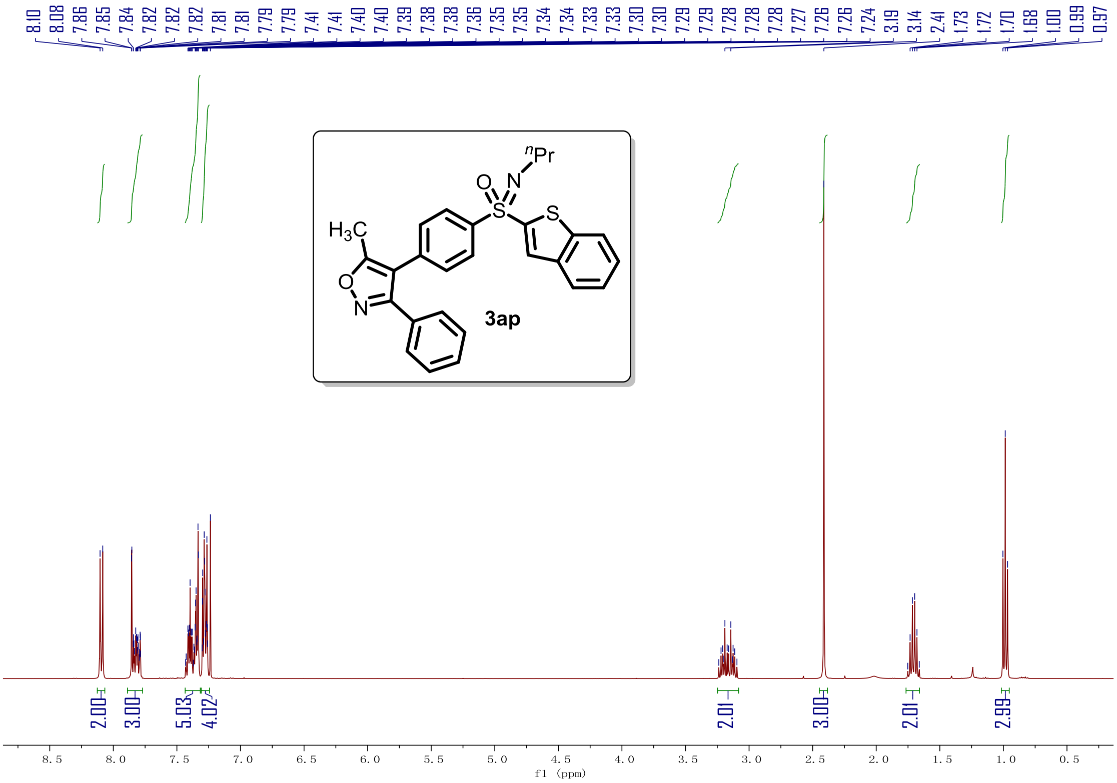

Supplementary Figure 94. ^{1}H NMR (400 MHz, $CDCl_3$) spectra of compound **3ap.**

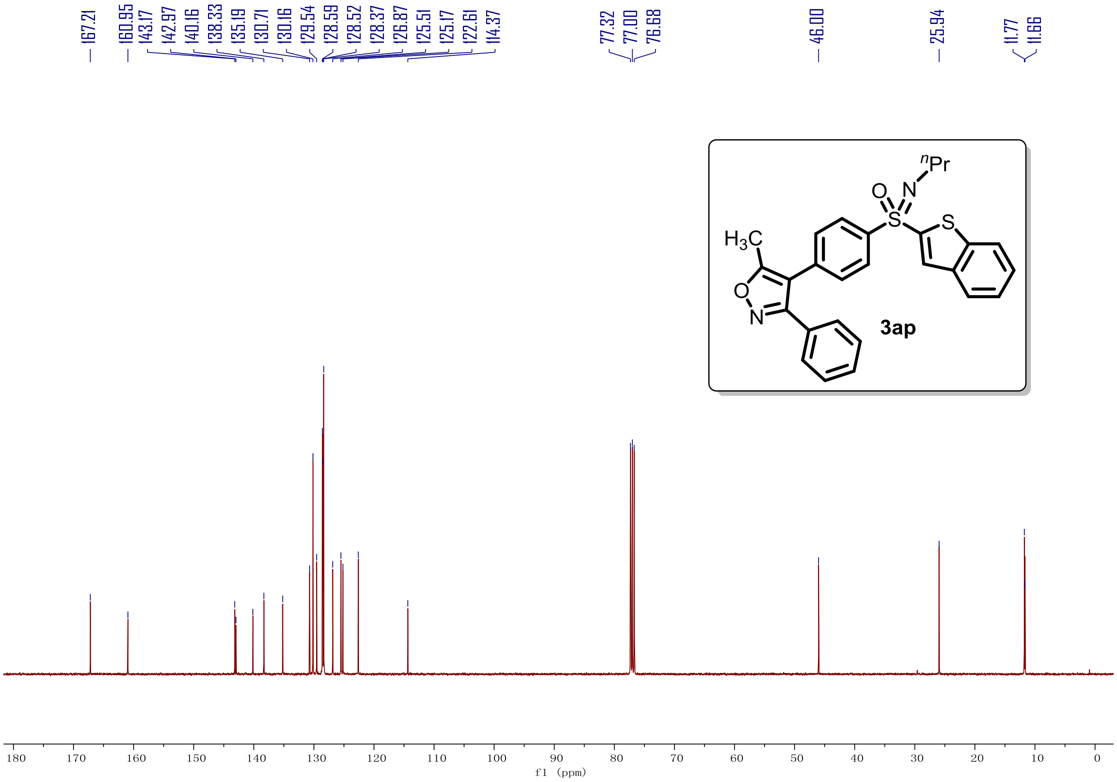

Supplementary Figure 95. ^{13}C NMR (101 MHz, $CDCl_3$) spectra of compound **3ap.**

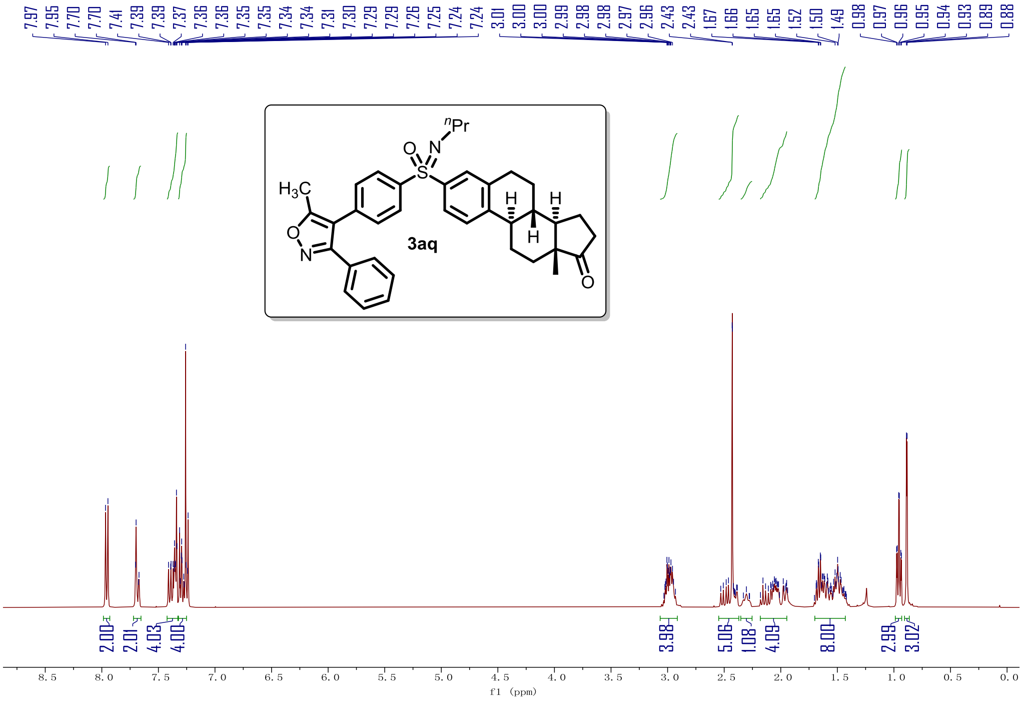

Supplementary Figure 96. ^{1}H NMR (400 MHz, $CDCl_3$) spectra of compound **3aq.**

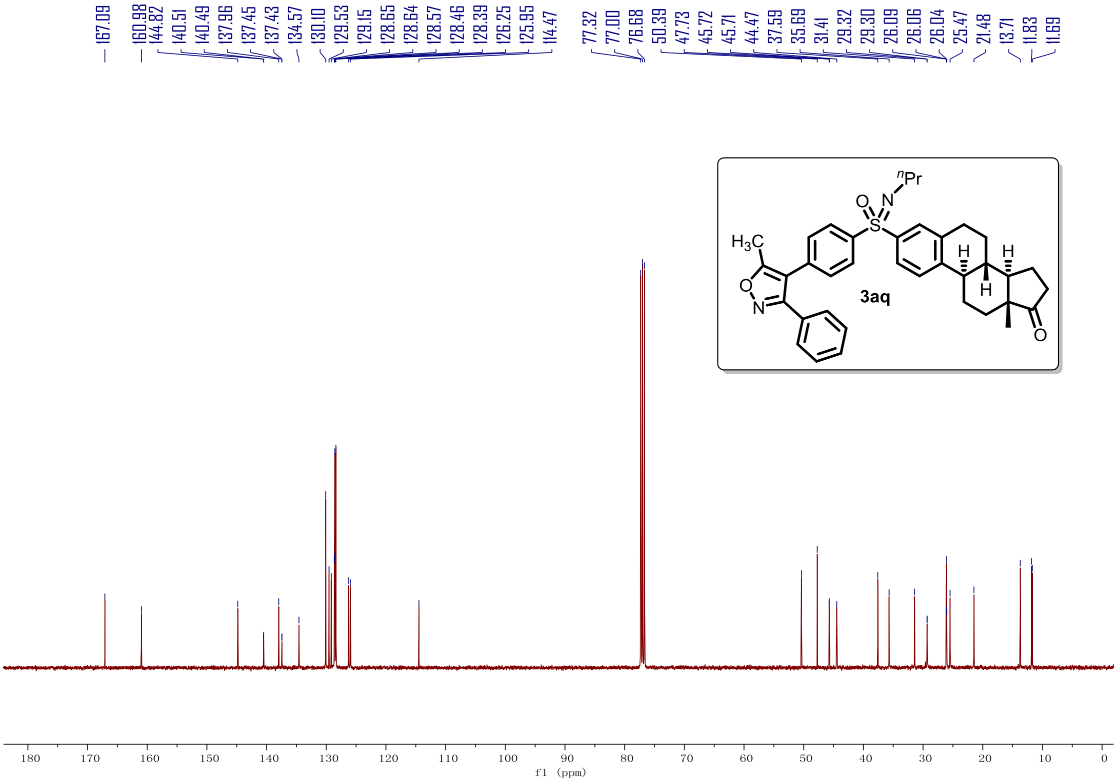

Supplementary Figure 97. ^{13}C NMR (101 MHz, $CDCl_3$) spectra of compound **3aq.**

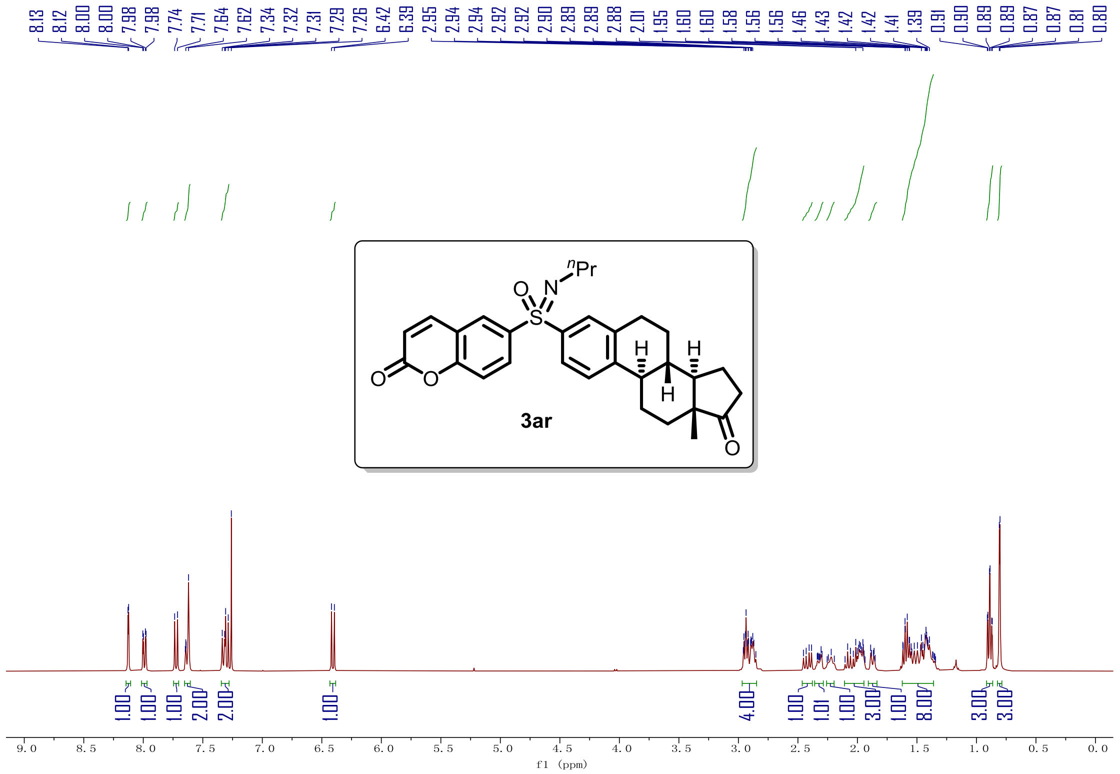

Supplementary Figure 98. ^{1}H NMR (400 MHz, $CDCl_3$) spectra of compound **3ar.**

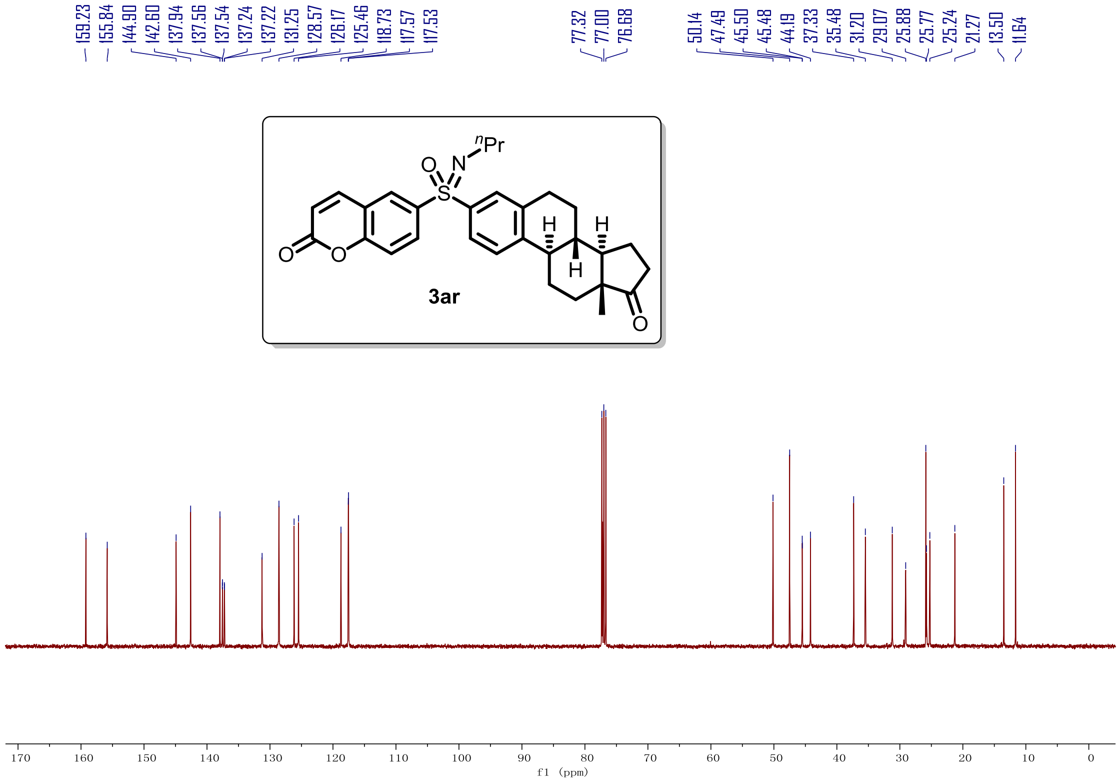

Supplementary Figure 99. ^{13}C NMR (101 MHz, $CDCl_3$) spectra of compound **3ar.**

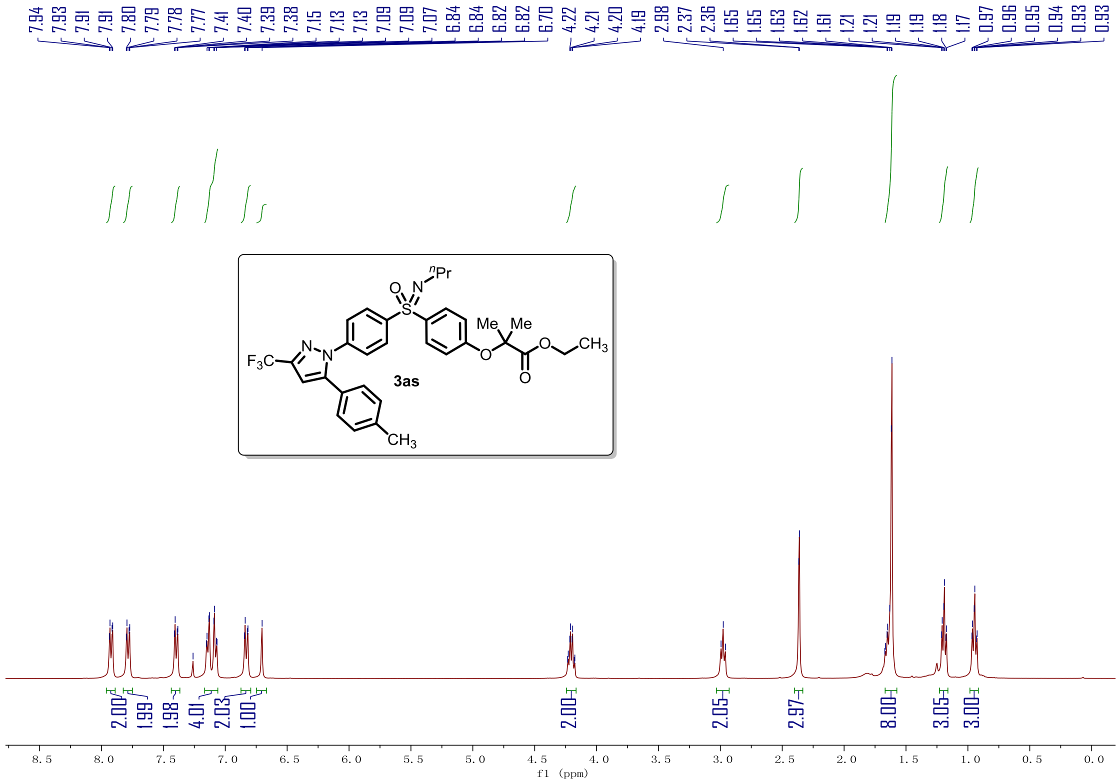

Supplementary Figure 100. ^{1}H NMR (400 MHz, $CDCl_3$) spectra of compound **3as.**

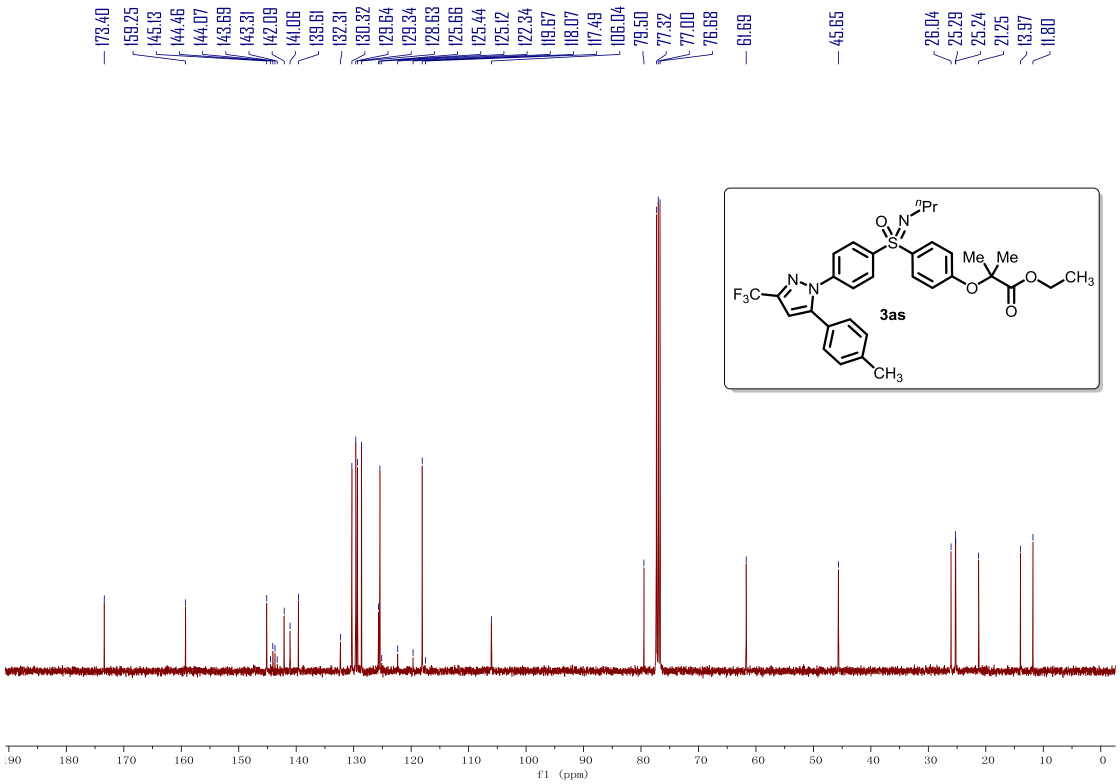

Supplementary Figure 101. ^{13}C NMR (101 MHz, $CDCl_3$) spectra of compound **3as.**

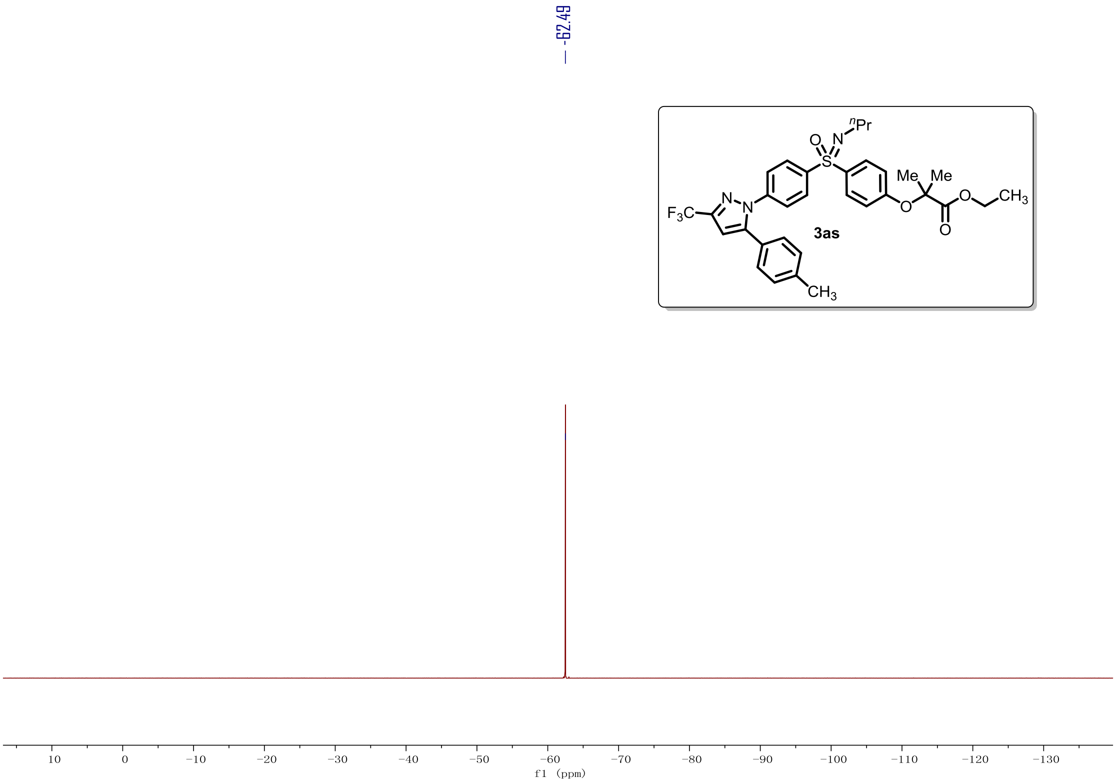

Supplementary Figure 102. ^{19}F NMR (376 MHz, $CDCl_3$) spectra of compound **3as.**

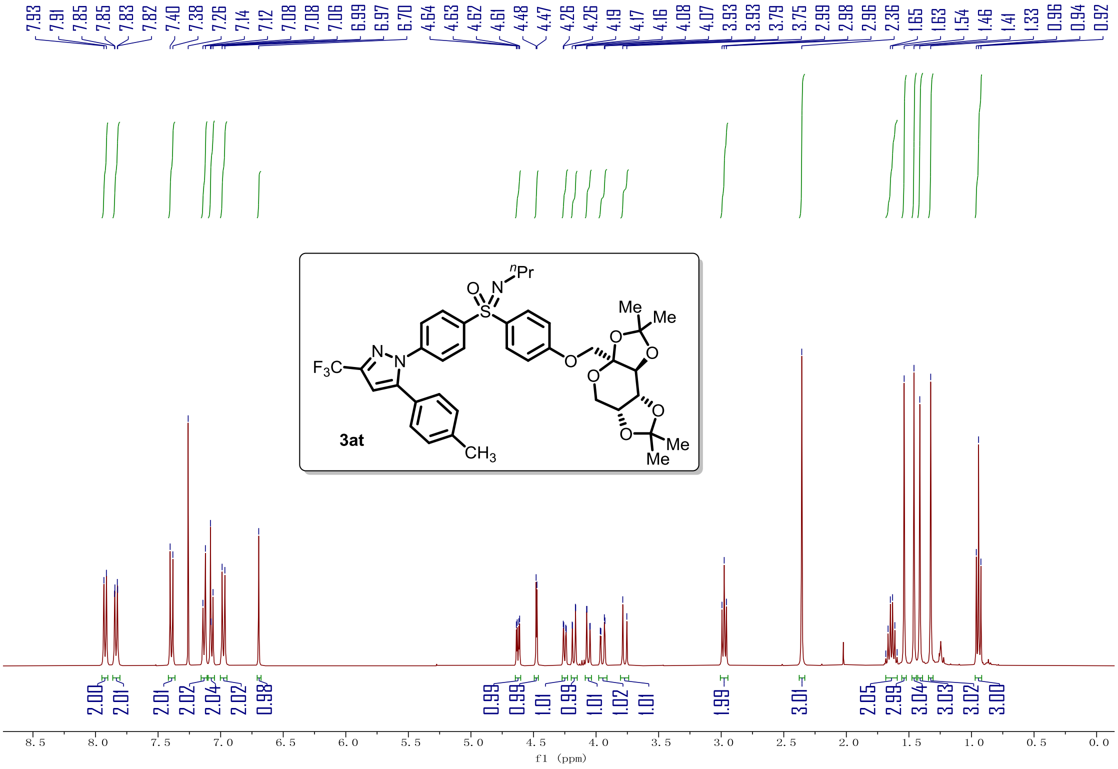

Supplementary Figure 103. ^{1}H NMR (400 MHz, $CDCl_3$) spectra of compound **3at.**

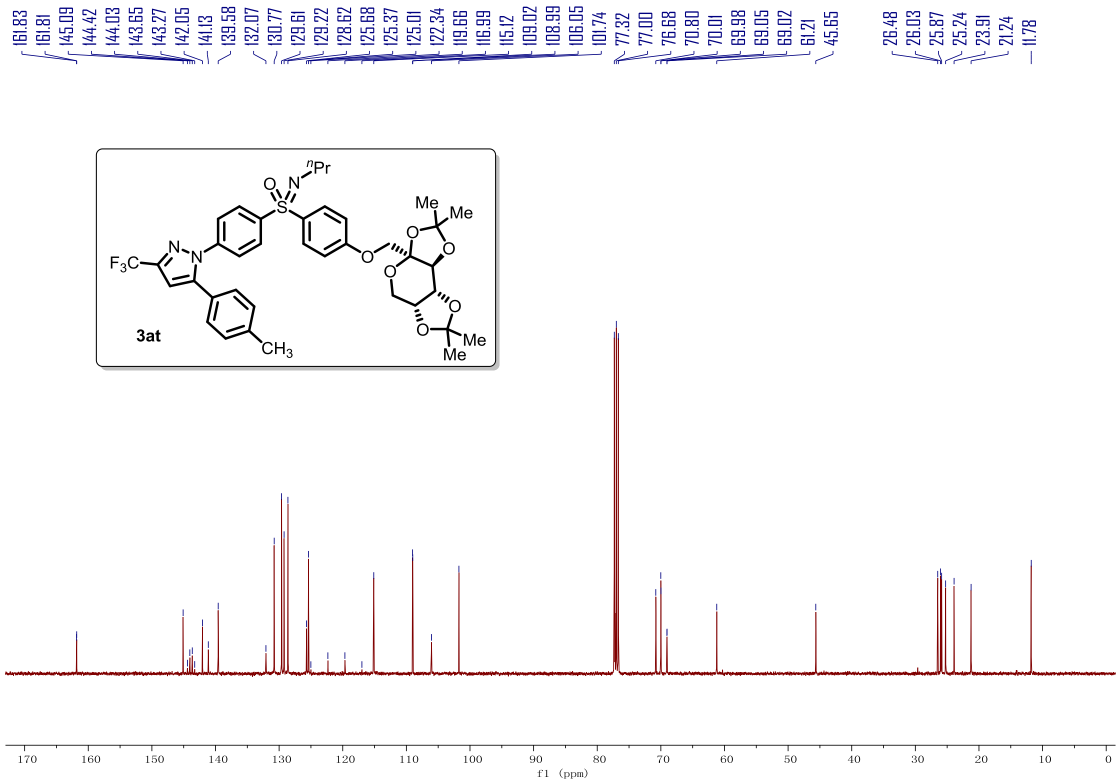

Supplementary Figure 104. ^{13}C NMR (101 MHz, $CDCl_3$) spectra of compound **3at.**

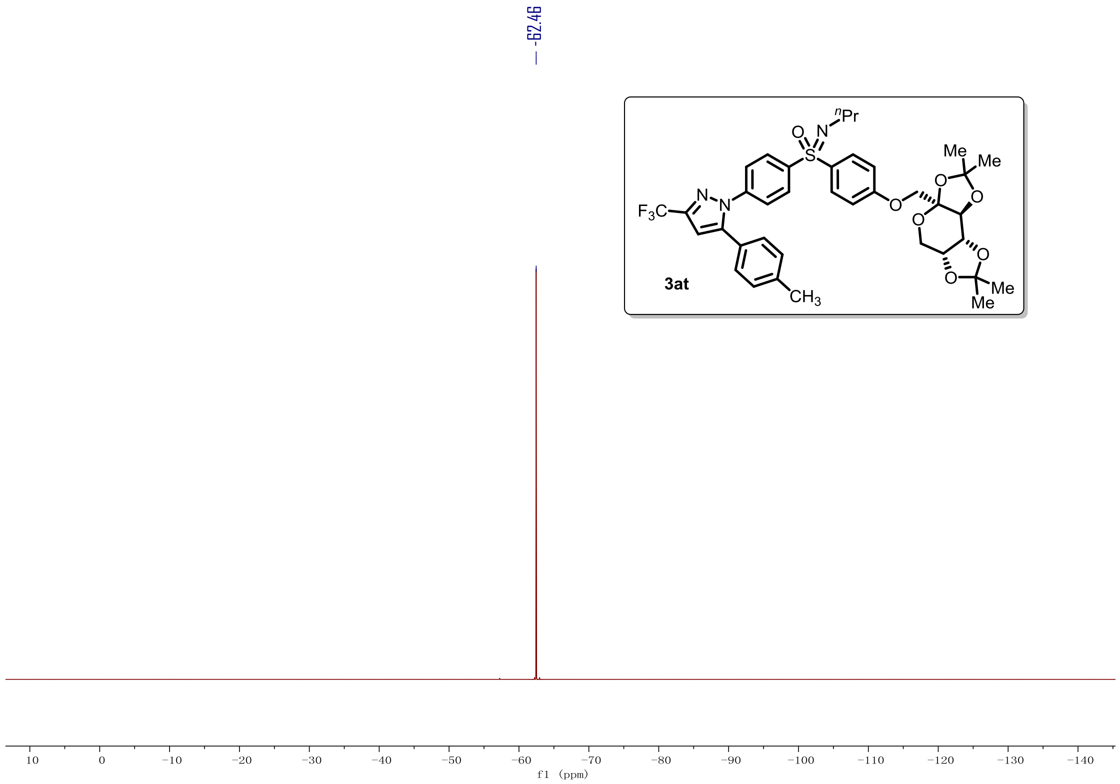

Supplementary Figure 105. ^{19}F NMR (376 MHz, $CDCl_3$) spectra of compound **3at.**

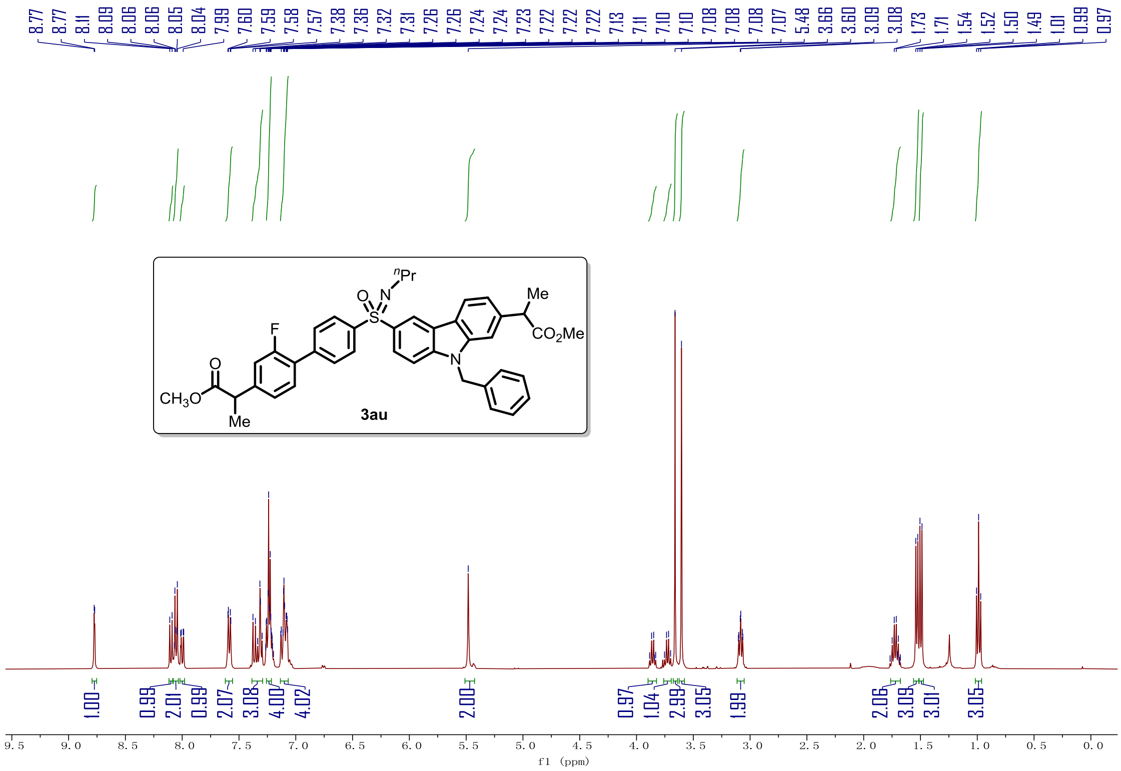

Supplementary Figure 106. ^{1}H NMR (400 MHz, $CDCl_3$) spectra of compound **3au.**

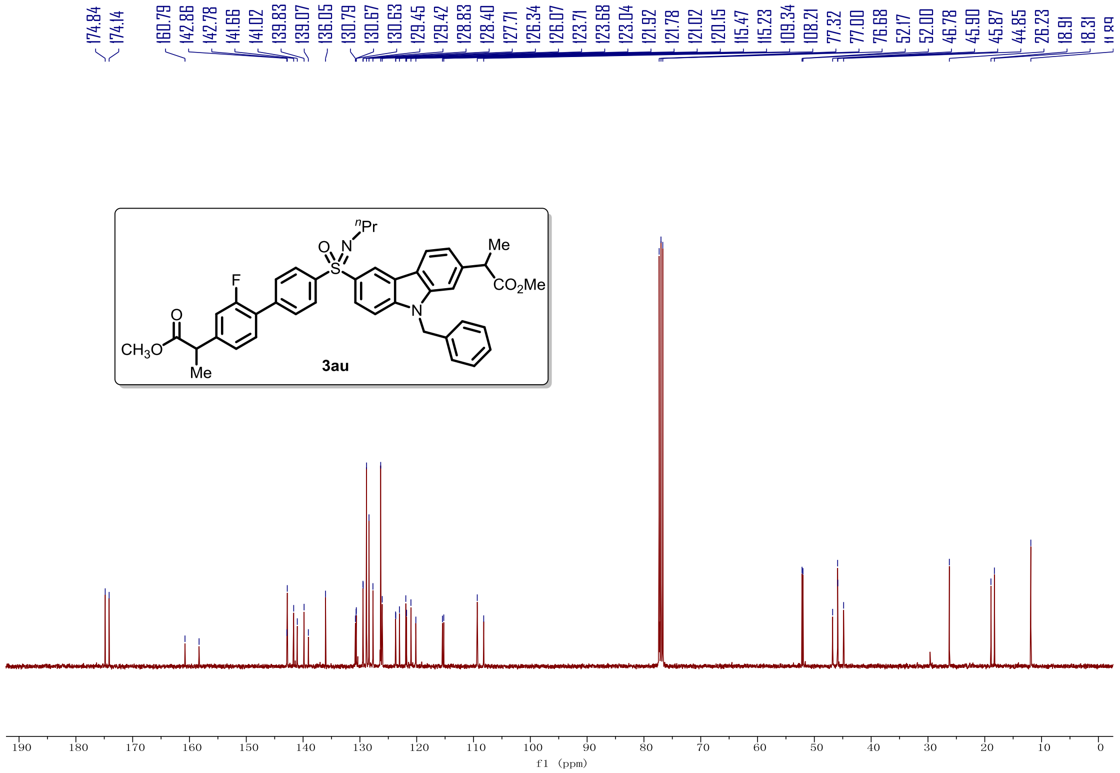

Supplementary Figure 107. ^{13}C NMR (101 MHz, $CDCl_3$) spectra of compound **3au.**

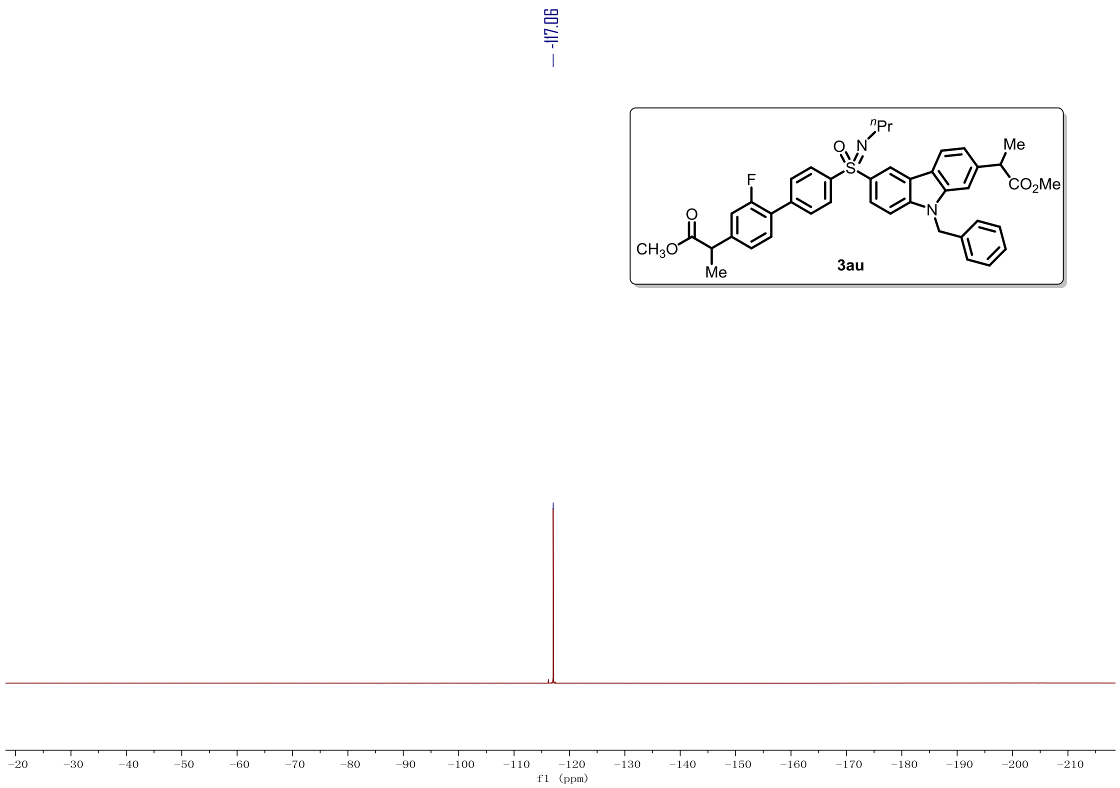

Supplementary Figure 108. ^{19}F NMR (376 MHz, $CDCl_3$) spectra of compound **3au.**

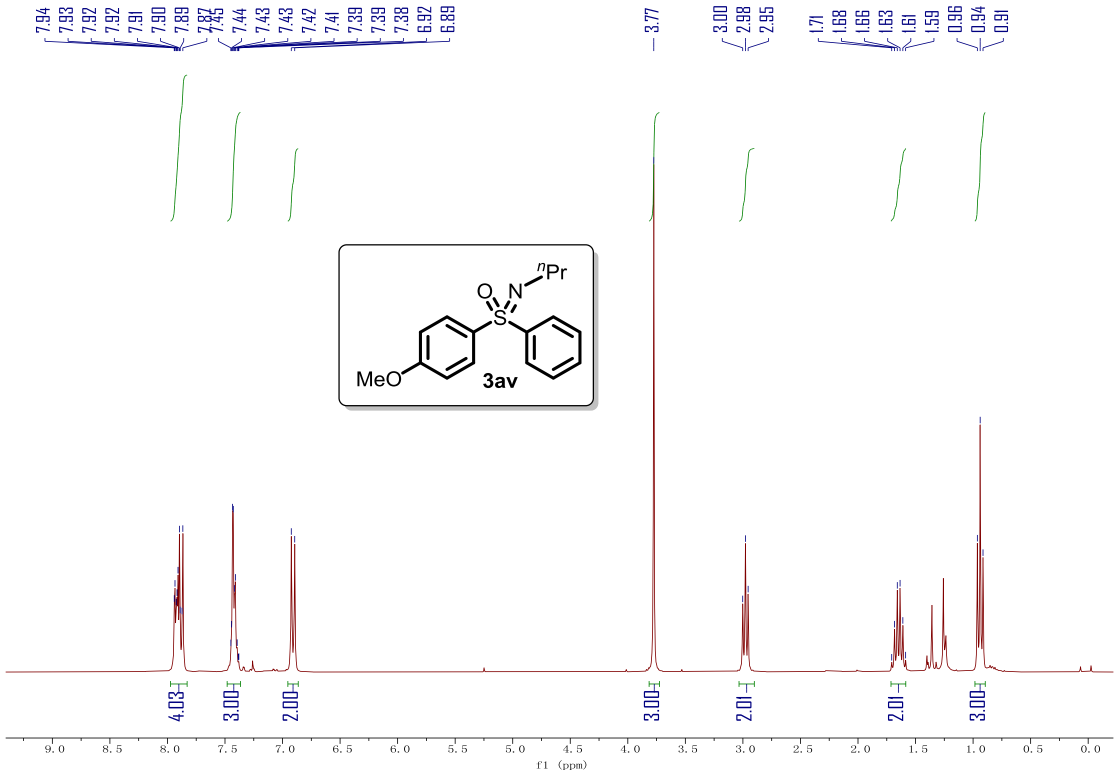

Supplementary Figure 109. ^{1}H NMR (400 MHz, $CDCl_3$) spectra of compound **3av.**

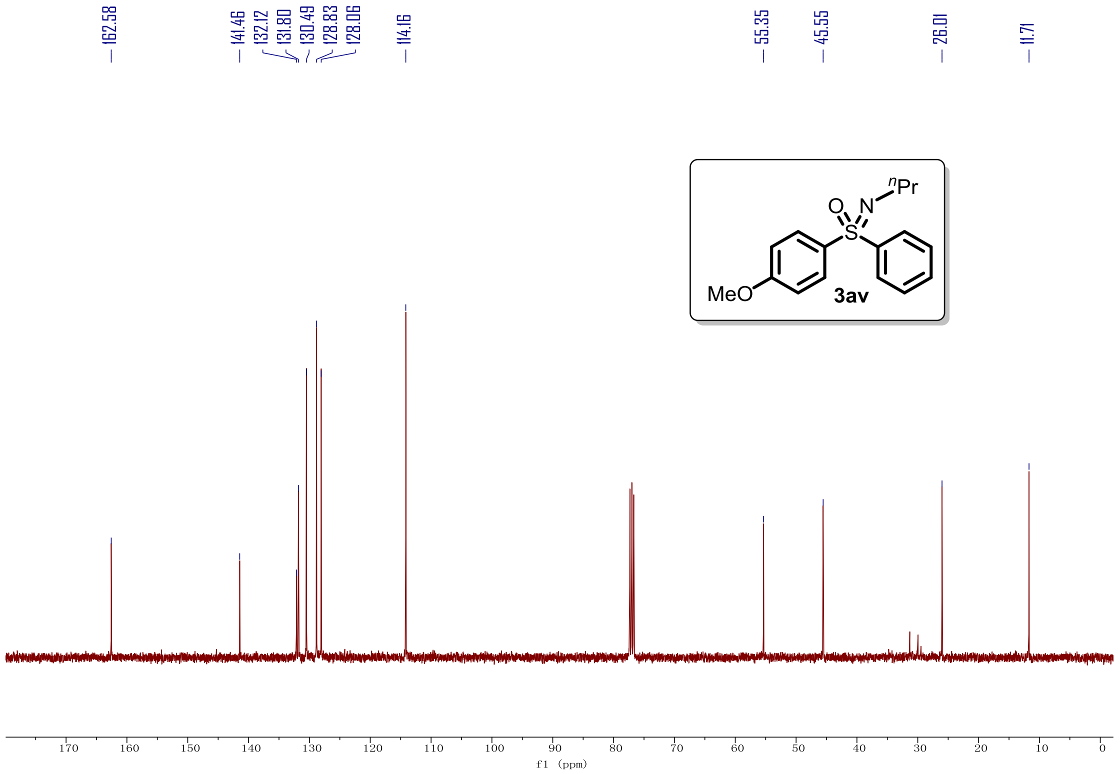

Supplementary Figure 110. ^{13}C NMR (101 MHz, $CDCl_3$) spectra of compound **3av.**

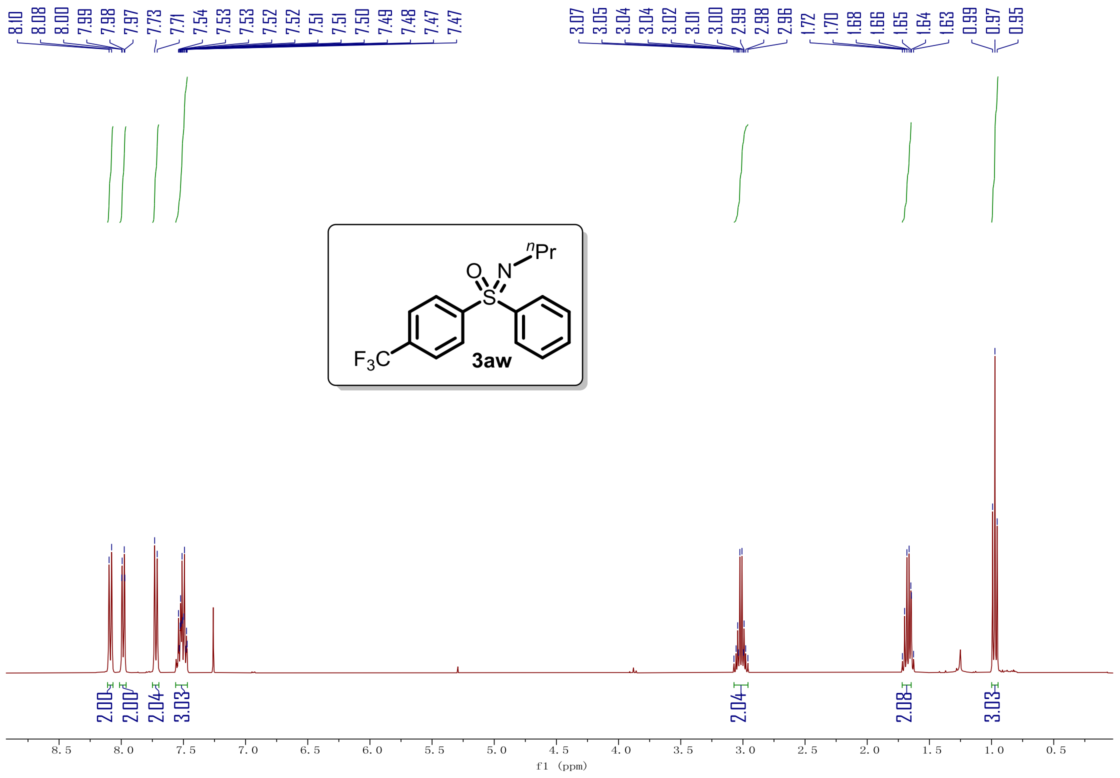

Supplementary Figure 111. ^{1}H NMR (400 MHz, $CDCl_3$) spectra of compound **3aw.**

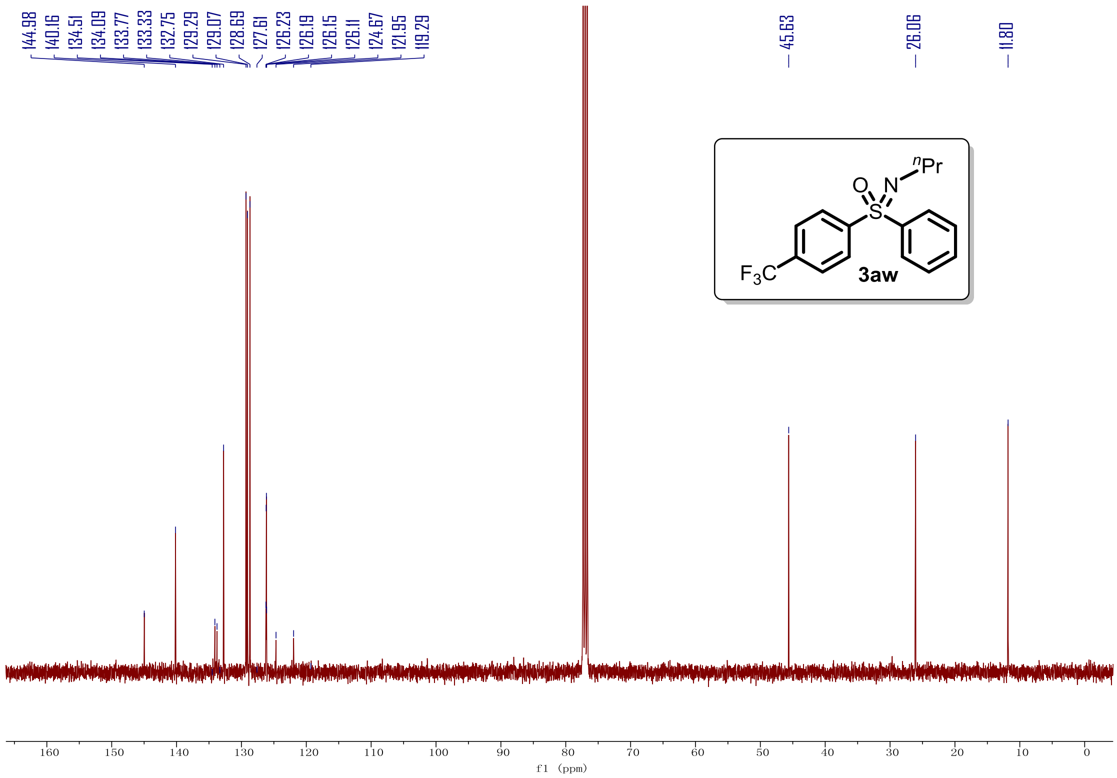

Supplementary Figure 112. ^{13}C NMR (101 MHz, $CDCl_3$) spectra of compound **3aw.**

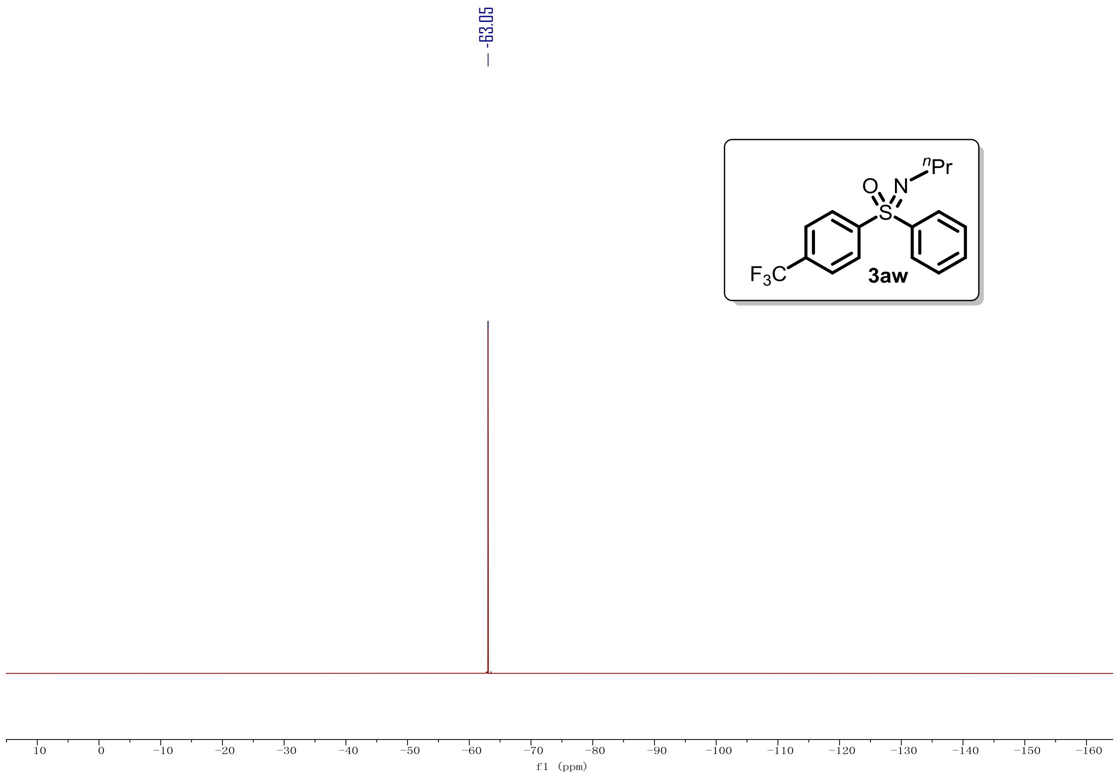

Supplementary Figure 113. ^{19}F NMR (376 MHz, $CDCl_3$) spectra of compound **3aw.**

X. References

(1) D. Zeng, Y. Ma, W.-P. Deng, M. Wang, X. Jiang, *Angew. Chem. Int. Ed.* **2022**, *61*, e202207100.

(2) D. Zeng, Y. Ma, W.-P. Deng, M. Wang, X. Jiang, *Nat. Synth*. **2022**, *1*, 455-463.

(3) Q. Gao, C. Wu, S. Deng, Q. Zhou, *J. Am. Chem. Soc.* **2021**, *143*, 7253-7260.

(4) M. J. Frisch, G. W. Trucks, H. B. Schlegel, G. E. Scuseria; M. A. Robb, J. R. Cheeseman, G. Scalmani, V. Barone, G. A. Petersson, H. Nakatsuji, X. Li, M. Caricato, A. V. Marenich, J. Bloino, B. G. Janesko, R. Gomperts, B. Mennucci, H. P. Hratchian, J. V. Ortiz, A. F. Izmaylov, J. L. Sonnenberg, D. Williams-Young, F. Ding, F. Lipparini, F. Egidi, J. Goings, B. Peng, A. Petrone, T. Henderson, D. Ranasinghe, V. G. Zakrzewski, J. Gao, N. Rega, G. Zheng, W. Liang, M. Hada, M. Ehara, K. Toyota, R. Fukuda, J. Hasegawa, M. Ishida, T. Nakajima, Y. Honda, O. Kitao, H. Nakai, T. Vreven, K. Throssell, J. A. Jr. Montgomery, J. E. Peralta, F. Ogliaro, M. J. Bearpark, J. J. Heyd, E. N. Brothers, K. N. Kudin, V. N. Staroverov, T. A. Keith, R. Kobayashi, J. Normand, K. Raghavachari, A. P. Rendell, J. C. Burant, S. S. Iyengar, J. Tomasi, M. Cossi, J. M. Millam, M. Klene, C. Adamo, R. Cammi, J. W. Ochterski, R. L. Martin, K. Morokuma, O. Farkas, J. B. Foresman, D. J. Fox, Gaussian, Inc., Wallingford CT, 2016.

(5) Z. Yan, D. G. Truhlar, *Theor. Chem. Acc.* **2008**, *119*, 525-525.

(6) A. V. Marenich, C. J. Cramer, D. G. Truhlar, *J. Phys. Chem. B.* **2009**, *113*, 6378-6396.

(7) Legault, C. Y. CYLView, 1.0b, Université de Sherbrooke, http://www.cylview.org. 2009.
